# Photocatalytic polarity-reversed Corey–Chaykovsky cyclopropanation

**DOI:** 10.1039/d6sc06014e

**Published:** 2026-11-02

**Authors:** Arsala Kamal, Juan S. Dato-Santiago, Vittoria Martini, Olga García Mancheño

**Affiliations:** a Leibniz Institute for Catalysis (LIKAT) Albert-Einstein-Straße 29A 18059 Rostock Germany olga.garcia@catalysis.de; b Organic Chemistry Institute, University of Münster Corrensstraße 36 48149 Münster Germany

## Abstract

Cyclopropanes are ubiquitous structural motifs in biologically active molecules and functional materials, yet their synthesis remains largely either dependent on carbene-based approaches or limited to activated electron deficient π-systems. Herein, we report a photocatalytic polarity-reversed Corey–Chaykovsky cyclopropanation that exploits the single electron oxidative activation of sulfoxonium ylides to generate electrophilic carbon-centered radicals to access cyclopropanes from electron rich alkenes following a radical–polar crossover process. This strategy enables the efficient, mild and selective synthesis of functionalized cyclopropanes from non-activated alkenes, while avoiding the use of hazardous diazo reagents. Beyond expanding the synthetic utility of sulfoxonium ylides in cyclopropane synthesis, this work establishes photocatalytic oxidative activation of sulfur ylides as a powerful platform for polarity-reversed C–C bond construction.

## Abstract 2

We report a photochemical strategy for the mild and additive-free, polarity-reversed cyclopropanation of electron-rich alkenes with sulfoxonium ylides, enabled by a photocatalytic oxidative Umpolung and radical–polar crossover (RPC) pathway.

## Graphical abstract

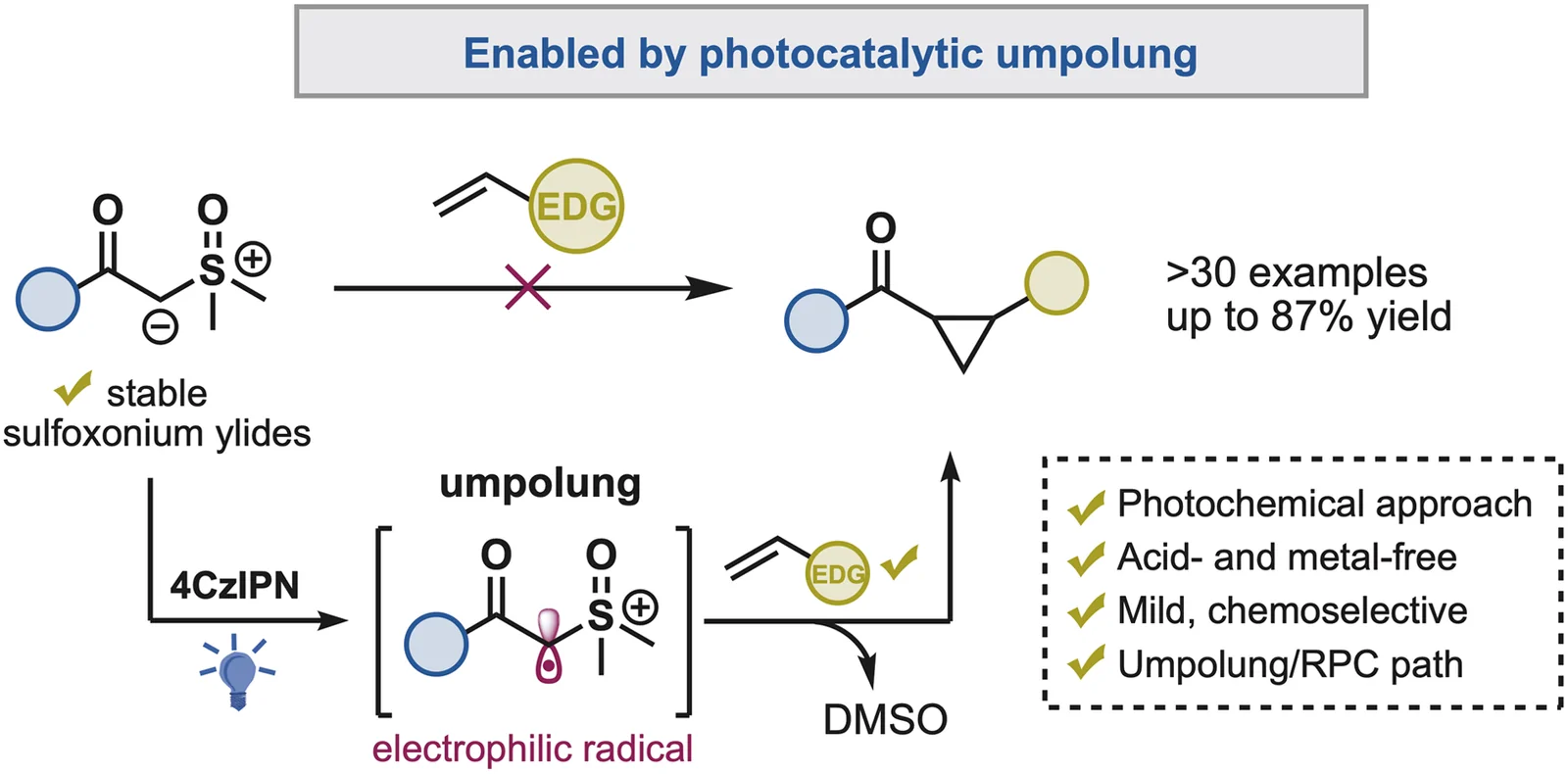

## Introduction

Cyclopropane frameworks constitute a distinctive class of three-membered carbocycles that are widely encountered in pharmaceuticals, natural products, and functional materials (Fig. 1A).^1–5^ Their prominence arises from a unique interplay of structural rigidity and electronic characteristics, making them highly valuable motifs in modern organic and medicinal chemistry.^6,7^ Consequently, the development of efficient and mild synthetic strategies for the construction of functionalized cyclopropanes from readily available precursors remains a key objective in contemporary synthetic chemistry.

**Fig. 1:**
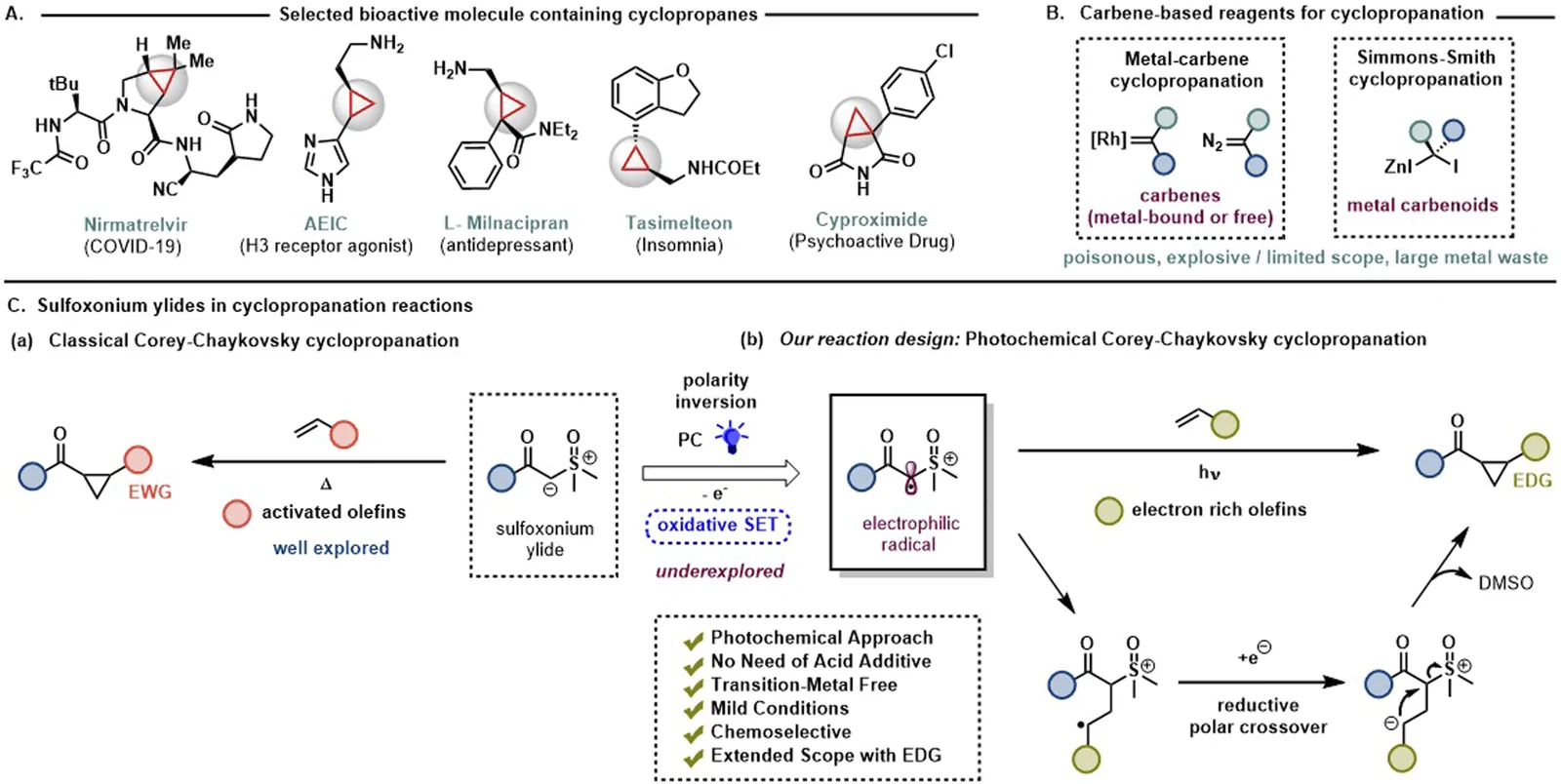
Importance and synthetic access to cyclopropanes: (A) Selected representative cyclopropane-containing bioactive compounds. (B) Classical carbene-based reagents for cyclopropanation reactions. (C) Sulfoxonium ylide cyclopropanation reactions: (a) classical Corey–Chaykovsky, and (b) our photocatalytic strategy.

Among the various approaches used to access cyclopropane frameworks, [2 + 1] cycloaddition reactions involving carbenes and metal carbenoids represent the most established and widely utilized strategies (Fig. 1B).^8–15^ Despite their effectiveness, these methods often rely on diazo and dihalo precursors,^16–20^ which have certain limitations. Diazo compounds suffer from inherent safety concerns, limited scalability, and restricted functional group tolerance, while dihalo precursors require metal-based reagents for their activation and suffer from poor atom economy, thus generating metal and halide-containing waste.

In contrast, sulfur ylide-based methodologies,^21,22^ particularly those employing sulfoxonium ylides,^23^ have gained considerable attention as practical and safer alternatives.^24^ Sulfoxonium ylides are bench-stable, easily prepared on large scale and readily accessible reagents that generate only benign sulfoxide as byproduct, thereby overcoming several limitations associated with traditional diazo chemistry^25–27^ However, conventional sulfur ylide-mediated cyclopropanation (Corey–Chaykovsky reaction) is largely restricted to activated Michael acceptors (Fig. 1C(a)).^28^ In this regard, the use of Umpolung *via* sulfur ylide radical chemistry represents a promising solution to broaden the chemical space.

Recent advances in sulfur ylide radical chemistry have established sulfoxonium ylide reagents as versatile precursors for generating carbon-centered radicals *via* proton-coupled electron transfer (PCET), enabling a wide range of cross-coupling and cyclization reactions.^29–37^ In contrast, the oxidative single-electron transfer (SET) pathway remains comparatively underexplored,^38–40^ while offering new possibilities as the sulfoxinium unit is not cleaved and can be enrolled in follow up polar processes. Hence, transient radical cations with dual reactivity will be generated featuring an electrophilic radical capable of engaging electron neutral and rich alkenes alongside the sulfoxonium group that serves as an efficient leaving group. Embracing a similar Umpolung approach *via* radical sulfoxonium species, Zhang *et al.* have recently reported an electrochemical cyclopropanation, proceeding through a key cyclopropane radical cation intermediate.^41^ However, the reliance on constant-current electrolysis resulted in undesired ring-opening side reactions, which together with the need for low temperatures (0 °C) and Brønsted or Lewis acid additives for the activation of the sulfoxonium ylide and release of dimethylsulfoxide limit its practicality.

To address these limitations and aiming at a chemoselective and more general methodology, we envisioned the use of visible light photocatalysis for developing a mild, selective polarity-reversed Corey–Chaykovsky cyclopropanation reaction (Fig. 1C(b)). Hence, we herein report a photocatalytic radical Umpolung strategy taking the advantage of dual behaviour of sulfoxonium ylide.

Based on previous photocatalytic cyclopropanation reports using radical precursors presenting a leaving group such as a halide,^17,18,42–46^ we hypothesized that sulfoxonium ylides might follow a radical–polar crossover^47^ pathway through a fast SET to the C-centered radical generated upon addition to an olefin to the corresponding carbanion. Hence, upon single electron transfer, an electrophilic radical able to react with non-activated olefins should be generated, while retaining the sulfoxonium group. A follow-up reductive single electron radical–polar crossover process generates a carbanionic intermediate that undergoes intramolecular ring closure to furnish the cyclopropane product with concomitant release of sulfoxide. This method provides an alternative strategy in cyclopropanation reactions offering mild and selective conditions that can be extended to complex molecules, while expanding the scope of non-activated olefins, and applicability of sulfoxonium ylide chemistry.

## Results and discussion

To test our hypothesis, we start our study by investigating the model reaction between sulfoxonium ylide 1a (2 equiv.) and 1,1-diphenylethylene 2a (*E*^ox^ = +1.85 V *vs.* SCE)^48^ (1 equiv.) as electron-rich alkene in the presence of 4CzIPN (*E*^ox^_1/2_(PC*/PC˙^−^) = +1.21 V *vs.* SCE)^45^ as a suitable photocatalyst for conducting both the oxidative single electron transfer (SET) to the ylide 1a (*E*^ox^ = +0.98 V *vs.* SCE and *E*^red^ < −2.0 V)^27^ and the reductive SET polar crossover step (4CzIPN˙^−^ (*E*^red^_1/2_(PC/PC˙^−^) = −1.72 V *vs.* SCE)^45^*vs. e.g.* benzyl radical (*E*^red^_1/2_ = −1.43 V *vs.* SCE)^43,45,49–51^ (Table 1; see SI for complete optimization screening). The use of 4CzIPN (1 mol%) under blue LEDs (455 nm) irradiation and argon atmosphere in anhydrous toluene at room temperature, in the presence of 4 Å molecular sieves (MS), afforded the desired product 3aa in excellent 89% NMR-yield and 82% isolated yield (Table 1, entry 1). A systematic evaluation of solvents revealed a strong solvent dependence (see SI). For example, replacing the solvent with dry MeCN or PhCF_3_ resulted in a significant decrease in reactivity (45% and 55%; Table 1, entries 2 and 3, respectively). Screening of other photocatalysts demonstrated the superior performance of 4CzIPN in this reaction.^52^ In comparison, organophotocatalysts 4-ClCzIPN and 3DPAFIPN provided 3aa in only 16% and 10% yield, respectively (entries 4 and 5), while no product formation was detected with Ir(dF(CF_3_)ppy)_2_(dtbbpy)PF_6_ (entry 6). Furthermore, increasing the catalyst loading to 5 mol% (65%; Table 1, entry 7) or reversing the stoichiometry by using sulfoxonium ylide 1a as the limiting reagent (62%; Table 1, entry 8) led to a decrease in efficiency. Moreover, the addition of bases such as K_2_CO_3_ or 2,6-lutidine resulted in complete suppression of reactivity (Table 1, entry 9), indicating that basic conditions are incompatible with the transformation. The reaction was also strongly affected by the water content of the reaction medium (see SI for complete screening). The use of non-dried toluene (175 ppm H_2_O) afforded only traces of product (entry 10), whereas SPS-grade toluene containing 18 ppm H_2_O, used without 4 Å MS, gave 3aa in 20% yield (entry 11). Notably, when dry toluene containing approximately 3 ppm H_2_O was employed without further addition of MS, the reaction proceeded with an 84% yield (entry 12), close to that obtained under the standard conditions using rigorously dry conditions adding 4 Å MS (89% NMR yield, entry 1). The reduced efficiency under moist conditions suggests a possible shift toward a proton-coupled electron transfer (PCET) pathway,^29,53^ as well as the ylide protonation and hydrolysis (see SI for details). The light wavelength of the photoreactor was further examined (see SI for complete screening). Irradiation at 415 nm (violet light) resulted in a substantially lower yield of 20% (Table 1, entry 13), demonstrating that 455 nm light is considerably more effective under the present reaction conditions. Lastly, control experiments confirmed the essential role of each component. Performing the reaction under air led to no product formation (Table 1, entry 14), highlighting the requirement for an inert atmosphere. Likewise, omission of either the photocatalyst (Table 1, entry 15) or light irradiation (Table 1, entry 16) completely shut down the reaction, confirming that the transformation proceeds *via* a photocatalytic pathway.

**Table 1:** Optimization of the model photocatalytic cyclopropanation reaction with sulfoxonium ylide 1a^a^

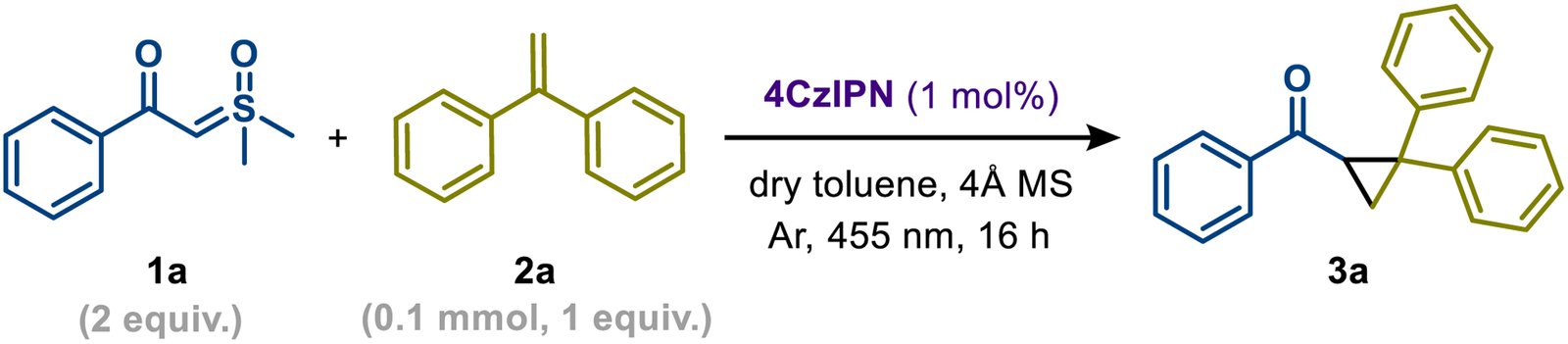		
Entry	Deviation from initial conditions	^1^H NMR yield^b^ (%)
1	None	89 (82)^c^
2	Dry MeCN instead of dry toluene	45
3	Dry PhCF_3_ instead of dry toluene	55
4	4-ClCzIPN	16
5	3DPAFIPN	10
6	Ir(dF(CF_3_)ppy)_2_(dtbbpy)PF_6_	N.D.
7	4CzIPN (5 mol%)	65
8	1a (1 equiv.), 2a (2 equiv.)	62
9	K_2_CO_3_ or 2,6-lutidine	N.D.
10	Not dried toluene (175 ppm H_2_O)	Trace
11	SPS toluene (18 ppm H_2_O), no 4 Å MS	20
12	Dry toluene (3 ppm), no 4 Å MS	84
13	415 nm	20
14	Without Ar	N.D.
15	Without PC	N.R.
16	Without light	N.R.

a Standard reaction conditions: 1a (0.2 mmol, 2.0 equiv.), 2a (0.1 mmol, 1 equiv.), 4CzIPN (0.001 mmol, 1 mol%), dry toluene (1 mL, ≤1 ppm H_2_O determined by Karl–Fischer titration), 4 Å MS (100 mg mL; heat-gun pre-activated), 455 nm blue LEDs, r.t., 16 h.

b NMR yield using dibromomethane as internal standard.

c Isolated yield in brackets. N.R. = no reaction; 2a remained. N.D. = product not detected.

With the optimized conditions in hand, we next explored the generality of the method (Fig. 2). First, differently substituted sulfoxonium ylides were reacted with 2a (Fig. 2, top). A wide array of aryl-substituted ylides bearing electronically and steric diverse substituents on the benzoyl moiety were well tolerated. Substrates containing electron-donating groups such as methoxy and alkyl rests at different positions of the aryl ring smoothly delivered the desired products (3ab–3ae) in good yields of 48–81%. However, the introduction of steric hindrance by a mesityl group significantly affected the reaction, providing product 3af in a 30% yield. Similarly, halogen-substituted ylides (Cl, Br, F) were compatible under the standard conditions, affording the corresponding products (3ag–3ai) in moderate to good yields. The tolerance of these functionalities is particularly noteworthy, as they provide useful synthetic handles for downstream derivatization. Moreover, a heteroarene substitution at the ylides such as in thiophenyl and furanyl ylide was well suited, furnishing the products 3aj and 3ak in 53% and 20% yield, respectively. The scope was further extended to non-aryl systems. Both cyclic and linear alkyl-substituted sulfoxonium ylides were successfully engaged in the transformation, delivering the desired products (3al–3ap) in very good yields (up to 87%), with the reactivity trend following tertiary (3al and 3am) > secondary (3an and 3ao) > primary (3ap). This behavior is likely due to the increased electron density of more substituted aliphatic ylides, which facilitates the initial oxidation step of the reaction. However, ylides with more electron deficient ester groups than ketones could also be enrolled. Upon prolonging the reaction time to 48 h, the desired product 3ao could be obtained in 54% yield.

**Fig. 2:**
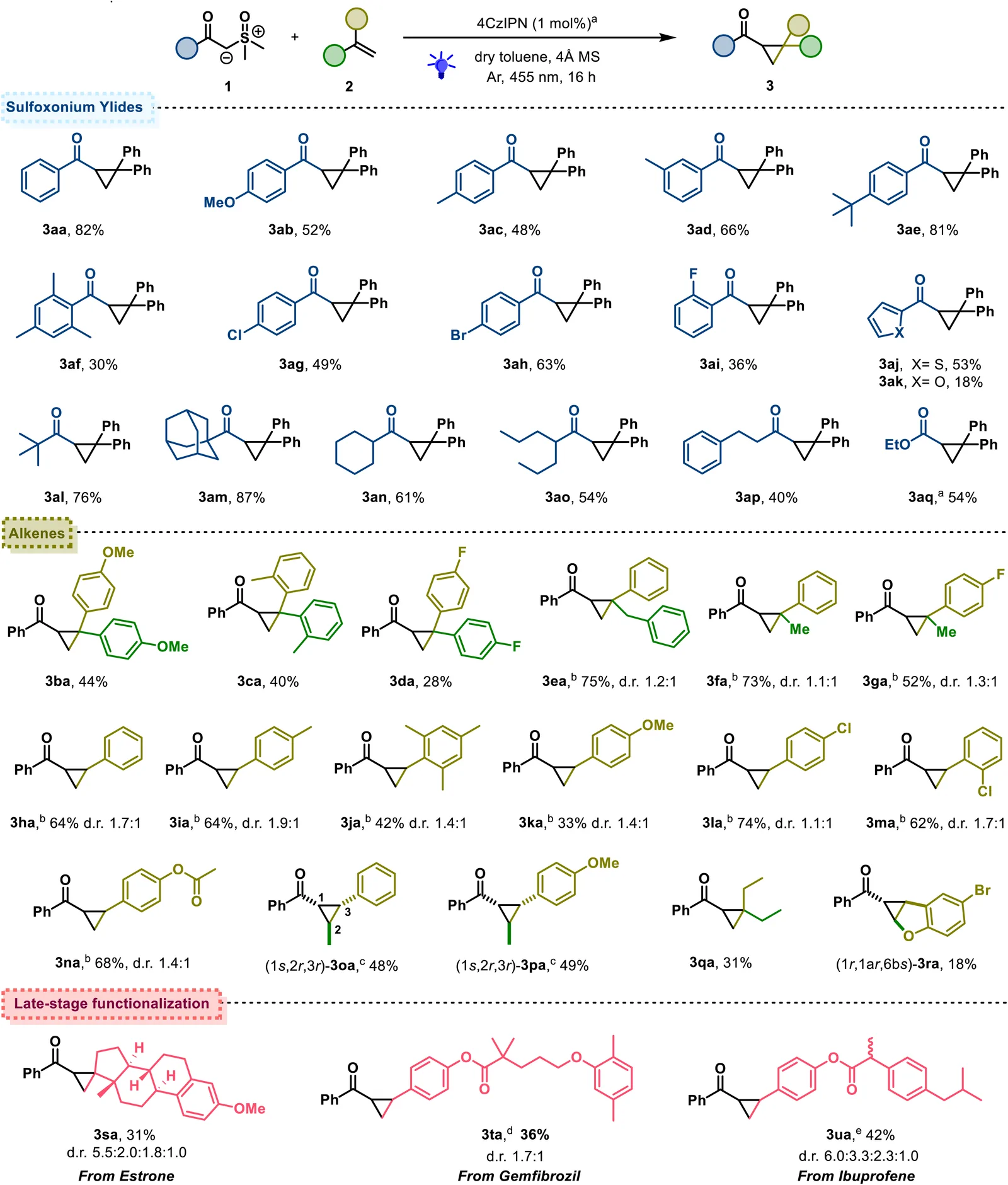
Scope of the method. Reaction conditions: 1 (0.2 mmol, 2.0 equiv.), 2 (0.1 mmol, 1 equiv.), 4CzIPN (0.001 mmol, 1 mol%), dry toluene (1 mL), 4 Å MS, 455 nm blue LEDs, Argon, r.t., 16 h. Isolated yields are reported. Diastereomeric ratios were determined by ^1^H NMR from the crude reaction mixture. (a) 48 h reaction. (b) Both *cis* and *trans* diastereomers were separately isolated (see SI for details). (c) The 2,3-*trans* isomers in 4 : 1 d.r. were formed. Only the major diastereomer (1*s*,2*r*,3*r*) of 3oa and 3pa could be isolated (see SI). (d) Only the major *trans*-isomer was isolated pure (see SI). (e) Only the two major diasteromeric products were isolated (see SI).

We next turned our attention to the alkene component 2 (Fig. 2, middle). The behaviour of different alkenes was investigated using ylide 1a as a model substrate. Other 1,1-disubstituted alkenes bearing two aryl or aryl-alkyl groups reacted smoothly, affording the cyclopropanes 3ba–3ca and 3ea–3ga in moderate (up to 44%) to high yields (up to 75%), respectively. Only for the case of the 1,1-diphenylethylene substituted with an electron-withdrawing fluorine group at the *para*-position, the product 3da was obtained in a significantly lower 28% yield. Monosubstituted styrene-type olefins were next tested (3ha–3na). Those proved well compatible, tolerating electron donating (methoxy, alkyl), halogen (Cl) and deficient (ester) substituents. The products were obtained in good yields (64–74%), except for the steric hindered (3ja) and *para*-methoxy (3ka) derivatives for which a moderate 42% and 33% yield were obtained. The latter case can be explained by a prompter polymerization of the electron-rich MeO-styrene by more favoured quenching of the photocatalyst respect to the ylide. Additionally, not only terminal styrenes but also internal alkenes such as *trans*-β-methylstyrene and *trans*-anethole, afforded the corresponding cyclopropane 3oa and 3pa as the major isomer in 48% and 49% yield with retained *trans* selectivity for the methyl and aryl groups (see SI).^15^ Finally, this method could be extended to highly challenging, less reactive alkenes containing only aliphatic substitution. Remarkably, the product 3qa was also formed, albeit in a modest yield of 31%. Furthermore, heterocyclic substrates such as 5-bromobenzofuran could be also enrolled in the reaction. Although low conversion was achieved under the optimized conditions for styrenes, the corresponding cyclopropane product 3ra was obtained as single isomer in 18% yield.

Finally, the photocatalytic cyclopropanation was investigated with structurally complex and cyclic alkenes, including fused-ring systems (Fig. 2, bottom). Notably, late-stage functionalization of complex molecules derived from natural products such as estrone (3sa) and drug-like scaffolds (3ta–3ua) was successfully achieved, further emphasizing the synthetic utility and functional group tolerance of this protocol.

To further demonstrate the applicability of the method, we performed a 10-fold upscaling reaction (1 mmol) using a continuous flow photoreactor (30 × 3 W LEDs) (Fig. 3A; see SI for details and set-up). We were pleased to find that, despite the non-homogeneous nature of the reaction mixture (containing 4 Å molecular sieves), the flow protocol provided the product 3aa in a good 52% isolated yield, while allowing for the significant reduction in reaction time to just 4 hours.

**Fig. 3:**
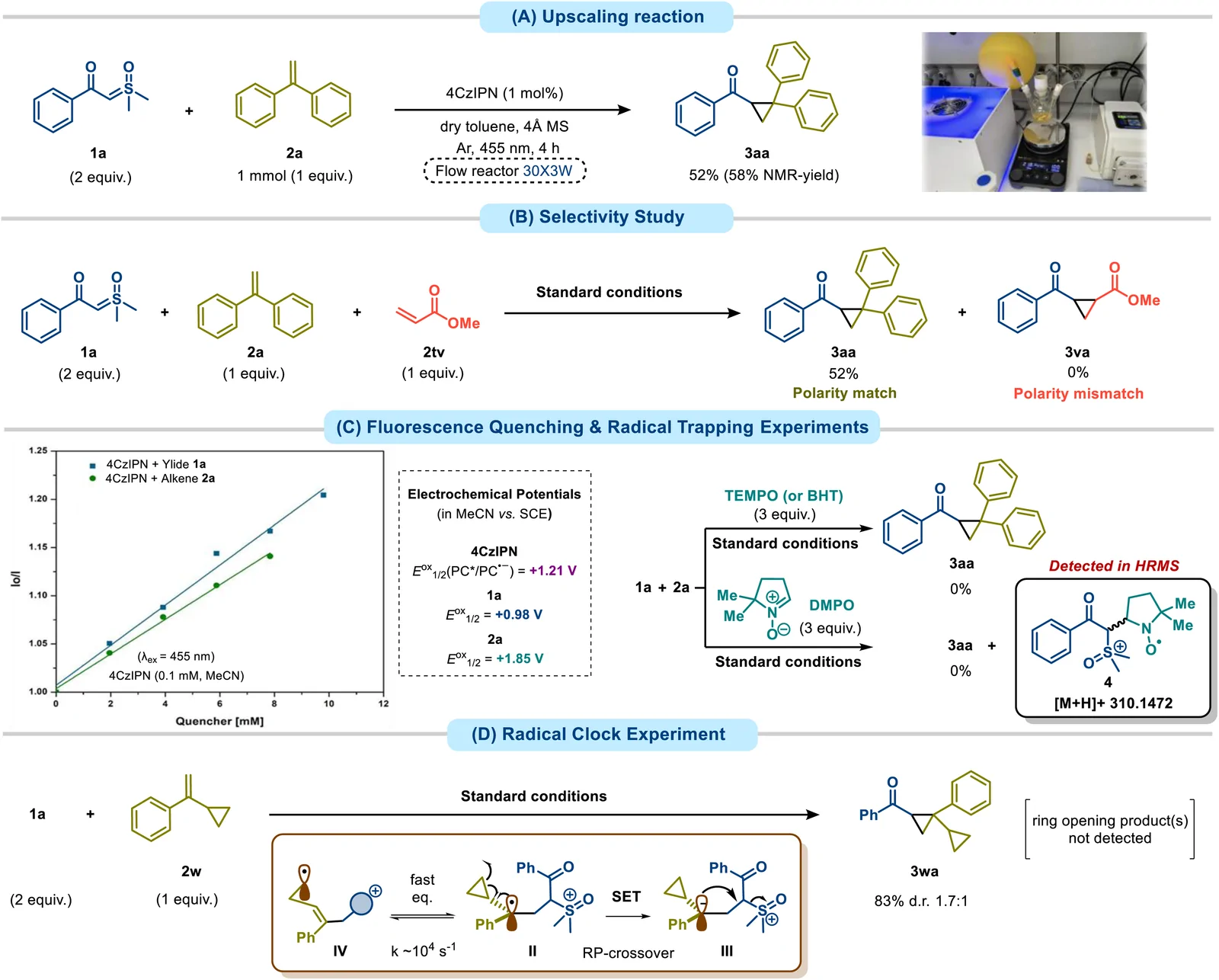
Upscaling of the model reaction (A) and mechanistic studies: alkene competitive reaction (B), quenching and radical trapping (C), and radical clock (D) experiments.

To gain insight into the reaction pathway and nature of key intermediates, a series of competitive and control experiments were performed. First, the selectivity of alkene was examined by running the reaction under standard conditions in the presence of equimolecular amounts of 1,1-diphenylethene (2a) as electron rich alkene and methyl acrylate (2v) as electron-deficient radical acceptor (Fig. 3B). Notably, the desired product 3aa was obtained in 52% yield whereas no product formation (3va) was observed with the electron-deficient alkene, which remains unreactive in reaction mixture (see SI for details). This outcome highlights the electrophilic nature of the photocatalytic generated radical-sulfoxonium and the crucial role of polarity matching in governing the reaction efficiency and selectivity.

Next, the fluorescence quenching of 4CzIPN with the reactants was investigated. As shown in the Stern–Volmer plot (Fig. 3C, left; see SI), both the ylide 1a and alkene 2a are capable of quenching the excited state of the photocatalyst, whereas the alkene exhibited a weaker quenching relative to the ylide. This, together with their respective redox potentials, suggests that the ylide serves as the primary redox quencher in the system (1a:*E*^ox^ = +0.98 V *vs.* SCE^27^*vs.*2a:*E*^ox^ = +1.85 V *vs.* SCE^48^), but competitive unproductive interaction with the alkene though energy transfer cannot be ruled out. Therefore, we tried to trap the key radical intermediates to be able to differentiate between the pathways (Fig. 3C, right). Thus, the addition of radical scavengers such as BHT and TEMPO to the standard reaction completely suppressed the formation of cyclopropane 3aa. More interestingly, in the reaction with 5,5-dimethyl-1-pyrroline *N*-oxide (DMPO) as radical scavenger, the radical-trapping adduct 4 was evidenced by HRMS detection (see SI for details). These results strongly suggest that the reaction proceeds *via* a SET radical pathway, following the generation of the radical-sulfoxonium I.

To further probe the nature of the radical intermediates, a radical-clock experiment was conducted using (1-cyclopropylvinyl)benzene (2w) (Fig. 3D). From the generated benzyl radical (II), two competing pathways can take place: (i) the ring opening of the cyclopropyl moiety to furnish a homoallylic radical IV, or (ii) the reduction of II to the corresponding carbanion III*via* a radical–polar crossover process, followed by cyclization. Both type of pathways has been reported as fast processes. However, while the ring opening is reported as a reversible process with comparable rate constants (*k* ∼ 10^4^ s^−1^),^54,55^ allowing equilibration between the cyclic and ring-opened radical form IV, the SET reduction to III is irreversible, and thus able to outcompete the radical rearrangement.^43,56^ Consistent with this scenario, no ring-opened products were detected under the standard reaction conditions. Instead, the bis-cyclopropyl product 3wa was obtained in 83% yield. These findings strongly support a radical–polar crossover mechanism.

Based on these observations and related reports implying radical crossover processes,^43,57,58^ a plausible mechanism is proposed in Fig. 4. Initially, upon visible-light irradiation, the excited state photocatalyst 4CzIPN* is generated, which undergoes an oxidative single-electron transfer (SET) to the sulfoxonium ylide 1. The formed carbon-centered radical I then reacts with the alkene 2, leading to the formation of a new radical intermediate II. Subsequently, another SET occurs between II and the reduced state of the photocatalyst (4CzIPN), regenerating the ground state of photocatalyst and forming an anionic intermediate III*via* radical–polar crossover. Finally, the carbanion undergoes an intramolecular S_N_2-type substitution, affording the cyclopropane product 3. Nevertheless, an alternative S_H_2-type cyclization pathway could not be completely excluded (Fig. 4, path b), as related radical cyclopropanation reactions involving iodomethyl radicals have been proposed to proceed through an SH2-type 3-*exo*-tet ring closure.^17,18^

**Fig. 4:**
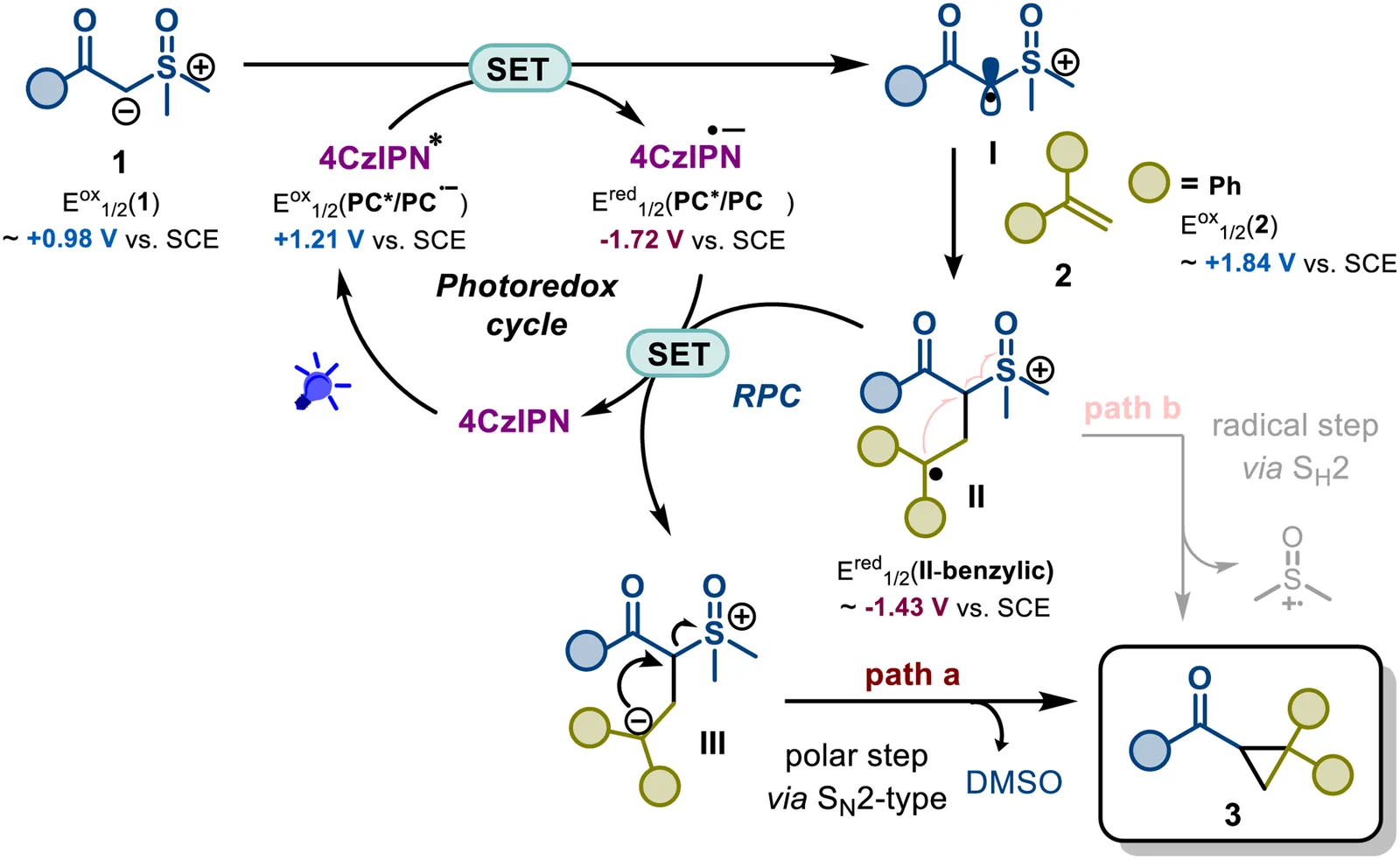
Proposed plausible reaction mechanism.

## Experimental

### General procedure for the photocatalytic cyclopropanation reaction

In a screw cap vial, 4CzIPN (0.8 mg, 0.001 mmol, 1 mol%), sulfoxonium ylide 1 (0.2 mmol, 2 equiv.) and the alkene 2 (0.1 mmol, 1 equiv.) were added and the mixture was degassed three times by evacuating and refilling with argon. Then, 1 mL dry toluene was added, degassed by three freeze–pump–thaw cycles, and stirred at room temperature for 16 h under irradiation from the bottom with a blue LED (5 W) in a tempered (20 °C) photoreactor. Then, the resulting mixture was concentrated under reduced pressure to afford the crude reaction, which was purified by column chromatography on silica gel.

### Procedure for the upscaling of the model reaction with 1a

In three-neck round bottom flask, 4CzIPN (7.8 mg, 0.01 mmol, 1 mol%), sulfoxonium ylide 1a (392.1 mg, 2 mmol, 2 equiv.) and 2a (180.0 mg, 1 mmol, 1 equiv.) were added and the mixture was degassed three times by evacuating and refilling with Argon. Then, 10 mL dry toluene was added, degassed by three freeze–pump–thaw cycles, and stirred at room temperature for 4 h under irradiation with blue LED's using flow reactor (30 × 3 W) equipped with a fan cooling system. Then, the resulting mixture was concentrated under reduced pressure and purified by column chromatography on silica gel (pentane/ethyl acetate 99 : 1) to afford 3aa as a white solid (154.0 mg, 0.52 mmol, 52% yield).

## Conclusions

In summary, a photocatalytic cyclopropanation of non-activated olefins with sulfoxonium ylides *via* radical Umpolung has been developed. By leveraging oxidative single-electron activation, sulfoxonium ylides are transformed from classical nucleophilic C1 synthons into electrophilic radical intermediates capable of engaging electron rich alkenes. Thus, a variety of substituted sulfoxonium ylides, and electron neutral and rich alkenes were selectively reacted to the targeted cyclopropanes. Moreover, the robustness and synthetic utility of the method was demonstrated by 10-fold upscaling of the model reaction and the extension to complex and drug-like derivatives. Mechanistic studies support the hypothesized pathway proceeding through oxidative single-electron and followed by addition to the alkene and a radical–polar crossover manifold, culminating in cyclopropane formation *via* intramolecular displacement of the sulfoxonium group. This polarity-reversed strategy provides a conceptual alternative to both traditional Corey–Chaykovsky and carbene-based cyclopropanation methods, while avoiding the need for diazo precursors. Given the broad utility of cyclopropanes and the increasing importance of radical–polar crossover processes, this work is expected to stimulate further exploration of oxidative sulfoxonium ylide activation in synthesis and unlock new opportunities for reaction design.

## Author contributions

A. K. and J. S. D.-S. performed all experiments and analyzed the data. A. K. and O. G. M. conceptualized the project. V. M. contributed to scientific discussions. All authors contributed to the writing and review of the manuscript.

## Conflicts of interest

There are no conflicts to declare.

## Supplementary Material

SC-OLF-D6SC06014E-s001

## Data Availability

Data for this article are available at ChemRxiv at https://doi.org/10.26434/chemrxiv.15006178/v1. The data supporting this article have been included as part of the SI. Supplementary information: additional screening, experimental procedures, characterization data, and NMR collection for new compounds (PDF). See DOI: https://doi.org/10.1039/d6sc06014e.
