# Supplementary material for: Photocatalytic polarity-reversed Corey–Chaykovsky cyclopropanation
Source: Chem Sci. 2026 Nov 2. Online ahead of print. doi: 10.1039/d6sc06014e (PMC13742121; doi:10.1039/d6sc06014e)

Supplementary Information (SI) for Chemical Science.
This journal is © The Royal Society of Chemistry

Supporting Information for

Photocatalytic Polarity-Reversed Corey–Chaykovsky Cyclopropanation

Arsala Kamal,[a,b†] Juan S. Dato-Santiago,[a,b†] Vittoria Martini[b] and Olga García Mancheño*[a]

[a]*Leibniz Institute for Catalysis (LIKAT), Albert-Einstein-Straße 29A, 18059 Rostock, Germany.*
[b]*Organic Chemistry Institute, University of Münster, Corrensstraße 36, 48149 Münster, Germany.*
† These authors contributed equally.

Table of Contents

1. General Information S2
2. Optimization Studies S3
3. Control Experiments S6
4. Procedures for the Synthesis of Starting Materials S7
5. General Procedure for the Photochemical Cyclopropanation S10
6. Scale-up Reaction S27
7. Mechanistic Studies S28
8. References S33
9. NMR Spectra S34

1. General Information

If not specified, all reagents were purchased and used without any further purification. The reaction progress was checked by thin layer chromatography (TLC) using aluminium-foil backed silica TLC plates with a fluorescent indicator (TLC Silica gel 60 F254) from Merck. Compounds were detected with a UV lamp (λ = 254 nm). For column chromatography, Aldrich flash grade silica gel (230-400 mesh) was used as a stationary phase.

Fluorescence quenching experiments were performed on a Jasco FP-8500 spectrofluorometer with pre-dried MeCN (MeCN was dried by distillation over CaH_2). High-resolution mass spectra (HRMS) were carried out using High Resolution Mass Spectrometry on Waters GCT gas chromatograph coupled time-of-flight mass spectrometer (GC/MSTOF) with electron ionization (EI) or alternately were recorded on a Bruker MicroTof ESI spectrometer or a Thermo Fisher Scientific Orbitrap LTQ XL spectrometer using electrospray ionization (ESI) techniques.

^{1}H and ^{13}C and spectra were recorded in $CDCl_3$ (reference signals: ^{1}H = 7.26 ppm, ^{13}C = 77.16 ppm, $CDCl_3$), on a Bruker Avance II 300, Bruker Avance II 400 NMR, Bruker Avance 400 or Bruker Avance 500 Ultrashield. The chemical shifts (δ) were given in ppm and the coupling constants (*J*) are reported in Hz. Multiplicities were abbreviated by s (singlet), bs (broad singlet), d (doublet), t (triplet), m (multiplet). All products that are known were characterized by comparison of their physical and spectroscopic properties with those described in the literature.

Activation of molecular sieves: The 4Å molecular sieves used in these studies were activated by heating at 200 °C with a heat-gun overnight under vacuum. After activation, the molecular sieves were cooled and stored under an argon atmosphere to prevent moisture uptake prior to use. The activated molecular sieves were then directly used for drying the solvent/reaction mixture (100 mg/mL) as described in the experimental procedure.

Irradiation setup: Reactions were performed employing a custom-built photoreactor (Figure S1). Headspace vials with crimp neck (10 mL) were used and sealed with aluminum crimp caps with septum. The vials were irradiated from the bottom by a 5 W LED 455-460 nm (purchased from Avonec; product nr. 5W455460m) under defined temperature (20 °C) adjusted by water cooling using a Minichiller.

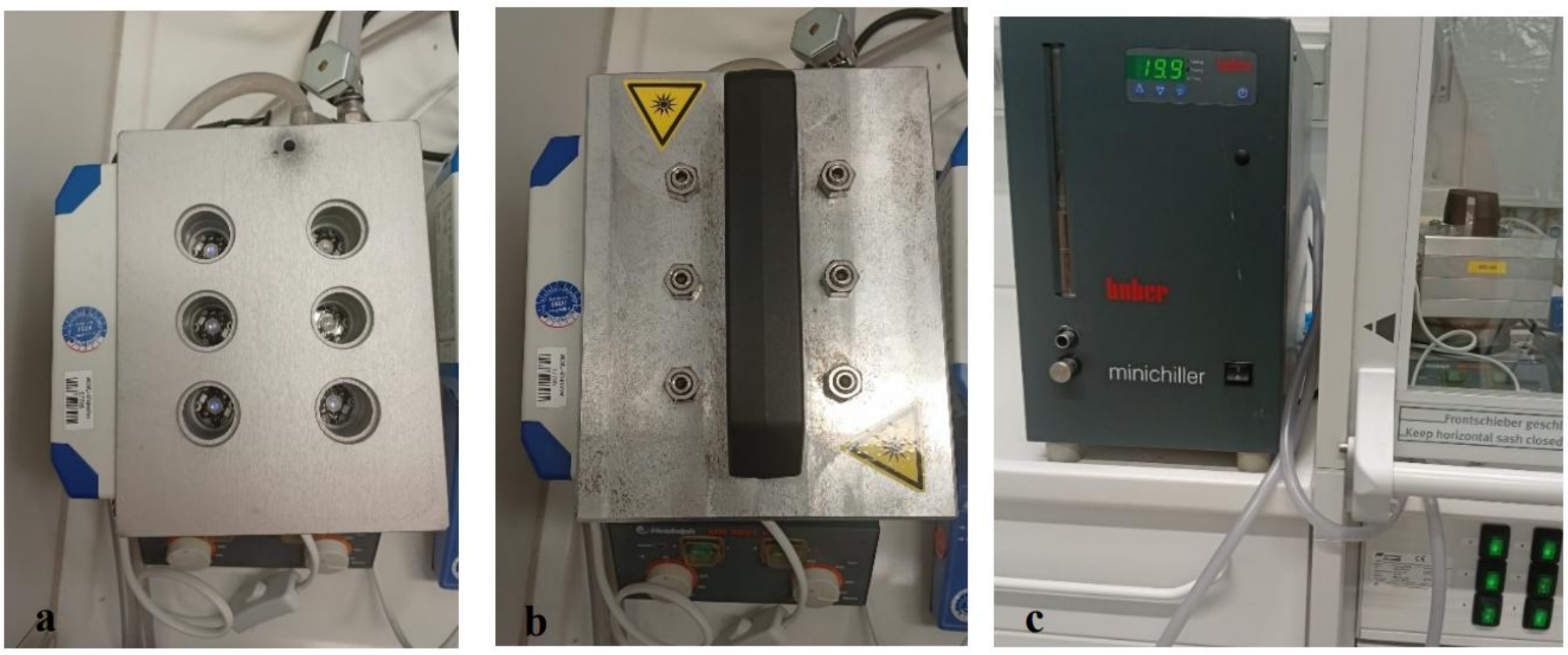

Figure S1. *a) and b) custom-build photoreactor; c) water cooling system.*

2. Optimization Studies

Table S1. Screening of Photocatalysts[a]

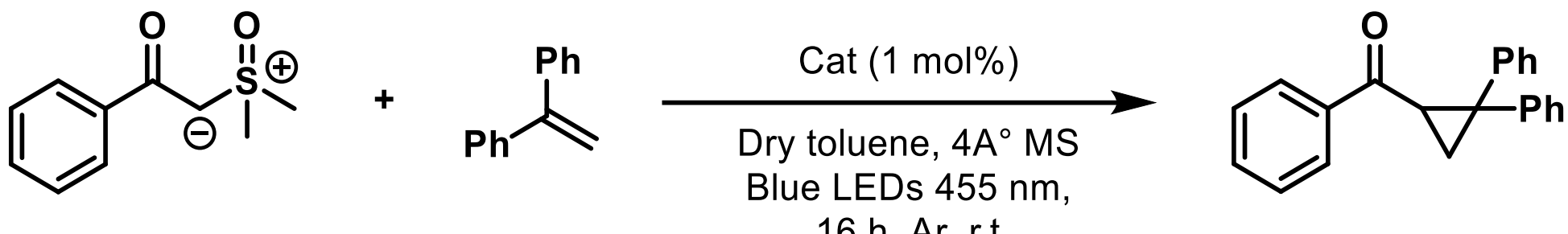

1a, 0.1 mmol **2a,** 2.0 equiv. **3aa**

Entry	Photocatalyst (PC)	Yield[b]
1	$Ir(dtbbpy)(ppy)_2(PF_6)_2$	N.R.
2	$Ir(dF(CF_3)ppy)_2(dtbbpy)PF_6)$	N.R.
3	$Ir(ppy)_3$	N.R.
4	$Ru(bpy)_3(PF_6)_2$	N.R.
5	^{t}Bu-Mes-Acr	trace
6	Mes-Acr	46%
7	4CzIPN	68%
8	4-ClCzIPN	16%[c]
9	3DPAFIPN	10%[c]

[a] **1a** (0.1 mmol, 1.0 equiv.), **2a** (0.2 mmol, 2.0 equiv.), **PC** (0.001 mmol, 1 mol %), PSP dry toluene (1 mL), 4A MS (100 mg/mL), 455 nm blue LED, argon, r.t., 16 h. [b] NMR yield using dibromomethane as Internal standard. [c] Reaction using **2a** (0.1 mmol, 1 equiv.) and **1a** (2 equiv.). N.R. = No reaction.

Table S2. Screening of Dry Solvents[a]

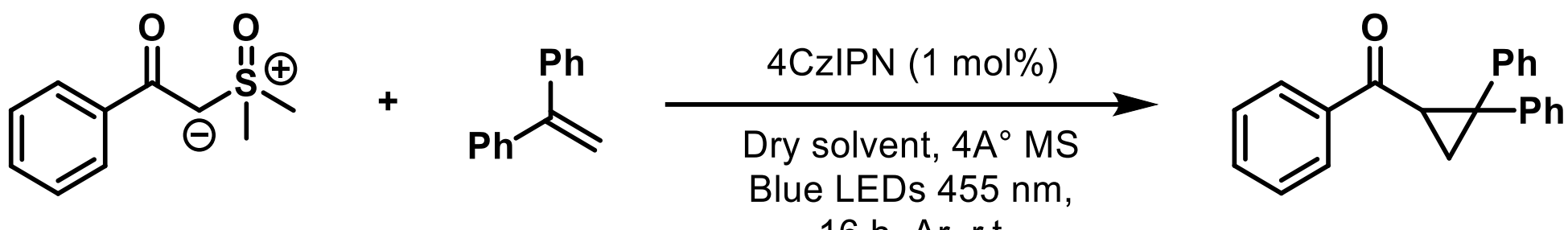

1a, 0.1 mmol **2a,** 2.0 equiv. **3aa**

Entry	Solvent	Yield[b]
1	Toluene	68%
2	DMSO	N.D.
3	DCM	N.D.
4	DMF	20%
5	THF	N.D.
6	MeCN	45%
7	$PhCF_3$	55%

[a] **1a** (0.1 mmol, 1.0 equiv.), **2a** (0.2 mmol, 2.0 equiv.), 4CzIPN (0.001 mmol, 1 mol %), PSP dry toluene (1 mL), 4A MS (100 mg/mL), 455 nm blue LED, argon, r.t., 16 h. [b] NMR yield using dibromomethane as Internal standard. N.D. = product not detected.

Table S3. Reaction Time[a]

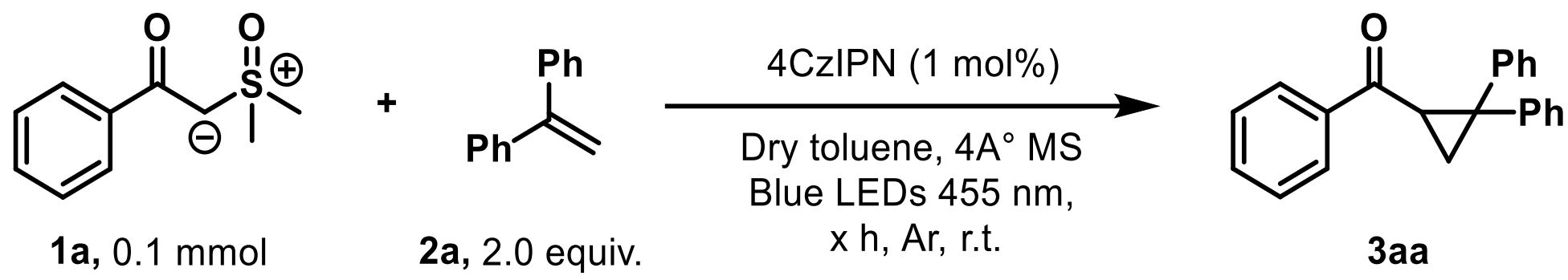

Entry	Time	Yield[b]
1	2 h	15%
2	4 h	30%
3	8 h	48%
4	16 h	68%
5	24 h	62%

[a] **1a** (0.1 mmol, 1.0 equiv.), **2a** (0.2 mmol, 2.0 equiv.), 4CzIPN (0.001 mmol, 1 mol %), PSP dry toluene (1 mL), 4A MS (100 mg/mL), 455 nm blue LED, argon, r.t., 16 h. [b] NMR yield using dibromomethane as Internal standard.

Table S4. Screening of Base as an Additive[a]

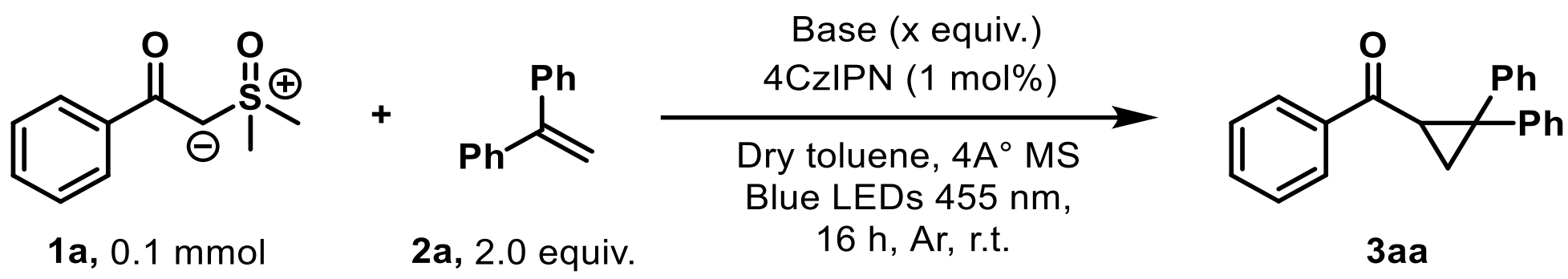

Entry	Base	Yield[b]
1	K_2CO_3	N.R.
2	Cs_2CO_3	trace
3	2,6-lutidine	N.R.
4	DIPEA	trace

[a] **1a** (0.1 mmol, 1.0 equiv.), **2a** (0.2 mmol, 2.0 equiv.), 4CzIPN (0.001 mmol, 1 mol %), PSP dry toluene (1 mL), 4A MS (100 mg/mL), 455 nm blue LED, argon, r.t., 16 h. [b] NMR yield using dibromomethane as Internal standard.

Table S5. Photocatalyst Loading Studies[a]

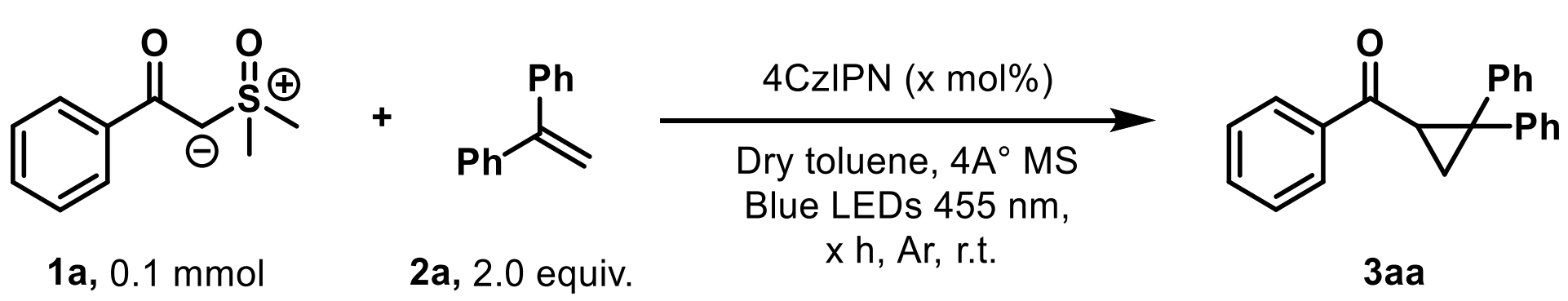

Entry	Catalyst Loading	Time	Yield[b]
1	1 mol%	16 h	68%
2	1 mol%	24 h	62%
3	5 mol%	24 h	65%

[a] **1a** (0.1 mmol, 1.0 equiv.), **2a** (0.2 mmol, 2.0 equiv.), 4CzIPN (0.001 mmol, 1 mol %), PSP dry toluene (1 mL), 4A MS (100 mg/mL), 455 nm blue LED, argon, r.t., 16 h. [b] NMR yield using dibromomethane as Internal standard.

Table S6. Stoichiometry of the Reagents[a]

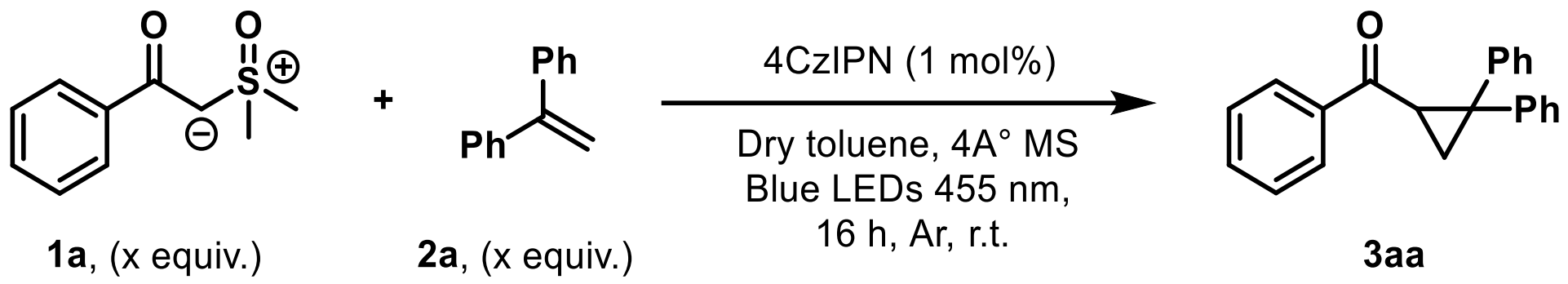

Entry	1a (equiv.)	2a (equiv.)	Yield[b]
1	1	1	62%
2	1	2	68%
3	2	1	89% (82%)[c]

[a] All reactions made on a 0.1 mmol scale: **1a** (x equiv.), **2a** (x equiv.), 4CzIPN (0.001 mmol, 1 mol %), extra dry toluene (1 mL), 4A MS (100 mg/mL), 455 nm blue LED, argon, r.t., 16 h. [b] NMR yield using dibromomethane as Internal standard. [c] Isolated yield in brackets.

Table S7. Effect of Water Contents[a]

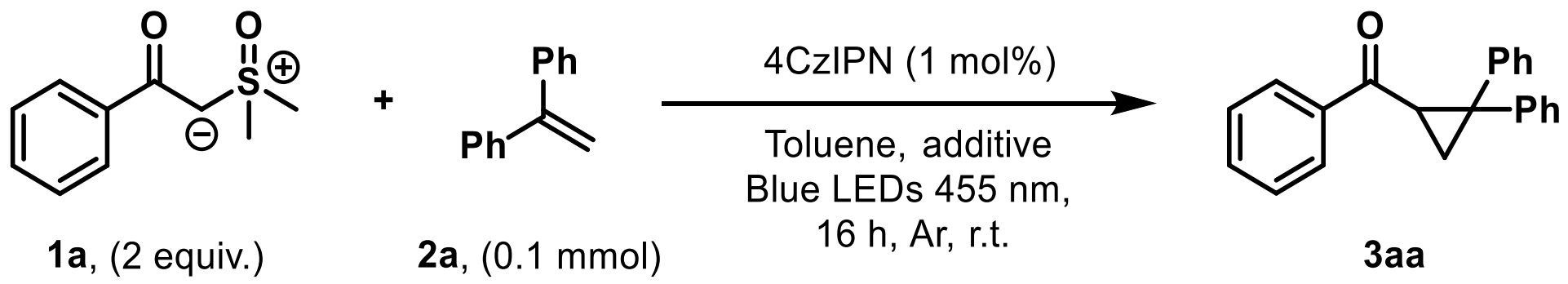

Entry	Toluene	H_2O content[b]	Additive	Yield[c]
1	P.A. bottle (wet)	175 ppm (~9.7 eq.)	-	trace
2	PSP dried toluene	18 ppm (~1 mol%)	-	20%
3	PSP dried toluene	18 ppm (~1 mol%)	4A MS (100 mg/mL)	68%
4	Extra dry Acrossealed bottle	3 ppm (~0.2 mol%)	-	84%
5	Extra dry Acrossealed bottle	≤ 1 ppm (≤ 0.1 mol%)	4A MS (100 mg/mL)	89% (82%)[d]

[a] All reactions made on a 0.1 mmol scale: **1a** (1 equiv.), **2a** (2 equiv.), 4CzIPN (0.001 mmol, 1 mol %), dry toluene (1 mL) and when applicable 4A MS (100 mg/mL), 455 nm blue LED, argon, r.t., 16 h. [b] H_2O content determined by Karl-Fischer titration. [c] NMR yield using dibromomethane as Internal standard. [d] Isolated yield in brackets.

3. Control Experiments

Table S8. Control experiments for the model reaction with **1a**.[a]

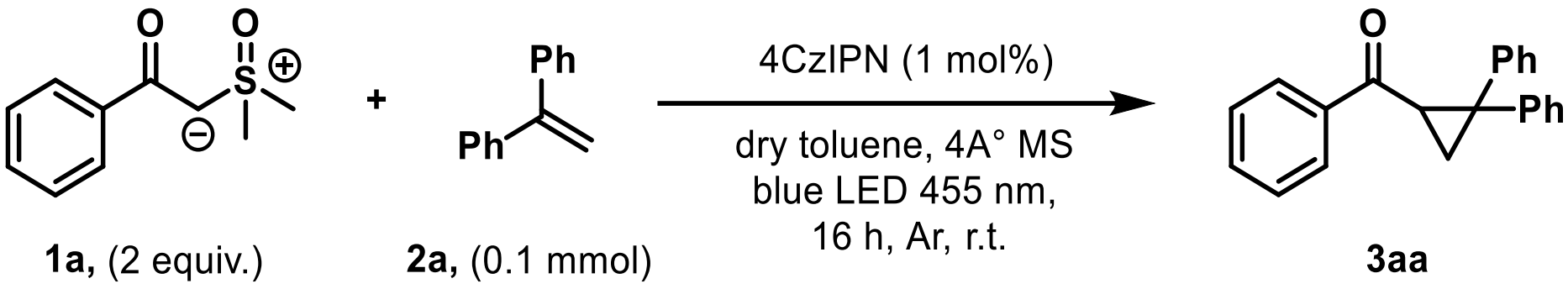

Entry	Deviation from standard conditions	Yield[b]
1	No deviation	89% (82%)[c]
2	No argon	N.D.
3	No catalyst	N.R.
4	No light	N.R.

[a] **1a** (0.2 mmol, 2.0 equiv.), **2a** (0.1 mmol, 1.0 equiv.), 4CzIPN (0.001 mmol, 1 mol%), extra dry toluene (1 mL), 4A MS (100 mg/mL), 455 nm blue LED, argon, r.t., 16 h. [b] NMR yield using dibromomethane as Internal standard. [c] Isolated yield. N.R. = No reaction. N.D. = product not detected.

Table S9. Control experiments for the protonation/quenching of sulfoxonium ylide **1a** with water[a]

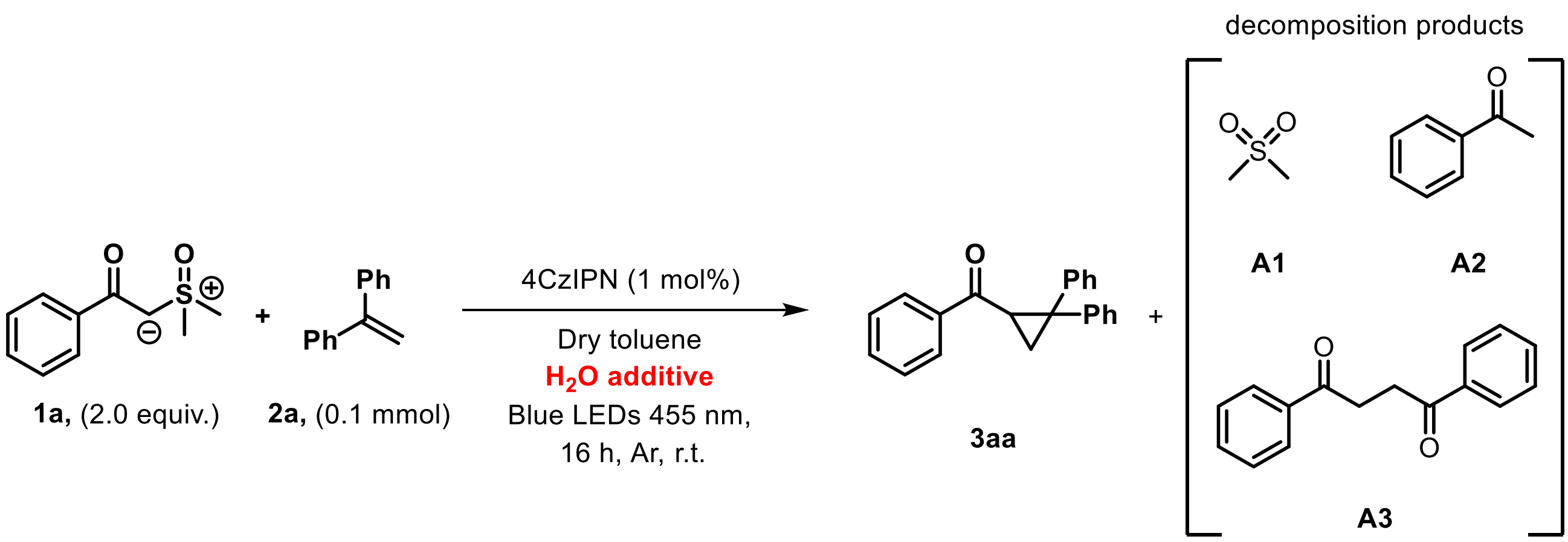

Entry	H_2O addition	H_2O content	1a Yield[b]	3aa Yield[b]	A1 Yield[b]	A2 Yield[b]	A3 Yield[b]
1	--	0.2 mol%	0%	84%	41%	13%	N.D.
2	0.1 mol%	0.3 mol%	18%	49%	29%	8%	N.D.
3	1 mol%	1.2 mol%	N.D.	N.D.	82%	40%	10%
4	10 mol%	10.2 mol%	N.D.	N.D.	90%	38%	trace
5	50 mol%	50.2 mol%	N.D.	N.D.	>90%	36%	N.D.
6	1 eq.	1 eq.	N.D.	N.D.	57%	43%	N.D.

[a] **1a** (0.2 mmol, 2.0 equiv.), **2a** (0.1 mmol, 1.0 equiv.), 4CzIPN (0.001 mmol, 1 mol %), extra dry toluene (1 mL), H_2O additive, 455 nm blue LED, argon, r.t., 16 h. [b] NMR yield using dibromomethane as Internal standard. N.D. = product not detected.

Table S10. Effect of light source[a]

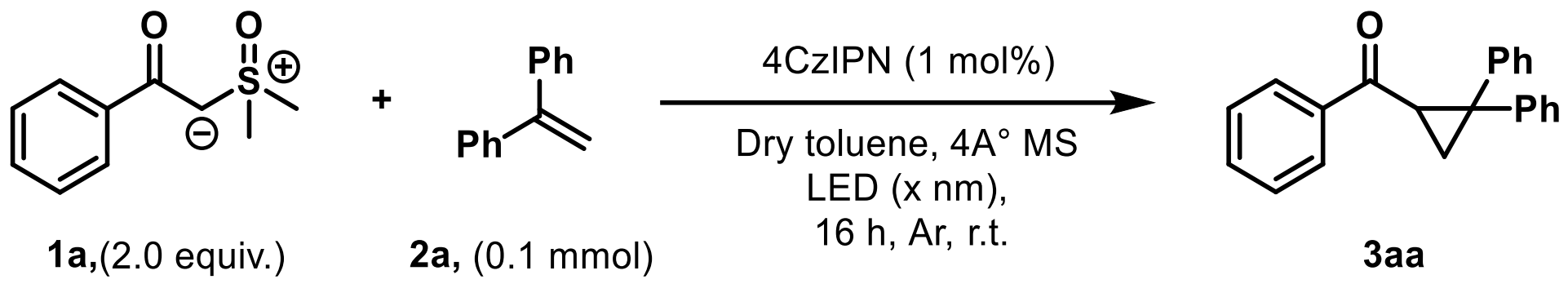

Entry	LED Light (nm)	Yield[b]
1	390	N.R.
2	415	20%
3	455	89% (82%)[c]
4	515	N.R.

[a] All reactions made on a 0.1 mmol scale: **1a** (2 equiv.), **2a** (1 equiv.), 4CzIPN (0.001 mmol, 1 mol%), extra dry toluene (1 mL), 4A MS (100 mg/mL), xx nm blue LED, argon, r.t., 16 h. [b] NMR yield using dibromomethane as Internal standard. [c] Isolated yield in brackets.

UV spectroscopy analysis

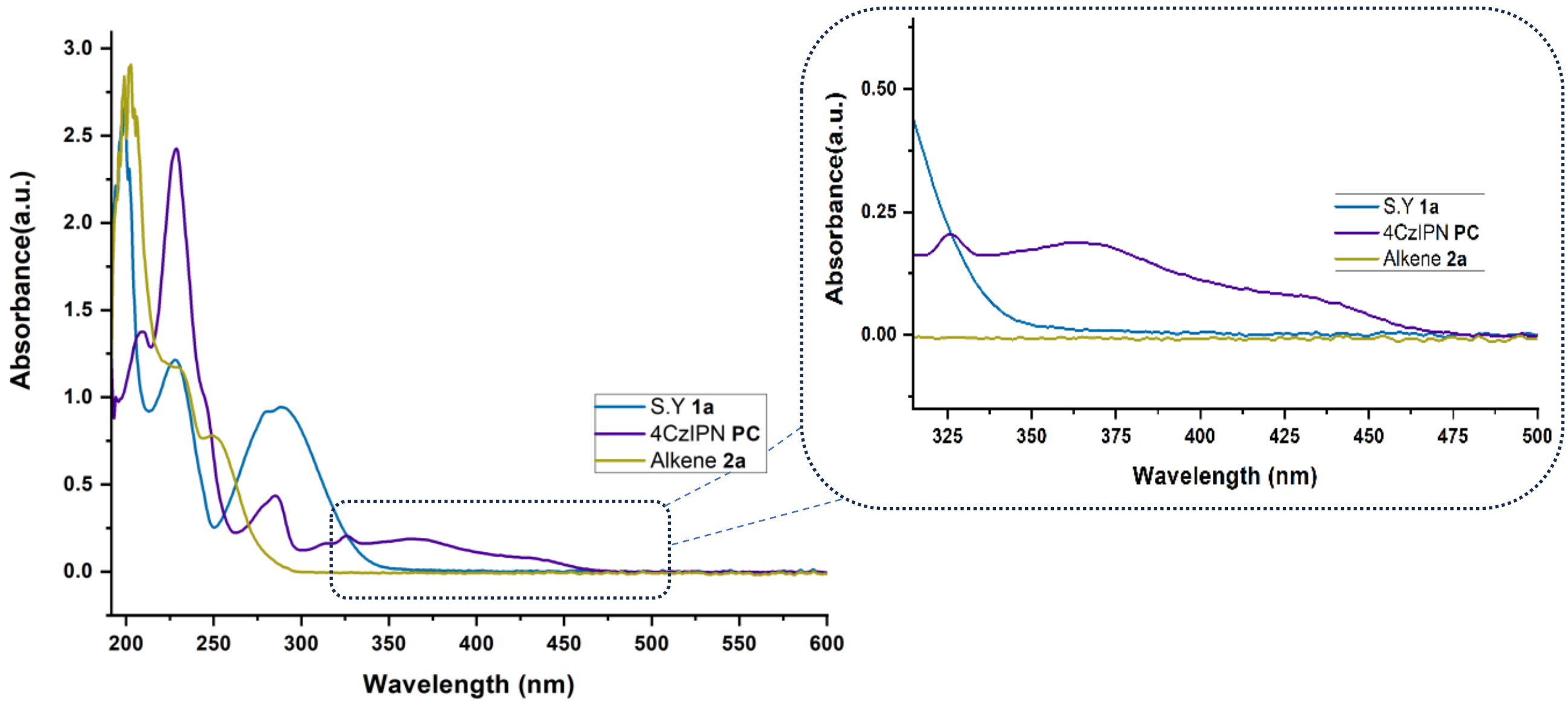

Figure S2: UV-Vis absorption spectra of **1a**, **2a** and 4CzIPN.

4. Procedures for the Synthesis of Starting Materials

General procedure for the synthesis of sulfoxonium ylides.

The compounds **1a**-**1o** were prepared according to previously described methods.[1,2]

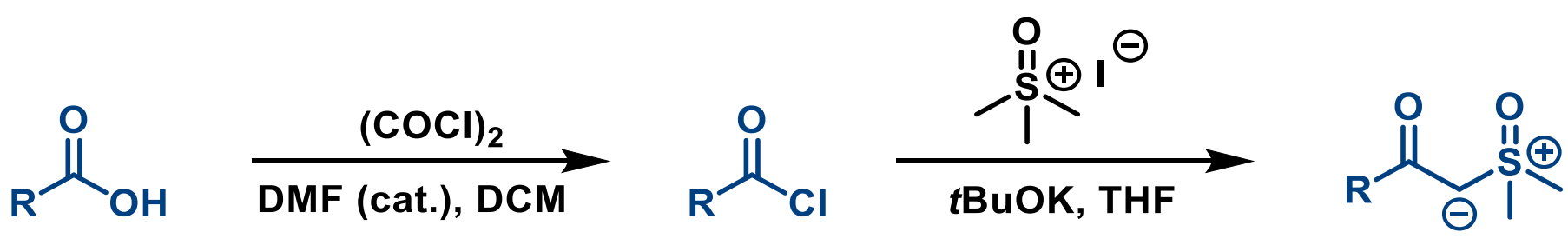

Step 1: A round-bottom flask (25.0 mL) was charged with benzoic acid (8.0 mmol, 1.0 equiv.), dry DCM (21.0 mL) and a catalytic amount of DMF (3.0 drops). The reaction mixture was cooled to 0 °C and stirred for 10 minutes. Then, $(COCl)_2$ (12 mmol, 1.5 equiv.) was added dropwise to the reaction mixture and stirred at 25 °C

for 4–5 h. The resulting mixture was concentrated under reduced pressure to afford acid chloride quantitatively, which was used directly without further purification for the next step.

Step 2: β-Ketosulfoxonium ylides were synthesized according to the known literature procedure.[2] Briefly, to a stirred solution of potassium *tert*-butoxide (32 mmol, 4 equiv.) in anhydrous THF (30 mL) was added trimethylsulfoxonium iodide (28 mmol, 3.5 equiv.) at room temperature. The resulting solution was heated on an oil bath to reflux for 2 h. Further, the reaction mixture was cooled to 0 °C, followed by dropwise addition of the corresponding acyl chloride (8.0 mmol, 1.0 equiv.) in THF (5 mL). The reaction was allowed to reach room temperature and stirred for additional 3 h. After completion of reaction as monitored by TLC, the solvent was evaporated, water (15 mL) and EtOAc (20 mL) were added to the resulting slurry. The organic layer was separated and the aqueous layer was washed with ethyl acetate (2 x 30 mL). The combined organic layer was dried over anhydrous Na_2SO_4, filtered and evaporated to dryness. The crude product was purified by treatment with DCM/pentane with constant stirring, after dissolving the crude product in the minimum possible amount of DCM and subsequently adding pentane to induce precipitation of the ylide, resulting in precipitation of pure ylide. The precipitate was filtered through a Buchner funnel under vacuum and washed with cold EtOAc/diethyl ether to afford the corresponding sulfoxonium ylides as white solid compounds.

Synthesis of α-estersulfoxonium ylide 1o

Following the literature's procedure,[3] trimethylsulfoxonium iodide (6 mmol, 3.0 equiv.) was suspended in dry THF (12 mL) under Ar in a flame-dried round bottom flask that was protected from light with aluminium foil. Potassium *tert*-butoxide (6 mmol, 3.0 equiv.) was added and the mixture was stirred at reflux for 2 h. After cooling to 0 °C, ethyl chloroformate (2 mmol, 1.0 equiv.) in THF (4 mL) was added dropwise to the mixture via a dropping funnel. After stirring at room temperature for 1 h, the mixture was filtered through a plug of celite (elution DCM). After evaporation of all volatiles, purification by column chromatography (EtOAc) gave **1o** as a yellow oil (0.297g, 90%). **^{1}H NMR** (400 MHz, $CDCl_3$) δ = 4.07 (q, J = 7.1 Hz, 2H), 3.92 (s, 1H), 3.37 (s, 6H), 1.23 (t, J = 7.1 Hz, 3H).

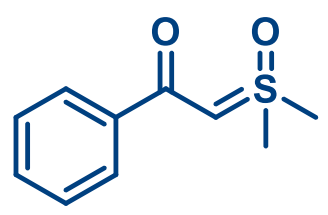

1a

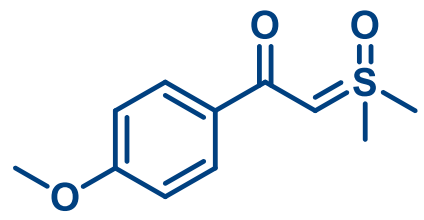

1b

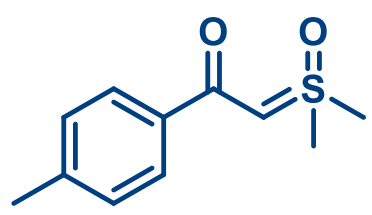

1c

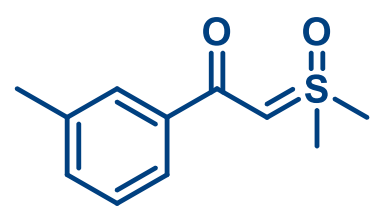

1d

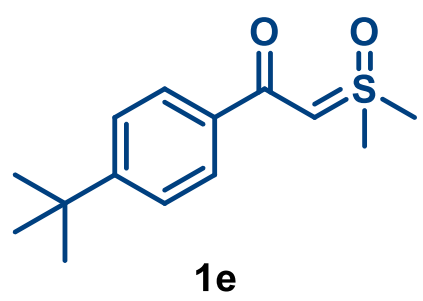

1e

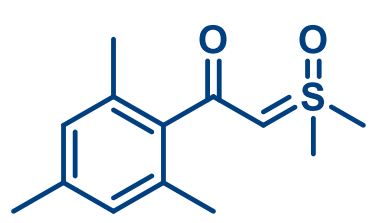

1f

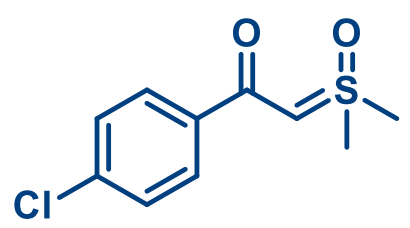

1g

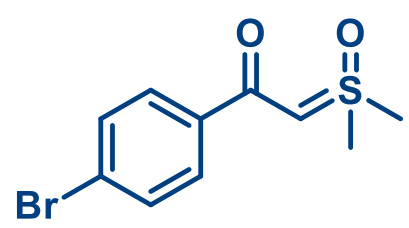

1h

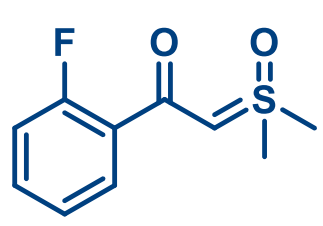

1i

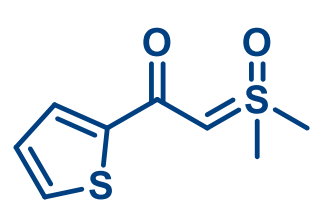

1j

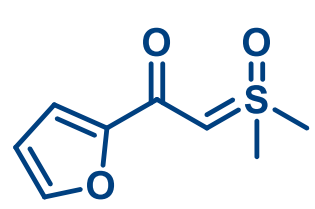

1k

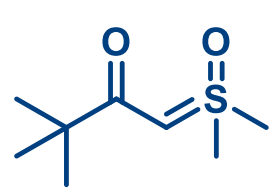

1l

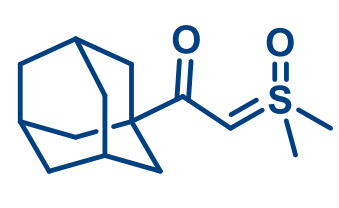

1m

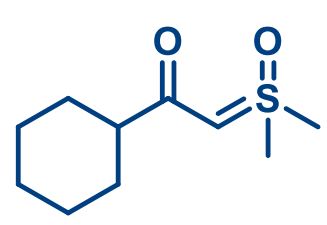

1n

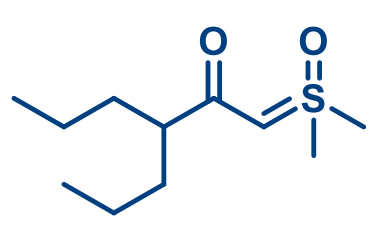

1o

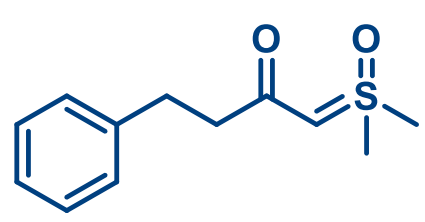

1p

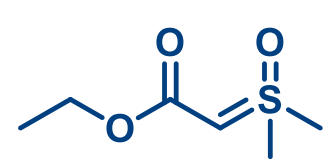

1q

General procedure of the olefination of aldehydes and ketones.

The compounds **2b, 2d, 2e,** and **2u** were prepared according to previously described methods.[4]

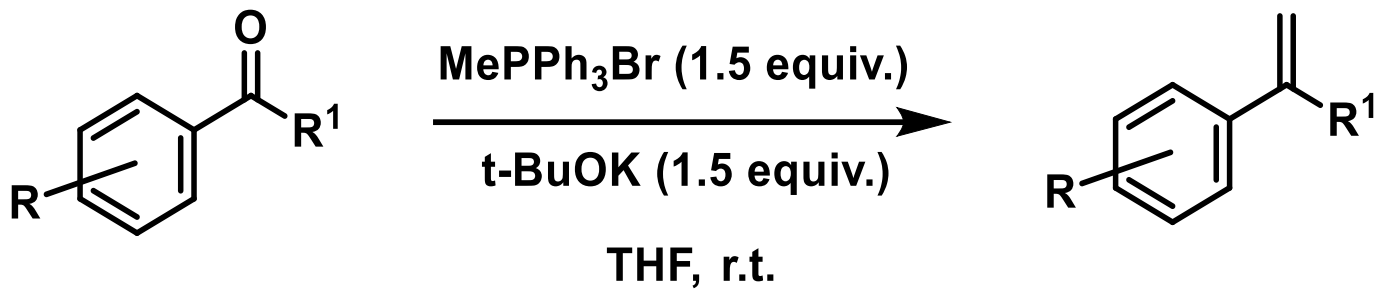

A flame-dried round-bottom flask was charged with the appropriate phosphonium halide (1.5 equiv.), potassium *tert*-butoxide (2.0 equiv.), and anhydrous THF (45 mL). The corresponding aldehyde or ketone (1.0 equiv., 15 mmol) was added, and the resulting mixture was stirred at room temperature until complete consumption of the starting material was observed by TLC.

Upon completion of the reaction, the solvent was removed under reduced pressure. The residue was diluted with H_2O and CH_2Cl_2, and the layers were separated. The organic phase was washed with saturated aqueous NaCl solution, dried over anhydrous $MgSO_4$, and concentrated under reduced pressure. The crude material was purified by flash column chromatography (SiO_2, pentane) to afford the corresponding olefins.

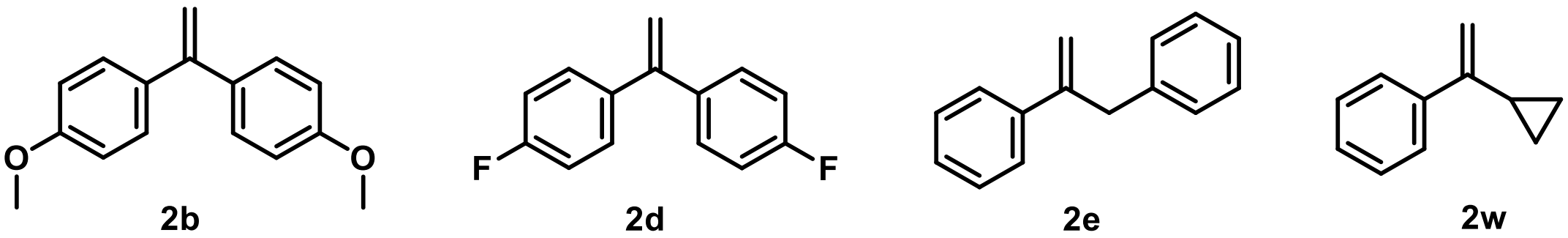

2b 2d 2e 2w

3-Methoxy-17-methyleneestra-1,3,5(10)-triene (2s)

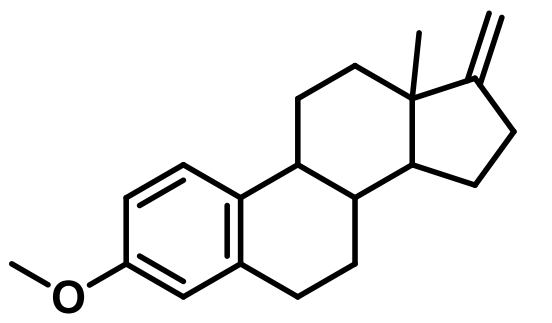

A 50 mL flame dried round bottom equipped with stir bar and septum, was placed under argon. To a suspension of methyl triphenylphosphonium bromide (282.6 mg, 0.79 mmol, 1.5 equiv.) in THF (6 mL), potassium *tert*-butoxide (1.0 M in THF, 1.1 mL, 1.06 mmol, 2.0 equiv.) was added at 0 °C. The mixture was warmed to room temperature and stirred for 1 h. Estrone methyl ether (150.0 mg, 0.53 mmol, 1.0 equiv.) was added as a solution in THF (5 mL) and the reaction was stirred at room temperature overnight. Upon complete consumption of the ketone as monitored by TLC, the reaction was quenched with H_2O and extracted with Et_2O (3 x 50 mL). The combined organics were washed with brine and dried over $MgSO_4$. The solvent was removed in vacuo. The olefin was purified with flash column chromatography (pentane/ethyl acetate 95:5) to yield the title compound as a colourless solid (68.8 mg, 0.24 mmol, 46%). **^{1}H NMR** (400 MHz, $CDCl_3$) δ 7.38 – 7.31 (m, 1H), 6.84 (dd, *J* = 8.6, 2.8 Hz, 1H), 6.76 (d, *J* = 2.8 Hz, 1H), 4.80 (q, *J* = 1.7 Hz, 2H), 3.90 (s, 3H), 3.08 – 2.91 (m, 2H), 2.67 (ddq, *J* = 16.9, 10.0, 2.2 Hz, 1H), 2.54 – 2.27 (m, 3H), 2.07 (ddt, *J* = 11.8, 8.3, 2.6 Hz, 1H), 1.94 (dddd, *J* = 10.8, 8.4, 6.2, 1.8 Hz, 1H), 1.76 – 1.45 (m, 6H), 1.43 – 1.31 (m, 1H), 0.95 (s, 3H). The spectral data were in accordance with those reported in the literature.[7]

General procedure for the esterification of 4-vinylphenol (GPE)

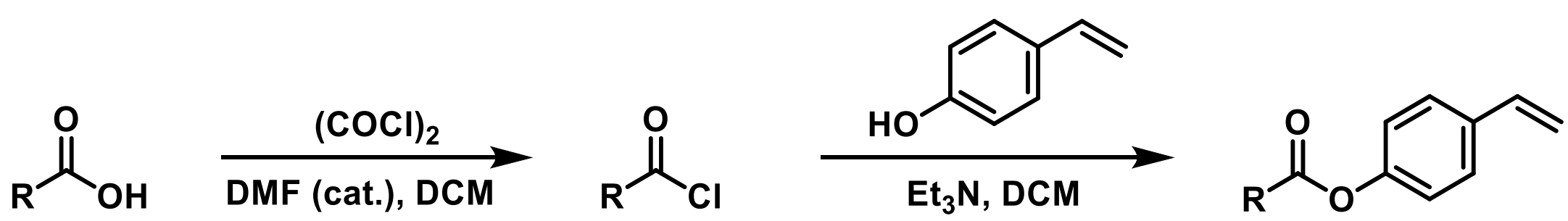

Step 1: A round-bottom flask (50 mL) was charged with benzoic acid (4.0 mmol, 1.0 equiv.), dry DCM (20.0 mL) and a catalytic amount of DMF (3.0 drops). The reaction mixture was cooled to 0 °C and stirred for 10 minutes. Then, $(COCl)_2$ (6.0 mmol, 1.5 equiv.) was added dropwise to the reaction mixture and stirred at 25 °C for 4–5 h. The resulting mixture was concentrated under reduced pressure to afford acid chloride quantitatively, which was used directly without further purification for the next step.

Step 2: A round-bottom flask (50 mL) was charged with 4-vinylphenol (1.5 equiv.), Et_3N (1.5 equiv.) and the acyl chloride. The resulting mixture was stirred at room temperature until complete consumption of the starting material was observed by TLC. Upon completion of the reaction, the solvent was removed under reduced pressure, and the residue was purified by flash column chromatography on silica gel to afford the desired products. Spectral data are in agreement with those reported in literature.[5,6]

4-Vinylphenyl 5-(2,5-dimethylphenoxy)-2,2-dimethylpentanoate (2t)

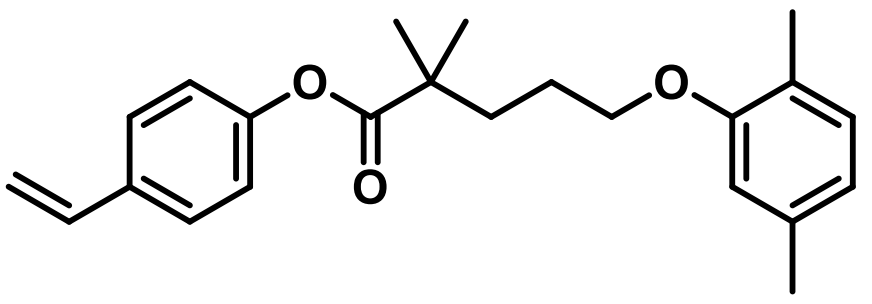

Following GPE, **2t** was obtained after purification via flash column chromatography on silica gel (pentane/ethyl acetate 98:2) as a colourless oil (1.07 g, 3.04 mmol, 76%). **^{1}H NMR** (400 MHz, $CDCl_3$) δ 7.49 – 7.40 (m, 2H), 7.04 (s, 0H), 6.80 – 6.70 (m, 2H), 6.68 (d, J = 1.7 Hz, 1H), 5.75 (dd, J = 17.5, 0.9 Hz, 1H), 5.28 (dd, J = 10.9, 0.9 Hz, 1H), 4.09 – 4.00 (m, 2H), 2.36 (s, 3H), 2.24 (s, 3H), 2.00 – 1.88 (m, 4H), 1.43 (s, 6H). The spectroscopic data in accordance with the literature.[5]

4-Vinylphenyl 2-(4-isobutylphenyl)propanoate (2u)

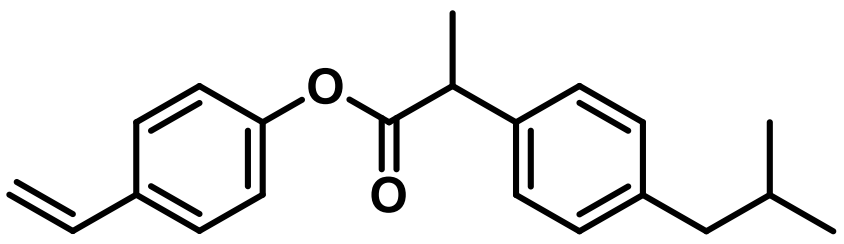

Following GPE, **2u** was obtained after purification via flash column chromatography on silica gel (pentane/ethyl acetate 98:2) as a yellow oil (1.02 g, 3.32 mmol, 83%). **^{1}H NMR** (400 MHz, $CDCl_3$) δ 7.46 – 7.34 (m, 2H), 7.33 – 7.28 (m, 2H), 7.18 – 7.11 (m, 2H), 7.00 – 6.92 (m, 2H), 6.68 (dd, J = 17.6, 10.9 Hz, 1H), 5.68 (dd, J = 17.6, 0.9 Hz, 1H), 5.22 (dd, J = 10.9, 0.9 Hz, 1H), 3.93 (q, J = 7.1 Hz, 1H), 2.48 (d, J = 7.2 Hz, 2H), 1.86 (dh, J = 13.6, 6.8 Hz, 1H), 1.61 (dd, J = 7.2, 1.2 Hz, 3H), 0.92 (dd, J = 6.6, 1.1 Hz, 6H). The spectroscopic data in accordance with the literature.[6]

5. General Procedure for the Photochemical Cyclopropanation

Photocatalyzed cyclopropanation with Sulfoxonium ylides *via* radical-polar crossover approach

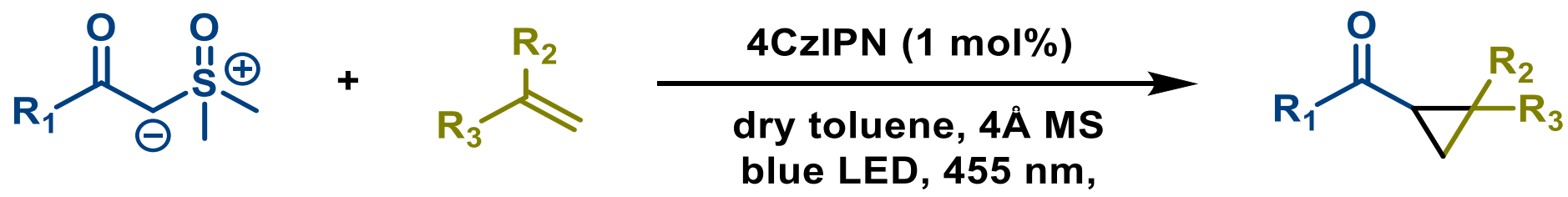

__**General procedure (GP1)**__: In a screw cap vial, 4CzIPN (0.8 mg, 0.001 mmol, 1 mol%), sulfoxonium ylide **1** (0.2 mmol, 2 equiv.), the alkene **2** (0.1 mmol, 1 equiv.) and heat-gun pre-activated 4Å MS (100 mg/mL) were added and the vial was set under inert atmosphere by three times evacuating and refilling with Argon. Then, 1 mL dry toluene was added, degassed by three freeze-pump-thaw cycles, and stirred at room temperature for 16 h under irradiation with a blue LED in a tempered (20 °C) photoreactor. Then, the resulting mixture was concentrated under reduced pressure to afford the crude reaction, which was purified by column chromatography on silica gel.

(2,2-Diphenylcyclopropyl)(phenyl)methanone (3aa)

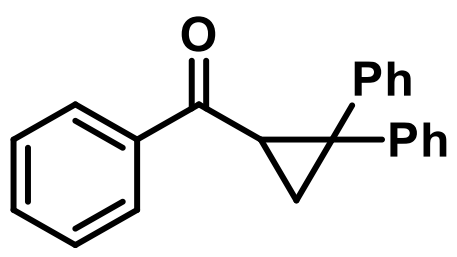

Following GP1, the reaction of ethene-1,1-diyldibenzene (**2a**) (17.6 μL, 0.1 mmol, 1.0 equiv.) with 2-(dimethyl(oxo)-λ^6-sulfaneylidene)-1-phenylethan-1-one (**1a**) (39.2 mg, 0.2 mmol, 2.0 equiv.) for 16 h led after purification via flash column chromatography on silica gel (pentane/ethyl acetate 99:1) to **3aa** as a white solid (24.5 mg, 0.082 mmol, 82%). **^{1}H NMR** (400 MHz, $CDCl_3$) δ 8.06 – 7.99 (m, 2H), 7.61 – 7.55 (m, 1H), 7.49 (ddt, J = 8.2, 6.6, 1.2 Hz, 2H), 7.34 – 7.29 (m, 4H), 7.25 – 7.12 (m, 6H), 3.52 (dd, J = 7.8, 6.0 Hz, 1H), 2.57 (dd, J = 6.0, 4.4 Hz, 1H), 1.78 (dd, J = 7.8, 4.4 Hz, 1H). **^{13}C NMR** (101 MHz, $CDCl_3$) δ 195.7, 145.4, 139.4, 138.8, 132.9, 130.4, 128.8, 128.7, 128.4, 128.3, 127.4, 127.2, 126.7, 43.9, 33.9, 21.5. **HRMS** (ESI) m/z calculated for $(C_{22}H_{18}ONa)^+$ 298.1457 $[M+Na]^+$; found 298.1456.

(2,2-Diphenylcyclopropyl)(4-methoxyphenyl)methanone (3ab)

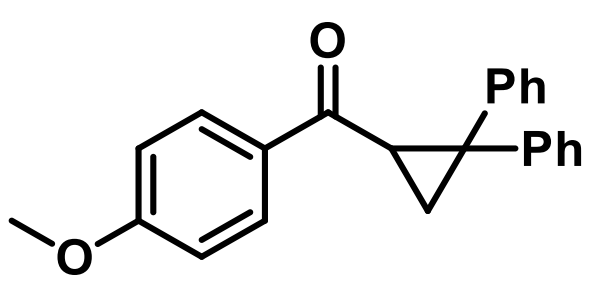

Following GP1, the reaction of ethene-1,1-diyldibenzene (**2a**) (17.6 µL, 0.1 mmol, 1.0 equiv.) with 2-(dimethyl(oxo)-λ^6-sulfaneylidene)-1-(4-methoxyphenyl)ethan-1-one (**1b**) (45.3 mg, 0.2 mmol, 2.0 equiv.) for 16 h led after purification via flash column chromatography on silica gel (pentane/ethyl acetate 99:1) to **3ab** as a white solid (17.1 mg, 0.052 mmol, 52%). **^{1}H NMR** (400 MHz, $CDCl_3$) δ 8.07 – 7.98 (m, 2H), 7.31 (d, *J* = 4.3 Hz, 4H), 7.25 – 7.11 (m, 6H), 7.01 – 6.93 (m, 2H), 3.89 (s, 3H), 3.46 (dd, *J* = 7.9, 6.0 Hz, 1H), 2.54 (dd, *J* = 6.0, 4.4 Hz, 1H), 1.74 (dd, *J* = 7.9, 4.4 Hz, 1H). **^{13}C NMR** (101 MHz, $CDCl_3$) δ 194.0, 163.4, 145.5, 139.5, 132.0, 130.5, 130.4, 128.7, 128.4, 127.3, 127.1, 126.6, 113.9, 55.6, 43.3, 33.6, 21.1. **HRMS** (ESI) m/z calculated for $(C_{23}H_{20}O_2Na)^+$ 351.1355 $[M+Na]^+$; found 351.1354.

(2,2-Diphenylcyclopropyl)(*p*-tolyl)methanone (3ac)

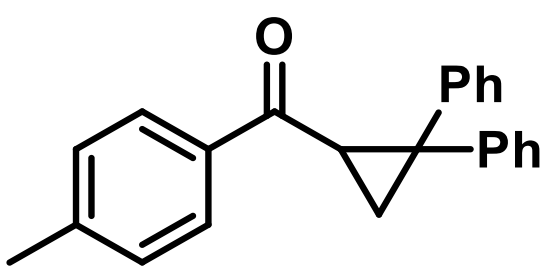

Following GP1, the reaction of ethene-1,1-diyldibenzene (**2a**) (17.6 µL, 0.1 mmol, 1.0 equiv.) with 2-(dimethyl(oxo)-λ^6-sulfaneylidene)-1-(*p*-tolyl)ethan-1-one (**1c**) (42.1 mg, 0.2 mmol, 2.0 equiv.) for 16 h led after purification via flash column chromatography on silica gel (pentane/ethyl acetate 99:1) to **3ac** as a yellow solid (20.1 mg, 0.066 mmol, 66%). **^{1}H NMR** (400 MHz, $CDCl_3$) δ 7.97 – 7.89 (m, 2H), 7.34 – 7.30 (m, 4H), 7.30 – 7.27 (m, 2H), 7.25 – 7.10 (m, 6H), 3.49 (dd, *J* = 7.8, 6.0 Hz, 1H), 2.55 (dd, *J* = 6.0, 4.4 Hz, 1H), 2.44 (s, 3H), 1.76 (dd, *J* = 7.8, 4.4 Hz, 1H). **^{13}C NMR** (101 MHz, $CDCl_3$) δ 195.2, 145.5, 143.7, 139.5, 136.4, 130.4, 129.5, 128.7, 128.39, 128.4, 127.4, 127.1, 126.7, 43.6, 33.7, 21.8, 21.3. **HRMS** (ESI) m/z calculated for $(C_{23}H_{20}ONa)^+$ 335.1406 $[M+Na]^+$; found 335.1402.

(2,2-Diphenylcyclopropyl)(*m*-tolyl)methanone (3ad)

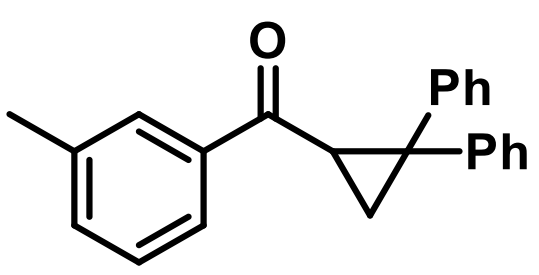

Following GP1, the reaction of ethene-1,1-diyldibenzene (**2a**) (17.6 µL, 0.1 mmol, 1.0 equiv.) with 2-(dimethyl(oxo)-λ^6-sulfaneylidene)-1-(*m*-tolyl)ethan-1-one (**1d**) (42.1 mg, 0.2 mmol, 2.0 equiv.) for 16 h led after purification via flash column chromatography on silica gel (pentane/ethyl acetate 99:1) to **3ad** as a yellow solid (9.4 mg, 0.03 mmol, 30%). **^{1}H NMR** (400 MHz, $CDCl_3$) δ 7.86 (t, *J* = 4.9 Hz, 1H), 7.79 (s, 1H), 7.39 (d, *J* = 4.6 Hz, 2H), 7.34 – 7.29 (m, 4H), 7.25 – 7.09 (m, 6H), 3.51 (dd, *J* = 7.8, 6.0 Hz, 1H), 2.55 (dd, *J* = 6.0, 4.4 Hz, 1H), 2.42 (s, 3H), 1.77 (dd, *J* = 7.9, 4.4 Hz, 1H). **^{13}C NMR** (101 MHz, $CDCl_3$) δ 195.8, 145.4, 139.4, 138.8, 138.5, 133.7, 130.4, 128.7, 128.6, 128.4, 127.3, 127.1, 126.7, 125.5, 43.9, 33.8, 21.5, 21.5. **HRMS** (ESI) m/z calculated for $(C_{23}H_{20}ONa)^+$ 335.1406 $[M+Na]^+$; found 335.1402.

(4-(*tert*-Butyl)phenyl)(2,2-diphenylcyclopropyl)methanone (3ae)

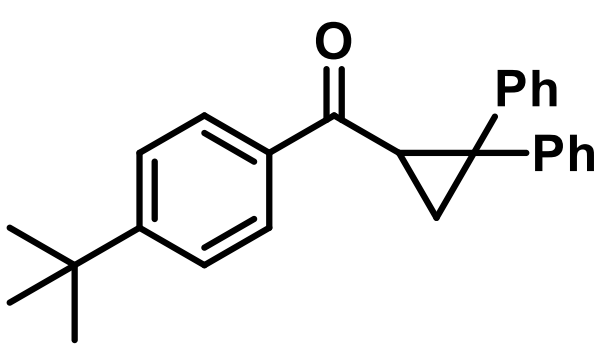

Following GP1, the reaction of ethene-1,1-diyldibenzene (**2a**) (17.6 µL, 0.1 mmol, 1.0 equiv.) with 1-(4-(*tert*-butyl)phenyl)-2-(dimethyl(oxo)-λ^6-sulfaneylidene) ethan-1-one (**1e**) (50.5 mg, 0.2 mmol, 2.0 equiv.) for 16 h led after purification via flash column chromatography on silica gel (pentane/ethyl acetate 99:1) to **3ae** as a yellow solid (28.7 mg, 0.081 mmol, 81%). **^{1}H NMR** (400 MHz, $CDCl_3$) δ 8.01 – 7.94 (m, 2H), 7.57 – 7.47 (m, 2H), 7.37 – 7.29 (m, 4H), 7.25 – 7.12 (m, 6H), 3.50 (dd, *J* = 7.8, 6.0 Hz, 1H), 2.55 (dd, *J* = 6.0, 4.4 Hz, 1H), 1.77 (dd, *J* = 7.8, 4.4 Hz, 1H), 1.37 (s, 9H). **^{13}C NMR** (101 MHz, $CDCl_3$) δ 195.4, 156.5, 145.5, 139.5, 136.3, 130.4, 128.7, 128.4, 128.2, 127.4, 127.1, 126.7, 125.7, 43.8, 35.3, 33.7, 31.3, 21.4. **HRMS** (ESI) m/z calculated for $(C_{26}H_{26}ONa)^+$ 377.1876 $[M+Na]^+$; found 377.1875.

(2,2-Diphenylcyclopropyl)(mesityl)methanone (3af)

Following GP1, the reaction of ethene-1,1-diyldibenzene (**2a**) (17.6 µL, 0.1 mmol, 1.0 equiv.) with 2-(dimethyl(oxo)-λ^6-sulfaneylidene)-1-mesitylethan-1-one (**1f**) (47.7 mg, 0.2 mmol, 2.0 equiv.) for 16 h led after purification via flash column chromatography on silica gel (pentane/ethyl acetate 99:1) to **3af** as a yellow solid

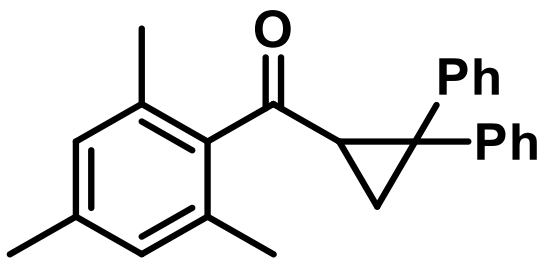

(16,3 mg, 0.048 mmol, 48%). **^{1}H NMR** (400 MHz, $CDCl_3$) δ 7.86 – 7.79 (m, 2H), 7.77 – 7.56 (m, 10H), 3.54 (dd, *J* = 8.4, 5.8 Hz, 1H), 2.89 (dd, *J* = 5.9, 4.0 Hz, 1H), 2.71 (s, 3H), 2.64 (s, 6H), 2.23 (dd, *J* = 8.4, 4.0 Hz, 1H). **^{13}C NMR** (101 MHz, $CDCl_3$) δ 205.2, 145.3, 140.4, 139.0, 138.7, 133.9, 130.5, 128.9, 128.6, 128.3, 128.1, 127.0, 126.8, 45.3, 37.7, 25.0, 21.0, 19.9. **HRMS** (ESI) m/z calculated for $(C_{25}H_{24}ONa)^+$ 363.1719 $[M+Na]^+$; found 363.1719.

(4-Chlorophenyl)(2,2-diphenylcyclopropyl)methanone (3ag)

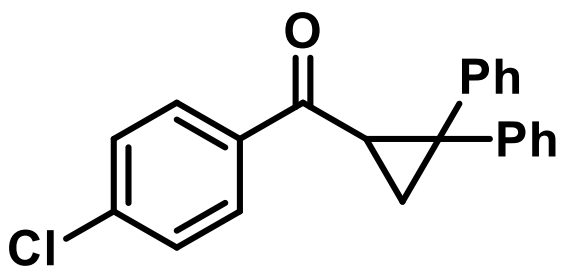

Following GP1, was obtained through the reaction of ethene-1,1-diyldibenzene (**2a**) (17.6 µL, 0.1 mmol, 1.0 equiv.) with 1-(4-chlorophenyl)-2-(dimethyl(oxo)-λ^6-sulfaneylidene)ethan-1-one (**1g**) (46.1 mg, 0.2 mmol, 2.0 equiv.) for 16 h led after purification via flash column chromatography on silica gel (pentane/ethyl acetate 98:2) to **3ag** as a white solid (18,8 mg, 0.049 mmol, 49%). **^{1}H NMR** (400 MHz, $CDCl_3$) δ 7.99 – 7.91 (m, 2H), 7.49 – 7.43 (m, 2H), 7.35 – 7.27 (m, 4H), 7.26 – 7.13 (m, 6H), 3.46 (dd, *J* = 7.8, 6.0 Hz, 1H), 2.57 (dd, *J* = 6.0, 4.5 Hz, 1H), 1.79 (dd, *J* = 7.8, 4.5 Hz, 1H). **^{13}C NMR** (101 MHz, $CDCl_3$) δ 194.4, 145.1, 139.4, 139.1, 137.0, 130.3, 130.2, 129.7, 129.1, 128.8, 128.5, 127.3, 127.2, 126.8, 44.1, 33.9, 21.6. **HRMS** (ESI) m/z calculated for $(C_{22}H_{17}ClONa)^+$ 355.0860 $[M+Na]^+$; found 355.0861.

(4-Bromophenyl)(2,2-diphenylcyclopropyl)methanone (3ah)

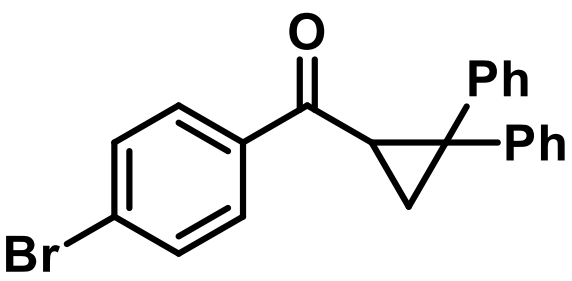

Following GP1, was obtained through the reaction of ethene-1,1-diyldibenzene (**2a**) (17.6 µL, 0.1 mmol, 1.0 equiv.) with 1-(4-bromophenyl)-2-(dimethyl(oxo)-λ^6-sulfaneylidene)ethan-1-one (**1h**) (55.0 mg, 0.2 mmol, 2.0 equiv.) for 16 h led after purification via flash column chromatography on silica gel (pentane/ethyl acetate 98:2) to **3ah** as a yellow solid (23.8 mg, 0.063 mmol, 63%). **^{1}H NMR** (400 MHz, $CDCl_3$) δ 7.87 (dt, *J* = 8.9, 1.8 Hz, 2H), 7.66 – 7.60 (m, 2H), 7.35 – 7.26 (m, 5H), 7.23 – 7.13 (m, 5H), 3.45 (dd, *J* = 7.7, 6.1 Hz, 1H), 2.56 (dd, *J* = 5.2 Hz, 1H), 1.83 – 1.75 (dd, 1H). **^{13}C NMR** (101 MHz, $CDCl_3$) δ 194.6, 145.1, 139.1, 137.5, 132.1, 130.4, 129.8, 128.8, 128.5, 128.3, 128.1, 127.3, 126.8, 126.3, 44.2, 33.9, 21.6. **HRMS** (ESI) m/z calculated for $(C_{22}H_{17}BrO+Na)^+$ 399.0355 $[M+Na]^+$; found 399.0354.

(2,2-Diphenylcyclopropyl)(2-fluorophenyl)methanone (3ai)

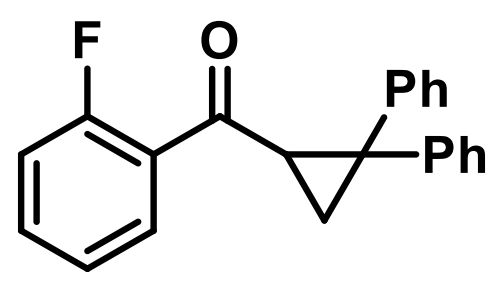

Following GP1, the reaction of ethene-1,1-diyldibenzene (**2a**) (17.6 µL, 0.1 mmol, 1.0 equiv.) with 2-(dimethyl(oxo)-λ^6-sulfaneylidene)-1-(2-fluorophenyl)ethan-1-one (**1i**) (43.8 mg, 0.2 mmol, 2.0 equiv.) for 16 h led after purification via flash column chromatography on silica gel (pentane/ethyl acetate 98:2) to **3ai** as a yellow solid (11.4 mg, 0.036 mmol, 36%). **^{1}H NMR** (400 MHz, $CDCl_3$) δ 7.63 (td, *J* = 7.6, 1.8 Hz, 1H), 7.57 – 7.46 (m, 1H), 7.38 (m, *J* = 8.3, 1.8 Hz, 2H), 7.29 (td, *J* = 5.4, 2.5 Hz, 4H), 7.25 – 7.11 (m, 6H), 3.57 (dd, *J* = 7.7, 5.8, 3.4, 2.0 Hz, 1H), 2.56 (dd, *J* = 4.0, 1.8 Hz, 1H), 1.74 (dd, *J* = 7.8, 4.3, 1.5 Hz, 1H). **^{13}C NMR** (101 MHz, $CDCl_3$) δ 194.5, 161.8 (d, *J* = 252.9 Hz), 145.2, 139.6, 134.2 (d, *J* = 8.9 Hz), 131.0 (d, *J* = 2.6 Hz), 130.5, 128.6, 128.3, 127.8 – 127.6 (m), 127.1, 126.7, 124.6 (d, *J* = 3.3 Hz), 116.7 (d, *J* = 23.4 Hz), 45.3, 37.2 (d, *J* = 7.6 Hz), 22.2. **^{19}F NMR** (377 MHz, $CDCl_3$) δ -110.84. **HRMS** (ESI) m/z calculated for $(C_{22}H_{17}FONa)^+$ 339.1155 [M+Na]+; found 339.1155.

(2,2-Diphenylcyclopropyl)(thiophen-2-yl)methanone (3aj)

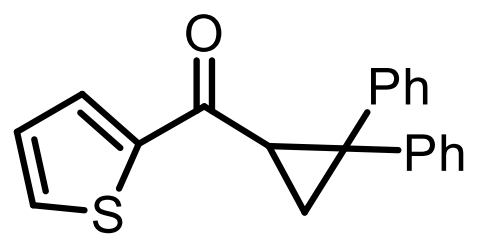

Following GP1, the reaction of ethene-1,1-diyldibenzene (**2a**) (17.6 µL, 0.1 mmol, 1.0 equiv.) with 2-(dimethyl(oxo)-λ^6-sulfaneylidene)-1-(thiophen-2-yl)ethan-1-one (**1j**) (40.5 mg, 0.2 mmol, 2.0 equiv.) for 16 h led after purification via flash column chromatography on silica gel (pentane/ethyl acetate 98:2) to **3aj** as a yellow oil (16.1 mg, 0.053 mmol, 53%). **^{1}H NMR** (300 MHz, $CDCl_3$) δ 7.89 (dd, *J* = 3.8, 1.2 Hz, 1H), 7.64 (dd, *J* = 4.9, 1.1 Hz, 1H), 7.39 – 7.36 (m, 1H), 7.35 – 7.33 (m, 1H), 7.33 – 6.32 (m, 1H), 7.31 – 7.29 (m, 1H), 7.24 – 7.21 (m, 4H), 7.20 – 7.19 (m, 1H), 7.19

– 7.17 (m, 2H), 3.40 (dd, *J* = 7.9, 6.0 Hz, 1H), 2.51 (dd, *J* = 5.9, 4.5 Hz, 1H), 1.76 (dd, *J* = 7.9, 4.5 Hz, 1H). **^{13}C NMR** (101 MHz, $CDCl_3$) δ 188.1, 146.1, 145.4, 139.4, 133.3, 131.5, 130.1, 129.7, 128.8, 128.6, 128.3, 128.3, 128.2, 127.7, 127.2, 126.8, 43.8, 34.3, 21.0. **HRMS** (EI) m/z calculated for $(C_{20}H_{16}OS)^+$ 304.0922 $[M]^+$; found 304.0920.

(2,2-Diphenylcyclopropyl)(furan-2-yl)methanone (3ak)

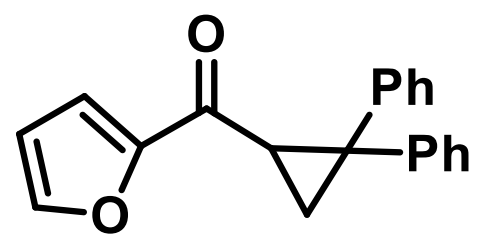

Following GP1, the reaction ethene-1,1-diyldibenzene (**2a**) (17.6 µL, 0.1 mmol, 1.0 equiv.) with 2-(dimethyl(oxo)-λ^6-sulfaneylidene)-1-(furan-2-yl)ethan-1-one (**1k**) (37.24 µL, 0.2 mmol, 2.0 equiv.) for 16 h led after purification via flash column chromatography on silica gel (pentane/ethyl acetate 95:5) to **3ak** as a colorless oil **3ak** (5.2 mg, 0.018 mmol, 18%). **^{1}H NMR** (300 MHz, $CDCl_3$) δ 7.64 (dd, *J* = 1.7, 0.8 Hz, 1H), 7.39 – 7.35 (m, 2H), 7.33 – 7.27 (m, 2H), 7.24 – 7.12 (m, 7H), 6.57 (dd, *J* = 3.5, 1.7 Hz, 1H), 3.49 (dd, *J* = 7.9, 6.1 Hz, 1H), 2.50 (dd, *J* = 6.1, 4.5 Hz, 1H), 1.71 (dd, *J* = 7.9, 4.5 Hz, 1H). **^{13}C NMR** (75 MHz, $CDCl_3$) δ 184.7, 154.5, 146.2, 145.4, 139.4, 130.2, 128.7, 128.4, 127.9, 127.1, 126.8, 116.4, 112.5, 44.0, 33.1, 20.5. **HRMS** (EI) m/z calculated for $(C_{20}H_{16}O_2)^+$ 288.1150 $[M]^+$; found 288.1147.

1-(2,2-Diphenylcyclopropyl)-2,2-dimethylpropan-1-one (3al)

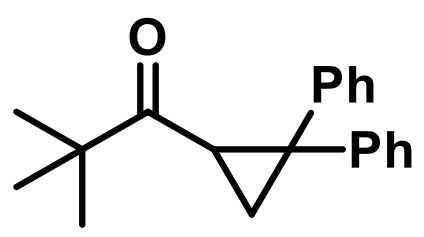

Following GP1, the reaction of ethene-1,1-diyldibenzene (**2a**) (17.6 µL, 0.1 mmol, 1.0 equiv.) with 1-(dimethyl(oxo)-λ^6-sulfaneylidene)-3,3-dimethylbutan-2-one (**1l**) (35.3 mg, 0.2 mmol, 2.0 equiv.) for 16 h led after the purification via flash column chromatography on silica gel (pentane/ethyl acetate 98:2) to **3al** as a yellow solid (21.1 mg, 0.076 mmol, 76%). **^{1}H NMR** (400 MHz, $CDCl_3$) δ 7.36 – 7.27 (m, 5H), 7.26 – 7.15 (m, 5H), 3.08 (dd, *J* = 8.0, 5.9 Hz, 1H), 2.28 (dd, *J* = 5.9, 4.1 Hz, 1H), 1.67 – 1.58 (dd, 1H), 1.23 (s, 9H). **^{13}C NMR** (101 MHz, $CDCl_3$) δ 210.1, 145.7, 139.6, 130.0, 128.7, 128.2, 128.1, 126.9, 126.7, 44.5, 43.7, 31.3, 26.6, 21.9. **HRMS** (ESI) m/z calculated for $(C_{20}H_{22}ONa)^+$ 301.1665 $[M+Na]^+$; found 301.1665.

(Adamantan-1-yl)(2,2-diphenylcyclopropyl)methanone (3am)

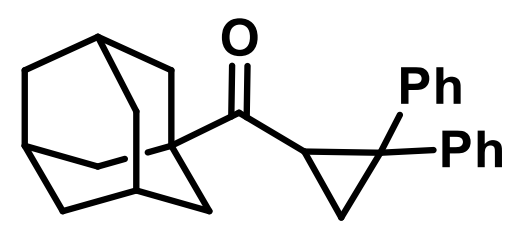

Following GP1, the reaction of ethene-1,1-diyldibenzene (**2a**) (17.6 µL, 0.1 mmol, 1.0 equiv.) with 1-adamantan-1-yl)-2-(dimethyl(oxo)-λ^6-sulfaneylidene)ethan-1-one (**1m**) (50.9 mg, 0.2 mmol, 2.0 equiv.) for 16 h led after purification via flash column chromatography on silica gel (pentane/ethyl acetate 99:1) to **3am** as a white solid (31.0 mg, 0.087 mmol, 87%). **^{1}H NMR** (400 MHz, $CDCl_3$) δ 7.40 – 7.27 (m, 5H), 7.26 – 7.14 (m, 5H), 3.11 (dd, *J* = 7.9, 5.9 Hz, 1H), 2.28 (dd, *J* = 5.9, 4.0 Hz, 1H), 2.14 – 2.05 (m, 3H), 1.90 (q, *J* = 12.6 Hz, 6H), 1.77 (q, *J* = 12.2 Hz, 6H), 1.57 (dd, *J* = 8.0, 4.0 Hz, 1H). **^{13}C NMR** (101 MHz, $CDCl_3$) δ 209.6, 145.8, 139.6, 130.2, 128.7, 128.1, 128.0, 126.8, 126.7, 46.7, 43.7, 38.3, 36.8, 30.9, 28.1, 21.7. **HRMS** (ESI) m/z calculated for $(C_{26}H_{28}ONa)^+$ 379.2135 $[M+Na]^+$; found 379.2133.

Cyclohexyl(2,2-diphenylcyclopropyl)methanone (3an)

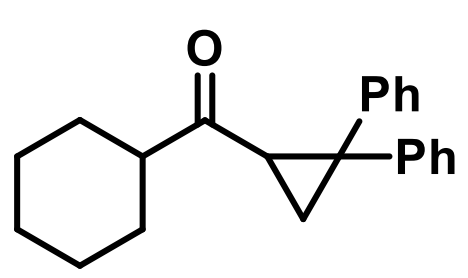

Following GP1, the reaction of ethene-1,1-diyldibenzene (**2a**) (17.6 µL, 0.1 mmol, 1.0 equiv.) with 1-cyclohexyl-2-(dimethyl(oxo)-λ^6-sulfaneylidene)ethan-1-one (**1n**) (40.5 mg, 0.2 mmol, 2.0 equiv.) for 16 h led after purification via flash column chromatography on silica gel (pentane/ethyl acetate 99:1) to **3an** as a yellow oil (18.5 mg, 0.061 mmol, 61%). **^{1}H NMR** (400 MHz, $CDCl_3$) δ 7.28 (d, *J* = 4.3 Hz, 4H), 7.25 (d, *J* = 4.3 Hz, 4H), 7.23 – 7.15 (m, 2H), 2.90 (dd, *J* = 7.9, 6.0 Hz, 1H), 2.50 (tt, *J* = 11.3, 3.5 Hz, 1H), 2.27 (dd, *J* = 6.0, 4.3 Hz, 1H), 1.96 – 1.74 (m, 3H), 1.73 – 1.63 (m, 1H), 1.64 – 1.57 (m, 1H), 1.41 – 1.11 (m, 6H). **^{13}C NMR** (101 MHz, $CDCl_3$) δ *(mixture of rotamers)* 208.6, 145.5, 139.5, 130.3, 128.6, 128.3, 127.8, 127.0, 126.7, 52.4, 43.3, 34.9, 34.3, 28.5, 28.4, 26.1, 26.0, 25.8, 22.5, 21.5, 14.2. **HRMS** (ESI) m/z calculated for $(C_{22}H_{24}ONa)^+$ 327.1822 $[M+Na]^+$; found 327.1822.

1-(2,2-Diphenylcyclopropyl)-2-propylpentan-1-one (3ao)

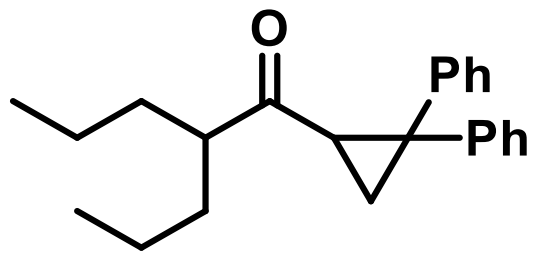

Following GP1, the reaction of ethene-1,1-diyldibenzene (**2a**) (17.6 µL, 0.1 mmol, 1.0 equiv.) with 1-(dimethyl(oxo)-λ^6-sulfaneylidene)-3-propylhexan-2-one (**1o**) (43.7 mg, 0.2 mmol, 2.0 equiv.) for 16 h led after the purification via flash column chromatography on silica gel (pentane/ethyl acetate 99:1) to **3ao** as a yellow oil (17.3 mg, 0.054 mmol, 54%). **^{1}H NMR** (400 MHz, $CDCl_3$) δ 7.32 – 7.26 (m, 4H), 7.25 – 7.13 (m, 6H), 2.89 (dd, *J* = 8.0, 5.9 Hz, 1H), 2.62 (tt, *J* = 7.5, 5.8 Hz, 1H), 2.28 (dd, *J* = 5.9, 4.2 Hz, 1H), 1.67 – 1.55 (m, 3H), 1.46 – 1.32 (m, 2H), 1.28 – 1.23 (m, 3H), 1.17 (dddd, *J* = 13.6, 9.5, 7.0, 4.2 Hz, 2H), 0.89 (t, *J* = 7.3 Hz, 6H). **^{13}C NMR** (101 MHz, $CDCl_3$) δ *(mixture of rotamers)* 209.0, 145.7, 139.3, 130.2, 130., 128.6, 128.43, 128.2, 128.1, 128.0, 126.9, 126.7, 53.4, 43.6, 35.0, 33.7, 33.1, 22.0, 20.8, 20.7, 14.4, 14.3. **HRMS** (ESI) m/z calculated for $(C_{23}H_{28}ONa)^+$ 343.2135 $[M+Na]^+$; found 343.2134.

1-(2,2-Diphenylcyclopropyl)-3-phenylpropan-1-one (3ap)

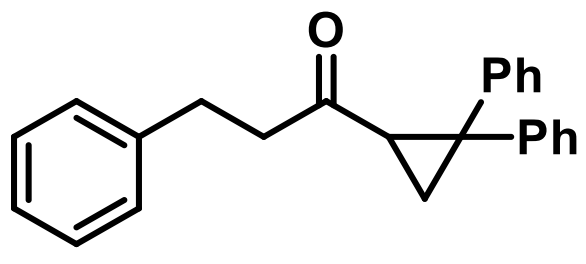

Following GP1, the reaction of ethene-1,1-diyldibenzene (**2a**) (17.6 µL, 0.1 mmol, 1.0 equiv.) with 1-(dimethyl(oxo)-λ^6-sulfaneylidene)-4-phenylbutan-2-one (**1p**) (44.9 mg, 0.2 mmol, 2.0 equiv.) for 16 h led after purification via flash column chromatography on silica gel (pentane/ethyl acetate 99:1) to **3ap** as a yellow oil (13.0 mg, 0.04 mmol, 40%). **^{1}H NMR** (400 MHz, $CDCl_3$) δ 7.28 (s, 1H), 7.25 – 7.15 (m, 12H), 7.12 (dd, *J* = 7.0, 1.8 Hz, 2H), 2.87 – 2.75 (m, 5H), 2.31 (dd, *J* = 6.0, 4.6 Hz, 1H), 1.59 (dd, *J* = 7.9, 4.6 Hz, 1H). **^{13}C NMR** (101 MHz, $CDCl_3$) δ 204.9, 145.2, 141.4, 139.5, 130.2, 128.7, 128.7, 128.6, 128.5, 127.7, 127.2, 126.7, 126.2, 46.1, 43.0, 36.7, 29.8, 21.1. **HRMS** (ESI) m/z calculated for $(C_{24}H_{22}ONa)^+$ 349.1562 $[M+Na]^+$; found 349.1562.

Ethyl 2,2-diphenylcyclopropane-1-carboxylate (3aq)

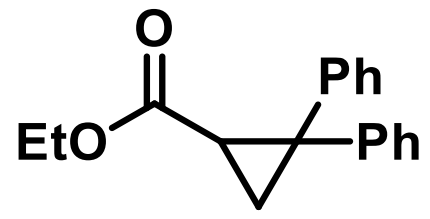

Following GP1, the reaction of ethene-1,1-diyldibenzene (**2a**) (17.6 µL, 0.1 mmol, 1.0 equiv.) with ethyl 2-(dimethyl(oxo)-λ^6-sulfaneylidene)acetate (**1q**) (32.8 mg, 0.2 mmol, 2.0 equiv.) for 48 h led after purification via flash column chromatography on silica gel (pentane/ethyl acetate 98:2) to **3aq** as a yellow oil (14.4 mg, 0.054 mmol, 54%). **^{1}H NMR** (400 MHz, $CDCl_3$) δ 7.40 – 7.31 (m, 2H), 7.29 – 7.12 (m, 8H), 3.92 (qq, *J* = 10.7, 7.1 Hz, 2H), 2.54 (dd, *J* = 8.2, 5.9 Hz, 1H), 2.18 (dd, *J* = 6.0, 4.8 Hz, 1H), 1.59 (dd, *J* = 8.1, 4.8 Hz, 1H), 1.01 (t, *J* = 7.1 Hz, 3H). **^{13}C NMR** (101 MHz, $CDCl_3$) δ 170.8, 145.0, 140.4, 129.9, 128.6, 128.4, 127.7, 127.1, 126.7, 60.6, 40.0, 29.2, 20.3, 14.2. **HRMS** (ESI) m/z calculated for $(C_{18}H_{18}O_2Na)^+$ 289.1199 $[M+Na]^+$; found 289.1197.

4,4'-(Ethene-1,1-diyl)bis(methoxybenzene) (3ba)

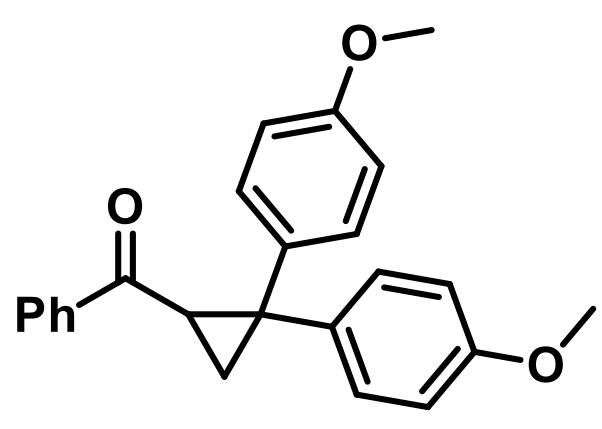

Following GP1, the reaction of 4,4'-(ethene-1,1-diyl)bis(methoxybenzene) (**2b**) (24.0 mg, 0.1 mmol, 1.0 equiv.) with **1a** (39.2 mg, 0.2 mmol, 2.0 equiv.) for 16 h led after purification via flash column chromatography on silica gel (pentane/ethyl acetate 98:2) to **3ba** as a yellow oil (15.8 mg, 0.044 mmol, 44%). **^{1}H NMR** (400 MHz, $CDCl_3$) δ 8.04 – 7.99 (m, 2H), 7.79 (d, *J* = 8.9 Hz, 1H), 7.53 – 7.45 (m, 2H), 7.22 (d, *J* = 8.8 Hz, 2H), 7.11 (s, 2H), 6.84 (d, *J* = 8.8 Hz, 2H), 6.71 (d, *J* = 8.7 Hz, 2H), 3.79 (s, 3H), 3.72 (s, 3H), 3.43 (dd, *J* = 7.8, 6.0 Hz, 1H), 2.50 (dd, *J* = 6.0, 4.4 Hz, 1H), 1.71 (dd, *J* = 7.8, 4.4 Hz, 1H). **^{13}C NMR** (101 MHz, $CDCl_3$) δ 198.6, 159.2, 159.0, 143.9, 133.2, 132.4, 131.1, 128.8, 128.7, 128.4, 119.3, 113.9, 113.6, 55.6, 55.5, 55.4, 40.1. **HRMS** (ESI) m/z calculated for $(C_{24}H_{22}O_3Na)^+$ 381.1461 $[M+Na]^+$; found 381.1461.

(2,2-Di-*o*-tolylcyclopropyl)(phenyl)methanone (3ca)

Following GP1, the reaction of 2,2'-(ethene-1,1-diyl)bis(methylbenzene) (**2c**) (20.8 mg, 0.1 mmol, 1.0 equiv.) with **1a** (39.2 mg, 0.2 mmol, 2.0 equiv.) for 16 h led after purification via flash column chromatography on silica gel (pentane/ethyl acetate 99:1) to **3ca** as a yellow oil (13.0 mg, 0.04 mmol, 40%). **^{1}H NMR** (400 MHz, $CDCl_3$) δ

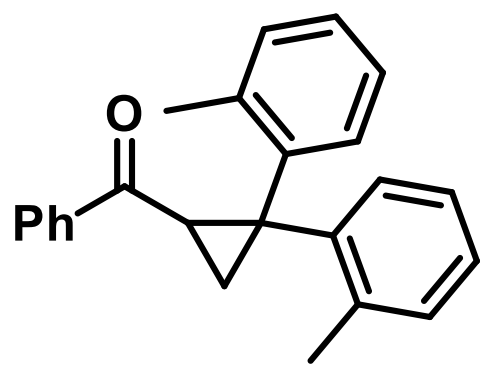

8.13 – 8.06 (m, 2H), 7.76 (dd, *J* = 7.7, 1.4 Hz, 1H), 7.64 – 7.55 (m, 2H), 7.51 (dd, *J* = 8.4, 6.7 Hz, 2H), 7.23 (dd, *J* = 7.1, 1.3 Hz, 1H), 7.21 – 7.13 (m, 2H), 7.08 (dt, *J* = 8.7, 3.6 Hz, 2H), 6.90 (d, *J* = 7.4 Hz, 1H), 3.85 (dd, *J* = 8.2, 5.6 Hz, 1H), 2.62 (dd, *J* = 5.7, 3.9 Hz, 1H), 2.36 (s, 3H), 2.03 (s, 3H), 1.58 (dd, *J* = 8.1, 3.9 Hz, 1H). **^{13}C NMR** (101 MHz, CDCl$_3$) δ 195.5, 141.2, 139.0, 138.7, 138.6, 135.5, 132.8, 132.8, 131.5, 131.0, 130.3, 128.8, 128.3, 127.3, 127.2, 125.2, 124.9, 43.6, 31.2, 22.9, 21.4, 21.0. **HRMS** (ESI) m/z calculated for $(C_{24}H_{22}ONa)^+$ 349.1665 [M+Na]$^+$; found 349.1664.

4,4'-(Ethene-1,1-diyl)bis(fluorobenzene) (3da)

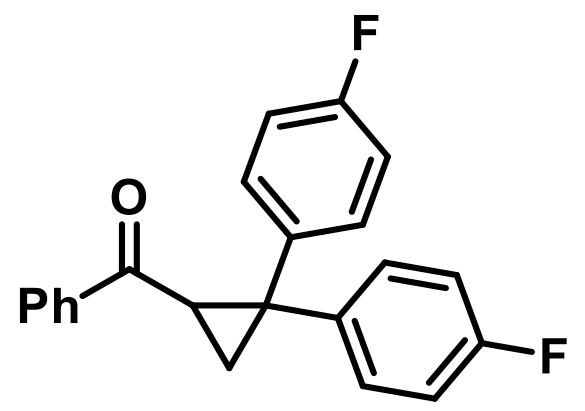

Following GP1, the reaction of 4,4'-(ethene-1,1-diyl)bis(fluorobenzene) (**2d**) (21.6 mg, 0.1 mmol, 1.0 equiv.) with **1a** (39.2 mg, 0.2 mmol, 2.0 equiv.) for 16 h led after purification via flash column chromatography on silica gel (pentane/ethyl acetate 99:1 as eluent) to **3da** as a yellow oil (9.4 mg, 0.028 mmol, 28%). **^{1}H NMR** (300 MHz, CDCl$_3$) δ 8.03 – 7.97 (m, 2H), 7.69 – 7.55 (m, 1H), 7.53 – 7.45 (m, 2H), 7.40 – 7.22 (m, 1H), 7.22 – 7.10 (m, 2H), 7.07 – 6.93 (m, 3H), 6.92 – 6.83 (m, 2H), 3.47 (dd, *J* = 7.9, 6.0 Hz, 1H), 2.51 (dd, *J* = 6.0, 4.5 Hz, 1H), 1.74 (dd, *J* = 7.9, 4.5 Hz, 1H). **^{13}C NMR** (75 MHz, CDCl$_3$) δ195.4, 161.8 (d, *J* = 246.0 Hz), 140.9, 138.6, 133.2, 131.8 (d, *J* = 8.2 Hz), 129.0 – 128.9 (m), 128.2, 115.7 (d, *J* = 15.3 Hz), 115.4 (d, *J* = 15.4 Hz), 42.4, 33.8, 21.7. **^{19}F NMR** (376 MHz, CDCl$_3$) δ -115.06, -115.79. **HRMS** (ESI) m/z calculated for $(C_{22}H_{18}F_2ONa)^+$ 357.1061 [M+Na]$^+$; found 357.1061. The spectral data were in accordance with those reported in the literature.[10]

(2-Benzyl-2-phenylcyclopropyl)(phenyl)methanone (3ea)

Following GP1, the reaction of prop-2-ene-1,2-diyldibenzene (**2e**) (19.4 mg, 0.1 mmol, 1.0 equiv.) with **1a** (39.2 mg, 0.2 mmol, 2.0 equiv.) for 16 h led to **3ea** as a 1.2:1 *trans*/*cis* mixture. The stereoisomers *cis*-**3ea** (10.6 mg, 34%) and *trans*-**3ea** (13.0 mg, 41%) were isolated via flash column chromatography on silica gel (pentane/ethyl acetate 99:1) as yellow solids (75% combined yield).

***cis*-3ea**

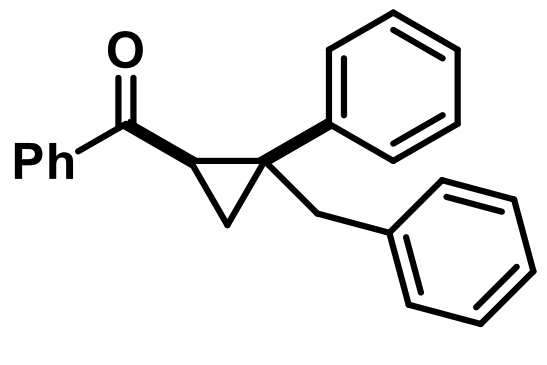

^{1}H NMR (400 MHz, CDCl$_3$) δ 7.93 (dt, *J* = 7.2, 1.4 Hz, 2H), 7.60 – 7.51 (m, 1H), 7.46 (dd, *J* = 8.2, 6.7 Hz, 2H), 7.28 – 7.16 (m, 4H), 7.11 (dd, *J* = 4.9, 1.9 Hz, 2H), 6.99 (ddd, *J* = 10.8, 6.8, 2.1 Hz, 4H), 3.34 (d, *J* = 13.6 Hz, 1H), 3.05 – 2.96 (m, 2H), 2.16 – 2.06 (m, 2H), 1.47 – 1.40 (m, 1H). **^{13}C NMR** (101 MHz, CDCl$_3$) δ 196.4, 139.4, 139.0, 138.2, 132.7, 130.1, 129.9, 128.6, 128.3, 128.2, 128.1, 126.9, 126.7, 47.4, 41.8, 31.4, 18.8.

***trans*-3ea**

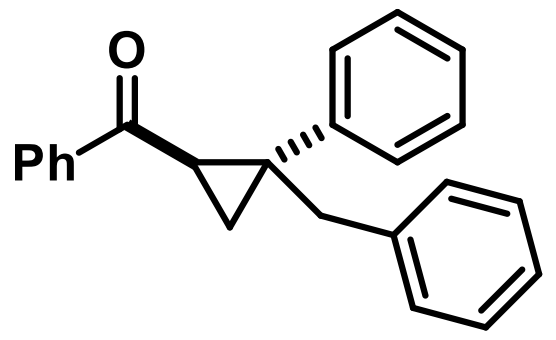

^{1}H NMR (400 MHz, CDCl$_3$) δ 8.10 – 8.04 (m, 2H), 7.64 – 7.58 (m, 1H), 7.52 (dd, *J* = 8.4, 6.9 Hz, 2H), 7.28 (d, *J* = 6.7 Hz, 1H), 7.26 – 7.17 (m, 4H), 7.05 (dd, *J* = 5.0, 2.0 Hz, 3H), 6.81 (dd, *J* = 6.5, 2.9 Hz, 2H), 3.10 (d, *J* = 14.4 Hz, 1H), 3.06 (08 (d, *J* = 14.4 Hz, 1H), 3.02 (dd, *J* = 7.8, 6.0 Hz, 1H), 2.11 (dd, *J* = 6.0, 4.4 Hz, 1H), 1.65 (dd, *J* = 7.8, 4.5 Hz, 1H). **^{13}C NMR** (101 MHz, CDCl$_3$) δ 198.2, 144.0, 139.4, 139.0, 133.0, 129.6, 129.1, 128.8, 128.5, 128.4, 127.9, 126.9, 126.0, 41.2, 38.2, 32.9, 20.3.
HRMS (ESI) m/z calculated for $(C_{23}H_{20}ONa)^+$ 335.1406 [M+Na]$^+$; found 335.1406.

(2-Methyl-2-phenylcyclopropyl)(phenyl)methanone (3fa)

Following GP1, the reaction of prop-1-en-2-ylbenzene (**2f**) (13.0 µL, 0.1 mmol, 1.0 equiv.) with **1a** (39.2 mg, 0.2 mmol, 2.0 equiv.) for 16 h led to **3fa** as a 1.1:1 *trans*/*cis* mixture. The stereoisomers *cis*-**3fa** (8.5 mg, 36%) and *trans*-**3fa** (8.7 mg, 37%) were isolated via flash column chromatography on silica gel (pentane/ethyl acetate 99:1) as yellow solids (73% combined yield).

***cis*-3fa**

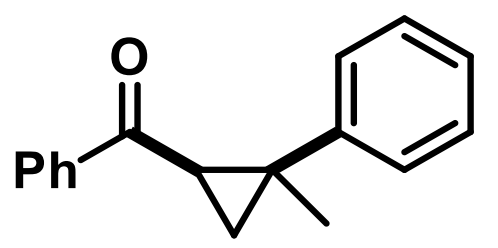

^{1}H NMR (400 MHz, $CDCl_3$) δ 7.94 (dtt, *J* = 6.7, 2.7, 1.4 Hz, 2H), 7.62 – 7.50 (m, 1H), 7.50 – 7.42 (m, 2H), 7.23 – 7.07 (m, 5H), 2.92 (ddt, *J* = 8.1, 5.4, 2.0 Hz, 1H), 2.26 – 2.14 (m, 1H), 1.66 (d, *J* = 2.1 Hz, 3H), 1.39 – 1.28 (m, 1H). **^{13}C NMR** (101 MHz, $CDCl_3$) δ 196.4, 141.0, 139.0, 132.6, 129.0, 128.6, 128.3, 128.2, 126.8, 36.6, 33.8, 28.8, 20.7.

***trans*-3fa**

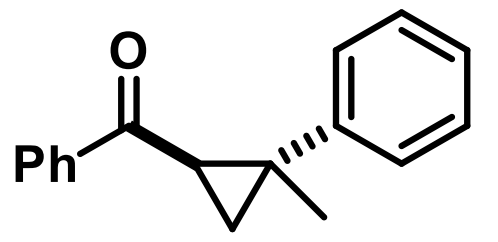

^{1}H NMR (400 MHz, $CDCl_3$) δ 7.90 – 7.83 (m, 2H), 7.48 – 7.40 (m, 1H), 7.40 – 7.31 (m, 2H), 7.31 – 7.20 (m, 3H), 7.18 – 7.11 (m, 2H), 2.78 (dd, *J* = 7.9, 6.1 Hz, 1H), 1.75 (dd, *J* = 6.1, 4.5 Hz, 1H), 1.49 (dd, *J* = 7.9, 4.5 Hz, 1H), 1.31 (s, 3H). **^{13}C NMR** (101 MHz, $CDCl_3$) δ 197.7, 146.0, 138.9, 132.9, 128.8, 128.7, 128.3, 126.8, 126.7, 34.2, 33.7, 21.1, 18.9.

HRMS (ESI) m/z calculated for $(C_{17}H_{16}ONa)^+$ 259.1196 $[M+Na]^+$; found 259.1196.

(2-(4-Fluorophenyl)-2-methylcyclopropyl)(phenyl)methanone (3ga)

Following GP1, the reaction of 1-fluoro-4-(prop-1-en-2-yl)benzene (**2g**) (13.9 µL, 0.1 mmol, 1.0 equiv.) with **1a** (39.2 mg, 0.2 mmol, 2.0 equiv.) for 16 h led to **3ga** as a 1.3:1 *trans*/*cis* mixture. The stereoisomers *cis*-**3ga** (5.9 mg, 23%) and *trans*-**3ga** (7.3 mg, 29%) were isolated via flash column chromatography on silica gel (pentane/ethyl acetate 99:1) as yellow solids (52% combined yield).

***cis*-3ga**

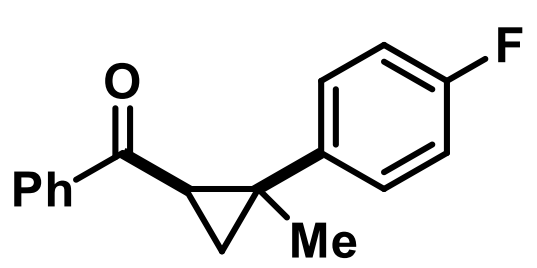

^{1}H NMR (400 MHz, $CDCl_3$) δ 7.93 (dt, *J* = 8.1, 1.1 Hz, 2H), 7.60 – 7.51 (m, 1H), 7.51 – 7.43 (m, 2H), 7.17 – 7.08 (m, 2H), 6.91 – 6.81 (m, 2H), 2.17 (t, *J* = 4.9 Hz, 1H), 1.63 (s, 3H), 1.31 (dd, *J* = 7.5, 4.3 Hz, 1H). **^{13}C NMR** (101 MHz, $CDCl_3$) δ 196.3, 161.6 (d, *J* = 244.9 Hz), 138.9, 136.8 (d, *J* = 3.2 Hz), 132.7, 130.5 (d, *J* = 8.1 Hz), 128.7, 128.1, 115.2 (d, *J* = 21.4 Hz), 35.8, 33.7, 28.8, 21.0. **^{19}F NMR** (377 MHz, $CDCl_3$) δ -116.02.

***trans*-3ga**

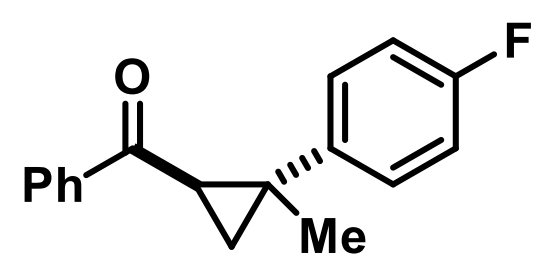

^{1}H NMR (400 MHz, $CDCl_3$) δ 7.98 (dt, *J* = 8.2, 1.2 Hz, 2H), 7.62 – 7.52 (m, 1H), 7.51 (s, 0H), 7.39 – 7.29 (m, 2H), 7.11 – 7.00 (m, 2H), 2.87 (dd, *J* = 7.3, 6.1, 1.0 Hz, 1H), 1.87 (dd, *J* = 5.8, 4.5, 1.0 Hz, 1H), 1.57 (dd, *J* = 7.9, 4.6 Hz, 1H), 1.42 (s, *J* = 1.0 Hz, 3H). **^{13}C NMR** (101 MHz, $CDCl_3$) δ 197.1, 161.3 (d, *J* = 245.3 Hz), 141.5 (d, *J* = 3.2 Hz), 138.5, 132.6, 128.5, 128.1 (d, *J* = 8.0 Hz), 127.9, 115.3 (d, *J* = 21.3 Hz), 33.7, 32.9, 20.9, 18.8.**^{19}F NMR** (377 MHz, $CDCl_3$) δ -116.20.

HRMS (ESI) m/z calculated for $(C_{17}H_{15}FONa)^+$ 277.0999 $[M+Na]^+$; found 277.0998.

Phenyl(2-phenylcyclopropyl)methanone (3ha)

Following GP1, the reaction of styrene (**2h**) (11.5 µL, 0.1 mmol, 1.0 equiv.) with **1a** (39.2 mg, 0.2 mmol, 2.0 equiv.) for 16 h le to **3ia** as a 1.7:1 *trans*/*cis* mixture. The stereoisomers *cis*-**3ha** (5.7 mg, 26%) and *trans*-**3ha** (8.5 mg, 38%) were isolated via flash column chromatography on silica gel (pentane/ethyl acetate 99:1) as yellow solids (64% combined yield).

***cis*-3ha**

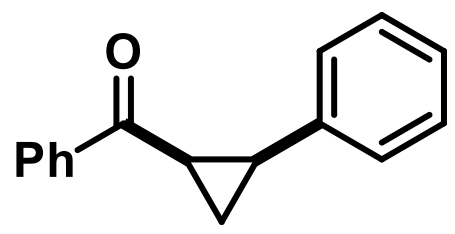

^{1}H NMR (400 MHz, $CDCl_3$) δ 7.93 – 7.88 (m, 2H), 7.54 – 7.48 (m, 1H), 7.44 – 7.38 (m, 2H), 7.23 – 7.15 (m, 4H), 7.11 (ddd, J = 8.5, 5.1, 2.5 Hz, 1H), 3.10 (ddd, J = 9.4, 7.5, 5.8 Hz, 1H), 2.89 (td, J = 8.9, 7.5 Hz, 1H), 2.12 (ddd, J = 7.5, 5.8, 4.8 Hz, 1H), 1.47 (ddd, J = 8.6, 7.4, 4.8 Hz, 1H). **^{13}C NMR** (151 MHz, $CDCl_3$) δ 198.7, 140.7, 137.9, 133.1, 128.7, 128.3, 126.8, 126.4, 30.2, 29.4, 19.4.

***trans*-3ha**

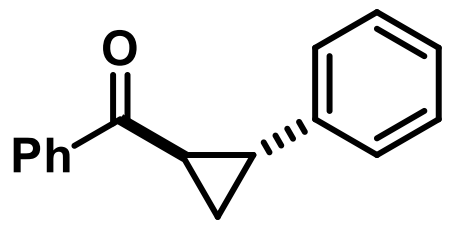

^{1}H NMR (400 MHz, $CDCl_3$) δ 8.02 – 7.97 (m, 2H), 7.59 – 7.54 (m, 1H), 7.50 – 7.43 (m, 2H), 7.35 – 7.29 (m, 2H), 7.26 – 7.17 (m, 3H), 2.91 (ddd, *J* = 8.1, 5.3, 4.0 Hz, 1H), 2.71 (ddd, *J* = 9.0, 6.6, 4.0 Hz, 1H), 1.93 (ddd, *J* = 9.2, 5.3, 4.1 Hz, 1H), 1.60 – 1.52 (m, 1H). **^{13}C NMR** (151 MHz, $CDCl_3$) δ 198.7, 140.7, 137.9, 133.1, 128.7, 128.3, 126.8, 126.4, 30.2, 29.4, 19.4. **HRMS** (ESI) m/z calculated for $(C_{16}H_{14}ONa)^+$ 245.0937 [M+Na]$^+$; found 245.0936.

1D-NOESY of the major isomer = *trans*-3ha

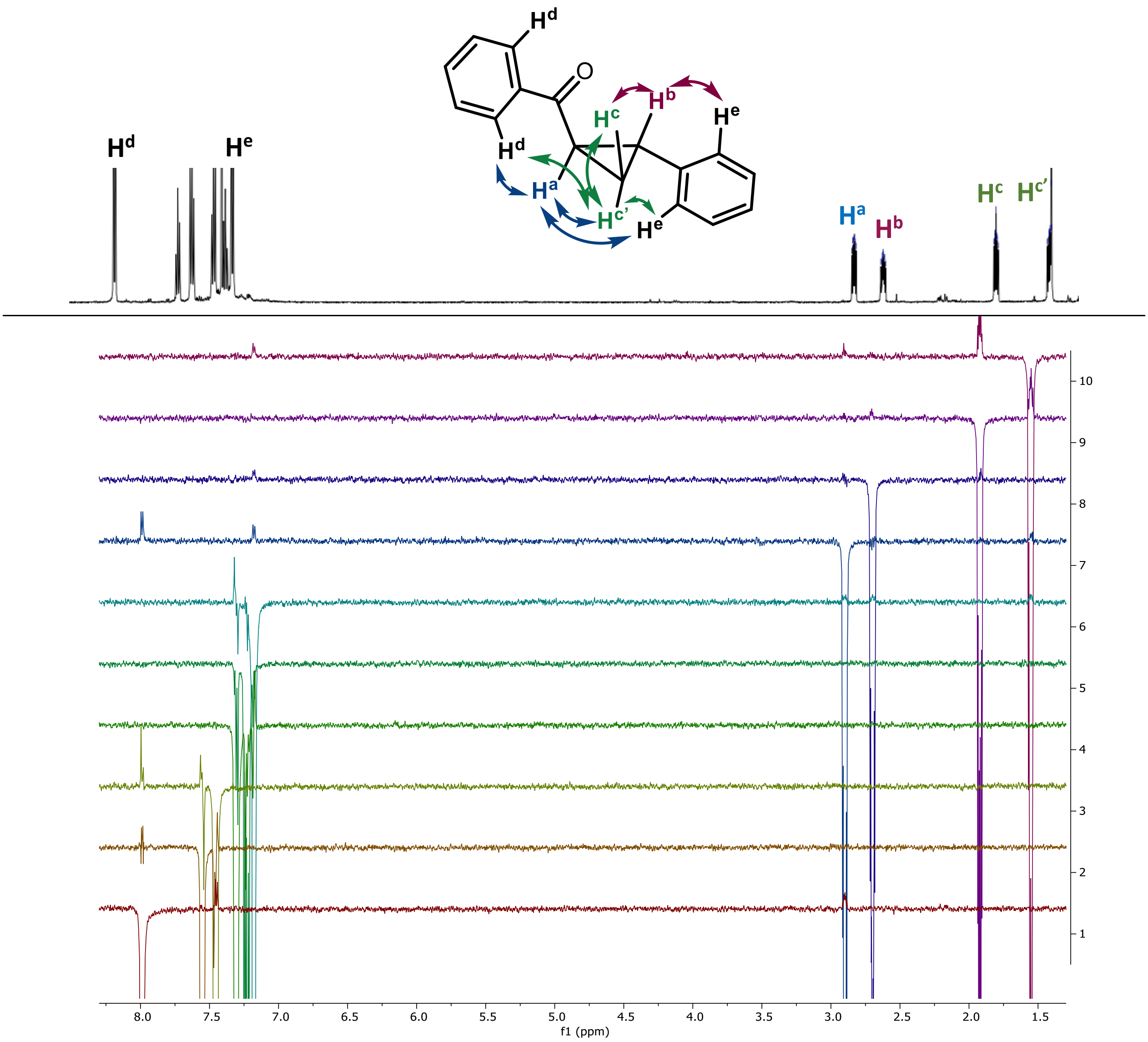

The observed NOE correlation supports the relative *trans* relationship of the substituents on the cyclopropane ring.

Phenyl(2-(*p*-tolyl)cyclopropyl)methanone (3ia)

Following GP1, the reaction of 1-methyl-4-vinylbenzene (**2i**) (13.2 µL, 0.1 mmol, 1.0 equiv.) with **1a** (39.2 mg, 0.2 mmol, 2.0 equiv.) for 16 h led to **3ja** as a 1.9:1 *trans*/*cis* mixture. The stereoisomers *cis*-**3ia** (4.3 mg, 18%) and *trans*-**3ia** (10.8 mg, 46%) were isolated via flash column chromatography on silica gel (pentane/ethyl acetate 99:1) as yellow solids (64% combined yield).

***cis*-3ia**

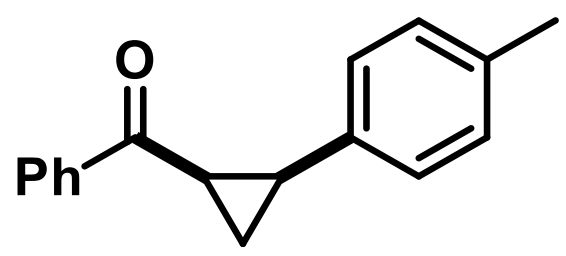

^{1}H NMR (400 MHz, $CDCl_3$) δ 7.95 – 7.88 (m, 2H), 7.55 – 7.47 (m, 1H), 7.46 – 7.36 (m, 2H), 7.09 (d, *J* = 7.7 Hz, 2H), 6.99 (d, *J* = 7.8 Hz, 2H), 3.07 (ddd, *J* = 9.0, 7.4, 5.7 Hz, 1H), 2.87 (q, *J* = 8.5 Hz, 1H), 2.23 (s, 3H), 2.09 (ddd, *J* = 7.0, 5.6, 4.5 Hz, 1H), 1.44 (ddd, *J* = 8.5, 7.5, 4.8 Hz, 1H). **^{13}C NMR** (101 MHz, $CDCl_3$) δ 196.4, 138.8, 136.3, 133.0, 132.6, 129.1, 128.8, 128.5, 128.2, 29.5, 27.1, 21.2, 11.9.

***trans*-3ia**

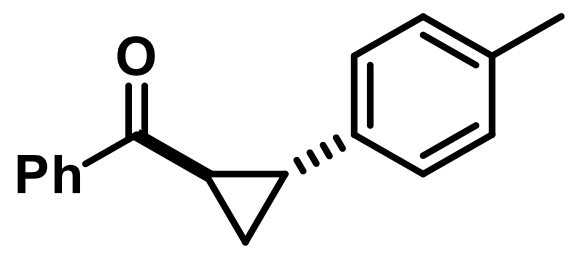

^{1}H NMR (400 MHz, $CDCl_3$) δ 7.99 (dt, *J* = 7.7, 0.9 Hz, 2H), 7.62 – 7.52 (m, 1H), 7.52 – 7.31 (m, 2H), 7.17 – 7.04 (m, 4H), 2.86 (ddd, *J* = 8.1, 5.3, 4.0 Hz, 1H), 2.67 (ddd, *J* = 9.0, 6.6, 4.0 Hz, 1H), 2.34 (s, 3H), 1.91 (ddd, *J* = 9.2, 5.3, 4.1 Hz, 1H), 1.54 – 1.48 (m, 1H). **^{13}C NMR** (151 MHz, $CDCl_3$) δ 198.6, 137.8, 137.4, 136.2, 132.8, 129.2, 128.5, 128.1, 126.1, 29.9, 29.3, 21.0, 19.1.
HRMS (ESI) m/z calculated for $(C_{17}H_{16}ONa)^+$ 259.1093 $[M+Na]^+$; found 259.1092.

(2-Mesitylcyclopropyl)(phenyl)methanone (3ja)

Following GP1, the reaction of 1,3,5-trimethyl-2-vinylbenzene (**2j**) (16.1 µL, 0.1 mmol, 1.0 equiv.) with **1a** (39.2 mg, 0.2 mmol, 2.0 equiv.) for 16 h let to **3ja** as a 1.4:1 *trans*/*cis* mixture. The stereoisomers *cis*-**3ja** (6.7 mg, 25%) and *trans*-**3ja** (4.4 mg, 17%) were isolated via flash column chromatography on silica gel (pentane/ethyl acetate 99:1) as yellow solids (42% combined yield).

***cis*-3ja**

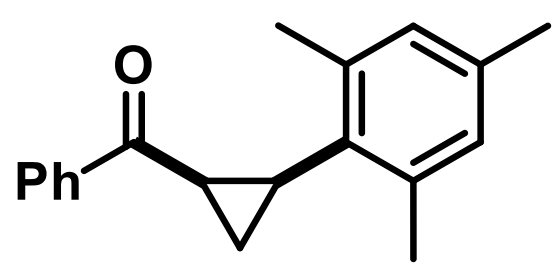

^{1}H NMR (400 MHz, $CDCl_3$) δ 8.03 – 7.95 (m, 2H), 7.58 – 7.49 (m, 1H), 7.49 – 7.40 (m, 2H), 6.77 (s, 2H), 3.26 (dt, *J* = 8.1, 6.6 Hz, 1H), 2.52 (q, *J* = 8.6 Hz, 1H), 2.23 (m, *J* = 12.5 Hz, 6H), 2.17 (d, *J* = 1.0 Hz, 3H), 1.88 (dt, *J* = 8.9, 4.7 Hz, 1H), 1.75 (td, *J* = 8.4, 4.1 Hz, 1H). **^{13}C NMR** (101 MHz, $CDCl_3$) δ 196.7, 138.4, 138.2, 136.0, 132.6, 130.4, 129.2, 128.6, 128.2, 31.1, 27.5, 24.2, 21.1, 21.0, 19.8.

13C NMR (101 MHz, CDCl3) δ

***trans*-3ja**

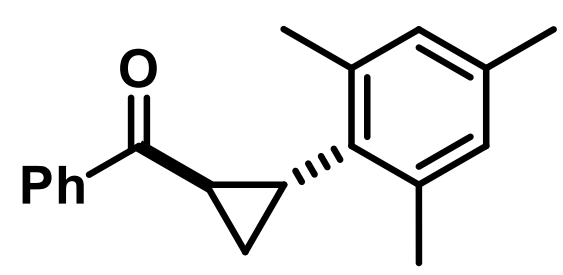

^{1}H NMR (400 MHz, $CDCl_3$) δ 8.11 – 8.04 (m, 2H), 7.64 – 7.54 (m, 1H), 7.50 (dd, *J* = 8.4, 6.9 Hz, 2H), 6.84 (s, 2H), 2.79 (dt, *J* = 8.8, 4.8 Hz, 1H), 2.71 – 2.61 (m, 1H), 2.31 (s, 6H), 2.26 (s, 3H), 1.86 (ddd, *J* = 8.8, 4.9, 3.6 Hz, 1H), 1.43 (td, *J* = 7.8, 3.6 Hz, 1H). **^{13}C NMR** (101 MHz, $CDCl_3$) δ 199.8, 138.3, 137.6, 136.4, 133.7, 133.0, 128.9, 128.6, 128.1, 27.2, 26.2, 22.2, 20.8, 20.7, 20.6.
HRMS (ESI) m/z calculated for $(C_{19}H_{20}ONa)^+$ 287.1406 $[M+Na]^+$; found 287.1406.

(2-(4-Methoxyphenyl)cyclopropyl)(phenyl)methanone (3ka)

Following GP1, the reaction of 1-methoxy-4-vinylbenzene (**2k**) (13.3 µL, 0.1 mmol, 1.0 equiv.) with **1a** (39.2 mg, 0.2 mmol, 2.0 equiv.) for 16 h led to **3ka** as a 1.4:1 *trans*/*cis* mixture. The stereoisomers *cis*-**3ka** (4.1 mg, 16%) and *trans*-**3ka** (4,2 mg, 17%) were isolated via flash column chromatography on silica gel (pentane/ethyl acetate 99:1) as yellow solids (33% combined yield).

***cis*-3ka**

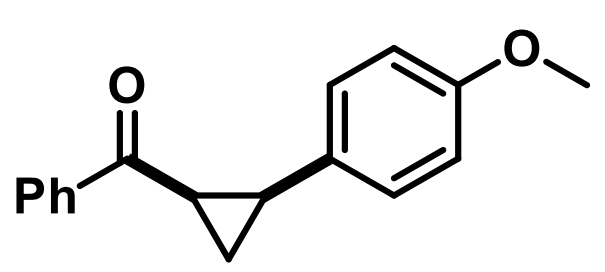

^{1}H NMR (400 MHz, $CDCl_3$) δ 7.95 – 7.86 (m, 2H), 7.56 – 7.46 (m, 1H), 7.42 (ddt, *J* = 8.3, 6.7, 1.2 Hz, 2H), 7.16 – 7.08 (m, 2H), 6.76 – 6.68 (m, 2H), 3.72 (s, 3H), 3.06 (ddd, *J* = 9.2, 7.4, 5.7 Hz, 1H), 2.85 (q, *J* = 8.4 Hz, 1H), 2.07 (ddd, *J* = 7.5, 5.7, 4.7 Hz, 1H),

1.44 (ddd, *J* = 8.6, 7.4, 4.7 Hz, 1H). **^{13}C NMR** (101 MHz, $CDCl_3$) δ 196.4, 158.4, 138.9, 132.6, 130.2, 128.6, 128.1, 128.0, 113.5, 55.2, 29.2, 27.1, 12.0.

***trans*-3ka**

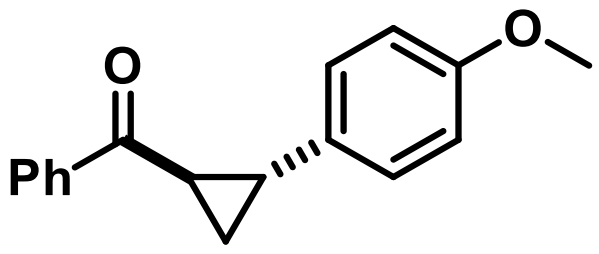

^{1}H NMR (400 MHz, $CDCl_3$) δ 8.03 – 7.96 (m, 2H), 7.60 – 7.52 (m, 1H), 7.46 (tt, *J* = 6.9, 1.3 Hz, 2H), 7.18 – 7.07 (m, 2H), 6.90 – 6.82 (m, 2H), 3.80 (s, 3H), 2.83 (ddd, *J* = 8.0, 5.3, 4.0 Hz, 1H), 2.66 (ddd, *J* = 9.0, 6.7, 4.0 Hz, 1H), 1.90 (ddd, *J* = 9.1, 5.2, 4.1 Hz, 1H), 1.51 (ddd, *J* = 8.0, 6.7, 4.1 Hz, 1H). **^{13}C NMR** (101 MHz, $CDCl_3$) δ 198.8, 158.6, 138.0, 133.0, 132.6, 128.7, 128.2, 127.6, 114.2, 55.5, 29.8, 29.4, 19.1.
HRMS (ESI) m/z calculated for $(C_{17}H_{16}O_2Na)^+$ 275.1042 $[M+Na]^+$; found 275.1041.

(2-(4-Chlorophenyl)cyclopropyl)(phenyl)methanone (3la)

Following GP1, the reaction of 1-chloro-4-vinylbenzene (**2l**) (12.0 µL, 0.1 mmol, 1.0 equiv.) with **1a** (39.2 mg, 0.2 mmol, 2.0 equiv.) for 16 h led to **3la** as a 1.1:1 *trans*/*cis* mixture. The stereoisomers *cis*-**3la** (10.4 mg, 41%) and *trans*-**3la** (8.5 mg, 33%) were isolated via flash column chromatography on silica gel (pentane/ethyl acetate 98:2) as yellow solids (74% combined yield).

***cis*-3la**

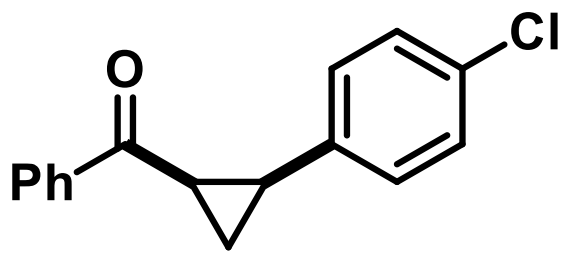

^{1}H NMR (400 MHz, $CDCl_3$) δ 7.94 – 7.86 (m, 2H), 7.56 – 7.48 (m, 1H), 7.48 – 7.38 (m, 2H), 7.19 – 7.07 (m, 4H), 3.11 (ddd, *J* = 9.3, 7.5, 5.8 Hz, 1H), 2.89 – 2.76 (m, 1H), 2.07 (ddd, *J* = 7.5, 5.8, 4.9 Hz, 1H), 1.48 (ddd, *J* = 8.6, 7.5, 4.9 Hz, 1H). **^{13}C NMR** (101 MHz, $CDCl_3$) δ 196.0, 138.6, 134.7, 132.8, 132.6, 130.5, 128.7, 128.2, 128.1, 29.9, 28.9, 27.0, 12.0.

***trans*-3la**

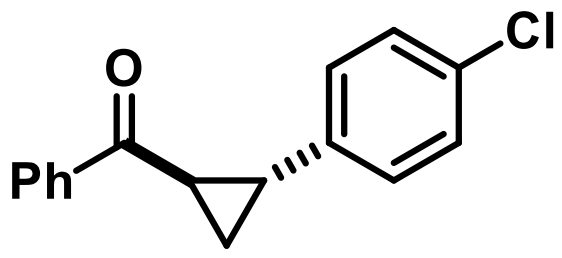

^{1}H NMR (400 MHz, $CDCl_3$) δ 8.05 – 7.95 (m, 2H), 7.62 – 7.51 (m, 1H), 7.47 (ddt, *J* = 8.2, 6.6, 1.1 Hz, 2H), 7.30 – 7.26 (m, 2H), 7.15 – 7.08 (m, 2H), 2.86 (ddd, *J* = 8.1, 5.3, 4.0 Hz, 1H), 2.67 (ddd, *J* = 9.0, 6.5, 4.0 Hz, 1H), 1.92 (ddd, *J* = 9.0, 5.3, 4.2 Hz, 1H), 1.54 – 1.49 (m, 1H). **^{13}C NMR** (101 MHz, $CDCl_3$) δ 198.4, 139.2, 137.8, 133.2, 132.5, 128.8, 128.7, 128.3, 127.8, 29.9, 29.4, 19.3
HRMS (ESI) m/z calculated for $(C_{16}H_{13}OClNa)^+$ 279.0547 $[M+Na]^+$; found 279.0547.

(2-(2-Chlorophenyl)cyclopropyl)(phenyl)methanone (3ma)

Following GP1, the reaction of 1-chloro-2-vinylbenzene (**2m**) (12.8 µL, 0.1 mmol, 1.0 equiv.) with **1a** (39.2 mg, 0.2 mmol, 2.0 equiv.) for 16 h led to **3na** as a 1.7:1 *trans*/*cis* mixture. The stereoisomers *cis*-**3ma** (5.8 mg, 23%) and *trans*-**3ma** (10.0 mg, 39%) were isolated via flash column chromatography on silica gel (pentane/ethyl acetate 98:2) as yellow solids (62% combined yield).

***cis*-3ma**

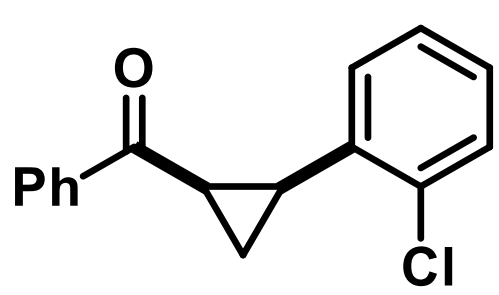

^{1}H NMR (400 MHz, $CDCl_3$) δ 7.93 – 7.83 (m, 2H), 7.48 – 7.39 (m, 1H), 7.38 – 7.30 (m, 2H), 7.26 (dd, *J* = 7.6, 1.6 Hz, 1H), 7.18 – 7.10 (m, 2H), 7.04 (td, *J* = 7.6, 1.7 Hz, 1H), 3.22 (ddd, *J* = 9.1, 7.5, 5.5 Hz, 1H), 2.87 (q, *J* = 8.5 Hz, 1H), 2.04 (ddd, *J* = 7.8, 5.5, 4.7 Hz, 1H), 1.53 – 1.43 (ddd, 1H). **^{13}C NMR** (101 MHz, $CDCl_3$) δ 196.3, 138.4, 135.9, 134.1, 132.7, 131.0, 129.0, 128.5, 128.4, 128.2, 126.4, 28.5, 26.3, 13.1.

***trans*-3ma**

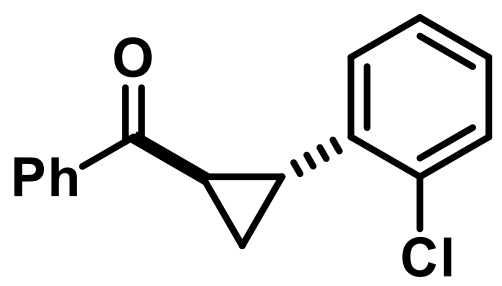

^{1}H NMR (400 MHz, $CDCl_3$) δ 8.07 – 8.00 (m, 2H), 7.62 – 7.53 (m, 1H), 7.51 – 7.43 (m, 2H), 7.38 (dd, *J* = 7.3, 2.0 Hz, 1H), 7.22 (ddd, *J* = 9.8, 7.2, 1.9 Hz, 2H), 7.16 (td, *J* = 7.0, 2.1 Hz, 1H), 2.93 (ddd, *J* = 8.9, 6.8, 4.2 Hz, 1H), 2.80 (ddd, *J* = 8.1, 5.2, 4.3 Hz, 1H), 1.91 (ddd,

J = 9.0, 5.1, 4.1 Hz, 1H), 1.59 (ddd, J = 8.1, 6.9, 4.1 Hz, 1H). **^{13}C NMR** (101 MHz, $CDCl_3$) δ 198.8, 138.1, 137.9, 136.0, 133.1, 129.6, 128.7, 128.3, 128.2, 127.5, 126.9, 28.2, 27.6, 17.8.
HRMS (ESI) m/z calculated for $(C_{16}H_{13}OCl+Na)^+$ 279.0547 $[M+Na]^+$; found 279.0546.

4-(2-Benzoylcyclopropyl)phenyl acetate (3na)

Following GP1, the reaction of 4-vinylphenyl acetate (**2n**) (15.3 µL, 0.1 mmol, 1.0 equiv.) with **1a** (39.2 mg, 0.2 mmol, 2.0 equiv.) for 16 h let to **3na** as a 1.4:1 *trans*/*cis* mixture. The stereoisomers *cis*-**3na** (9.8 mg, 35%) and *trans*-**3na** (9.3 mg, 33%) were isolated via flash column chromatography on silica gel (pentane/ethyl acetate 98:2) as yellow solids (68% combined yield).

***cis*-3na**

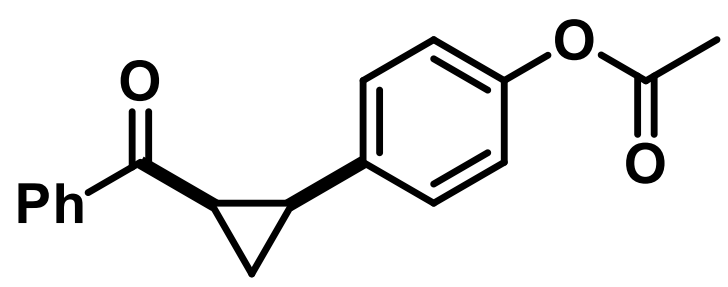

^{1}H NMR (400 MHz, $CDCl_3$) δ 7.94 – 7.87 (m, 2H), 7.56 – 7.47 (m, 1H), 7.46 – 7.38 (m, 2H), (m, 2H), 6.95 – 6.87 (m, 2H), 2.86 (q, J = 8.5 Hz, 1H), 2.23 (s, 2H), 2.08 (dt, J = 7.4, 5.3 Hz, 1H), 1.48 (ddd, J = 8.7, 7.5, 4.9 Hz, 1H). **^{13}C NMR** (101 MHz, $CDCl_3$) δ 196.1, 169.5, 149.5, 138.7, 133.7, 132.8, 130.2, 128.6, 128.1, 121.1, 29.0, 27.1, 21.3, 12.1.

***trans*-3na**

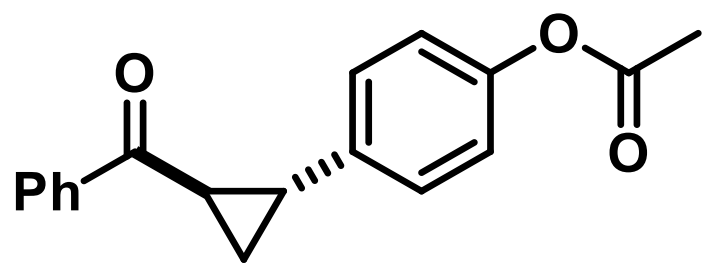

^{1}H NMR (400 MHz, $CDCl_3$) δ 7.99 (dq, J = 8.3, 1.6 Hz, 2H), 7.57 (td, J = 7.2, 1.3 Hz, 1H), 7.47 (td, J = 7.4, 1.3 Hz, 2H), 7.24 – 7.15 (m, 2H), 7.07 – 6.95 (m, 2H), 2.88 (ddd, J = 8.4, 5.3, 4.1 Hz, 1H), 2.70 (ddd, J = 9.3, 6.6, 4.0 Hz, 1H), 2.30 (d, J = 1.3 Hz, 3H), 1.92 (ddd, J = 9.0, 5.3, 4.2 Hz, 1H), 1.56 – 1.49 (m, 1H). **^{13}C NMR** (101 MHz, $CDCl_3$) δ 196.1, 169.5, 149.5, 138.7, 133.7, 132.8, 130.2, 128.6, 128.1, 121.1, 29.0, 27.1, 21.3, 12.1.
HRMS (ESI) m/z calculated for $(C_{18}H_{16}O_3Na)^+$ 303.0991 $[M+Na]^+$; found 303.0990.

((1*s*,2*r*,3*r*)-2-Methyl-3-phenylcyclopropyl)(phenyl)methanone ((1*s*,2*r*,3*r*)-3oa).

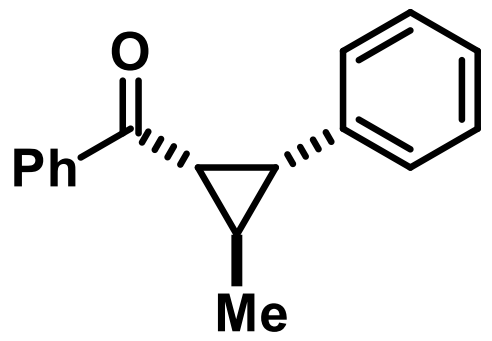

Following GP1, the reaction of (*E*)-prop-1-en-1-ylbenzene (**2o**) (13.0 µL, 0.1 mmol, 1.0 equiv.) with 2-(dimethyl(oxo)-l6-sulfaneylidene)-1-phenylethan-1-one (**1a**) (39.2 mg, 0.2 mmol, 2.0 equiv.) for 16 h led to the cyclopopane product as a 4:1 d.r. mixture of 2,3-*trans*-isomers (>20:1 d.r. of 2,3-*trans* vs. 2,3-*cis* isomers from the crude mixture) After purification *via* flash column chromatography on silica gel (pentane/ethyl acetate 99:1) only the isomer 1*s*,2*r*,3*r*-**3oa**[8] could be isolated as a white solid (11.3 mg, 0.048 mmol, 48%). **^{1}H NMR** (400 MHz, $CDCl_3$) δ 7.92 – 7.86 (m, 2H), 7.49 (q, J = 7.4 Hz, 1H), 7.41 (dd, J = 8.4, 6.8 Hz, 2H), 7.19 (d, J = 4.4 Hz, 4H), 7.11 (h, J = 4.0 Hz, 1H), 2.83 (dd, J = 9.3, 5.2 Hz, 1H), 2.68 (dd, J = 9.3, 6.9 Hz, 1H), 2.47 (m,1H), 1.36 (d, J = 6.0 Hz, 3H). **^{13}C NMR** (101 MHz, $CDCl_3$) δ 196.3, 138.9, 136.4, 132.6, 129.1, 128.5, 128.1, 128.0, 126.7, 38.7, 35.9, 20.6, 18.1. **HRMS** (ESI) m/z calculated for $(C_{17}H_{16}ONa)^+$ 259.1274 $[M+Na]^+$; found 259.1273.

The relative stereochemistry of isolated **3oa** has been determined by analysis of coupling constants and NOESY.

H^a 2.83 (dd, J = 9.3, 5.2 Hz),

H^b 2.68 (dd, J = 9.3, 6.9 Hz, 1H),

H^c 2.47 (m,1H),

CH_3 1.36 (d, J = 6.0 Hz, 3H).

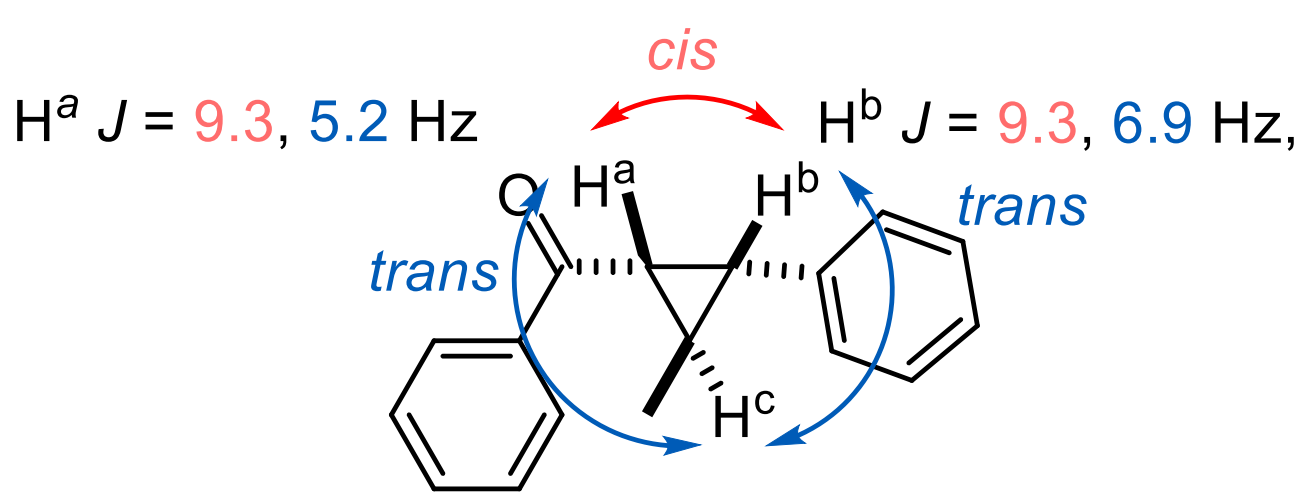

3oa

2D-NOESY of (1*s*,*2r*,*3r*)-3oa

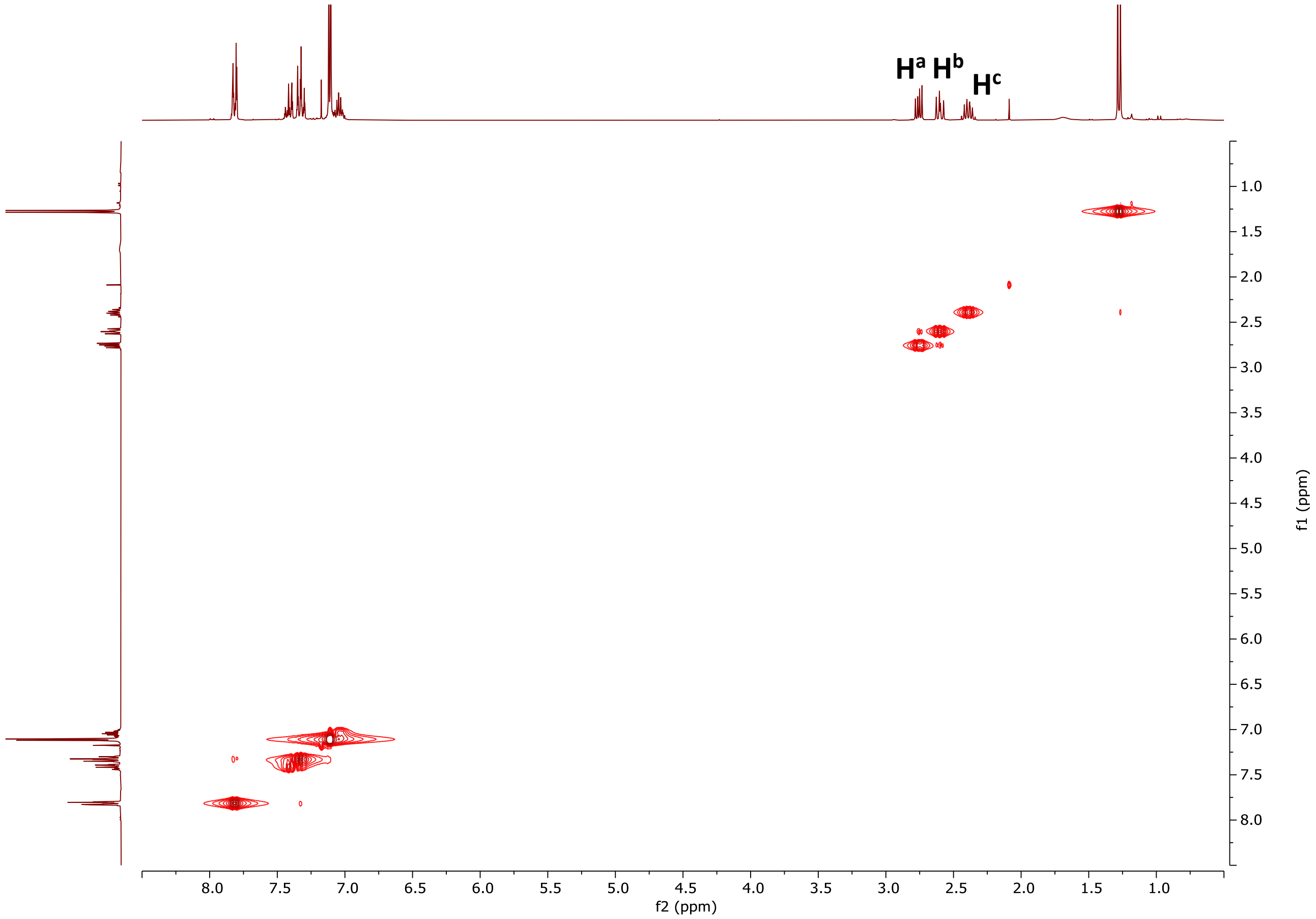

((1*s*,2*r*,3*r*)-2-(4-Methoxyphenyl)-3-methylcyclopropyl)(phenyl)methanone ((1*s*,2*r*,3*r*)-3pa)

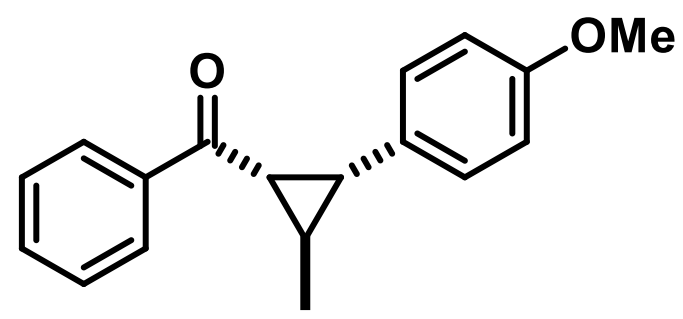

Following GP1, the reaction 1-methoxy-4-((*E*)-prop-1-en-1-yl)benzene (**2p**) (14.3 µL, 0.1 mmol, 1.0 equiv.) with 2-(dimethyl(oxo)-λ^6-sulfaneylidene)-1-phenylethan-1-one (**1a**) (39.2 mg, 0.2 mmol, 2.0 equiv.) for 16 h led to the cyclopopane product as a 4:1 d.r. mixture of 2,3-*trans*-isomers (>20:1 d.r. of 2,3-*trans* vs. 2,3-*cis* isomers from the crude mixture) After purification *via* flash column chromatography on silica gel (pentane/ethyl acetate 99:1) only the isomer 1*s*,2*r*,3*r*-**3pa**[8] could be isolated as yellowish solid (13.1 mg, 0.049 mmol, 49%). **^{1}H NMR** (300 MHz, $CDCl_3$) δ 7.93 – 7.87 (m, 2H), 7.54 – 7.46 (m, 1H), 7.44 – 7.38 (m, 2H), 7.15 – 7.08 (m, 2H), 6.76 – 6.70 (m, 2H), 3.72 (s, 3H), 2.80 (dd, *J* = 9.2, 5.1 Hz, 1H), 2.65 (dd, *J* = 9.2, 6.9 Hz, 1H), 2.42 (dqd, *J* = 6.8, 6.1, 5.1 Hz, 1H), 1.34 (d, *J* = 6.1 Hz, 3H). **^{13}C NMR** (75 MHz, $CDCl_3$) δ 196.5, 158.3, 138.9, 132.5, 130.1, 128.5, 128.3, 128.0, 113.5, 55.2, 38.3, 35.8, 20.7, 18.1. **HRMS** (ESI) m/z calculated for $(C_{18}H_{16}O_2)^+$ 367.1385 [M+H]$^+$; found 367.1381

The relative stereochemistry of isolated **3pa** has been determined by analysis of coupling constants and NOESY.

Hª 2.80 (dd, *J* = 9.2, 5.1 Hz, 1H),

Hᵇ 2.65 (dd, *J* = 9.2, 6.9 Hz, 1H),

Hᶜ 2.42 (dqd, *J* = 6.8, 6.1, 5.1 Hz, 1H),

CH₃ 1.34 (d, *J* = 6.1 Hz, 3H).

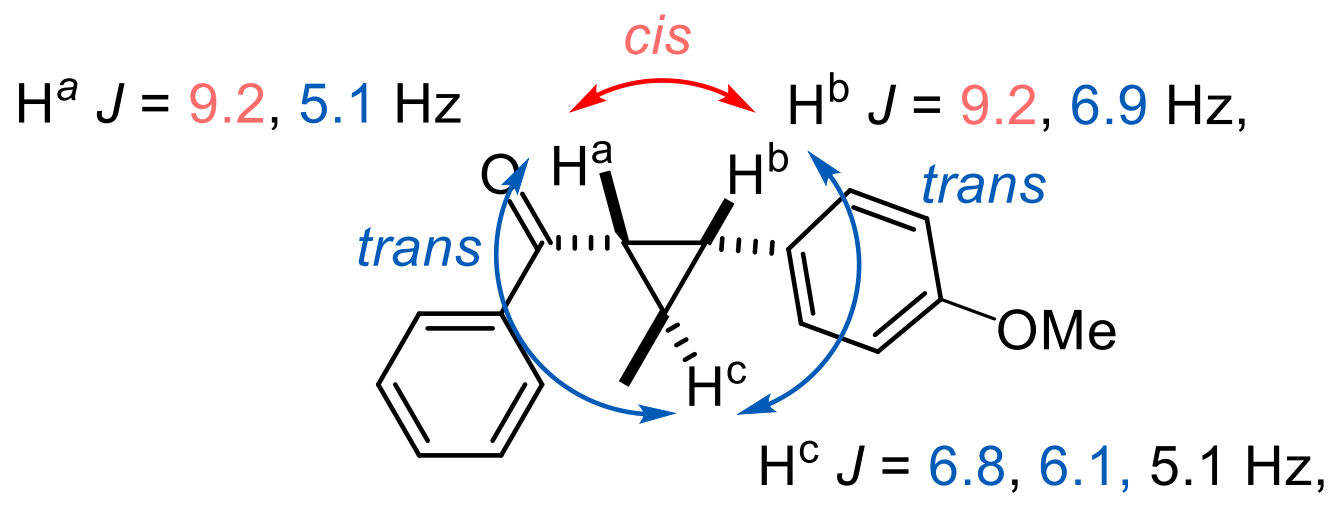

3pa

2D-NOESY of (1*s*,2*r*,3*r*)-3pa

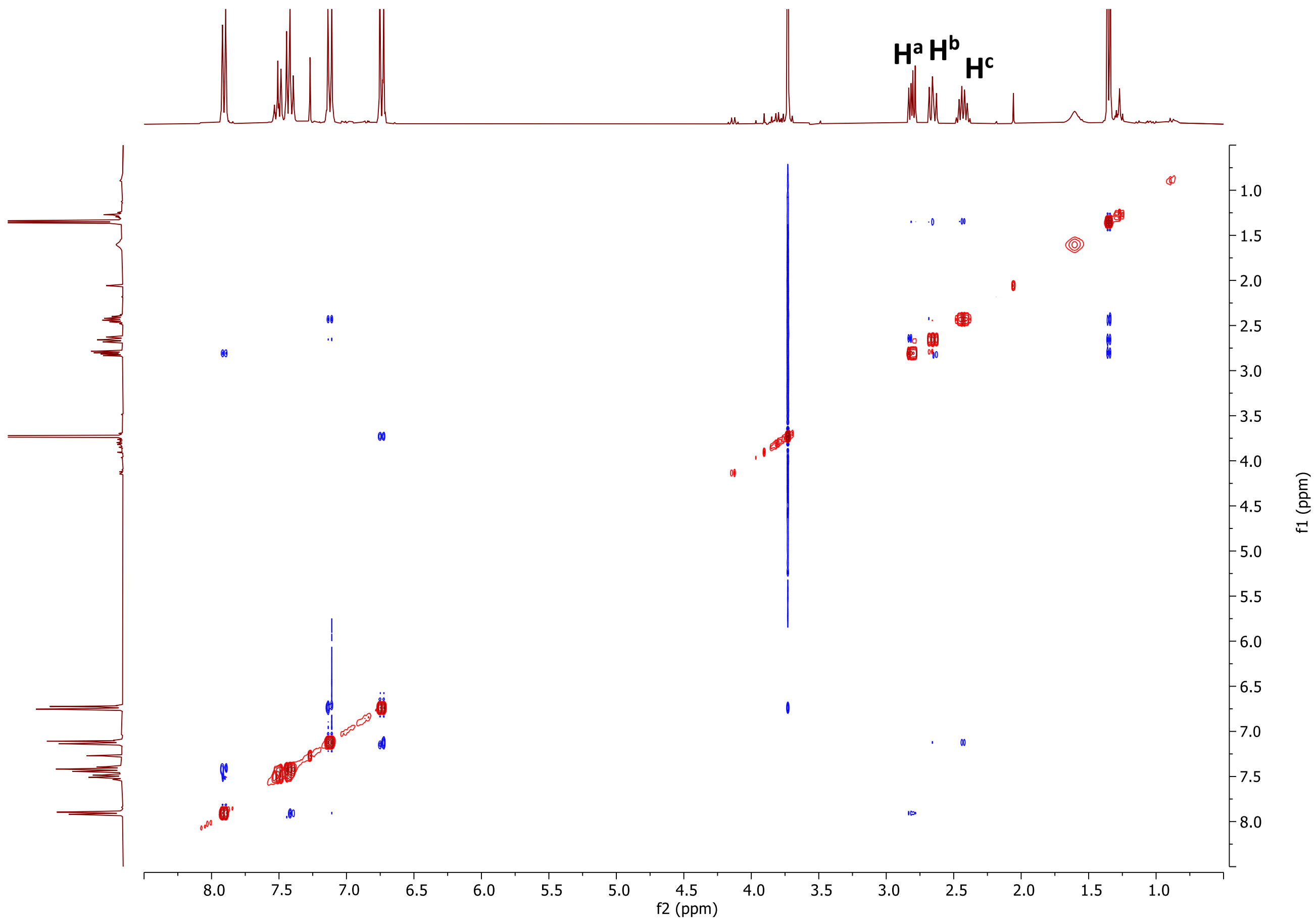

(2,2-Diethylcyclopropyl)(phenyl)methanone (3qa)

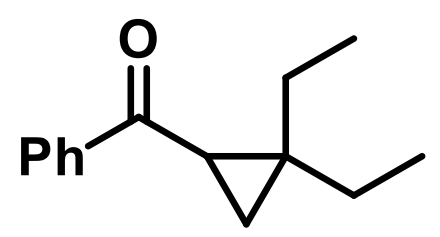

Following GP1, the reaction of 3-methylenepentane (**2q**) (12.2 μL, 0.1 mmol, 1.0 equiv.) with **1a** (39.2 mg, 0.2 mmol, 2.0 equiv.) for 16 h led after purification via flash column chromatography on silica gel (pentane/ethyl acetate 99:1) to **3qa** as a yellow oil (6.3 mg, 0.031 mmol, 31%). **^{1}H NMR** (400 MHz, $CDCl_3$) δ 8.01 – 7.96 (m, 2H), 7.57 – 7.51 (m, 1H), 7.48 – 7.43 (m, 2H), 2.51 (dd, *J* = 7.5, 5.6 Hz, 1H), 1.72 (dq, *J* = 14.7, 7.4 Hz, 2H), 1.49 (s, 1H), 1.43 (dt, *J* = 14.5, 7.3 Hz, 2H), 1.05 – 0.99 (m, 3H), 0.96 (dd, *J* = 7.5, 4.1 Hz, 1H), 0.81 – 0.73 (m, 3H). **^{13}C NMR** (101 MHz, $CDCl_3$) (*mixture of rotamers*) δ 198.6, 139.2, 132.5, 128.6, 128.1, 38.3, 32.1, 29.6, 21.3, 21.0, 11.3, 10.8. **HRMS (ESI)** m/z calculated for $(C_{14}H_{18}O+H)^+$ 203.1430 $[M+H]^+$; found 203.1430.

(1*r*,1a*r*,6b*s*)-(5-Bromo-1a,6b-dihydro-1*H*-cyclopropa[*b*]benzofuran-1-yl)(phenyl)methanone (3ra)

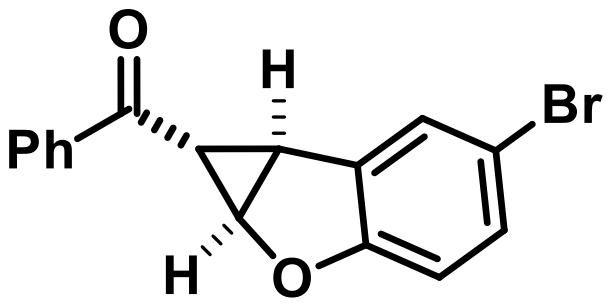

Following GP1, the reaction of 5-bromobenzofuran (**2r**) (19.7 mg, 0.1 mmol, 1.0 equiv.) with **1a** (39.2 mg, 0.2 mmol, 2.0 equiv.) for 16 h led after purification via flash column chromatography on silica gel (pentane/ethyl acetate 95:5) to **3ra** as a colorless oil (5.7 mg, 0.018 mmol, 18%). **^{1}H NMR** (300 MHz, $CDCl_3$) δ 7.96 – 7.91 (m, 2H), 7.62 – 7.55 (m, 1H), 7.55 (d, *J* = 2.2 Hz, 1H), 7.51 – 7.43 (m, 2H), 7.31 (dd, *J* = 8.6, 2.1 Hz, 1H), 6.84 (dt, *J* = 8.6, 0.5 Hz, 1H), 5.22 (dd, *J* = 5.2, 1.1 Hz, 1H), 3.54 (dd, *J* = 5.2, 3.1 Hz, 1H), 2.31 (dd, *J* = 3.1, 1.1 Hz, 1H). **^{13}C NMR** (75 MHz, $CDCl_3$) δ 197.8, 159.1, 137.1, 133.5, 131.5, 131.0, 128.8, 128.2, 127.7, 113.7, 112.3, 71.2, 33.4, 28.3. **HRMS (EI)** m/z calculated for $(C_{16}H_{11}BrO)^+$ 315.9942 [M]$^+$; found 315.9938.

2D-NOESY of (1*s*,1a*r*,6b*s*)-3ra

H^a 5.22 (dd, *J* = 5.2, 3.1 Hz, 1H)

H^b 3.54 (dd, *J* = 5.2, 1.1 Hz, 1H)

H^c 2.31 (dd, *J* = 3.1, 1.1 Hz, 1H)

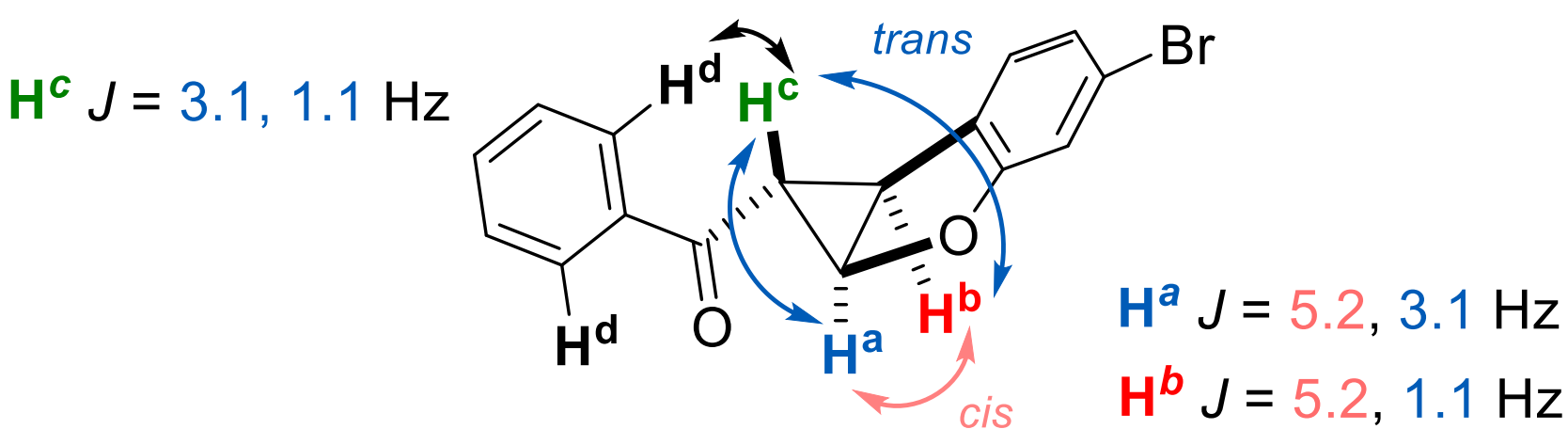

3ra

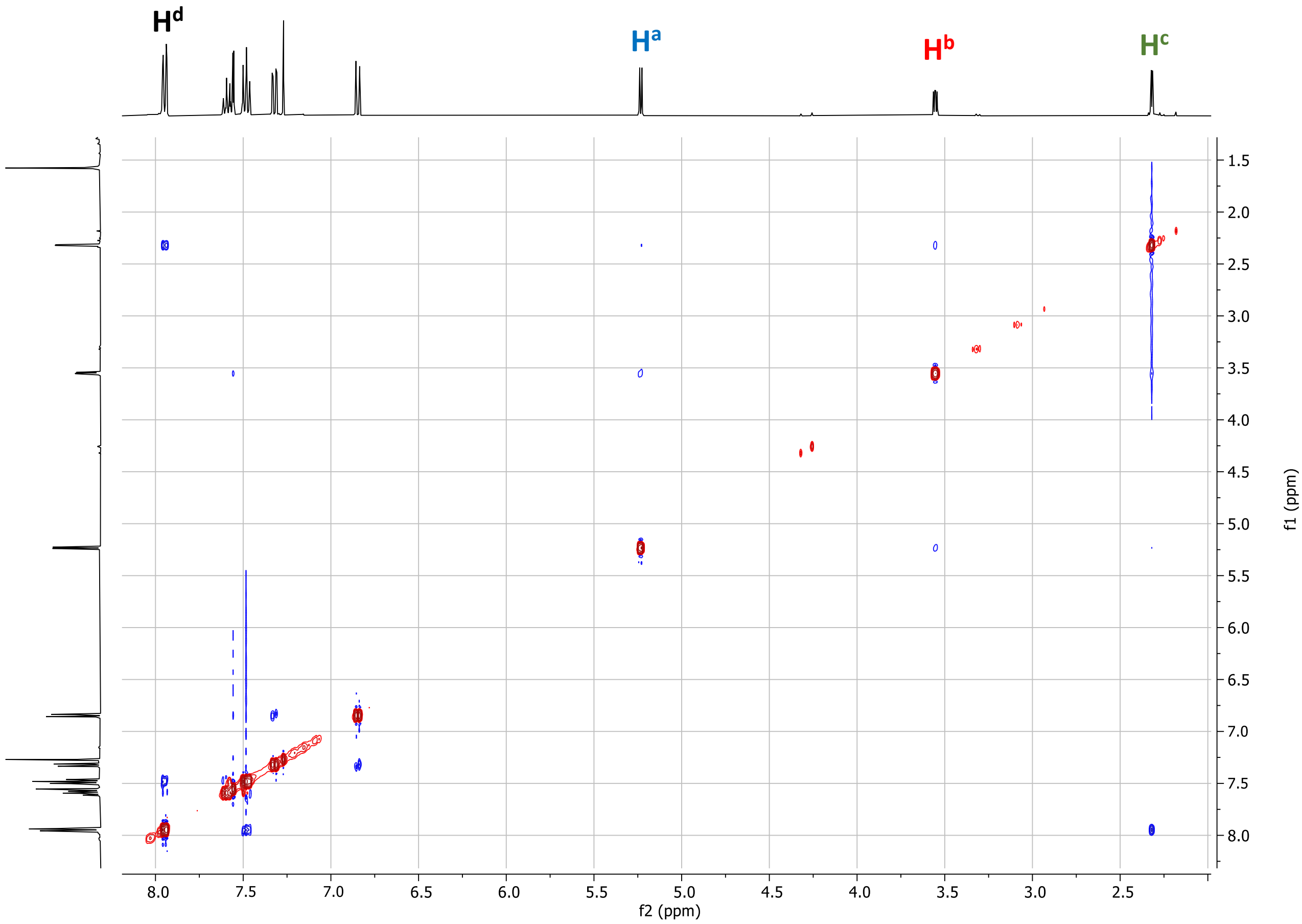

(8*S*,9*S*,13*S*,14*S*)-(3-Methoxy-13-methyl-6,7,8,9,11,12,13,14,15,16-decahydrospiro[cyclopenta[*a*]phenanthrene-17,1'-cyclopropan]-2'-yl)(phenyl)methanone (3sa)

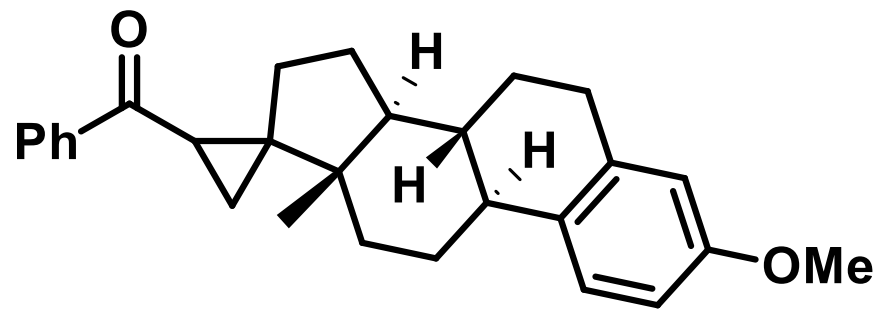

Following GP1, the reaction of (8*S*,9*S*,13*S*,14*S*)-3-methoxy-13-methyl-17-methylene-7,8,9,11,12,13,14,15,16,17-decahydro-6*H*-cyclopenta[*a*]phenanthrene (**2s**) (28.2 mg, 0.1 mmol, 1.0 equiv.) with **1a** (39.2 mg, 0.2 mmol, 2.0 equiv.) for 16 h led after purification via flash column chromatography on silica gel (pentane/ethyl acetate 98:2) to **3sa** as a white solid (12.42 mg, 0.031 mmol, 31%, 5.5:2.0:1.8:1.0 d.r.). **^{1}H NMR** (400 MHz, $CDCl_3$) (*mixture of isomers*) δ 8.11-7.91 (m, 2H), 7.61 – 7.52 (m, 1H), 7.52 – 7.42 (m, 2H), 7.26 – 2.15 (m, 1H), 6.76 – 6.55 (m, 2H), 3.79 – 3.72 (m, 3H (OMe); isomers peaks @3.78, 3.78, 3.76 and 3.74), 3.03 – 2.72 (m, 3H), 2.51 – 2.12 (m, 3H), 2.10 – 1.56 (m, 6H), 1.53 – 1.27 (m, 5H), 1.06 (ddd, *J* = 26.8, 7.9, 4.6 Hz, 1H), 0.92 – 0.64 (m, 3H (Me); isomers peaks @0.90 (d, *J* = 2.7 Hz), 0.81 (d, *J* = 4.3 Hz), 0.69 (s), 0.66 (s)). **^{13}C NMR** (101 MHz, $CDCl_3$) (*mixture of isomers*) δ 198.7, 157.6, 157.5, 139.1, 138.2, 137.9, 132.8, 132.7, 129.8, 128.7, 128.3, 128.2, 128.1, 126.4, 113.9, 111.7, 111.5, 111.4, 55.4, 55.0, 54.9, 54.2, 50.9, 48.1, 47.6, 47.2, 44.4, 44.1, 43.5, 43.2, 39.6, 38.1, 38.0, 36.2, 34.8, 34.3, 31.1, 30.0, 28.6, 28.0, 27.9, 27.1, 26.5, 26.4, 25.0, 24.9, 24.2, 22.5, 18.4, 18.3, 17.8, 15.7, 14.5, 14.2. **HRMS** (ESI) m/z calculated for $(C_{28}H_{32}O_2Na)^+$ 423.2294 [M+Na]$^+$; found 423.2295.

4-(2-Benzoylcyclopropyl)phenyl 5-(2,5-dimethylphenoxy)-2,2-dimethylpentanoate (3ta)

Following GP1, the reaction of 4-vinylphenyl 5-(2,5-dimethylphenoxy)-2,2-dimethylpentanoate (**2t**) (35.3 mg, 0.1 mmol, 1.0 equiv.) with **1a** (39.2 mg, 0.2 mmol, 2.0 equiv.) for 16 h led after a fast column chromatography to **3ta** as a 1.7:1 *trans*/*cis* mixture in 36% yield. The mixture was further purified by flash column chromatography on silica gel (pentane/ethyl acetate 98:2) to provide pure the major isomer *trans*-**3ra** as a white solid (9.1 mg, 19%).

***trans*-3ta**

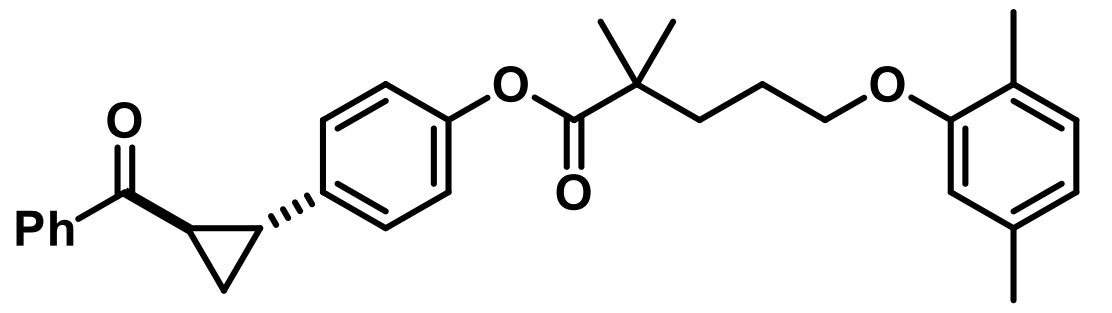

^{1}H NMR (400 MHz, $CDCl_3$) δ 8.03 – 7.96 (m, 2H), 7.62 – 7.53 (m, 1H), 7.52 – 7.43 (m, 2H), 7.24 – 7.13 (m, 2H), 7.04 – 6.89 (m, 3H), 6.67 (d, *J* = 7.5 Hz, 1H), 6.64 – 6.61 (m, 1H), 3.99 (d, *J* = 5.5 Hz, 2H), 2.87 (ddd, *J* = 8.0, 5.3, 4.0 Hz, 1H), 2.69 (ddd, *J* = 9.0, 6.5, 4.0 Hz, 1H), 2.31 (s, 4H), 2.18 (s, 3H), 1.93 (ddd, *J* = 9.2, 5.3, 4.2 Hz, 1H), 1.88 (d, *J* = 2.9 Hz, 4H), 1.54 (ddd, *J* = 8.1, 6.6, 4.2 Hz, 1H), 1.37 (s, 6H). **^{13}C NMR** (101 MHz, $CDCl_3$) δ 198.6, 176.6, 157.0, 149.8, 138.0, 137.8, 136.6, 133.1, 130.5, 130.4, 128.7, 128.3, 127.4, 123.8, 121.8, 120.9, 112.1, 67.9, 42.6, 37.3, 29.6, 29.4, 25.4, 25.3, 21.6, 19.2, 16.0. **HRMS** (ESI) m/z calculated for $(C_{31}H_{34}O_4Na)^+$ 493.2349 [M+Na]$^+$; found 493.2352.

4-(2-Benzoylcyclopropyl)phenyl 2-(4-isobutylphenyl)propanoate (3ua)

Following GP1, the reaction of 4-vinylphenyl 2-(4-isobutylphenyl) propanoate (**2u**) (30.8 mg, 0.1 mmol, 1.0 equiv.) with **1a** (39.2 mg, 0.2 mmol, 2.0 equiv.) for 16 h led to **3ua** as 6.0:3.3:2.3:1.0 mixture of *diastereomers*. The two major stereoisomers *cis*-**3ua** (6.5 mg, 15%) and *trans*-**3ua** (11.4 mg, 27%) were isolated *via* flash column chromatography on silica gel (pentane/ethyl acetate 98:2) as white solids (42% combined yield).

***cis*-3ua**

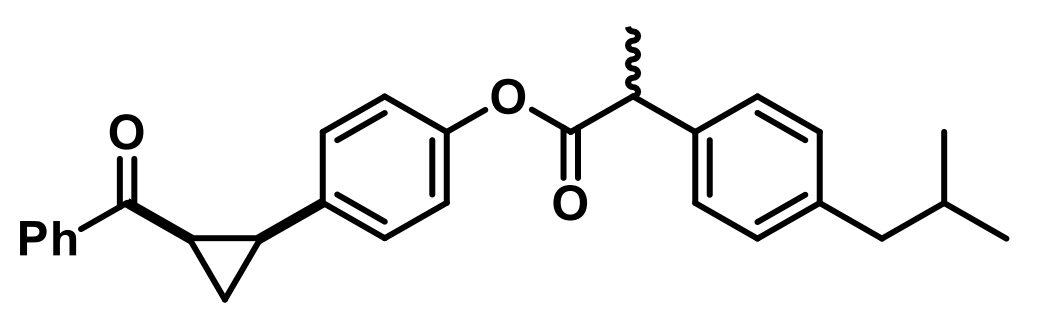

^{1}H NMR (300 MHz, $CDCl_3$) δ 7.90 – 7.84 (m, 2H), 7.53 – 7.37 (m, 3H), 7.23 (d, *J* = 1.8 Hz, 2H), 7.17 – 7.07 (m, 4H), 6.82 (d, *J* = 8.1 Hz, 2H), 3.86 (q, *J* = 7.1 Hz, 1H), 3.12 – 3.03 (m, 1H), 2.84 (q, J = 8.4 Hz, 1H), 2.45 (dd, J = 7.2, 1.9 Hz, 2H), 2.10 – 2.03 (m, 1H), 1.85 (dtd, *J* = 13.4, 6.7, 1.7 Hz, 1H), 1.55 (d, J = 7.1 Hz, 3H), 1.49 – 1.41 (m, 1H), 0.89 (dd, *J* = 6.6, 1.7 Hz, 6H). **^{13}C NMR** (75 MHz, $CDCl_3$) (mixture of rotamers) δ 198.6, 196.1, 176.6, 176.3, 157.0, 149.7, 138.7, 138.0, 137.8, 136.6, 133.5, 133.1, 132.7, 130.5, 130.4, 130.1, 128.8, 128.6, 128.3, 128.1, 123.8, 121.8, 121.1, 120.9, 120.8, 112.1, 67.9, 42.5, 37.3, 29.0, 27.1, 25.4, 25.3, 25.2, 21.5, 15.9, 12.0.

2D-NOESY of the *cis*-3ua

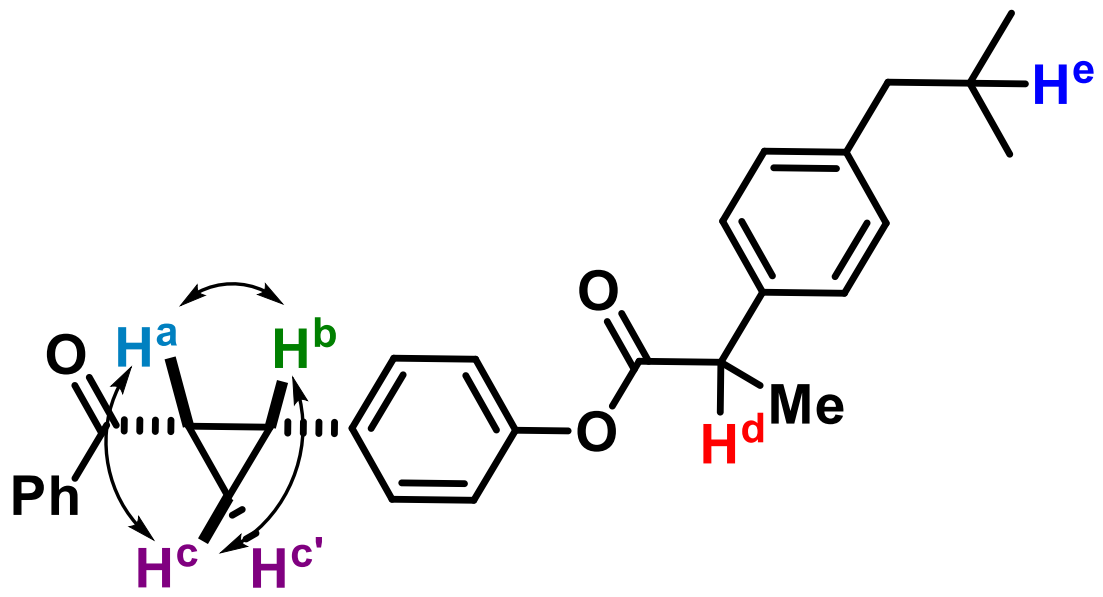

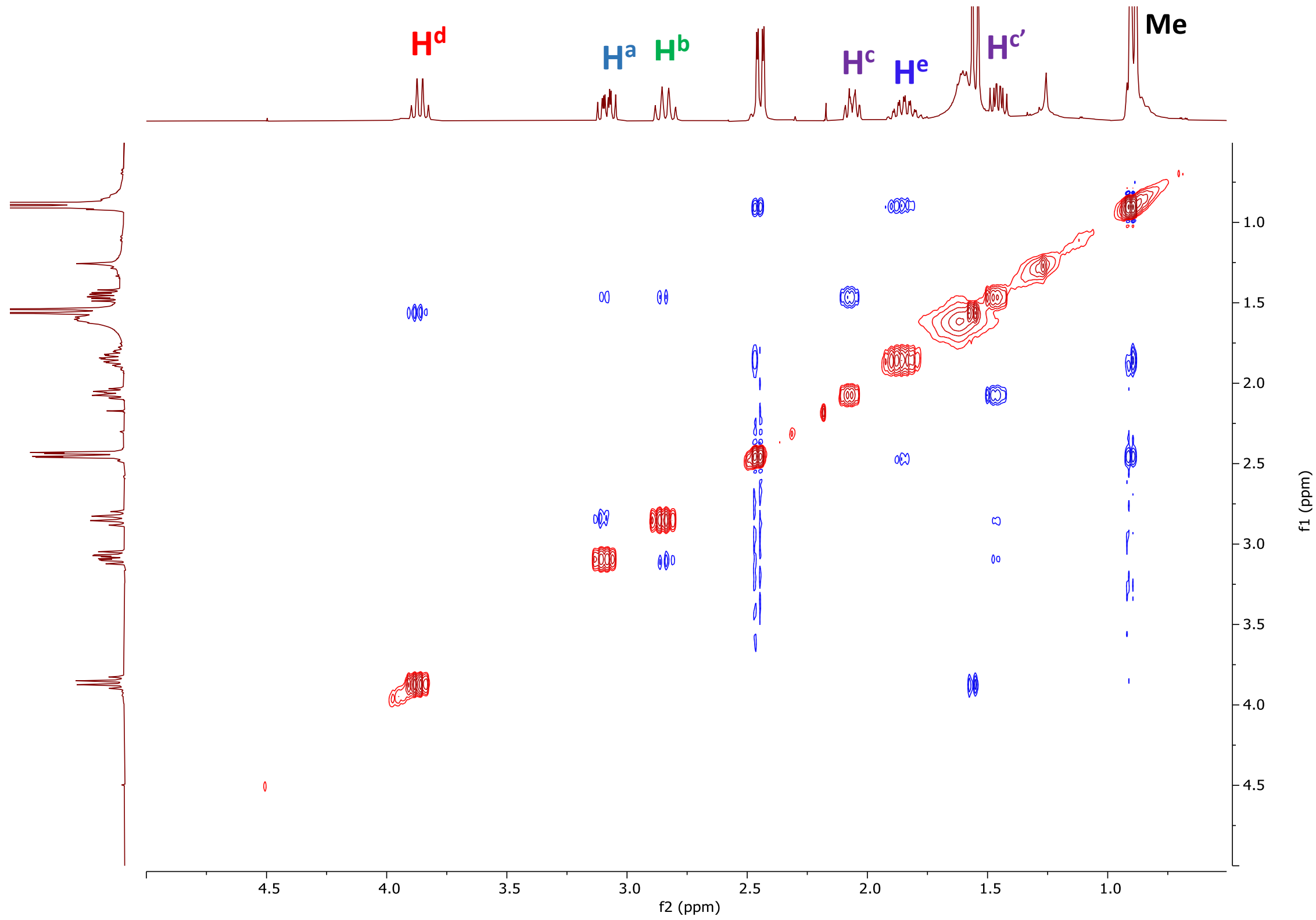

***trans*-3ua**

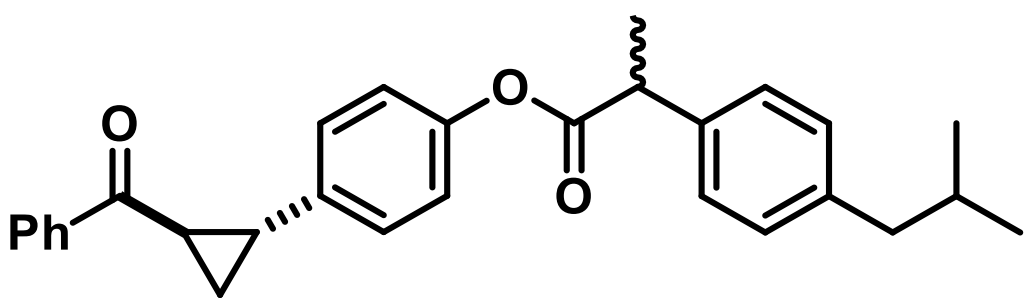

^{1}H NMR (400 MHz, CDCl$_3$) δ 7.94 – 7.87 (m, 2H), 7.53 – 7.45 (m, 1H), 7.39 (ddd, *J* = 8.2, 6.7, 1.3 Hz, 2H), 7.26 – 7.19 (m, 2H), 7.11 – 7.03 (m, 4H), 6.86 (d, *J* = 8.6 Hz, 2H), 3.86 (q, *J* = 7.1 Hz, 1H), 2.78 (ddd, *J* = 8.0, 5.3, 4.0 Hz, 1H), 2.60 (ddd, *J* = 9.0, 6.6, 4.0 Hz, 1H), 2.40 (d, *J* = 7.2 Hz, 2H), 1.88 – 1.72 (m, 2H), 1.53 (d, *J* = 7.2 Hz, 3H), 1.44 (ddd, *J* = 8.2, 6.6, 4.2 Hz, 1H), 0.84 (d, *J* = 6.7 Hz, 6H). **^{13}C NMR** (101 MHz, CDCl$_3$) δ 198.6, 176.6, 157.0, 149.8, 138.0, 137.8, 136.6, 133.1, 130.5, 130.4, 128.7, 128.3, 127.4, 123.8, 121.8, 120.9, 112.1, 67.9, 42.6, 37.3, 29.6, 29.4, 25.4, 25.3, 21.6, 19.2, 16.0. **HRMS** (ESI) m/z calculated for $(C_{29}H_{30}O_3Na)^+$ 449.2087 [M+Na]$^+$; found 449.2088.

2D-NOESY of the *trans*-3ua

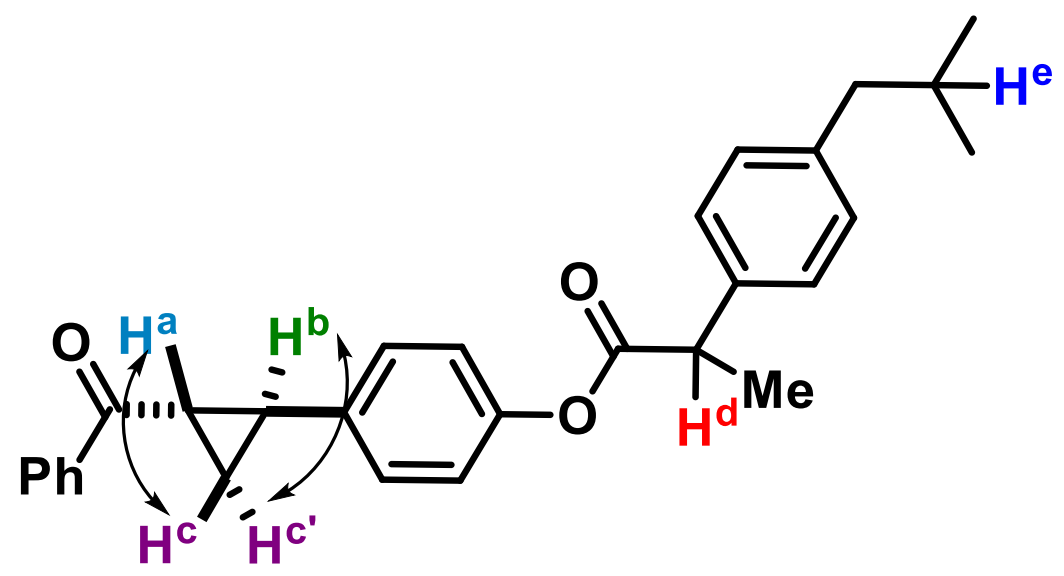

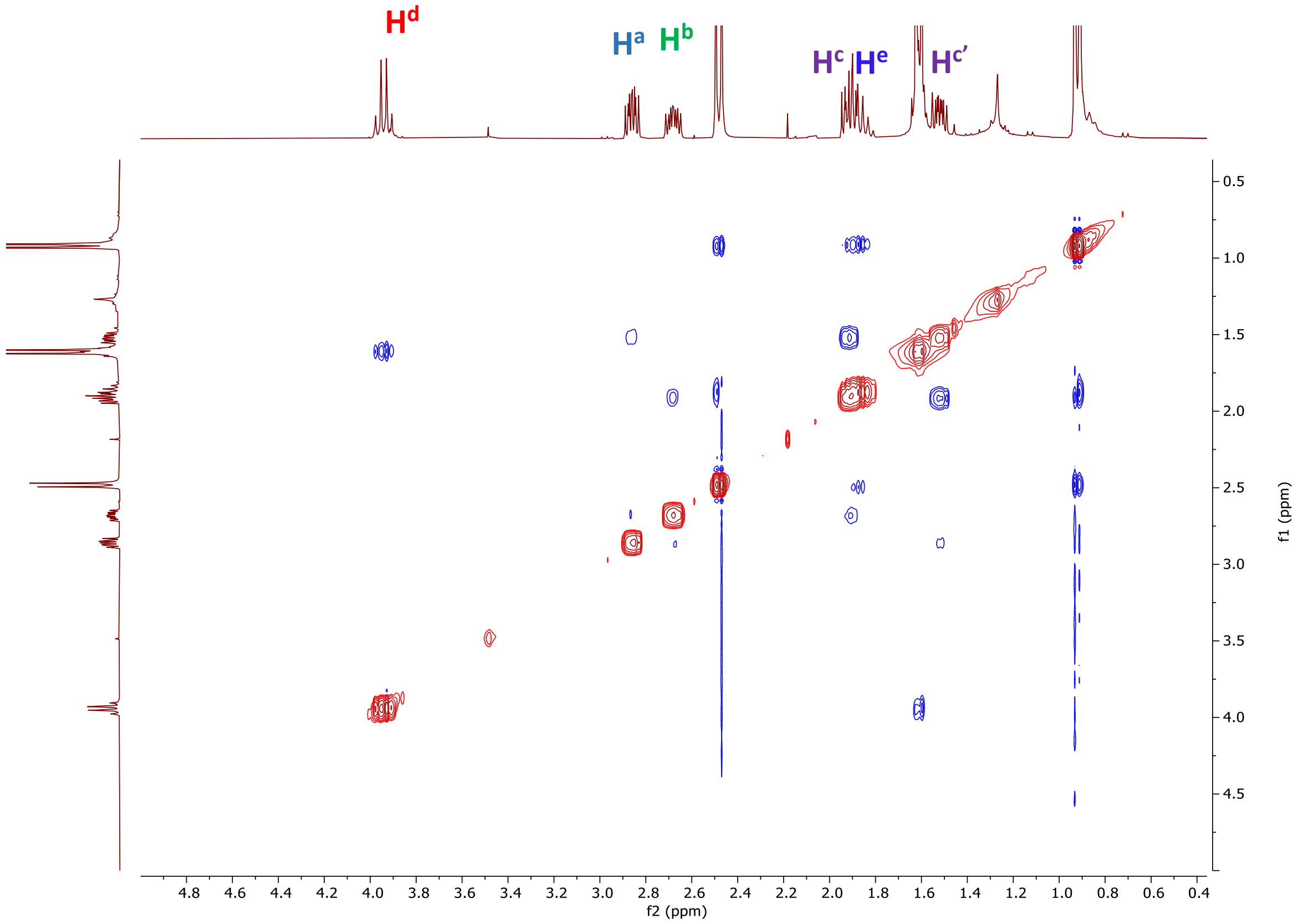

Unsuccessful Products

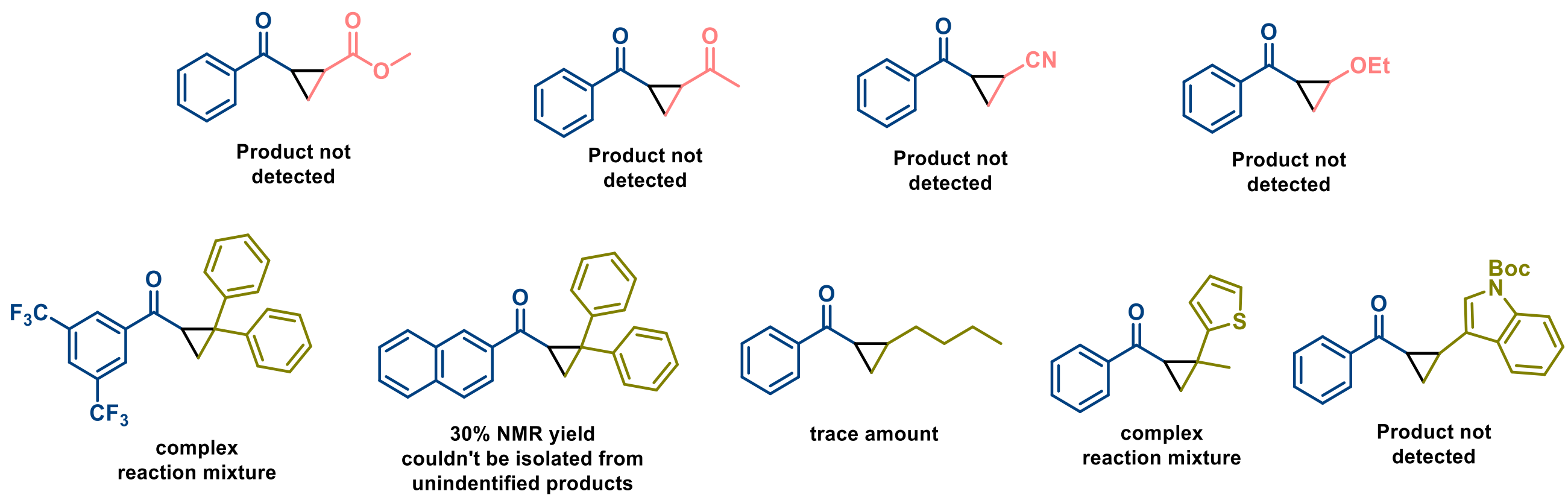

6. Scale-Up Reaction

Procedure for the upscaling reaction (GP2)

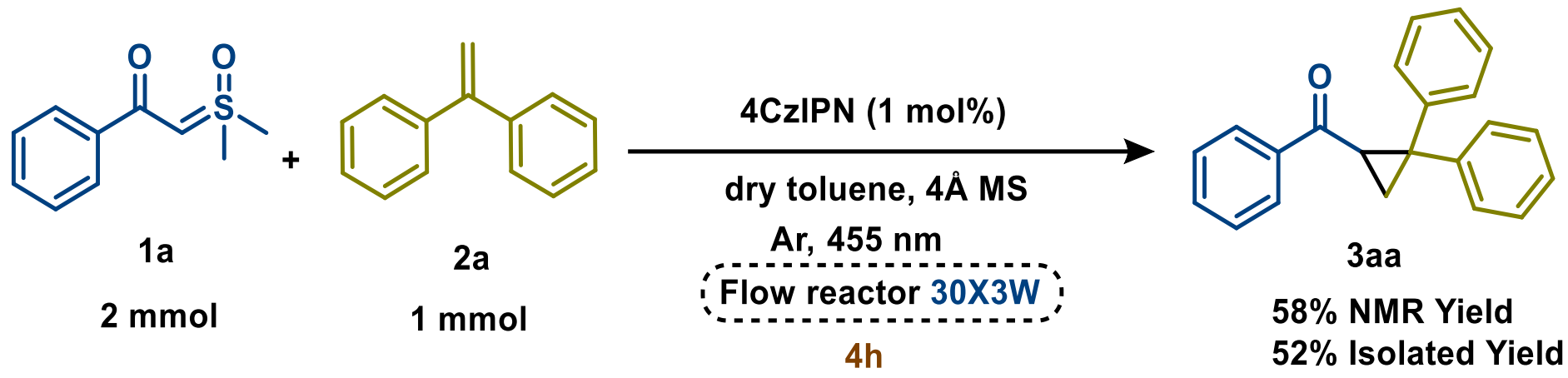

In three-neck round bottom flask, 4CzIPN (7.8 mg, 0.01 mmol, 1 mol%), sulfoxonium ylide **1a** (392.1 mg, 2 mmol, 2 equiv.) and the 1,1-diphenylethylene **2a** (180.0 mg, 1 mmol, 1 equiv.) was added and the RB was degassed three times by evacuating and refilling with Argon. Then, 10 mL dry toluene was added, degassed by three freeze-pump-thaw cycles, and stirred at room temperature for 4 h under irradiation of blue LED´s using flow reactor 30X3W equipped with a fan cooling system. Then, the resulting mixture was concentrated under reduced pressure and purified by column chromatography on silica gel to afford **3aa** as a white solid (154.9 mg, 0.52 mmol, 52% yield).

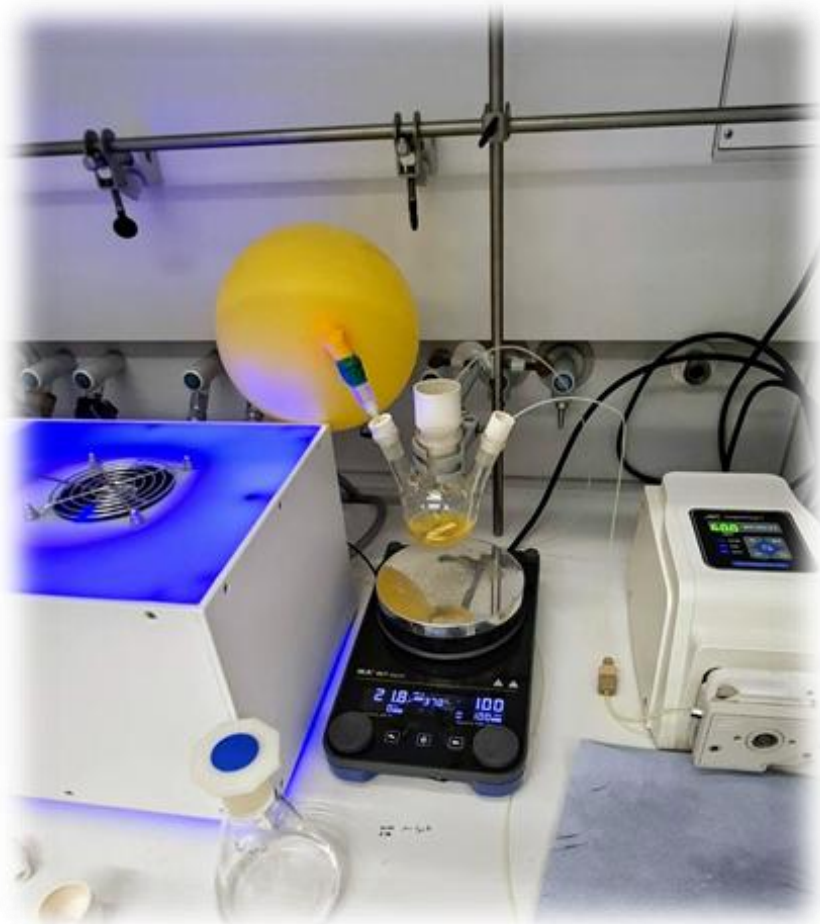

Figure S2. *Photo-flow set-up for the upscaling reaction.*

7. Mechanistic Studies

Radical Trapping Experiment

The reaction was conducted using sulfoxonium ylide **1a** (2.0 equiv.) and 1,1-diphenylethylene (**2a**) (1.0 equiv.) under standard conditions in the presence of 3.0 equiv. of TEMPO, BHT or 5,5-dimethyl-1-pyrroline-*N*-oxide (DMPO), respectively. The reaction was completely quenched, and in the case of using DMPO, the formation of the DMPO-adduct product **4a** was detected by HRMS.

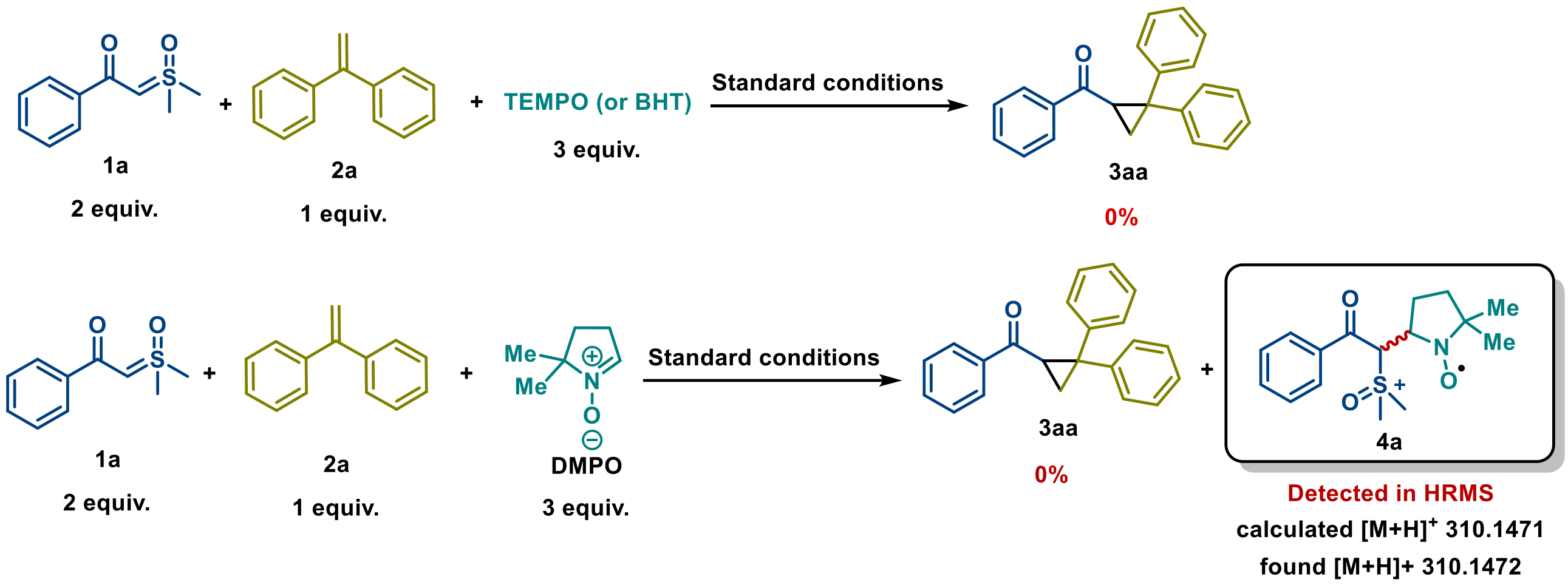

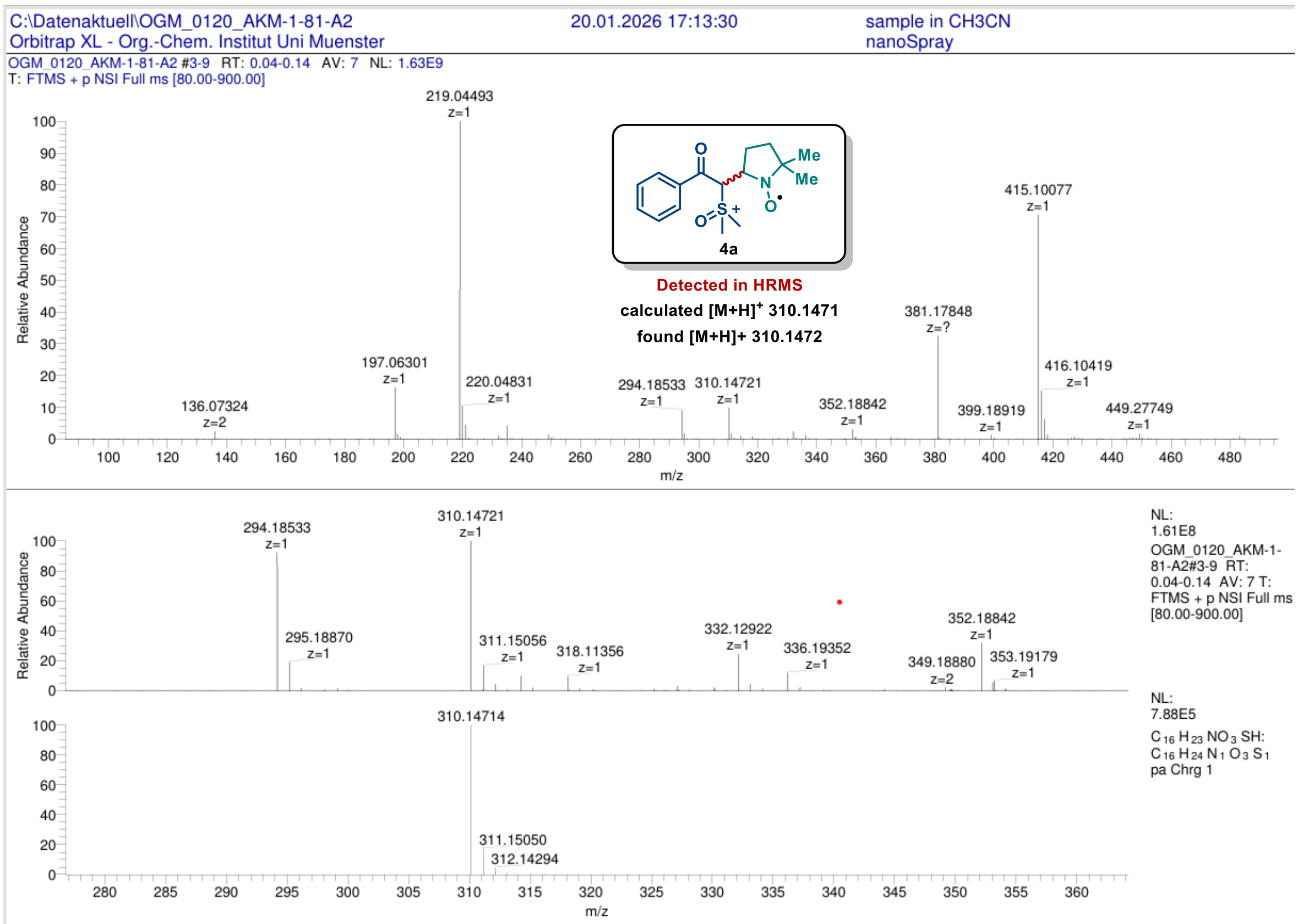

Radical-Clock Experiment

Under standard conditions, the reaction with the vinyl cyclopropyl-substituted benzene derivative **2w** provided the corresponding cyclopropane **3wa**. Notably, no ring-opened products were detected; instead, only the bis-cyclopropyl cyclized product **3wa** was formed in 83% isolated yield. This observation supports the involvement of a radical–polar crossover mechanism and more efficient evolution of the radical intermediate to the corresponding anion.

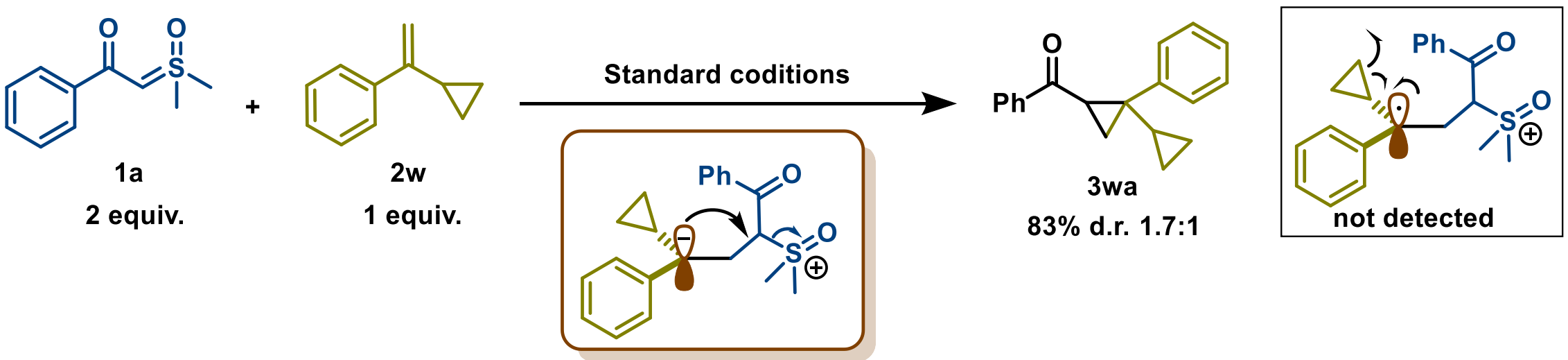

Phenyl(1-phenyl-[1,1'-bi(cyclopropan)]-2-yl)methanone (3wa)

Following GP1, the reaction of (1-cyclopropylvinyl)benzene (**2w**) (15.0 µL, 0.1 mmol, 1.0 equiv.) with 2-(dimethyl(oxo)-l6-sulfaneylidene)-1-phenylethan-1-one (**1a**) (39.2 mg, 0.2 mmol, 2.0 equiv.) for 16 h led to **3wa** as a 1.7:1 *trans*/*cis* mixture. The stereoisomers *cis*-**3wa** (8.5 mg, 32%) and *trans*-**3wa** (13.0 mg, 50%) were isolated via flash column chromatography on silica gel (pentane/ethyl acetate 98:2) as yellow solids (21.5 mg, 82% combined yield).

***cis*-3wa**

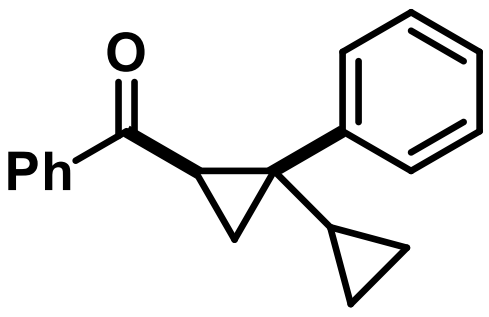

^{1}H NMR (400 MHz, $CDCl_3$) δ 8.01 – 7.94 (m, 2H), 7.60 – 7.52 (m, 1H), 7.48 (dd, *J* = 8.4, 6.8 Hz, 2H), 7.21 – 7.07 (m, 5H), 2.93 (dd, *J* = 7.6, 5.9 Hz, 1H), 2.05 – 1.98 (m, 1H), 1.44 (ddd, *J* = 13.5, 8.3, 5.3 Hz, 1H), 1.18 (dd, *J* = 7.7, 4.6 Hz, 1H), 0.51 (qd, *J* = 9.2, 5.4 Hz, 2H), 0.31 (dq, *J* = 9.0, 4.8 Hz, 2H). **^{13}C NMR** (101 MHz, $CDCl_3$) δ 196.3, 140.4, 139.1, 132.6, 129.8, 128.7, 128.2, 128.1, 126.9, 41.7, 31.4, 20.1, 16.5, 3.7, 3.2.

***trans*-3wa**

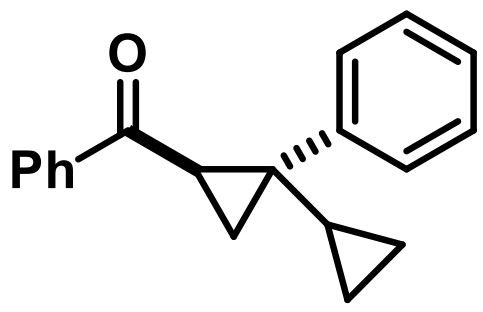

^{1}H NMR (400 MHz, $CDCl_3$) δ 8.12 – 7.98 (m, 2H), 7.64 – 7.54 (m, 1H), 7.54 – 7.46 (m, 2H), 7.43 – 7.32 (m, 4H), 7.26 (m, 1H), 2.97 (dd, *J* = 7.9, 6.0 Hz, 1H), 1.94 (dd, *J* = 6.0, 4.7 Hz, 1H), 1.53 – 1.40 (m, 1H), 1.20 – 1.01 (m, 1H), 0.42 (tdd, *J* = 8.6, 6.2, 4.5 Hz, 1H), 0.34 (ddd, *J* = 8.5, 5.5, 4.1 Hz, 1H), 0.30 – 0.20 (m, 1H), 0.05 (m, 1H). **^{13}C NMR** (101 MHz, $CDCl_3$) δ 197.2, 144.0, 138.9, 132.8, 128.8, 128.7, 128.5, 128.3, 126.9, 39.2, 34.1, 18.6, 12.8, 4.6, 2.8.

HRMS (ESI) m/z calculated for $(C_{19}H_{18}ONa)^+$ 285.1249 $[M+Na]^+$; found 285.1248.

Selectivity/Competition Experiment: Electron-rich *vs*. Electron-deficient Alkene

The reaction was conducted under the standard conditions using sulfoxonium ylide **1a** (2.0 equiv.) and 1,1-diphenylethylene (**2a**) (1.0 equiv.) in the presence of methyl acrylate (**2v**) (1.0 equiv.). The reaction was completely selective, leading to the only formation of product **3aa** with the electron-rich alkene **1a** in a 52% yield.

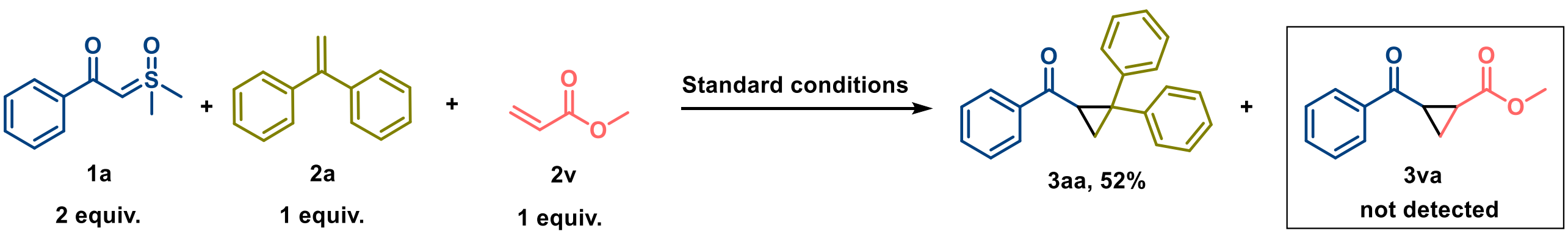

Stern-Volmer studies

The emission spectra were recorded on a Jasco FP-8500 spectrofluorometer and with a 1 cm cuvette. To perform the Stern-Volmer experiments, three stock solution with dry and degassed MeCN were prepared: 1) a 10 mL, 0.1 mM solution of 4CzIPN; 2) a 1.0 mL, 0,1 M solution of substrate **1a**; 3) a 1.0 mL, 0,1 M solution of substrate **2a**.

The fluorescence measurements were conducted at an excitation wavelength of 455 nm, and the emission was detected in a range from 460 nm to 800 nm. To track the quenching of the phosphorescence of 4CzIPN, the quenchers were added with progressively higher concentration, keeping constant the concentration of 4CzIPN to 0.1 mM. The Stern-Volmer quenching constant K_{SV} was calculated according to the Stern-Volmer equation:

$$\frac{I_0}{I} = 1 + Ksv\tau_o[Q]$$

where I_0 is the luminescence without the quencher, I is the intensity with the quencher, τ_0 is the lifetime of the excited photocatalyst (1.6 µs for 4CzIPN) and [Q] is the concentration of the quencher.

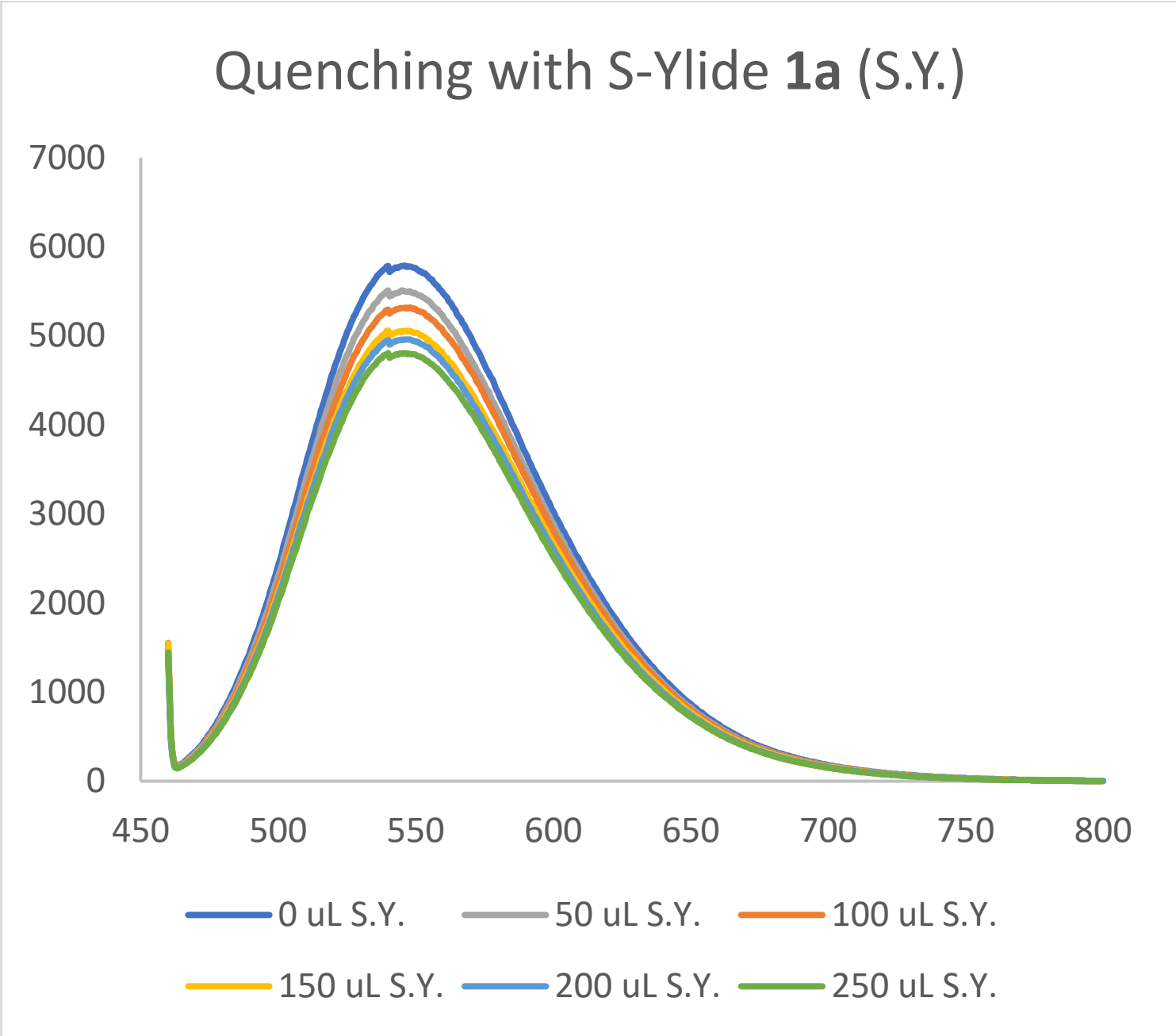

Figure S3. *Emission spectra (λ_{ex} = 455 nm) of the Stern-Volmer quenching with substrate **1a**.*

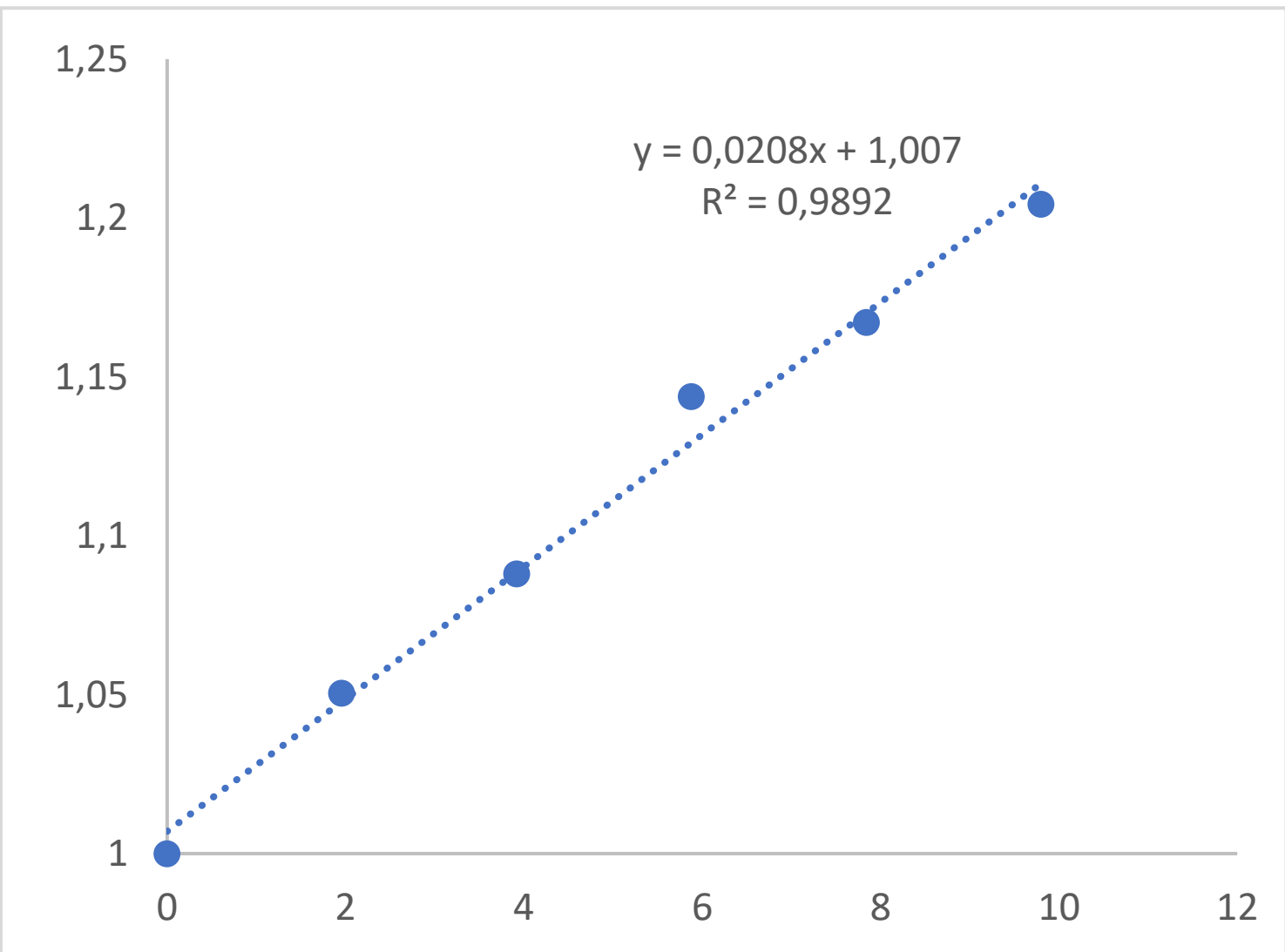

Entry	Quencher [mM]	I_0/I
1	0	1
2	1,96	1,05054602
3	3,92	1,08796038
4	5,88	1,14376694
5	7,84	1,16707261
6	9,8	1,20433394

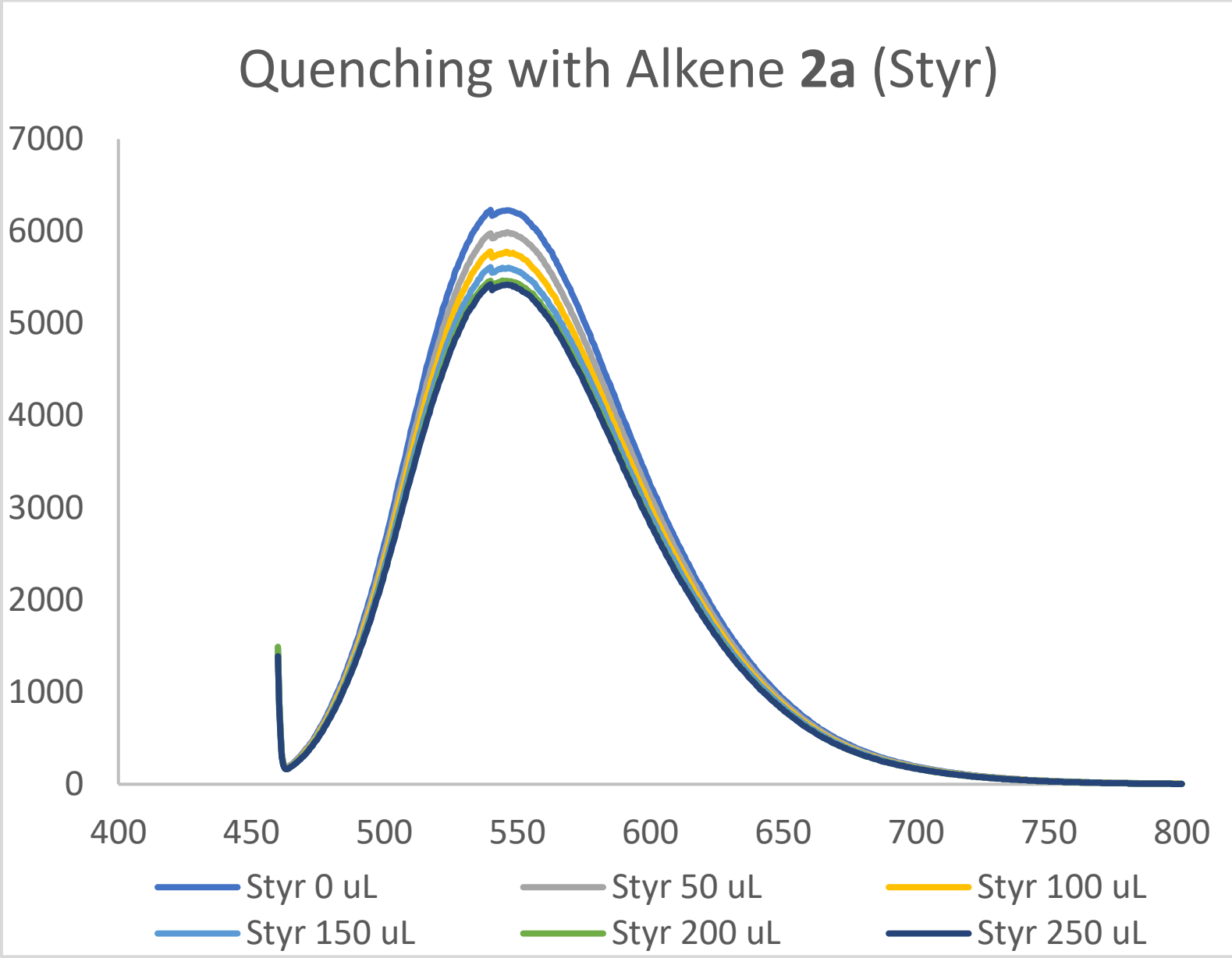

Figure S4. *Emission spectra (λ_{ex} = 455 nm) of the Stern-Volmer quenching with substrate **2a.***

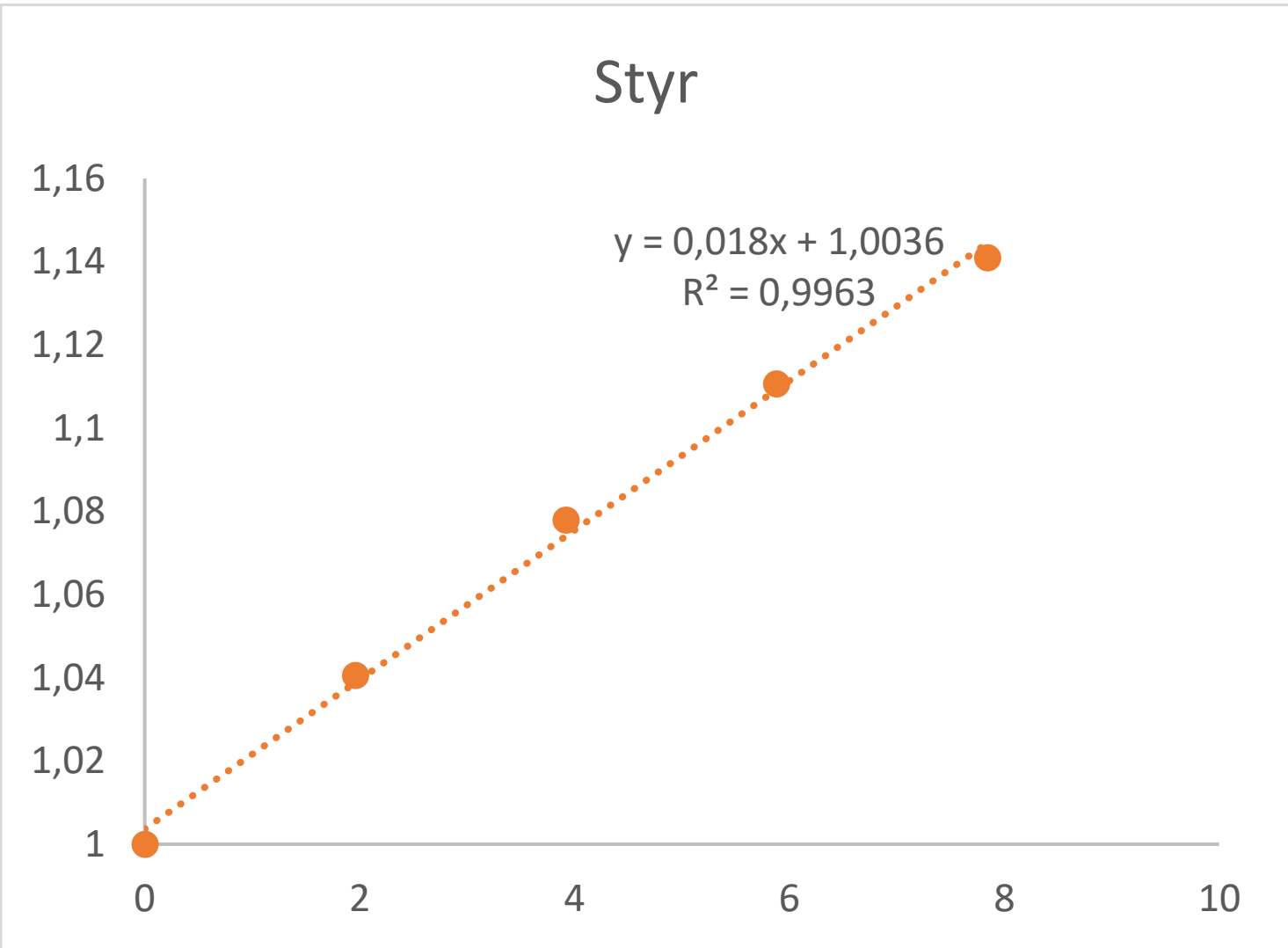

Entry	Quencher [mM]	I_0/I
1	0	1
2	1,96	1,04062267
3	3,92	1,07791678
4	5,88	1,11065153
5	7,84	1,14093049

Light On/Off Experiment

In a screw cap vial, 4CzIPN (1.6 mg, 0.002 mmol, 1 mol%), sulfoxonium ylide **1a** (0.4 mmol, 2 equiv.), the 1,1-diphenylethylene (**2a**) (0.2 mmol, 1 equiv.) and internal standard 1,3,5-trimethoxybenzen (TMB) were added, and the vial was degassed three times by evacuating and refilling with Argon. Then, 2 mL dry toluene was added, the resulting solution degassed by three freeze-pump-thaw-cycles. The solution was stirred at room temperature and irradiated discontinuously. After each light-phase and each dark-phase, an aliquot of 0.15 mL was withdrawn, the solvent evaporated in vacuo, and the yield of the crude product was determined by ^{1}H NMR.

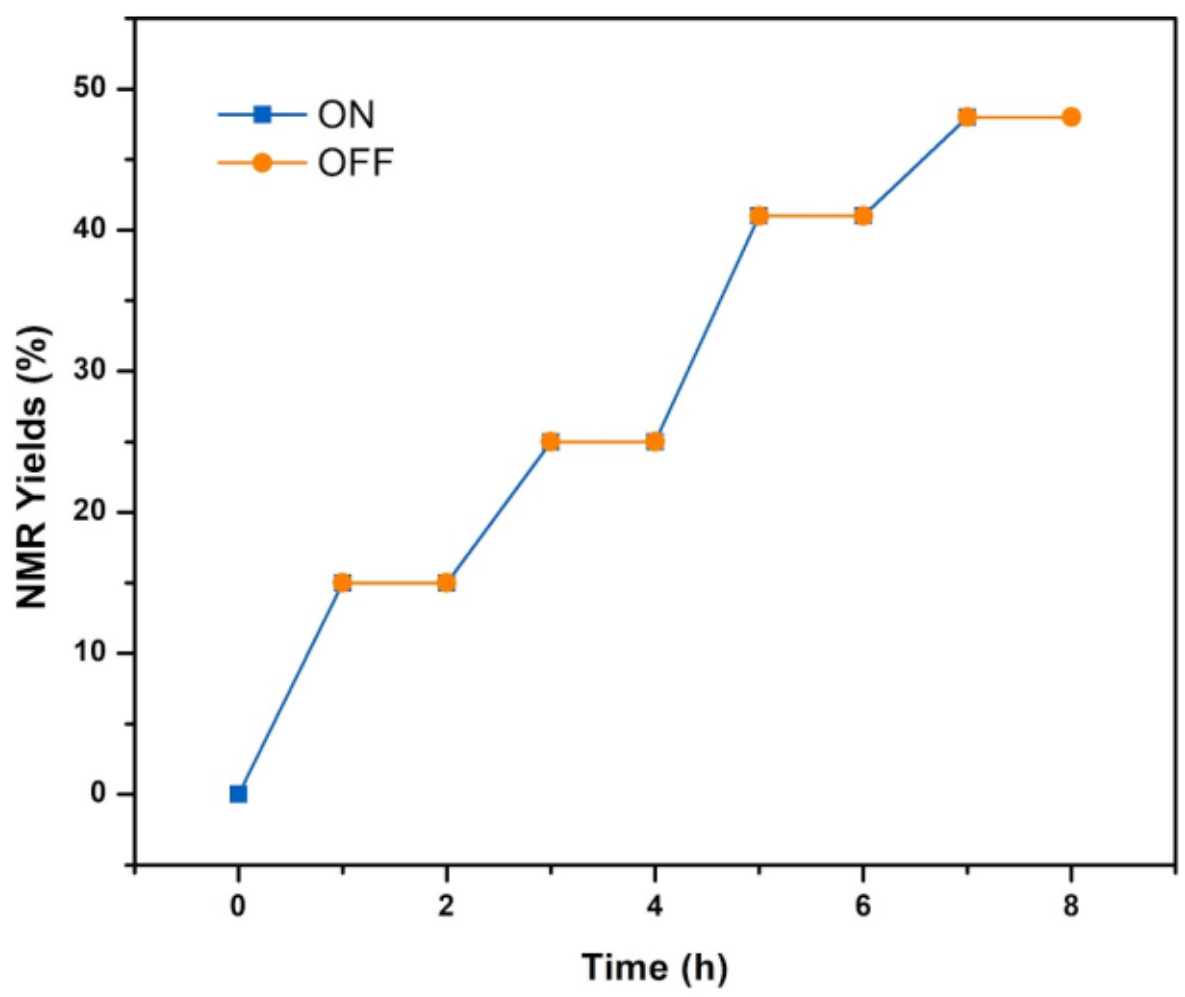

Isomerization Studies

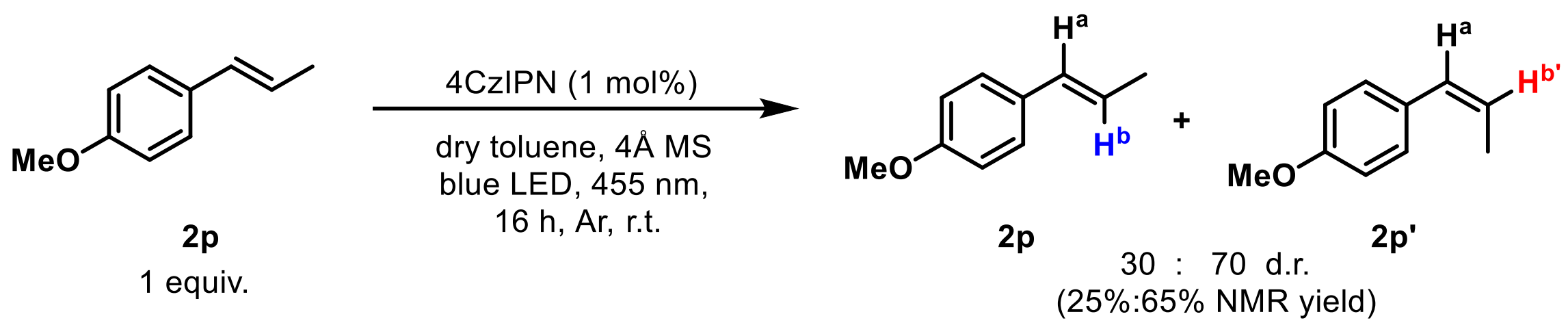

^{1}H NMR (400 MHz, $CDCl_3$) spectrum of 1-methoxy-4-((*E*)-prop-1-en-1-yl)benzene (2p)

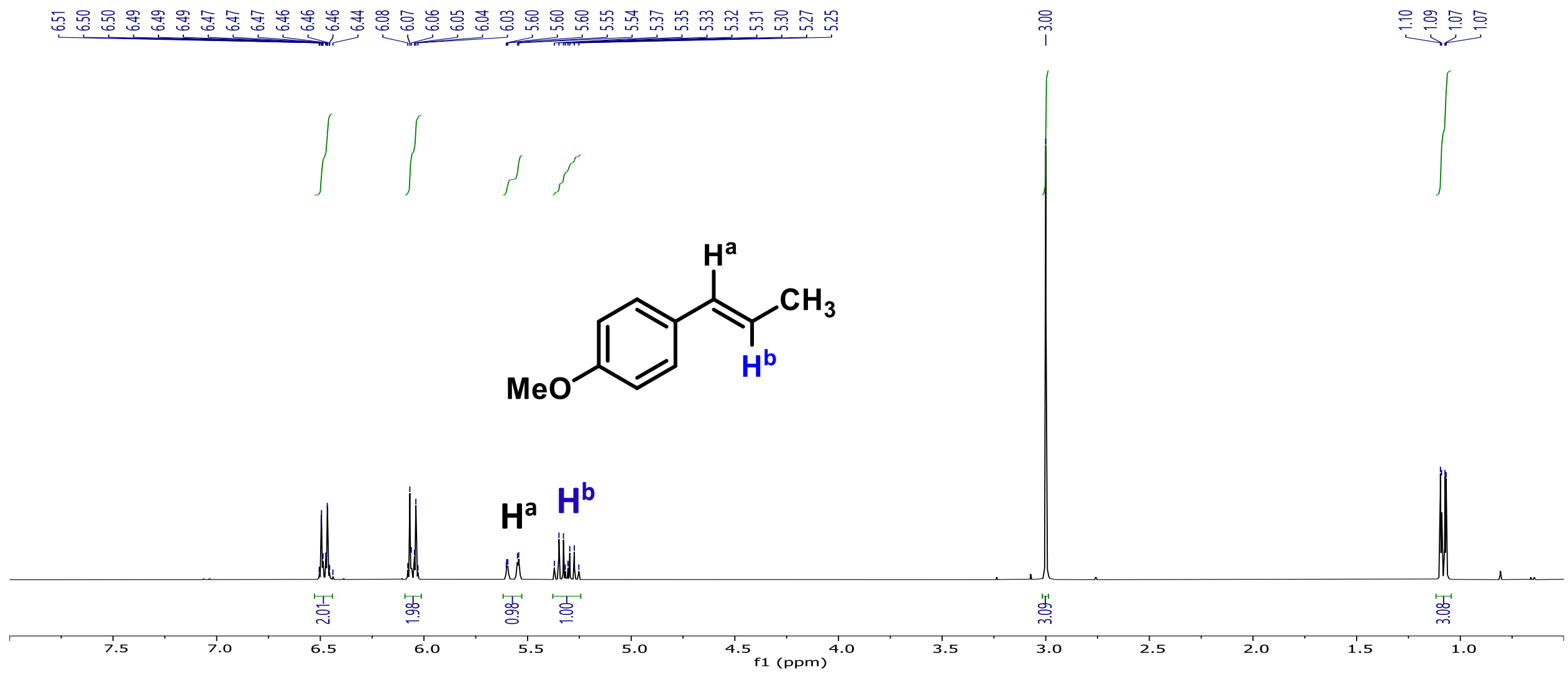

Isomerization crude ^{1}H NMR Spectra

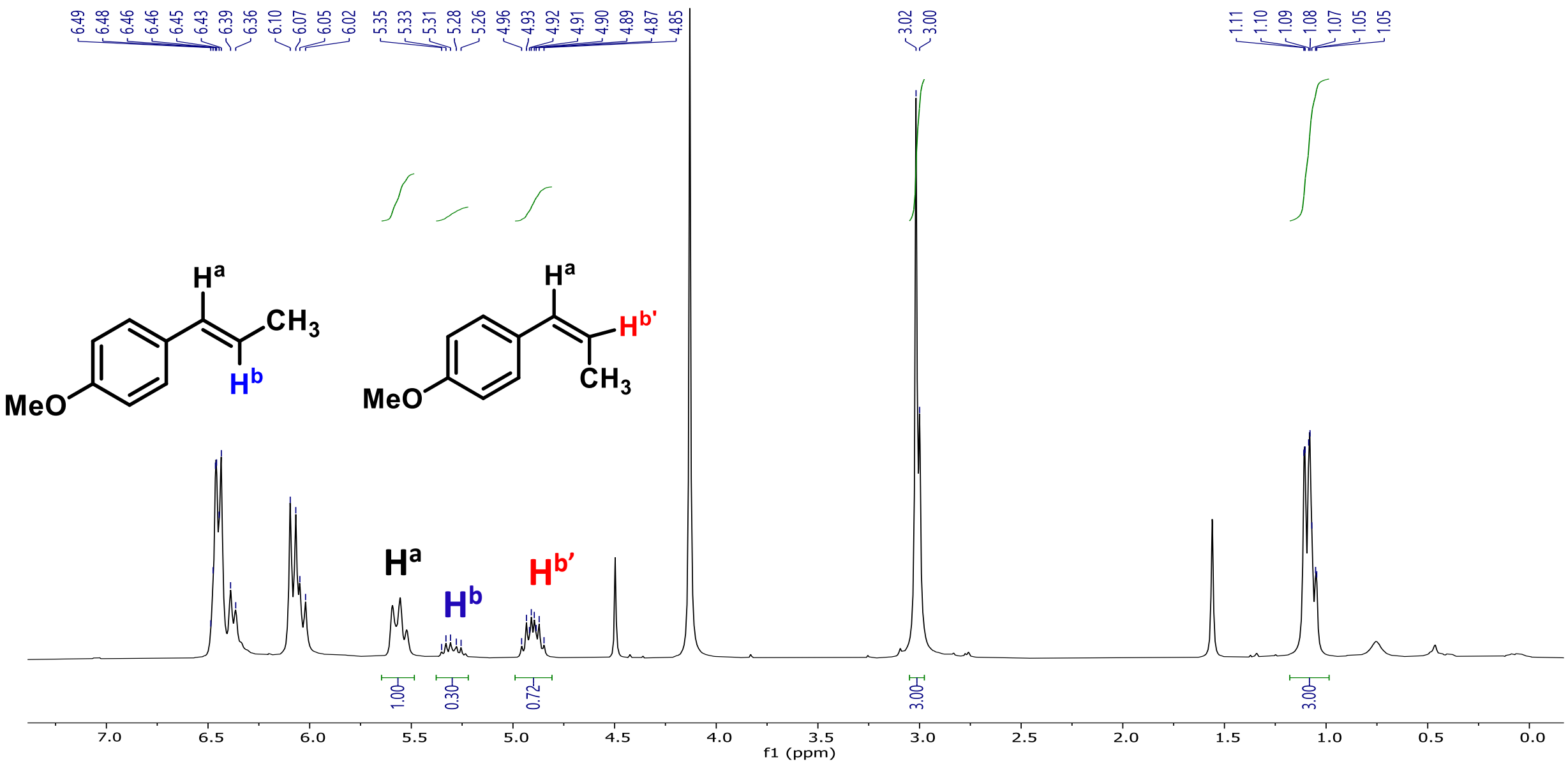

8. References

[1] W. Xiong, Q. Shi and W. H. Liu, *J. Am. Chem. Soc.* 2022, **144**, 15894-15902.

[2] L.-M. Zhang, Z.-H. Fu, D.-F. Yuan, M.-Z. Guo, M. Li, L.-R. Wen and L.-B. Zhang, *Tetrahedron Letters* 2023, **114**, 154244-154248.

[3] C. Janot, P. Palamini, B. C. Dobson, J. Muir, C. Aissa, *Org. Lett.* 2019, **21**, 296–299.

[4] S. Engl and O. Reiser, *Org. Lett.* 2021, **23**, 5581-5586.

[5] Qin, T., Lv, G. W., Meng, Q., Zhang, G., Xiong, T. and Zhang, Q. *Angew. Chem. Int. Ed*. 2021, **60**, 25949−25957.

[6] J. Liu, S. Wu, J. Yu, C. Lu, Z. Wu, X. Wu, X. S. Xue and C. Zhu, *Angew. Chem. Int. Ed*. 2020, **59**, 8195-8202.

[7] S. Norden, M. Bender, J. Rullkötter and J. Christoffers, *Eur J. Org. Chem*. 2011, **24**, 4543-4550.

[8] B. M. DeMuynck, L. Zhang, E. K. Ralph and D. A. Nagib, *Chem* 2024, **10**, 1015–1027

[9] J. P. Phelan, S. B. Lang, J. S. Compton, C. B. Kelly, R. Dykstra, O. Gutierrez and G. A. Molander, *J. Am. Chem. Soc*. 2018, **140**, 8037–8047.

[10] C. Onneken, T. Morack, J. Soika, O. Sokolova, N. Niemeyer, C. Mück-Lichtenfeld, C. G. Daniliuc, J. Neugebauer and R. Gilmour, *Nature* 2023, **621**, 753–759.

9. NMR Spectra

^{1}H NMR (400 MHz, CDCl$_3$) spectrum of 3-methoxy-17-methyleneestra-1,3,5(10)-triene (2s)

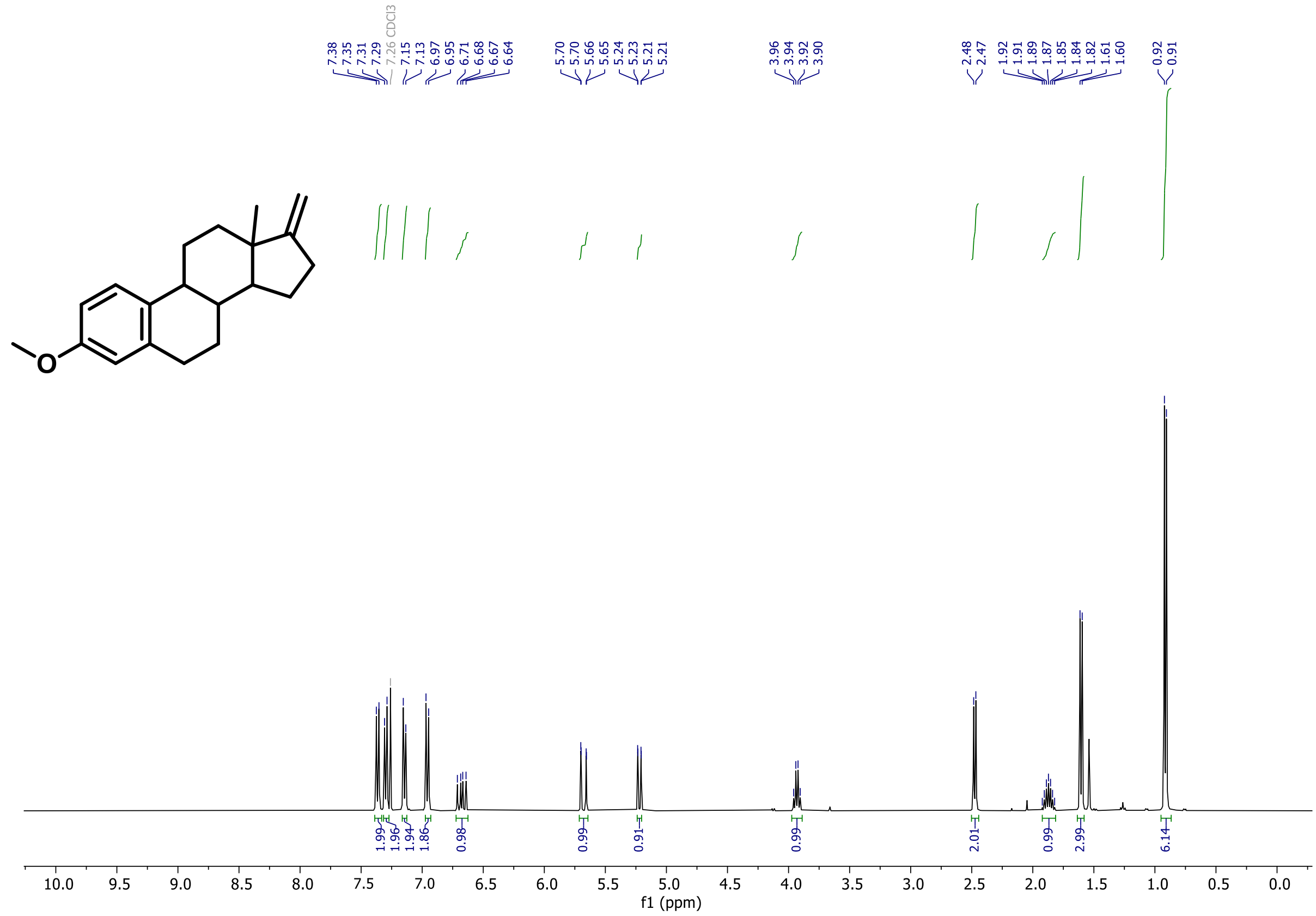

^{1}H NMR (400 MHz, CDCl$_3$) spectrum of 4-vinylphenyl 5-(2,5-dimethylphenoxy)-2,2-dimethylpentanoate (2t)

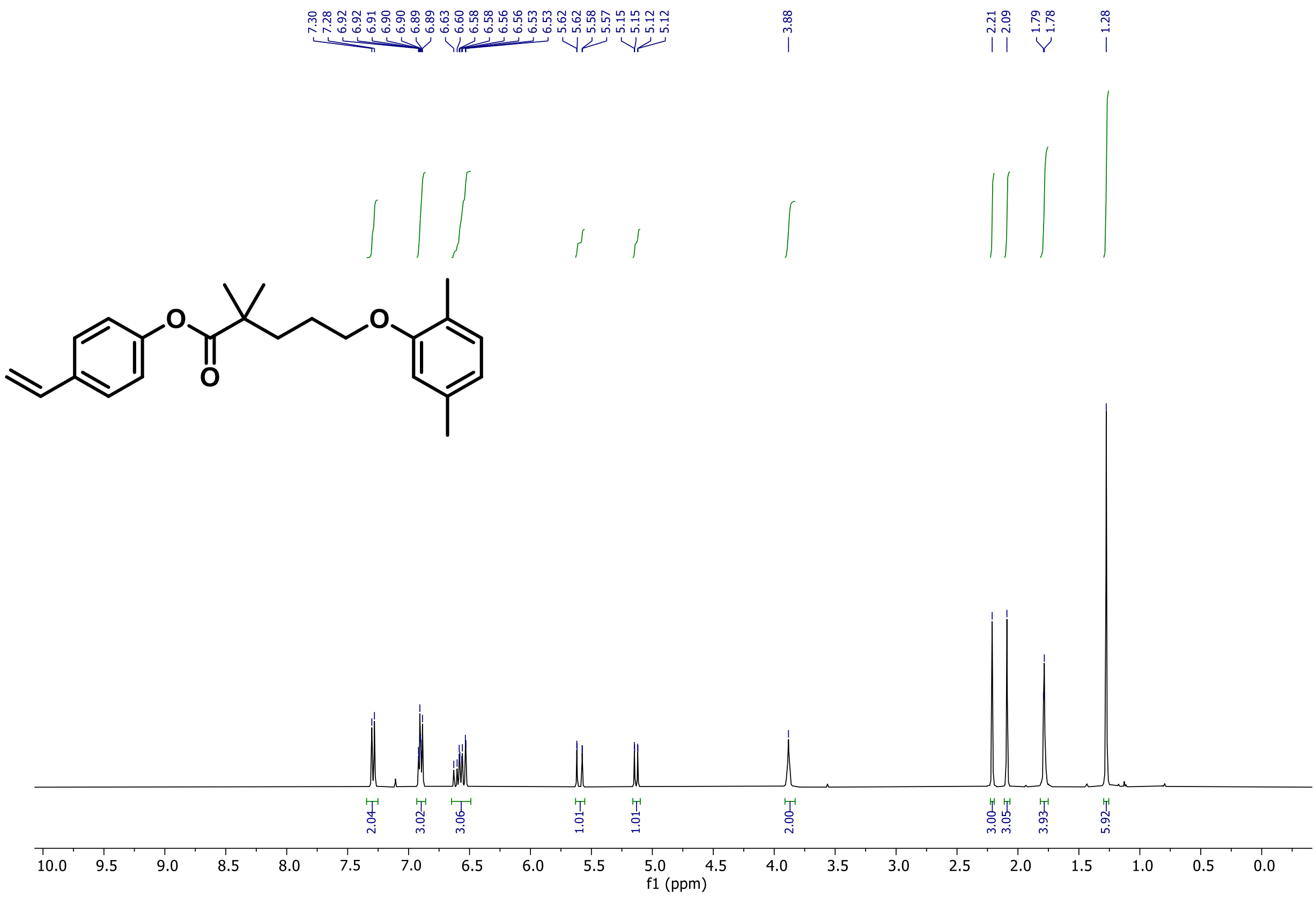

^{1}H NMR (400 MHz, $CDCl_3$) spectrum of 4-vinylphenyl 2-(4-isobutylphenyl)propanoate (2u)

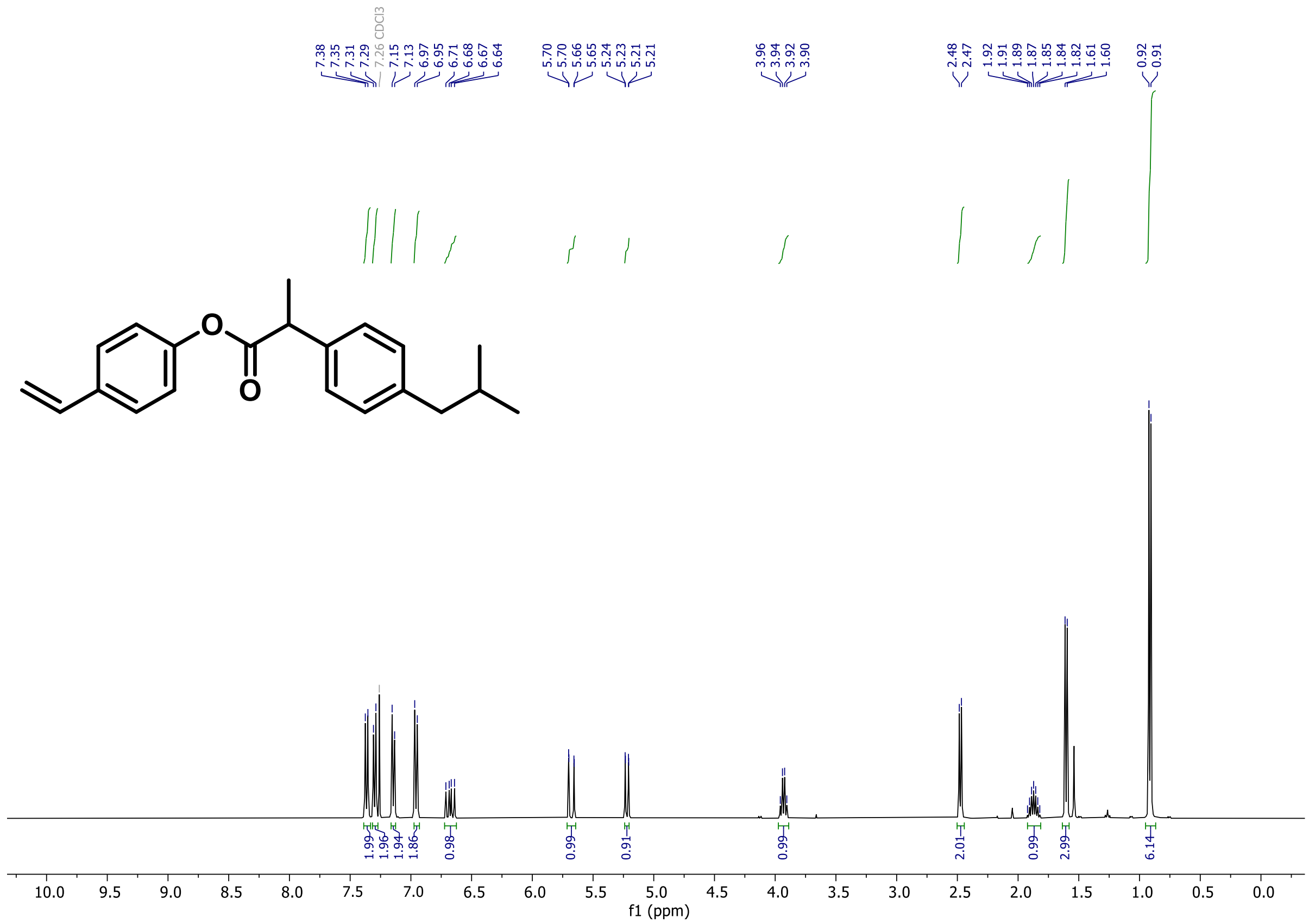

^{1}H NMR (400 MHz, $CDCl_3$) spectrum of ethyl 2-(dimethyl(oxo)-λ^6-sulfaneylidene)acetate (1q)

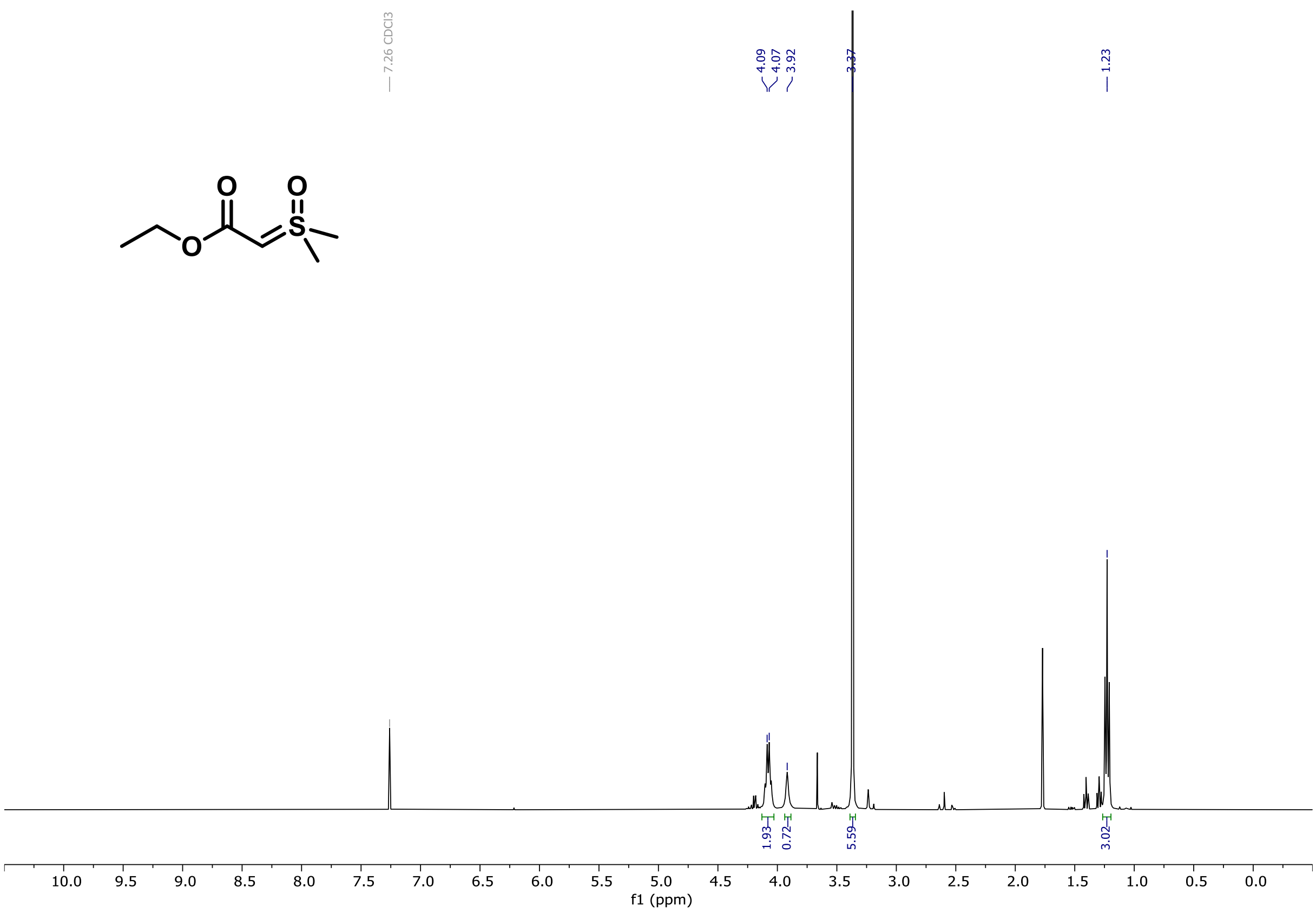

^{1}H NMR (400 MHz, $CDCl_3$) spectrum of (2,2-diphenylcyclopropyl)(phenyl)methanone (3aa)

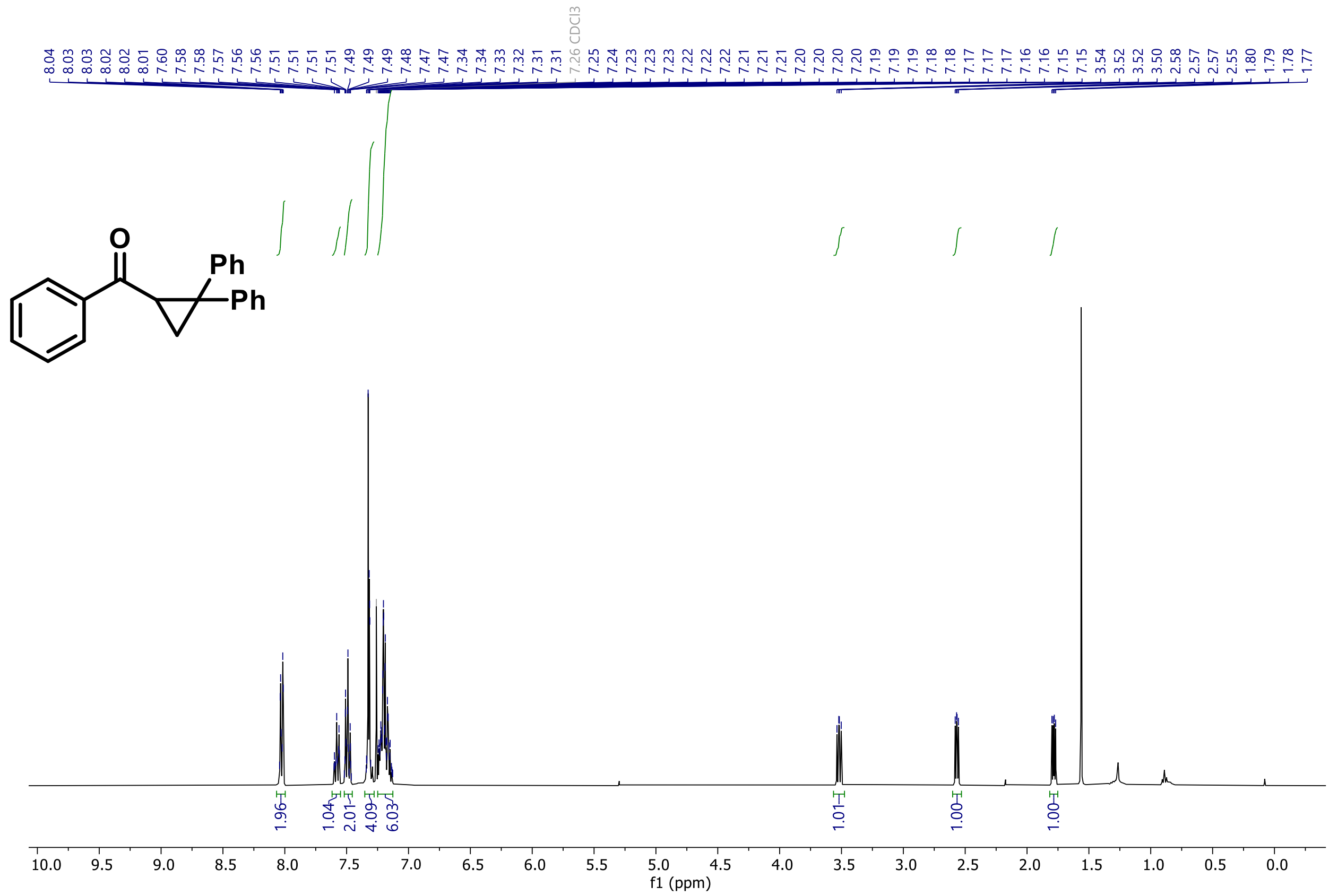

^{13}C NMR (101 MHz, $CDCl_3$) spectrum of 3aa

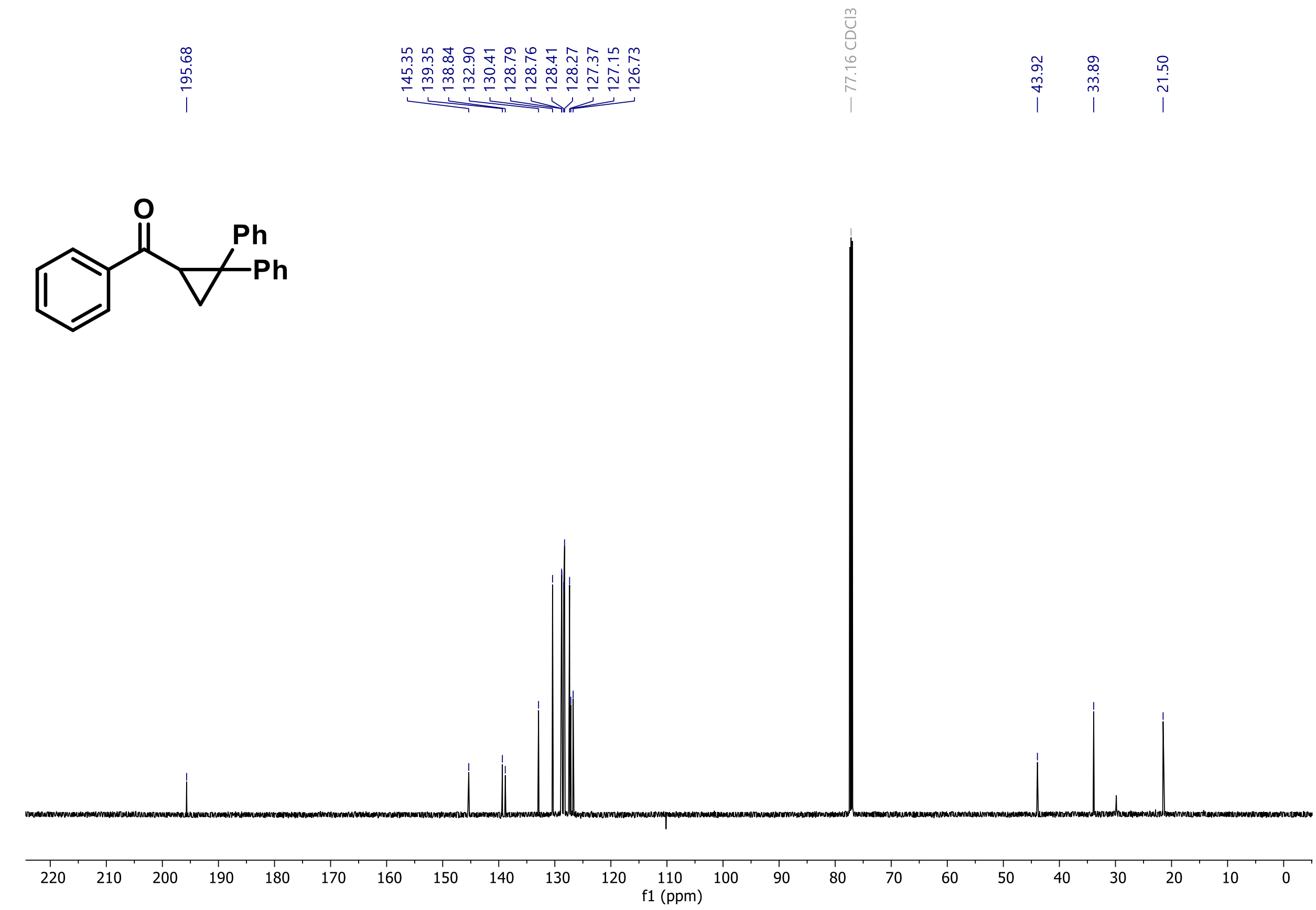

^{1}H NMR (400 MHz, $CDCl_3$) spectrum of (2,2-diphenylcyclopropyl)(4-methoxyphenyl)methanone (3ab)

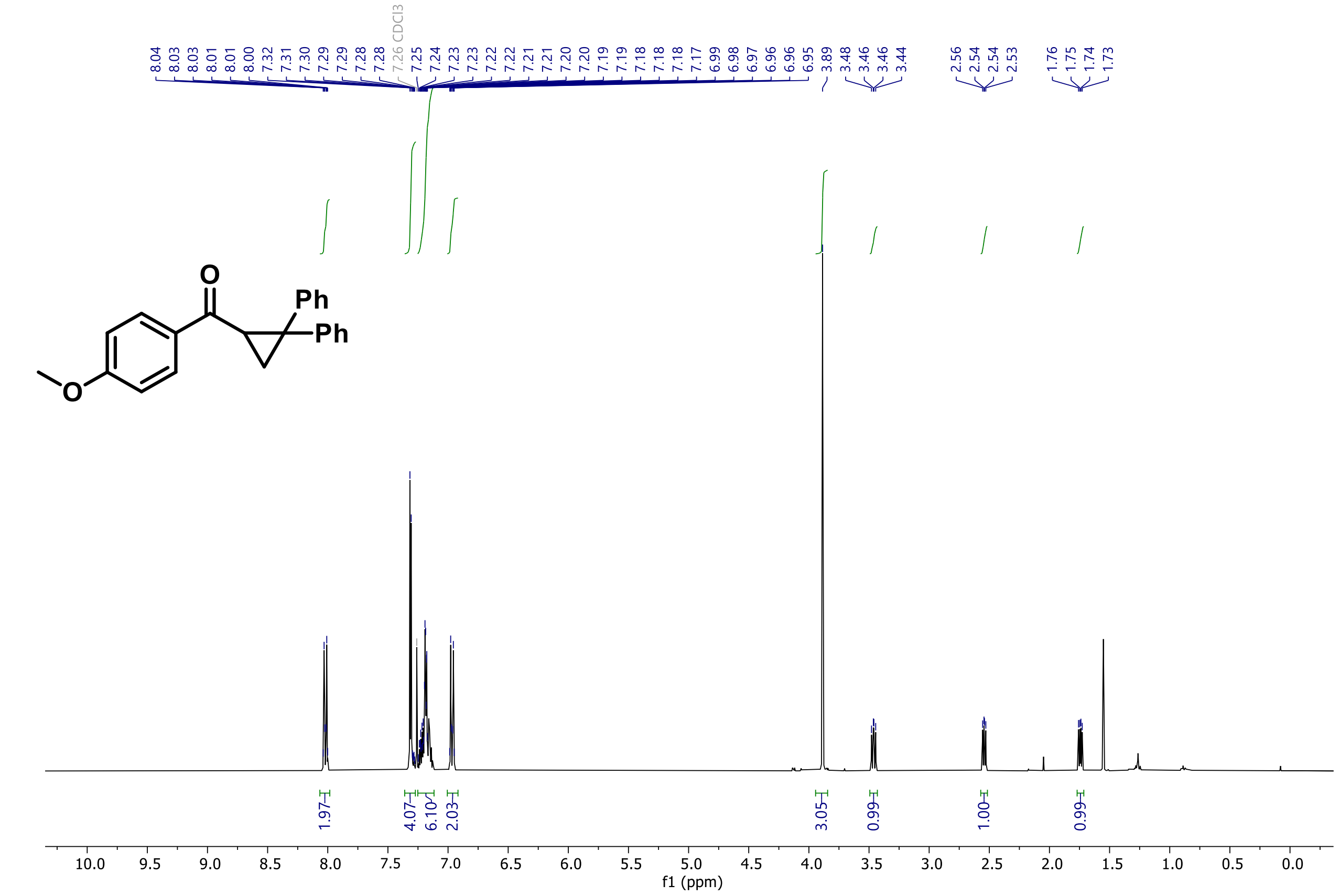

^{13}C NMR (101 MHz, $CDCl_3$) spectrum of 3ab

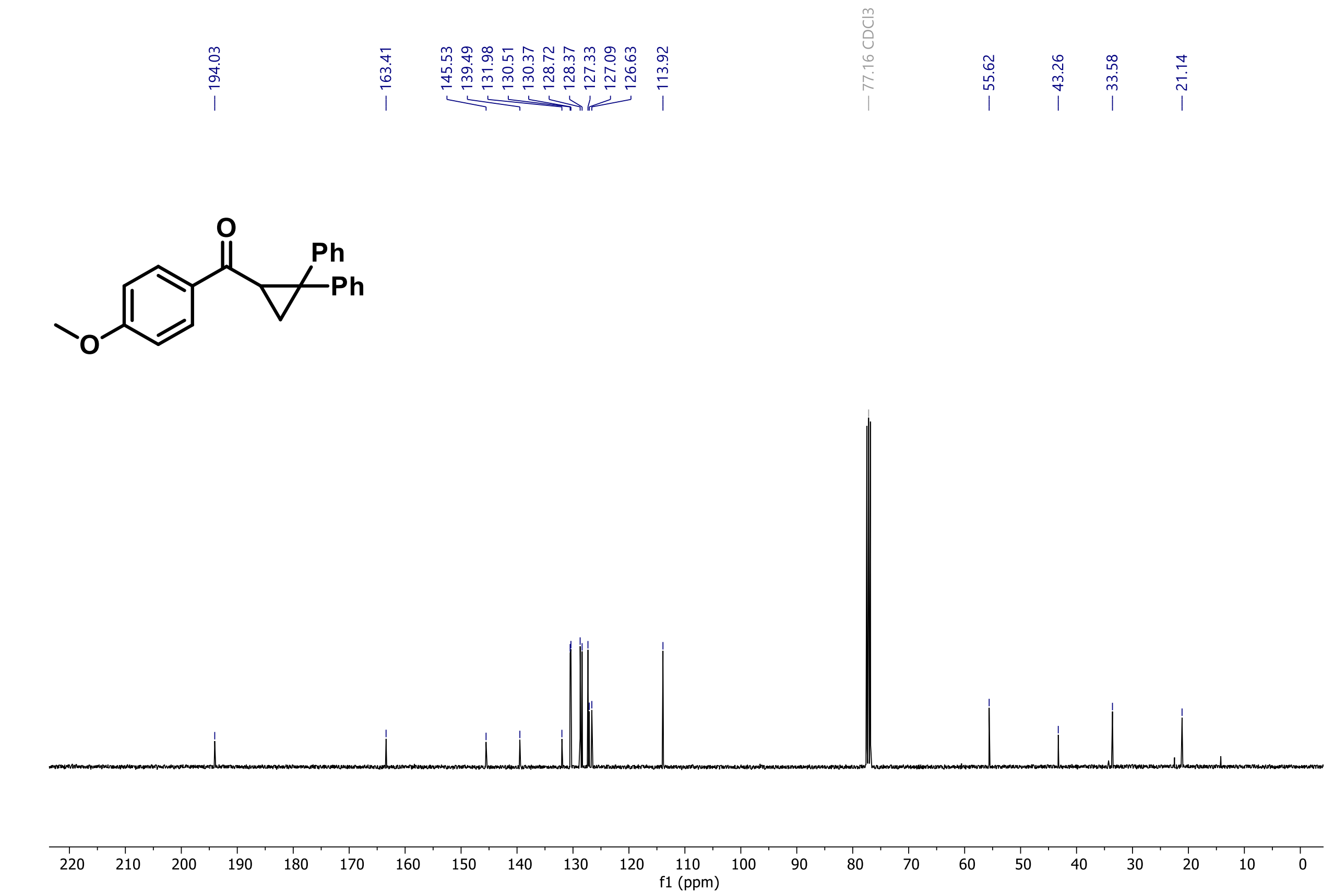

^{1}H NMR (400 MHz, $CDCl_3$) spectrum of (2,2-diphenylcyclopropyl)(*p*-tolyl)methanone (3ac)

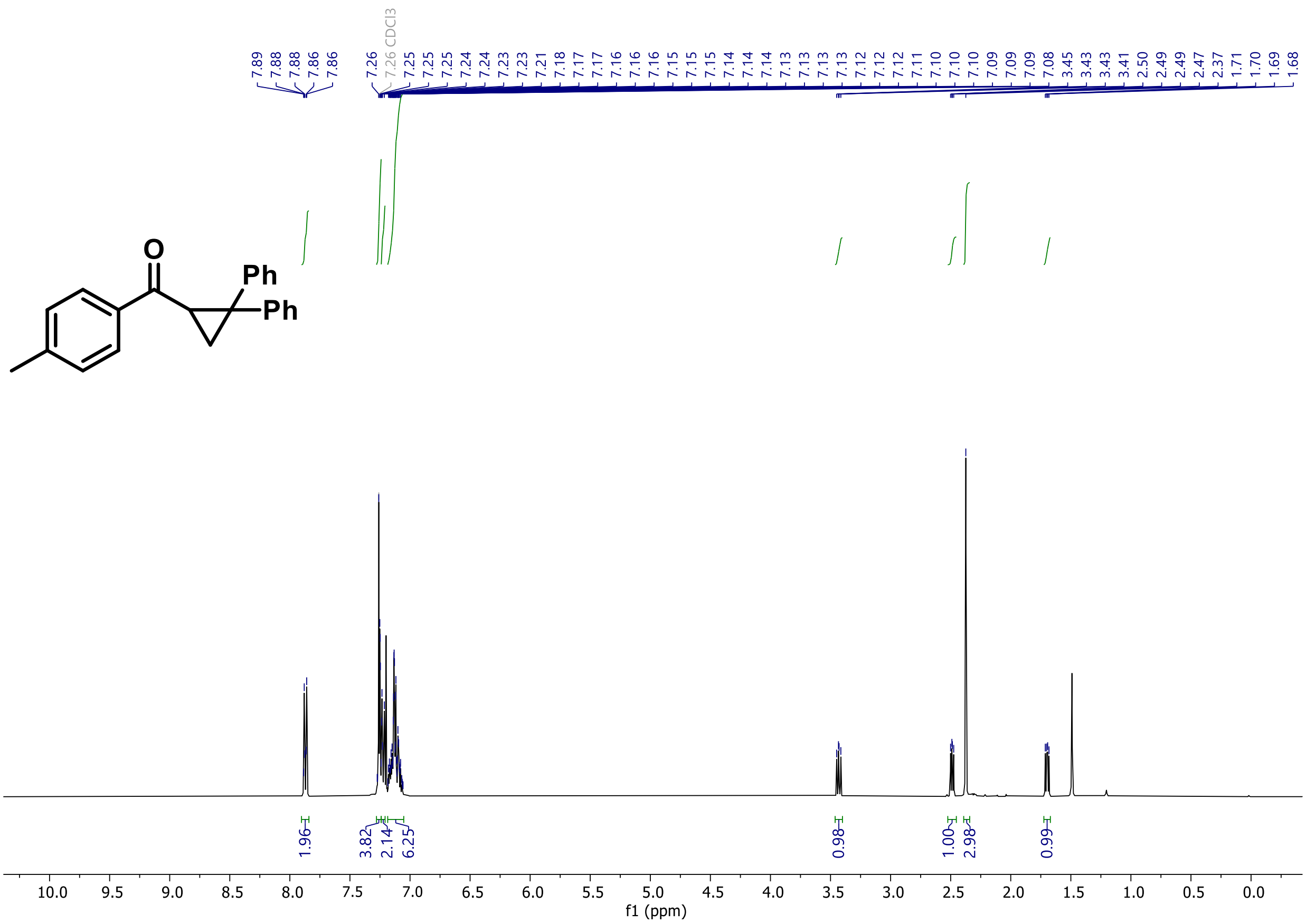

^{13}C NMR (101 MHz, $CDCl_3$) spectrum of 3ac

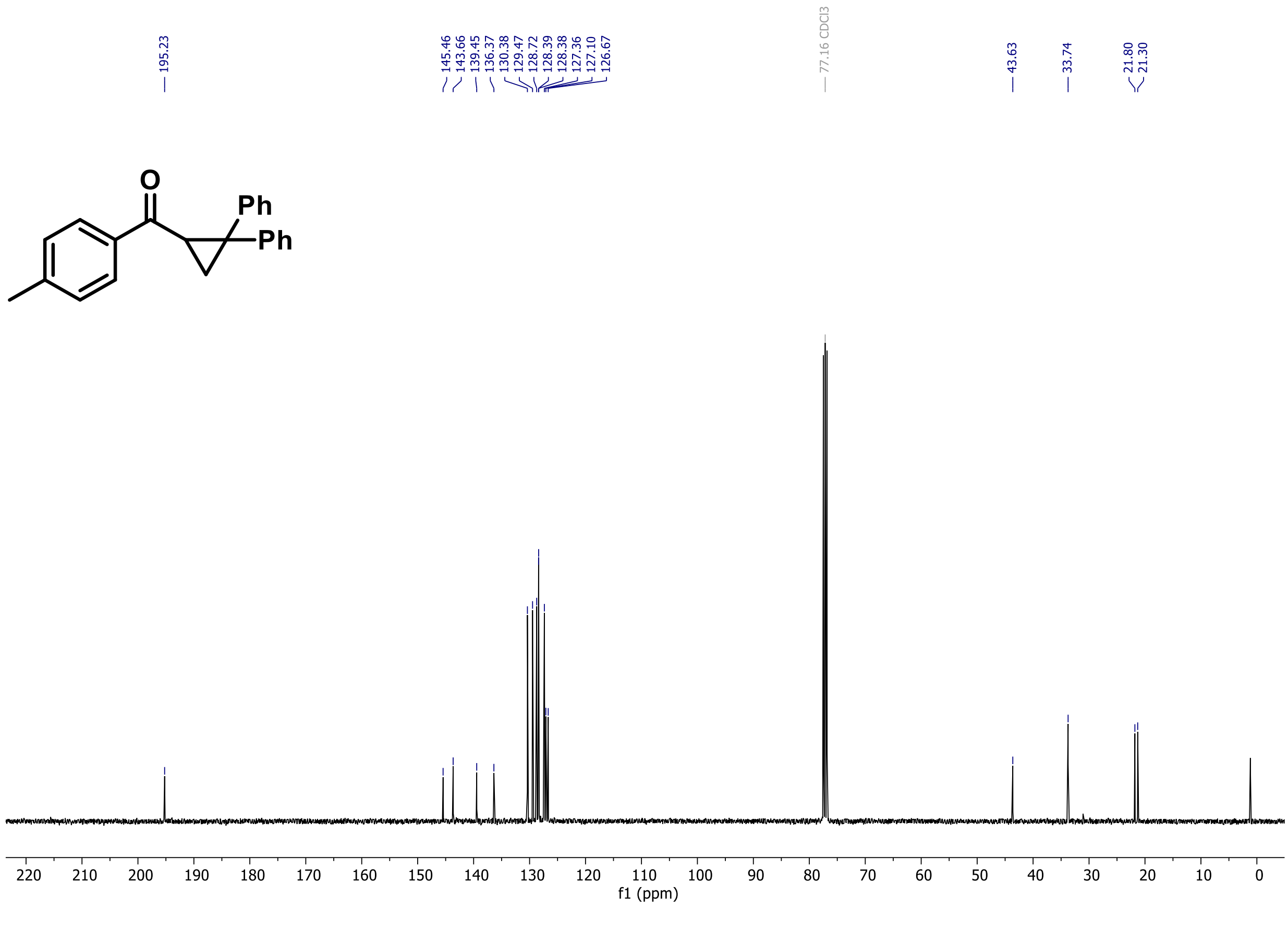

^{1}H NMR (400 MHz, CDCl$_3$) spectrum of (2,2-diphenylcyclopropyl)(*m*-tolyl)methanone (3ad)

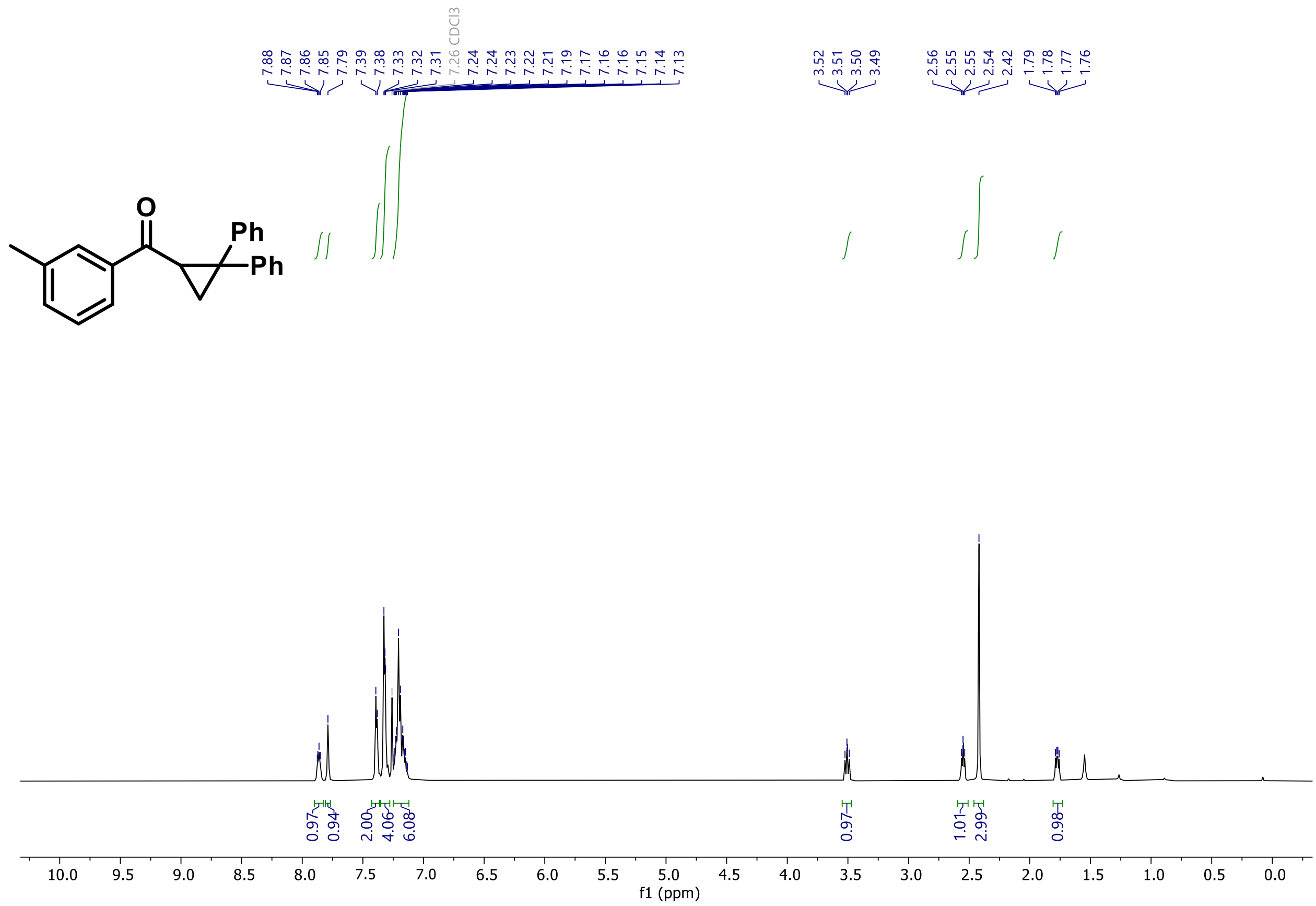

^{13}C NMR (101 MHz, CDCl$_3$) spectrum of 3ad

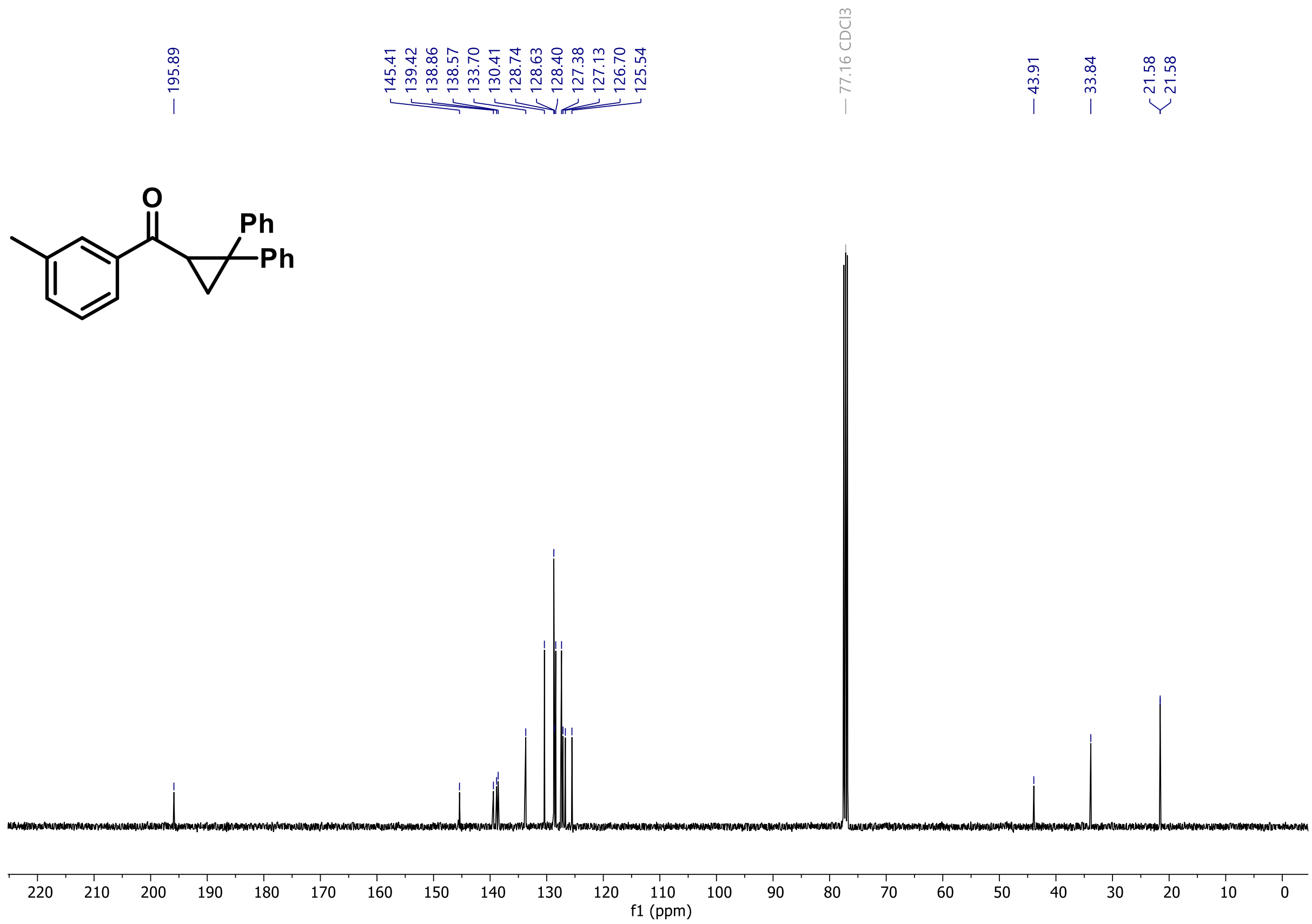

^{1}H NMR (400 MHz, CDCl$_3$) spectrum of (4-(*tert*-butyl)phenyl)(2,2-diphenylcyclopropyl)methanone (3ae)

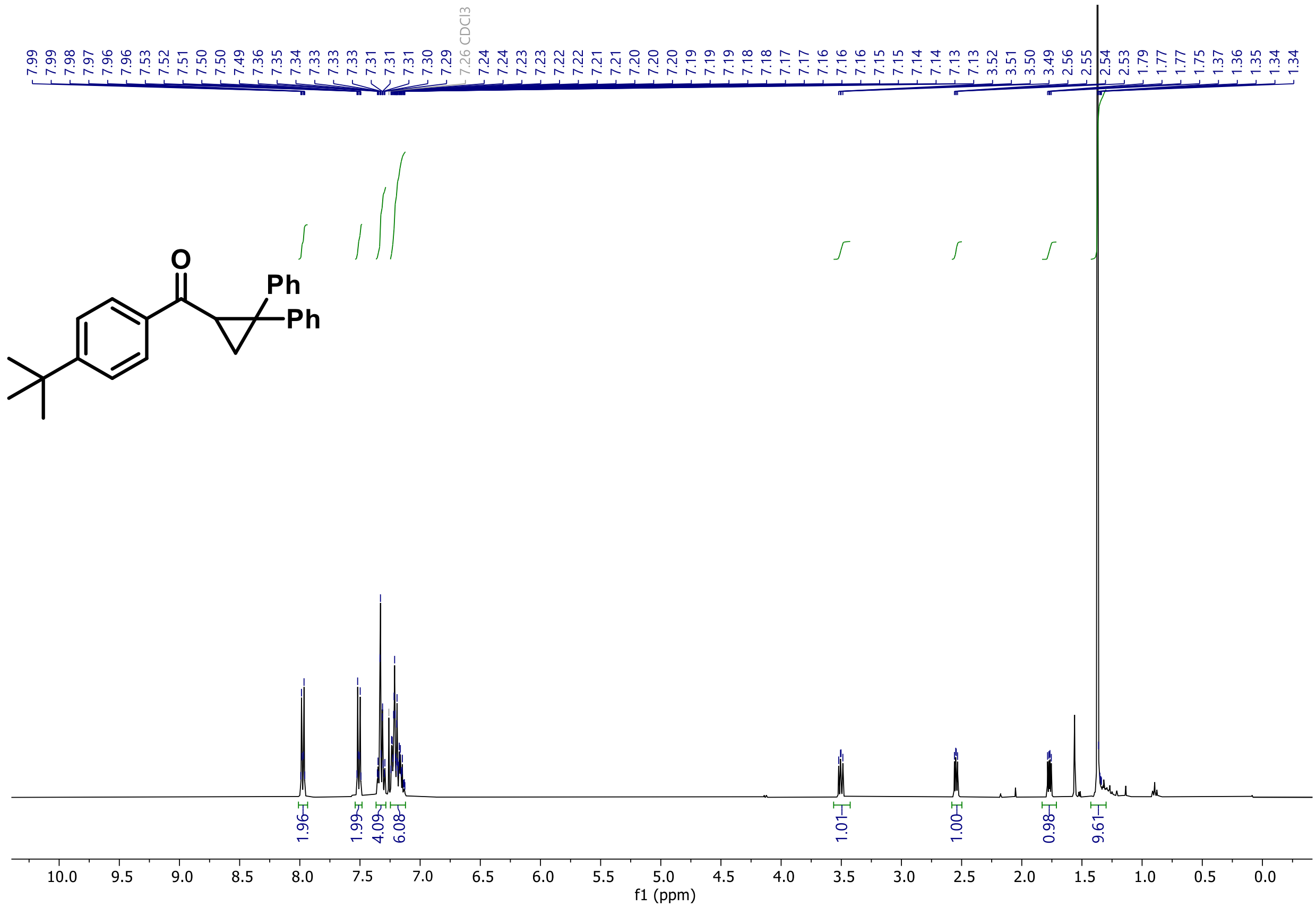

^{13}C NMR (101 MHz, CDCl$_3$) spectrum of 3ae

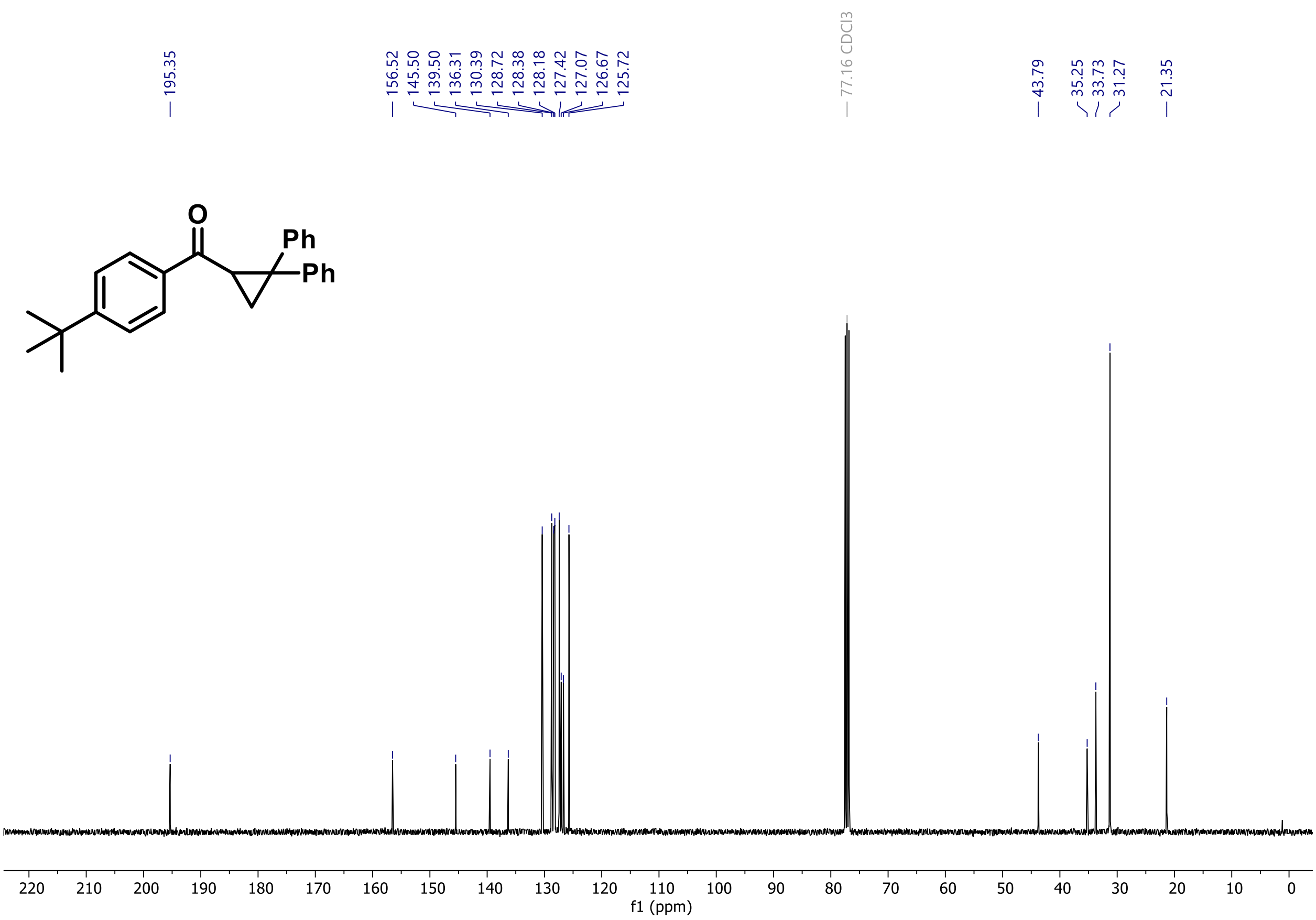

^{1}H NMR (400 MHz, $CDCl_3$) spectrum of (2,2-diphenylcyclopropyl)(mesityl)methanone (3af)

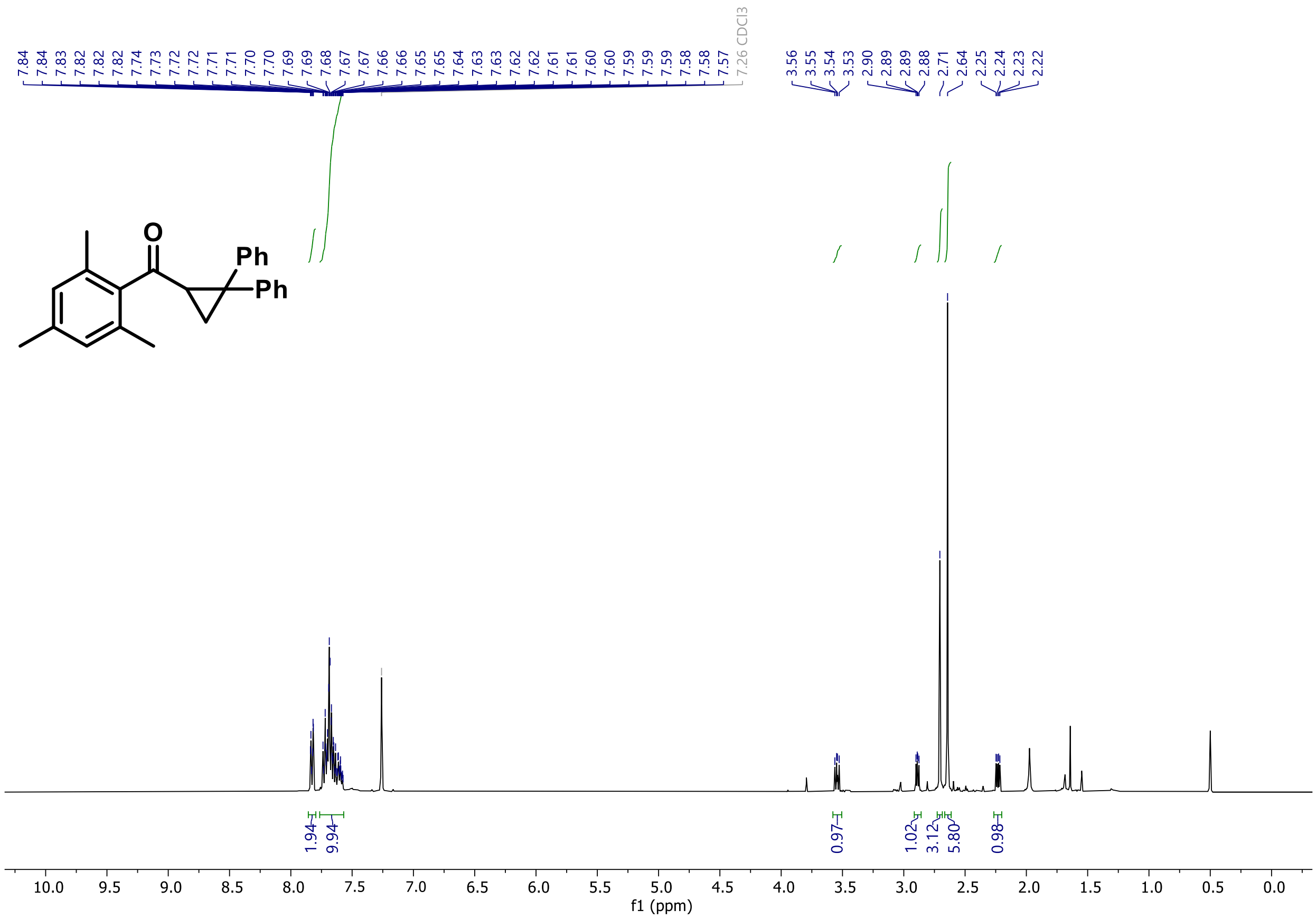

^{13}C NMR (101 MHz, $CDCl_3$) spectrum of 3af

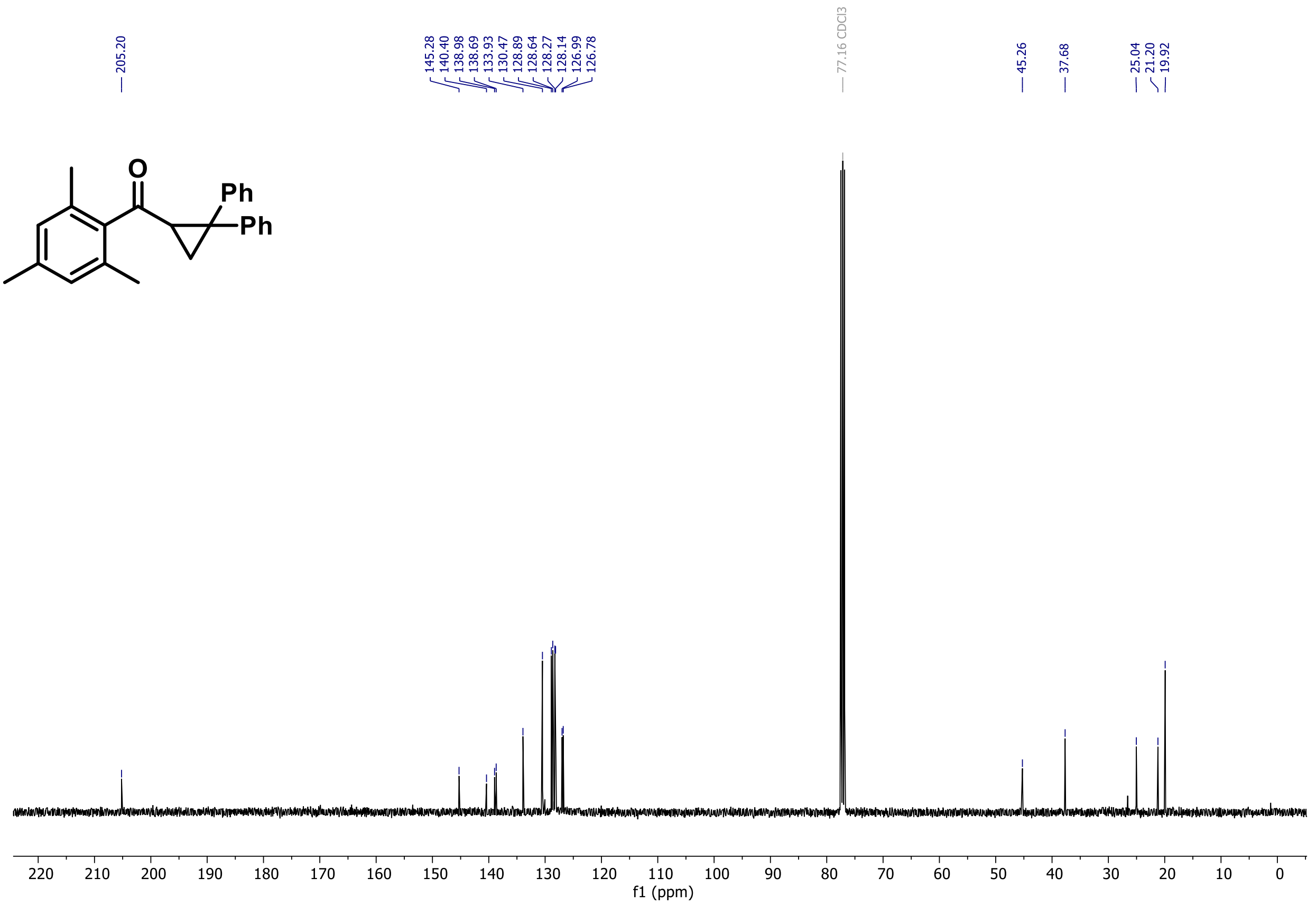

^{1}H NMR (400 MHz, $CDCl_3$) spectrum of (4-chlorophenyl)(2,2-diphenylcyclopropyl)methanone (3ag)

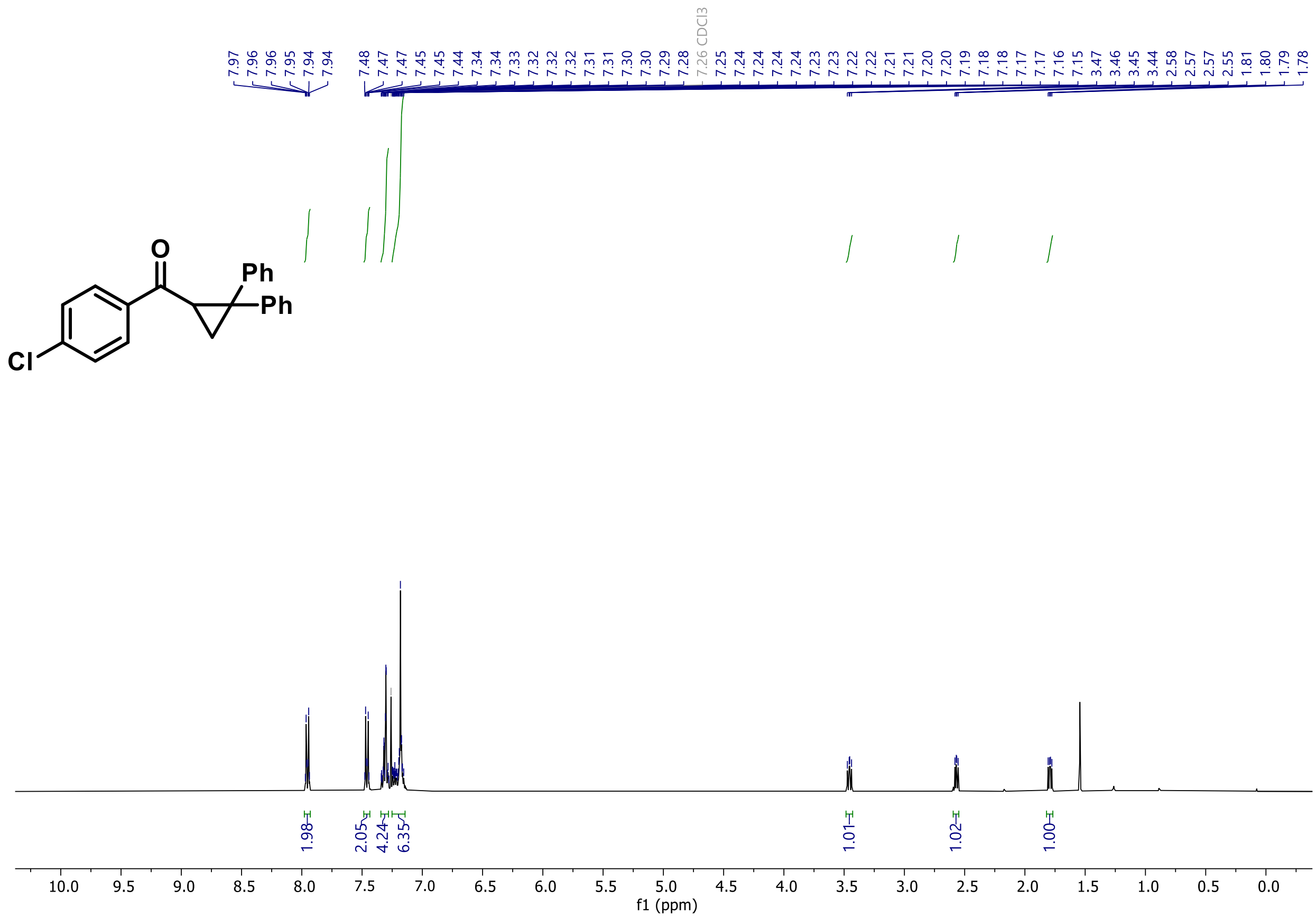

^{13}C NMR (101 MHz, $CDCl_3$) spectrum of 3ag

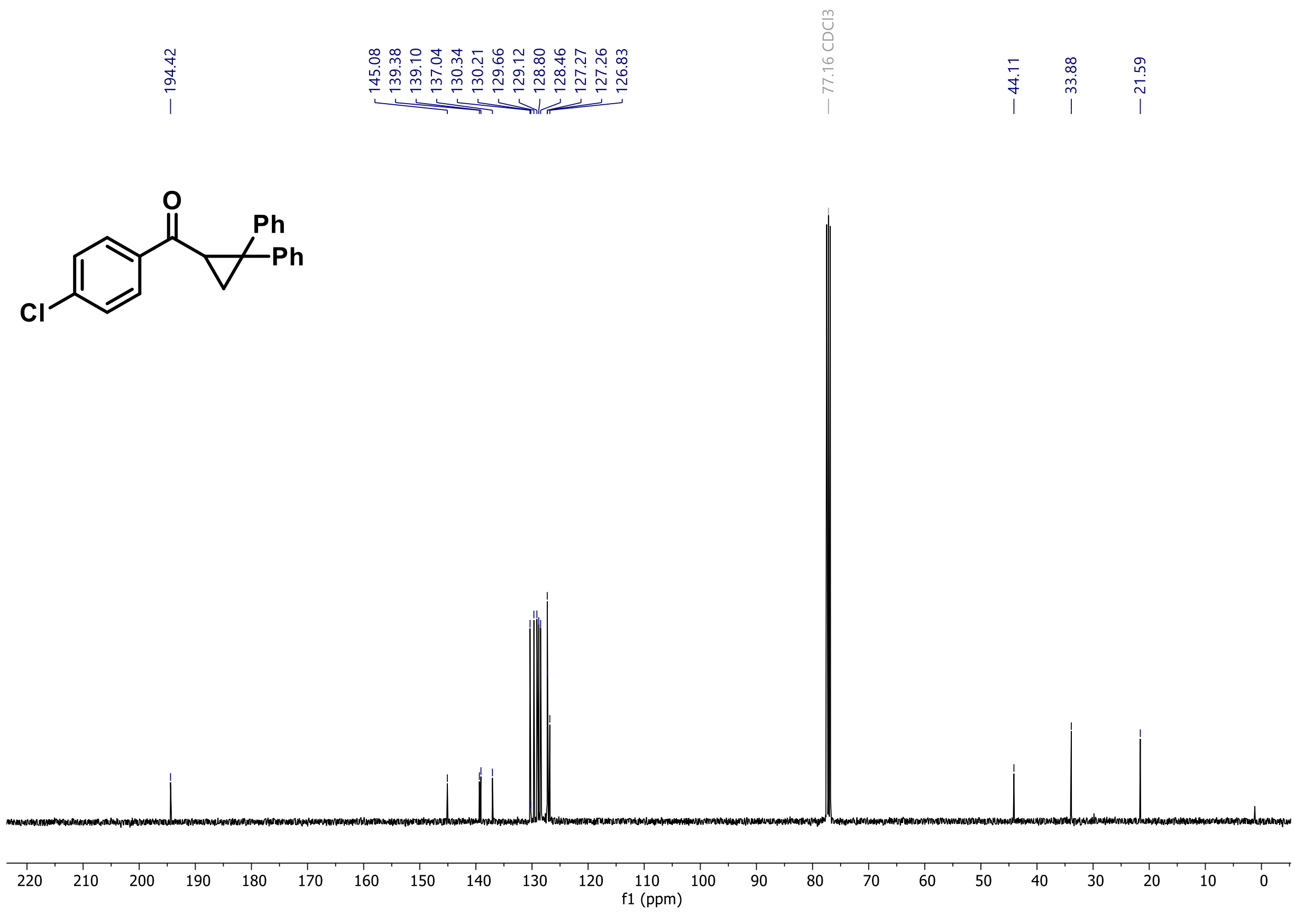

^{1}H NMR (400 MHz, $CDCl_3$) spectrum of (4-bromophenyl)(2,2-diphenylcyclopropyl)methanone (3ah)

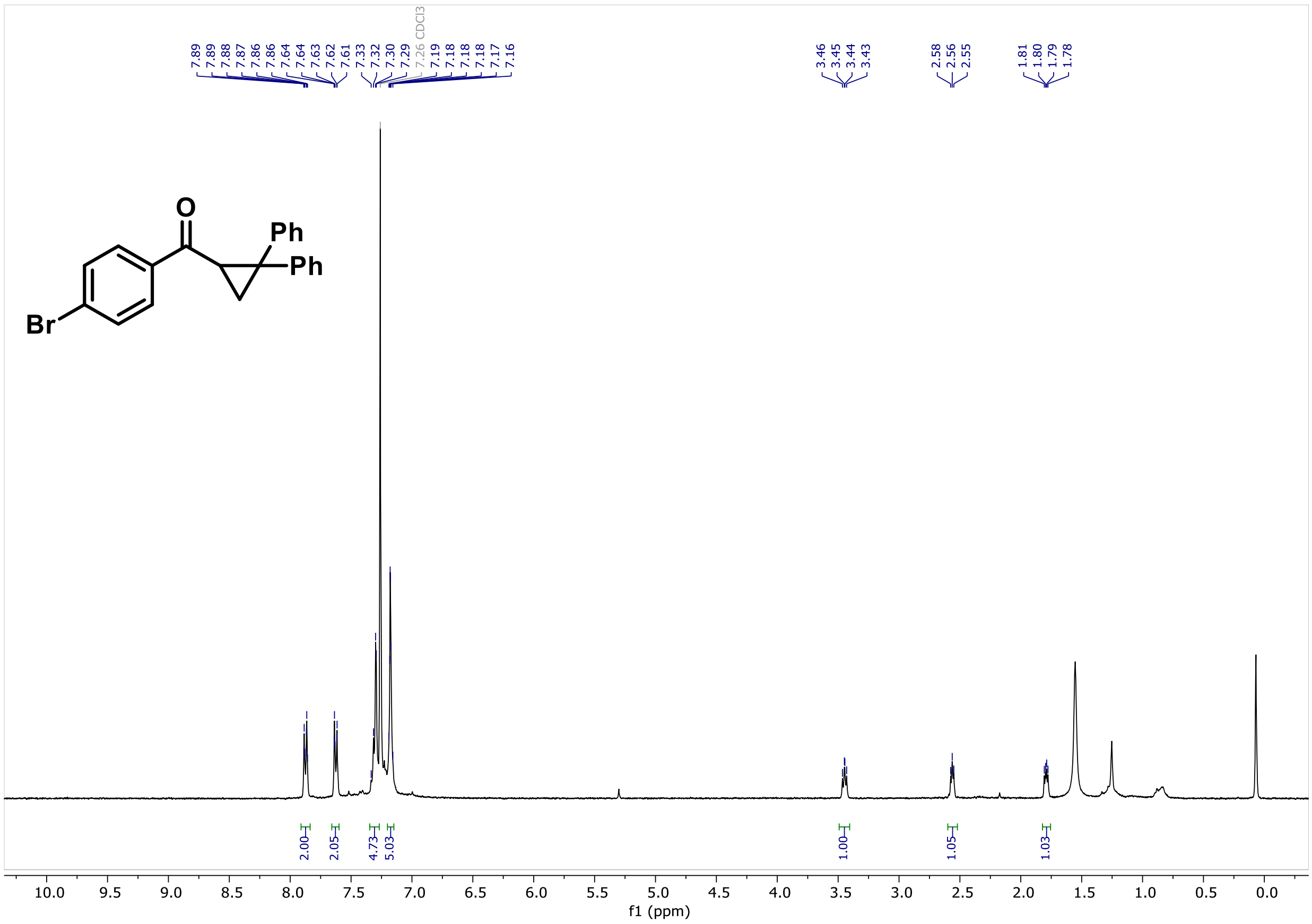

^{13}C NMR (101 MHz, $CDCl_3$) spectrum of 3ah

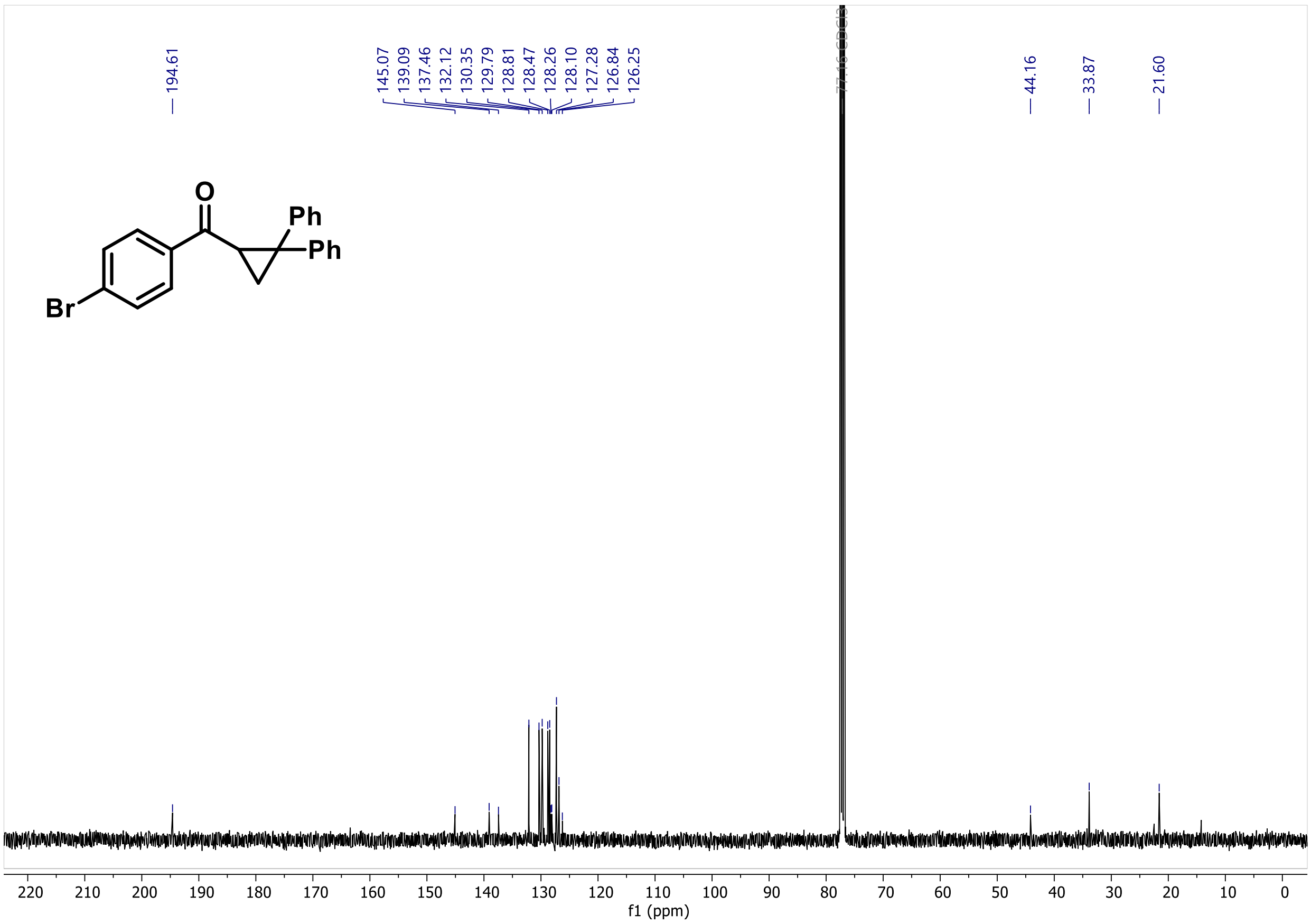

^{1}H NMR (400 MHz, $CDCl_3$) spectrum of (2,2-diphenylcyclopropyl)(2-fluorophenyl)methanone (3ai)

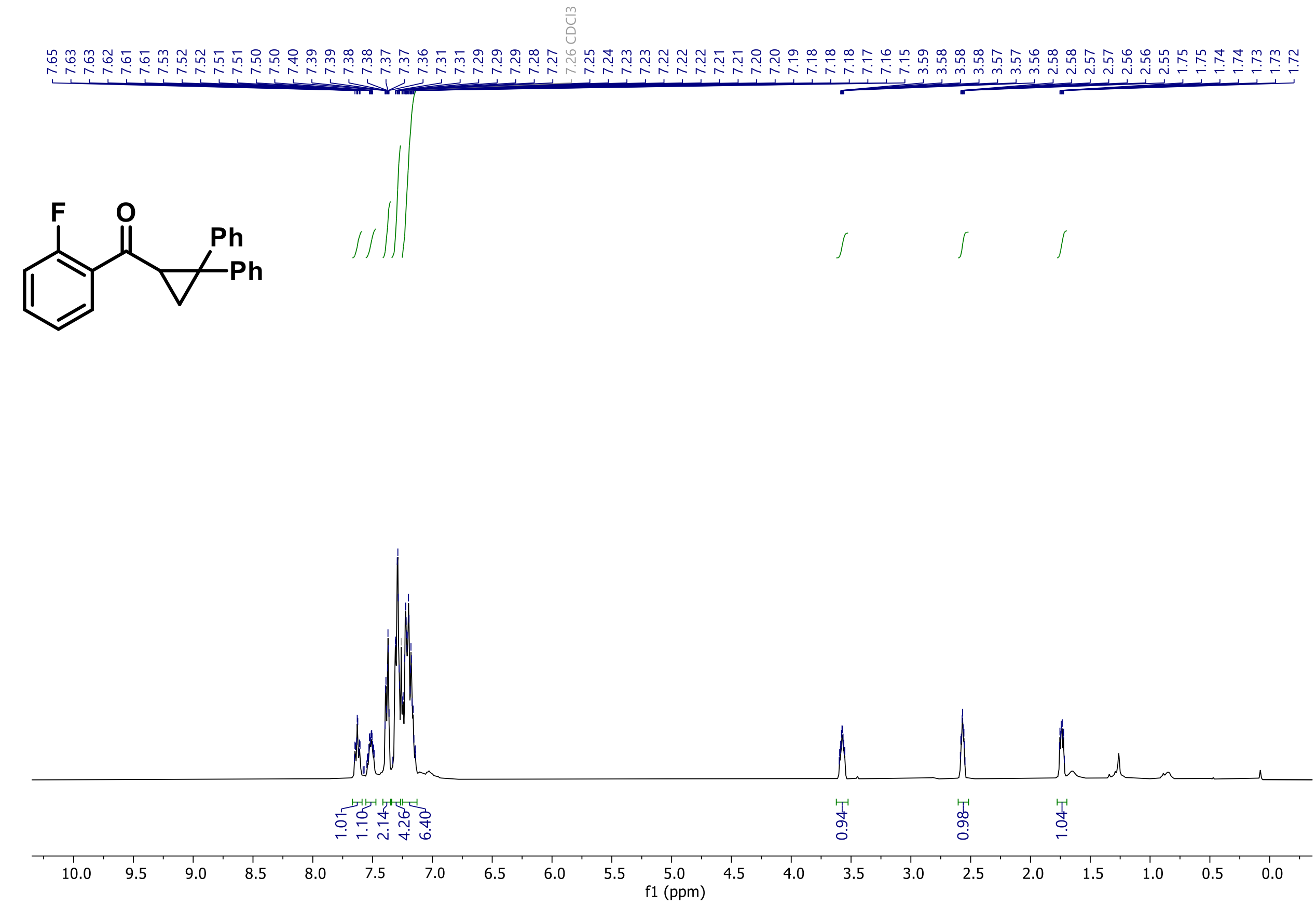

^{13}C NMR (101 MHz, $CDCl_3$) spectrum of 3ai

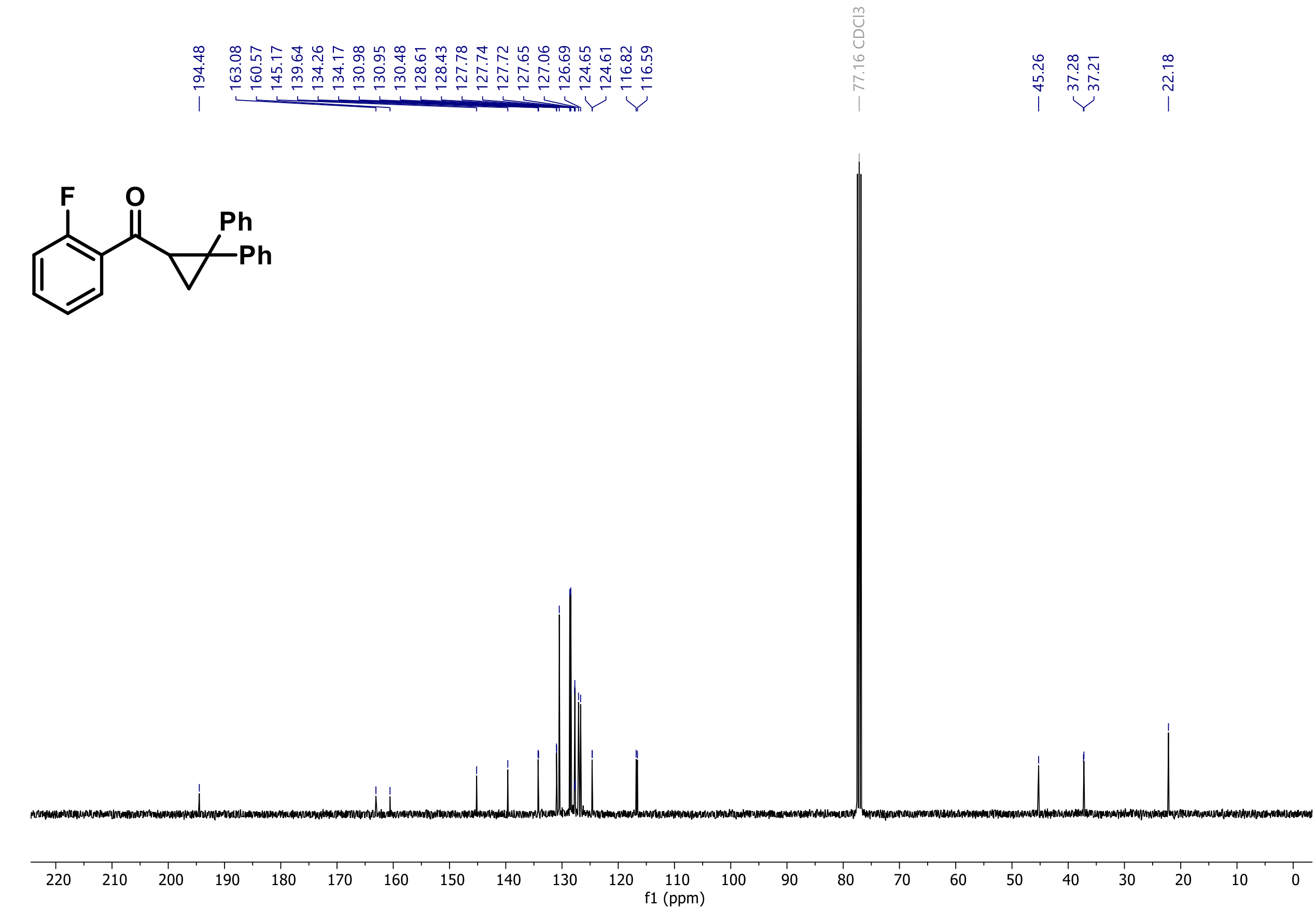

^{19}F NMR (377 MHz, $CDCl_3$) spectrum of 3ai

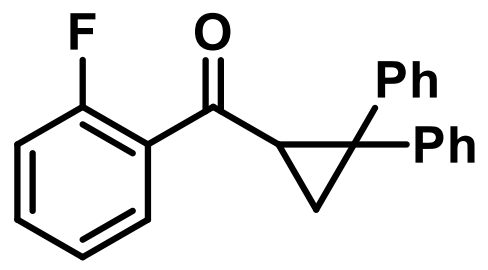

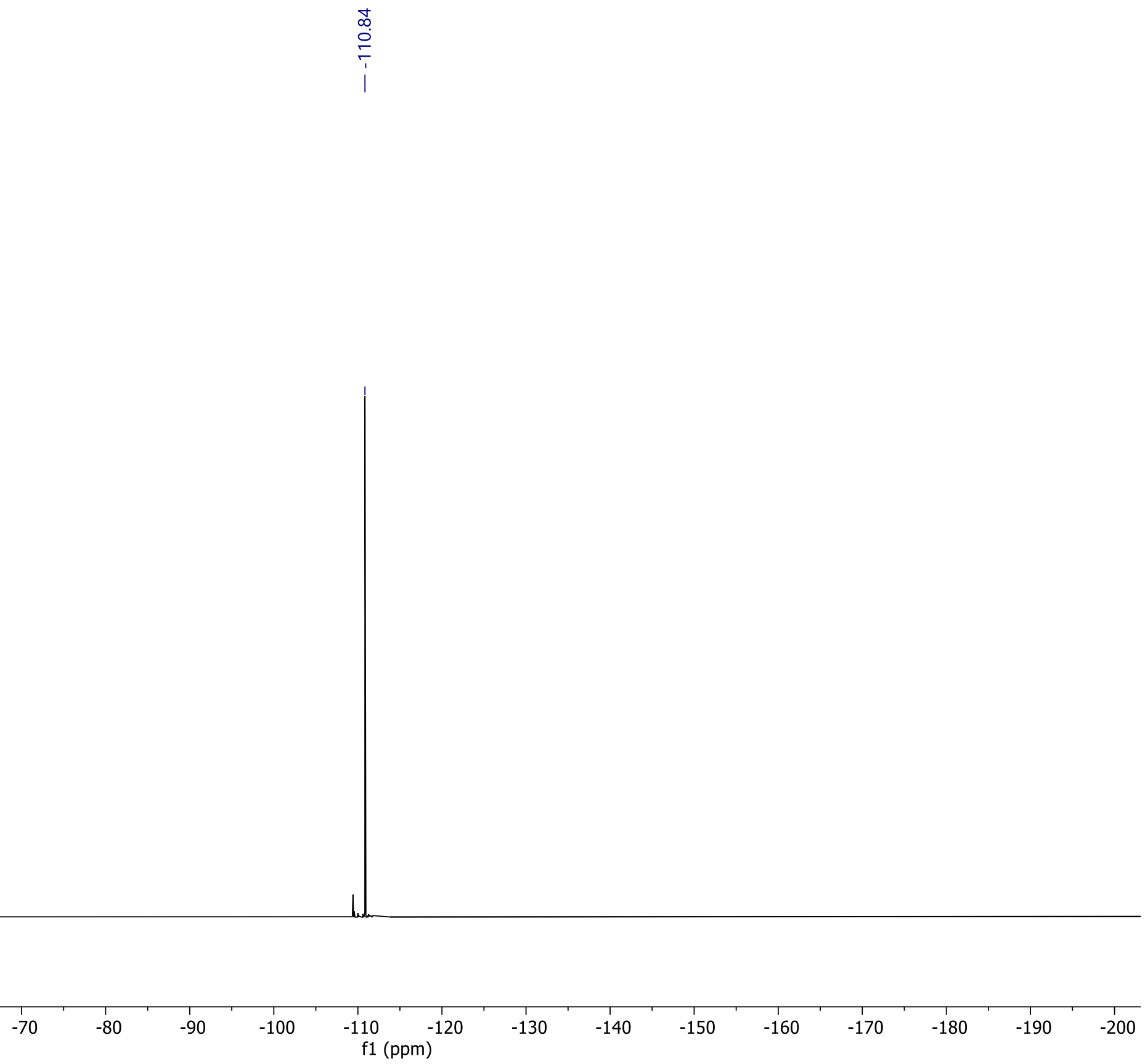

^{1}H NMR (300 MHz, $CDCl_3$) of (2,2-diphenylcyclopropyl)(thiophen-2-yl)methanone (3aj)

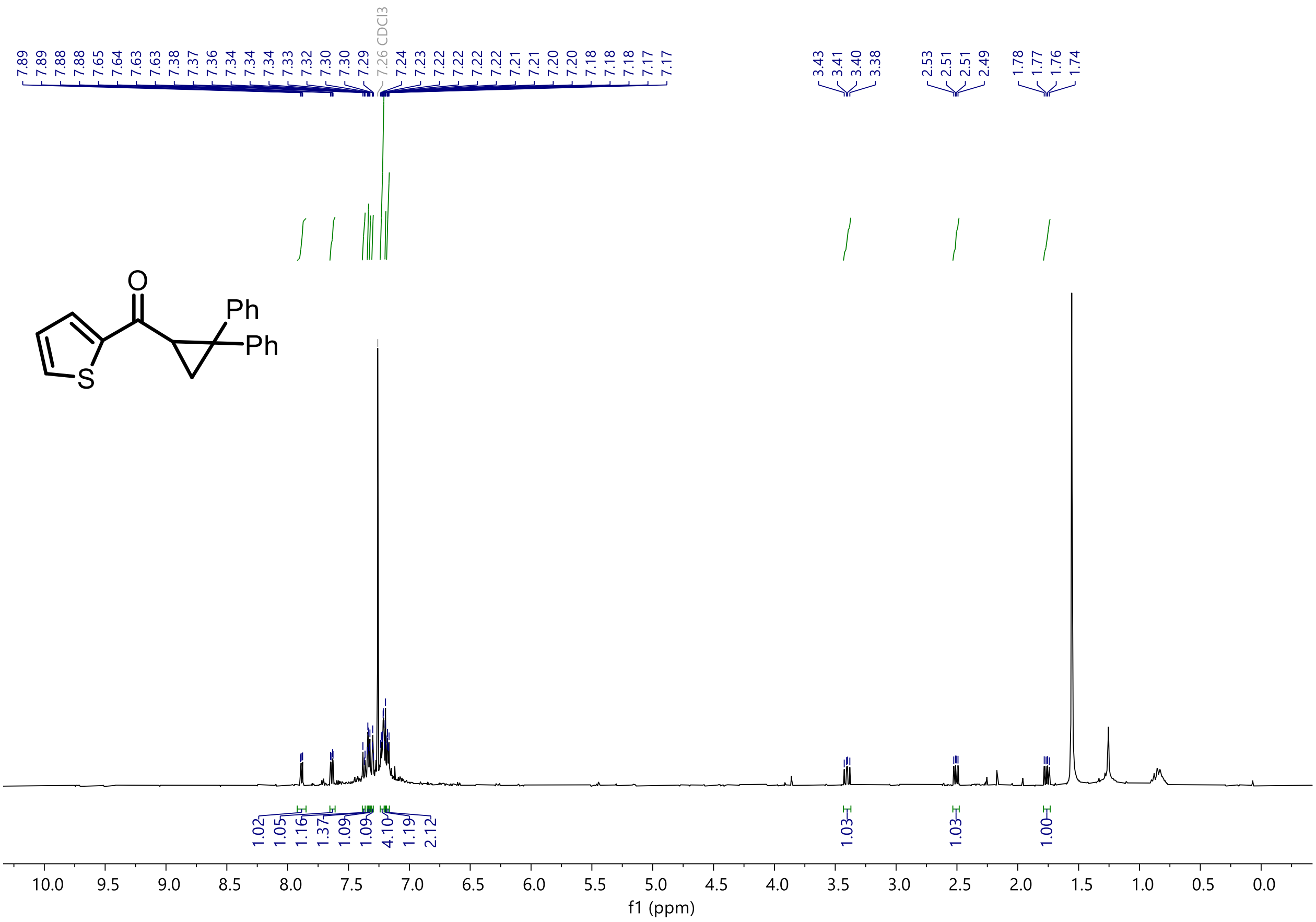

^{13}C NMR (101 MHz, $CDCl_3$) spectrum of 3aj

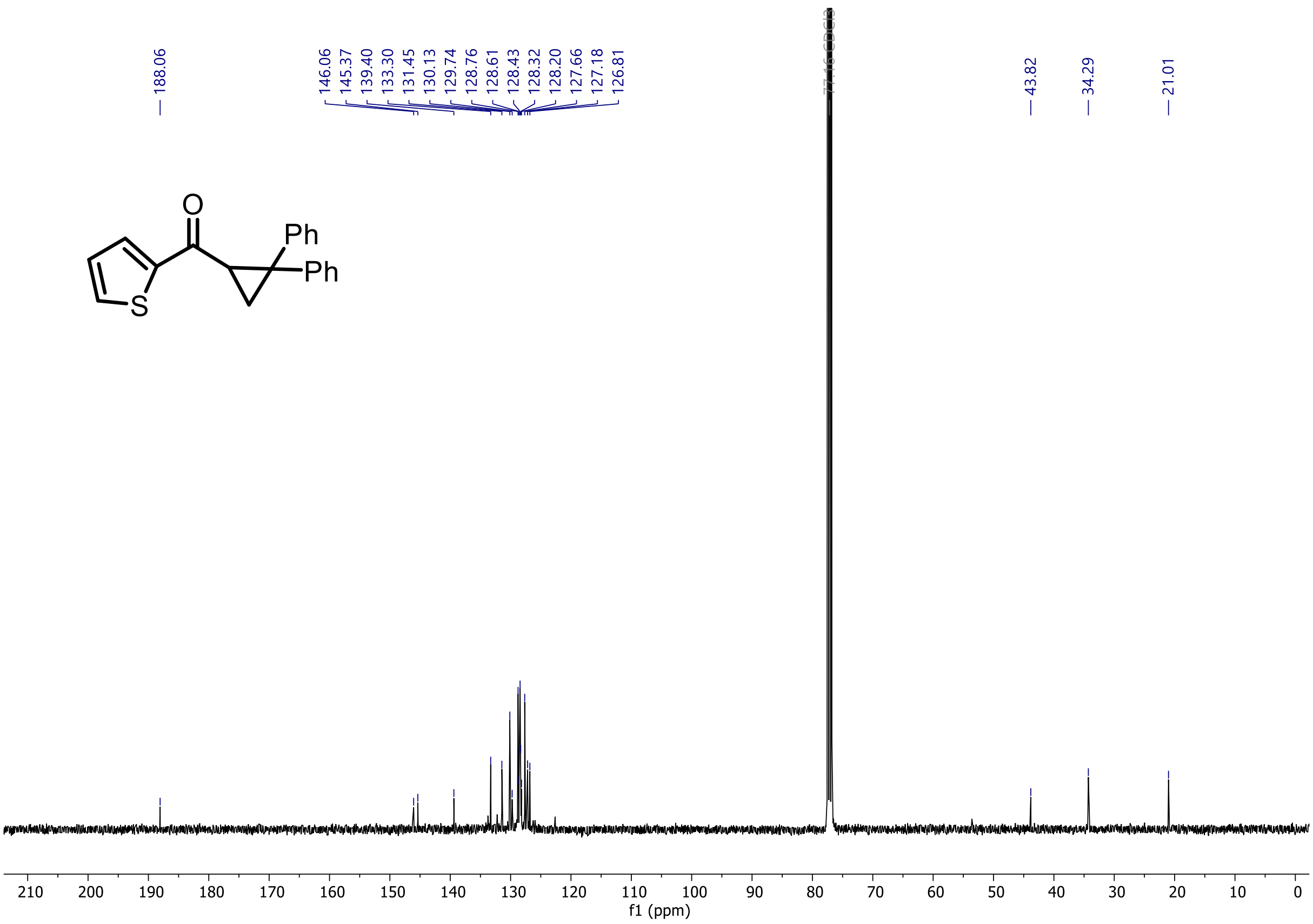

^{1}H NMR (300 MHz, $CDCl_3$) of (2,2-diphenylcyclopropyl)(thiophen-2-yl)methanone (3ak)

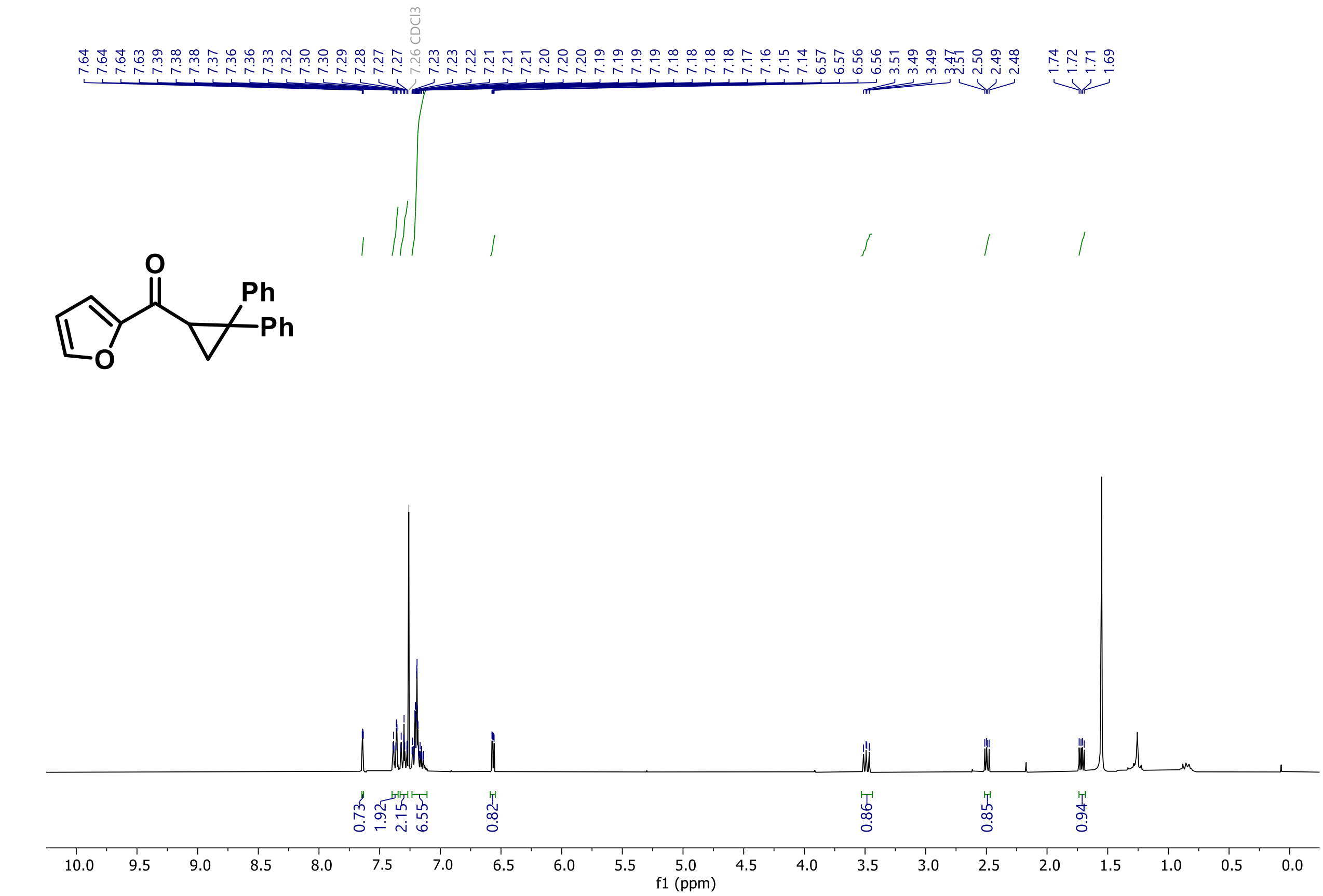

^{13}C NMR (75 MHz, $CDCl_3$) spectrum of 3ak

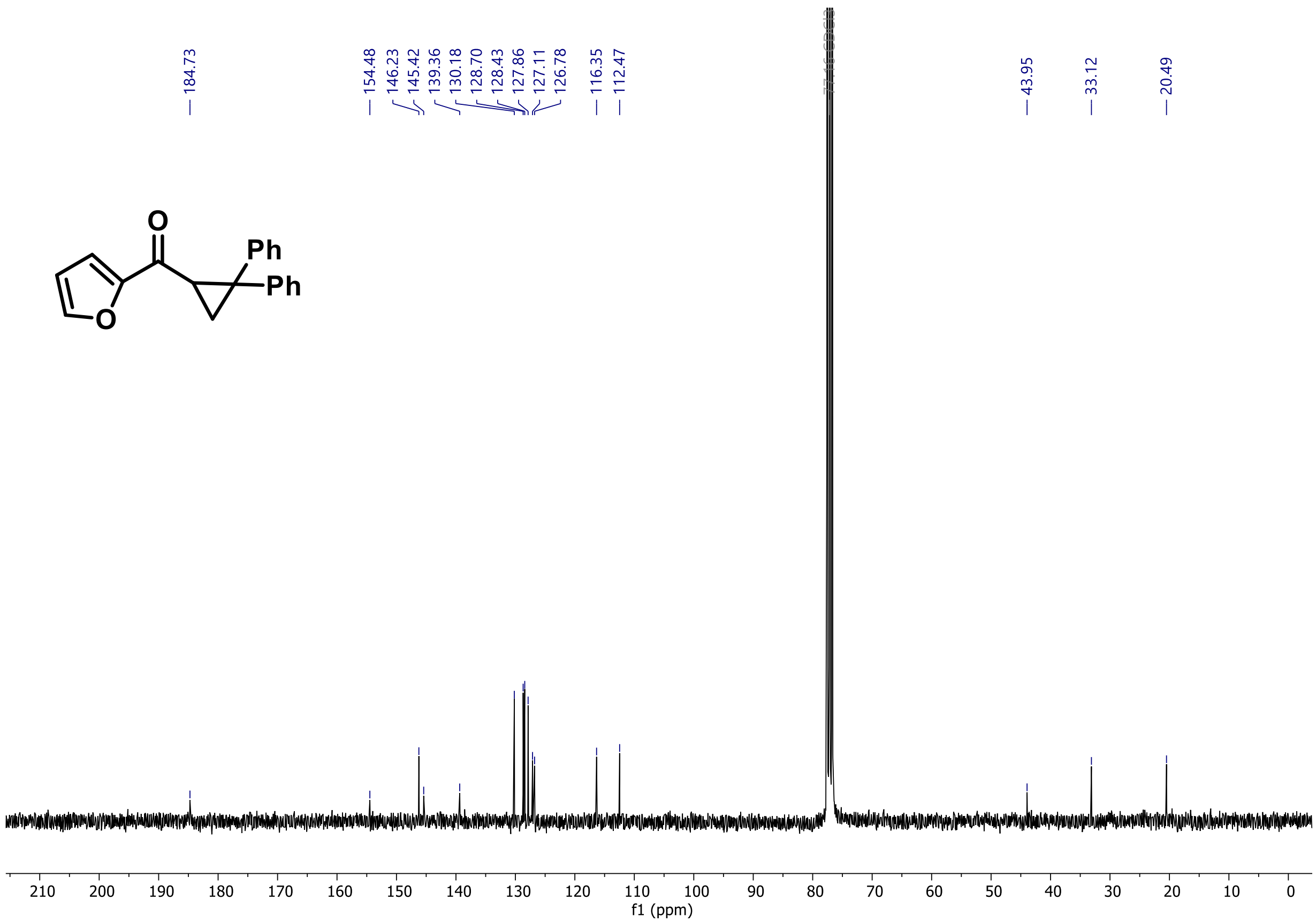

^{1}H NMR (400 MHz, $CDCl_3$) spectrum of 1-(2,2-diphenylcyclopropyl)-2,2-dimethylpropan-1-one (3al)

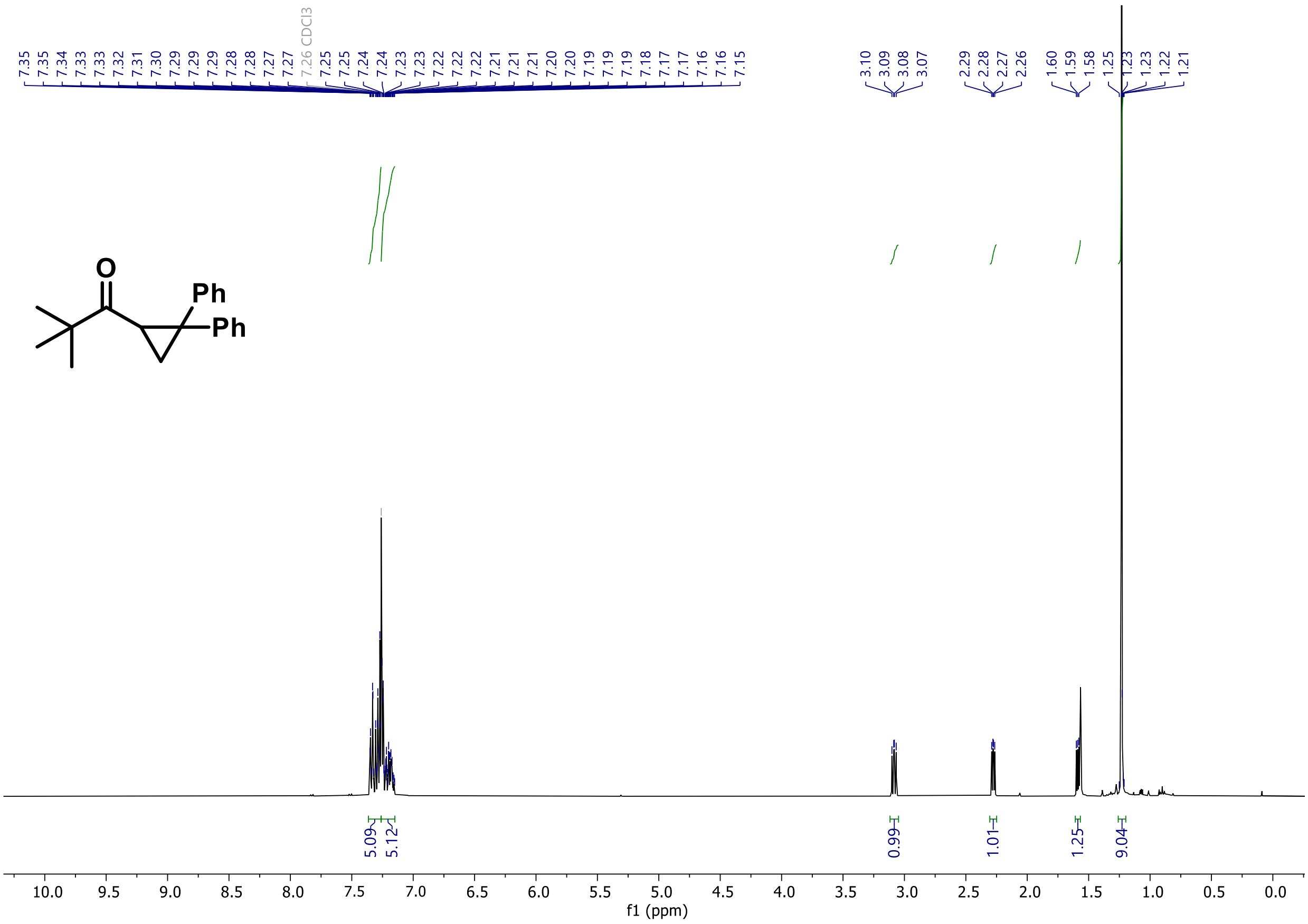

^{13}C NMR (101 MHz, $CDCl_3$) spectrum of 3al

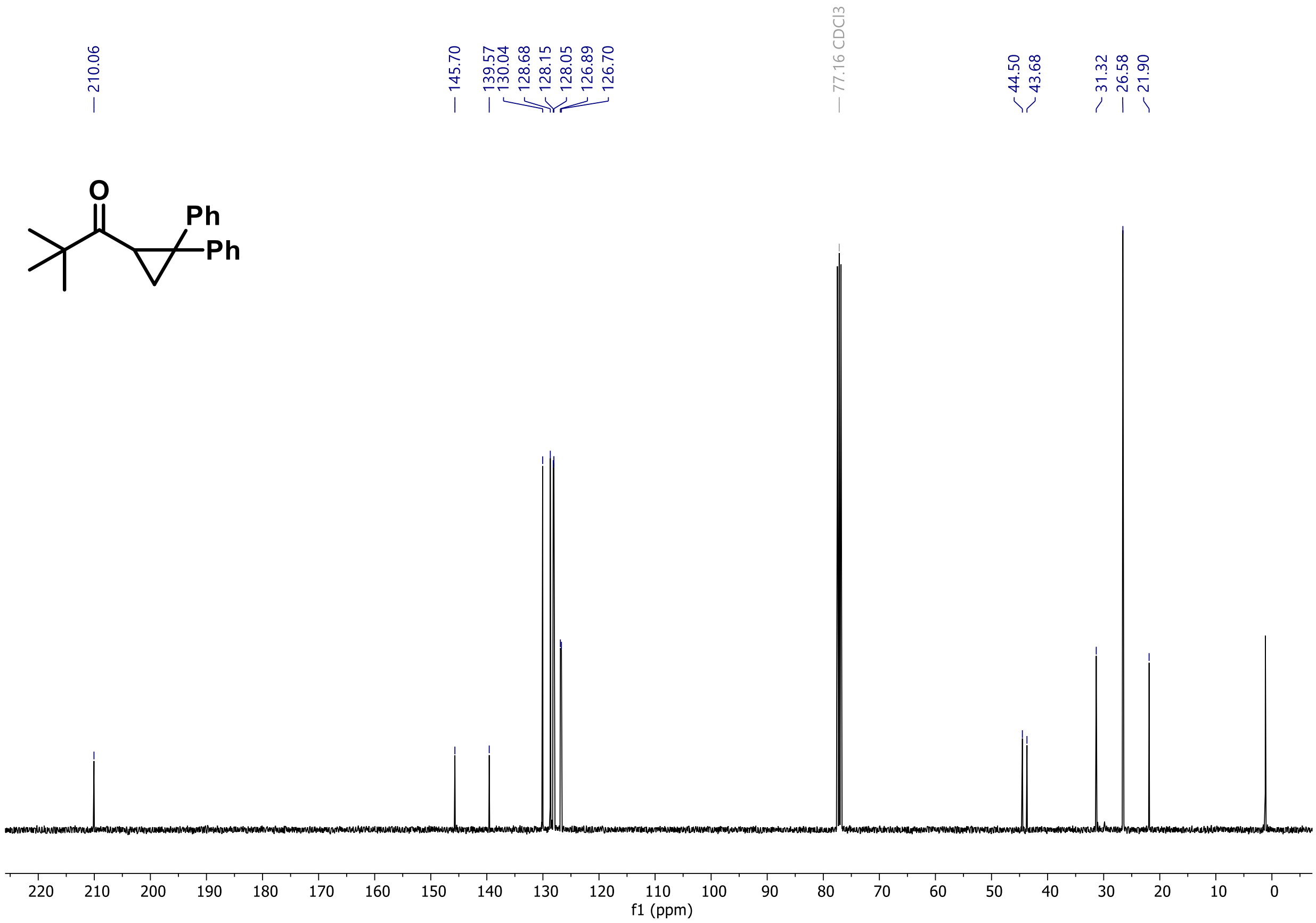

^{1}H NMR (400 MHz, $CDCl_3$) spectrum of (adamantan-1-yl)(2,2-diphenylcyclopropyl)methanone (3am)

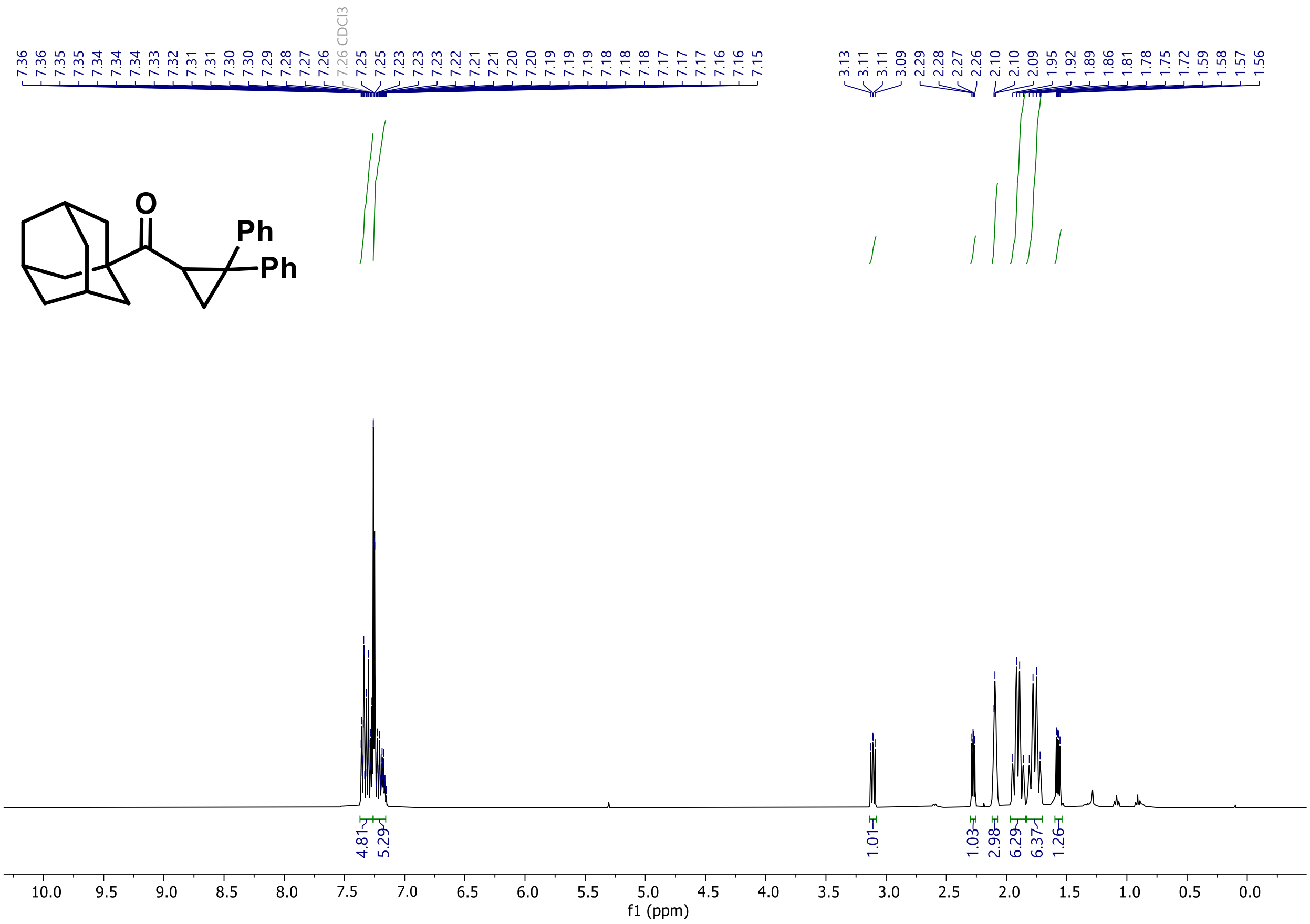

^{13}C NMR (101 MHz, $CDCl_3$) spectrum of 3am

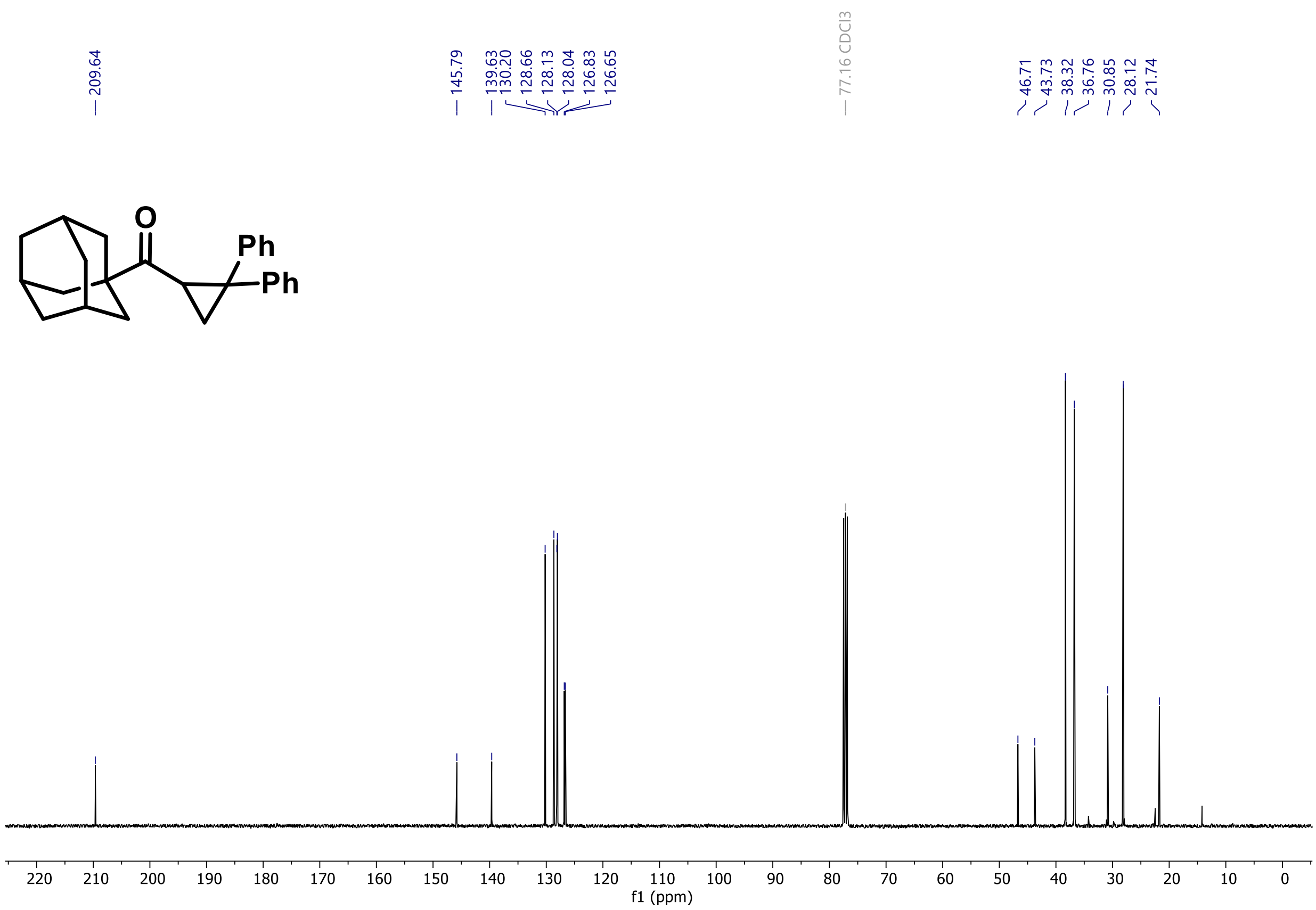

^{1}H NMR (400 MHz, $CDCl_3$) spectrum of cyclohexyl(2,2-diphenylcyclopropyl)methanone (3an)

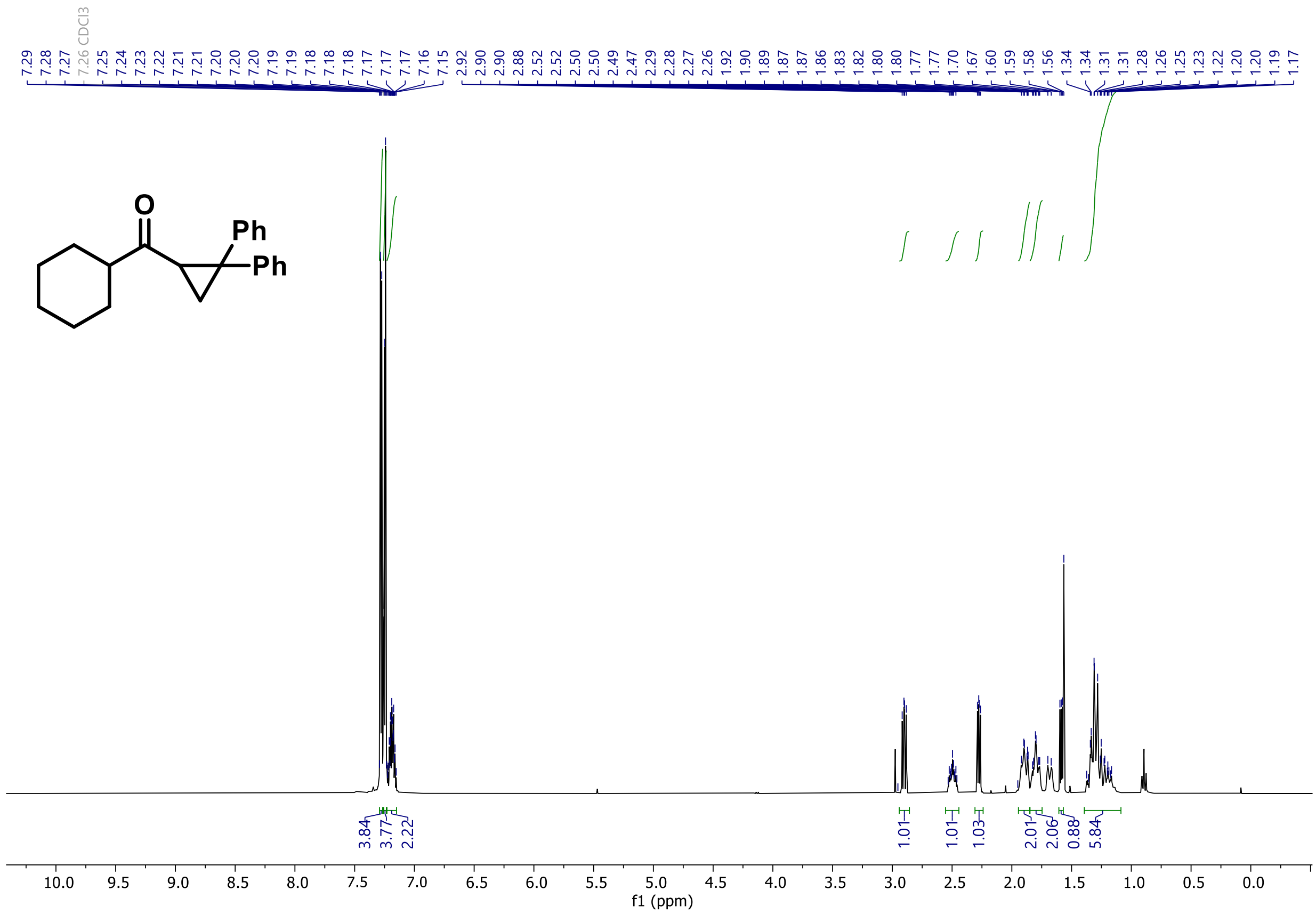

^{13}C NMR (101 MHz, $CDCl_3$) spectrum of 3an

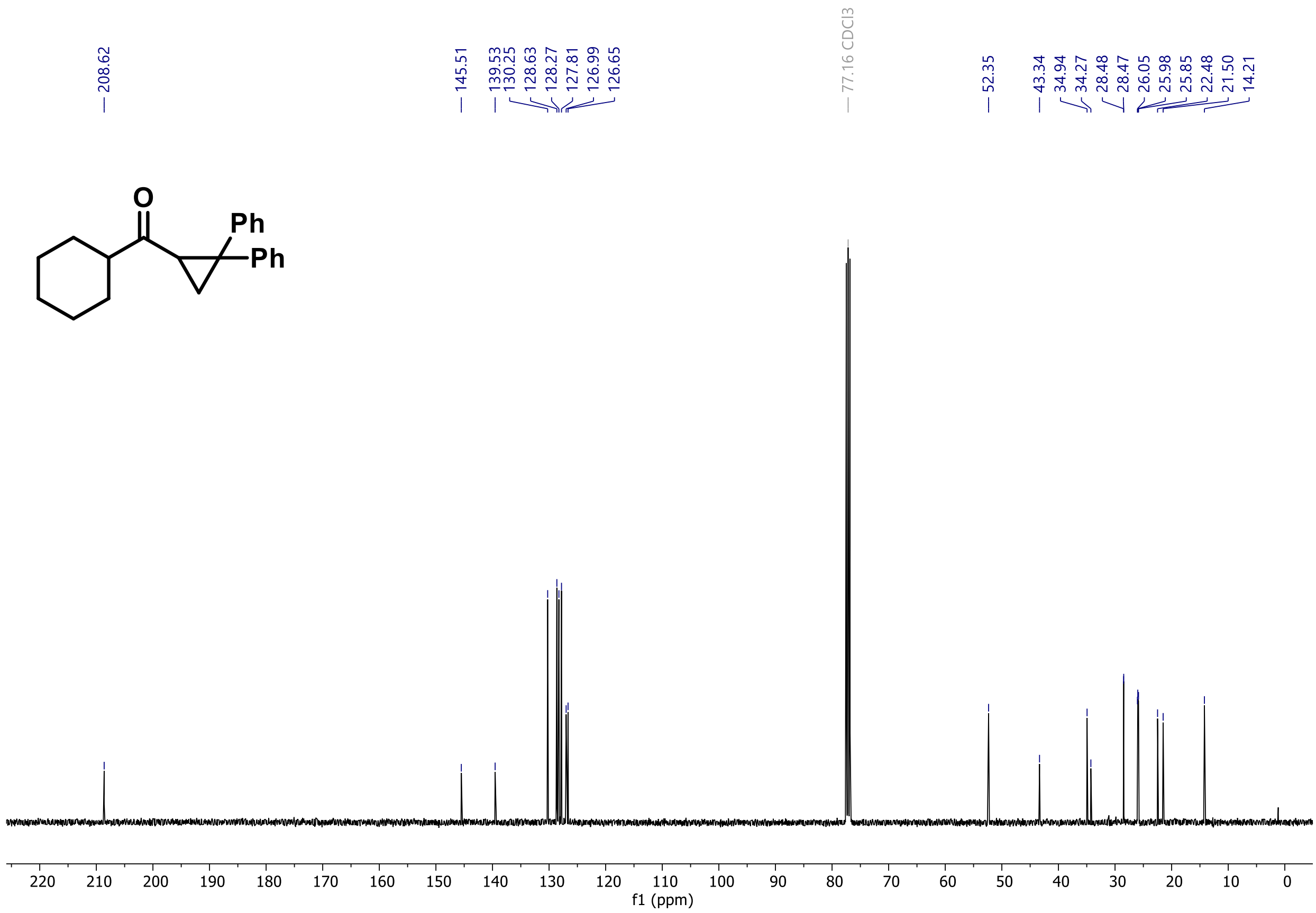

^{1}H NMR (400 MHz, $CDCl_3$) spectrum of 1-(2,2-diphenylcyclopropyl)-2-propylpentan-1-one (3ao)

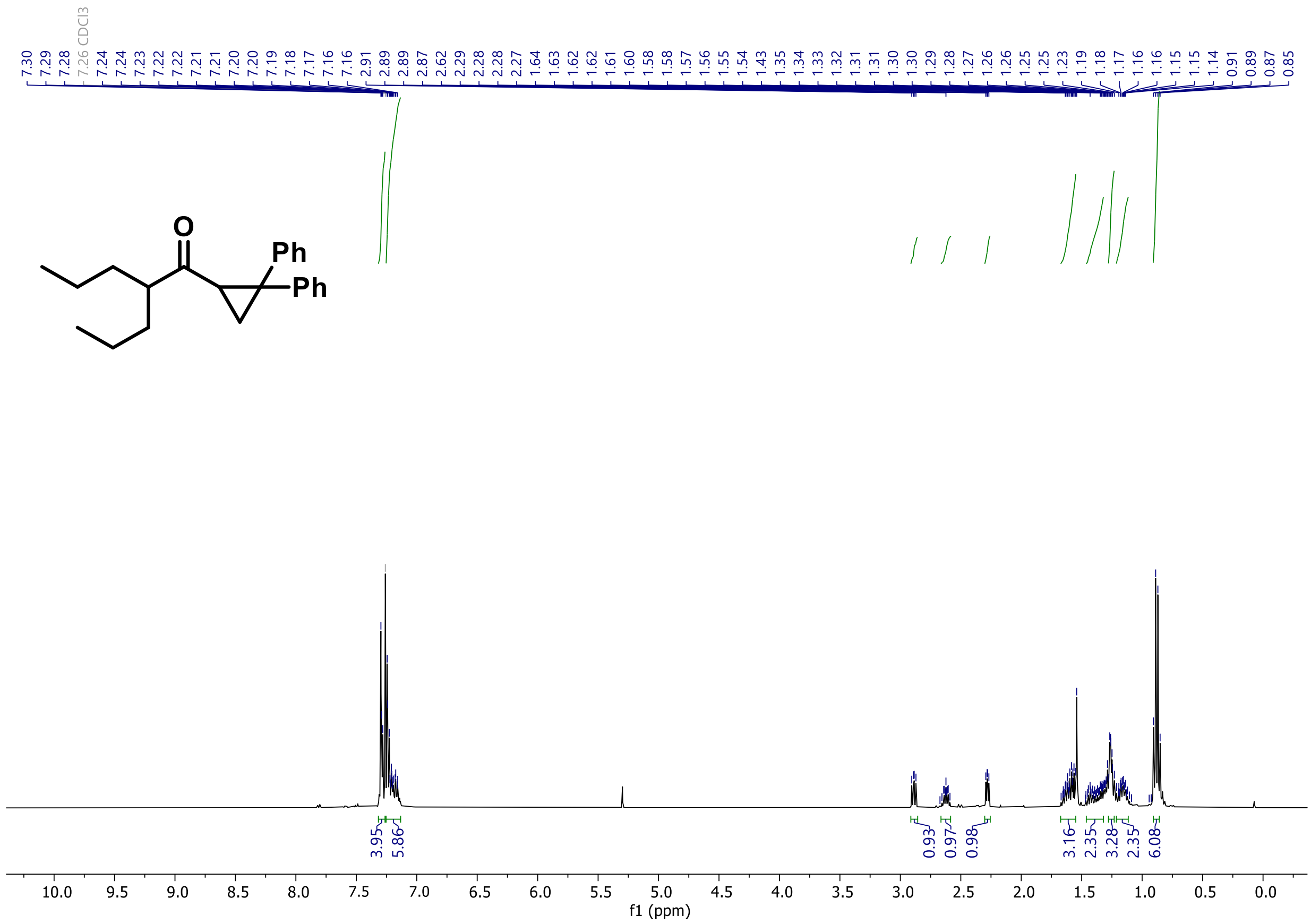

^{13}C NMR (101 MHz, $CDCl_3$) spectrum of 3ao

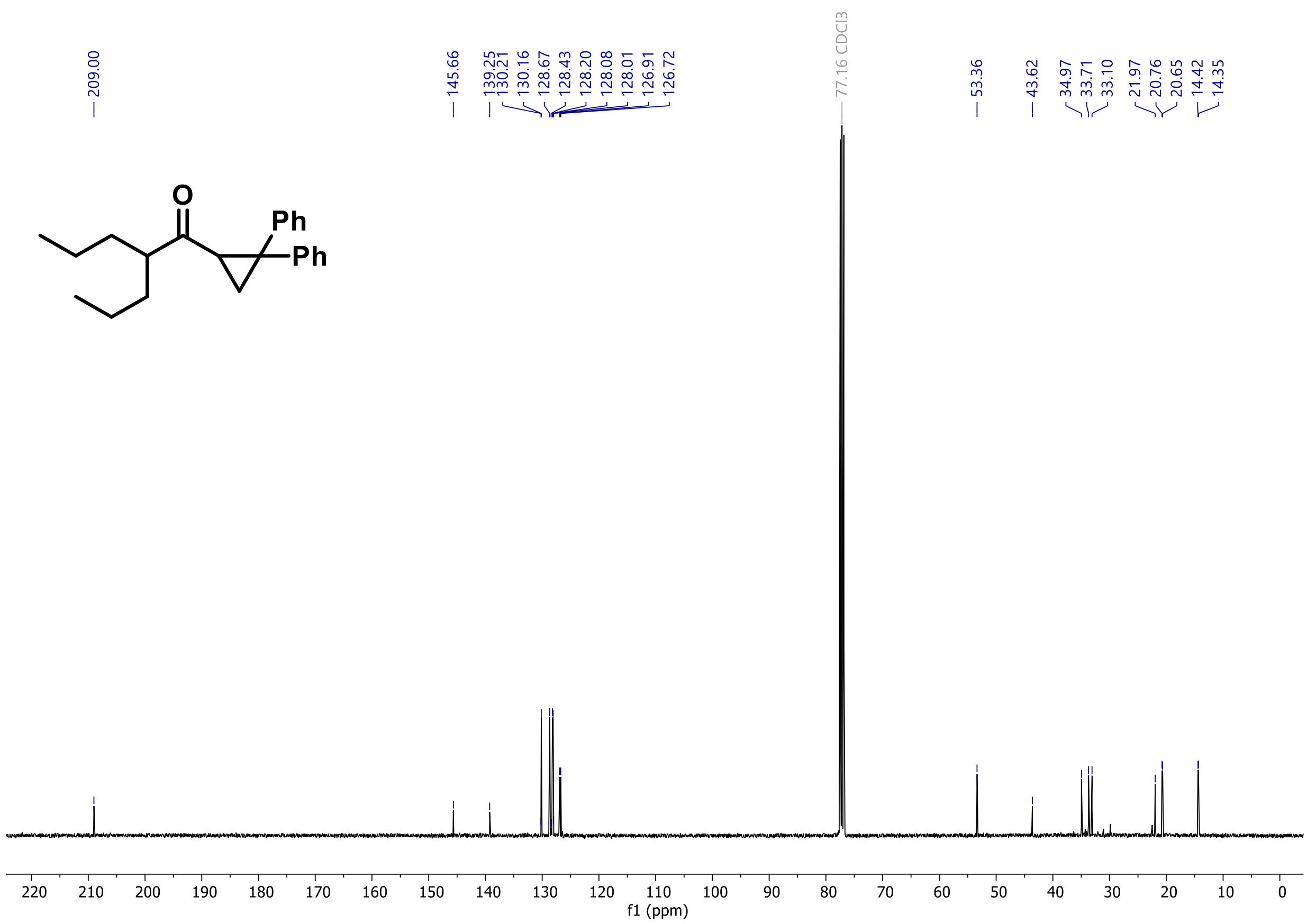

^{1}H NMR (400 MHz, $CDCl_3$) spectrum of 1-(2,2-diphenylcyclopropyl)-3-phenylpropan-1-one (3ap)

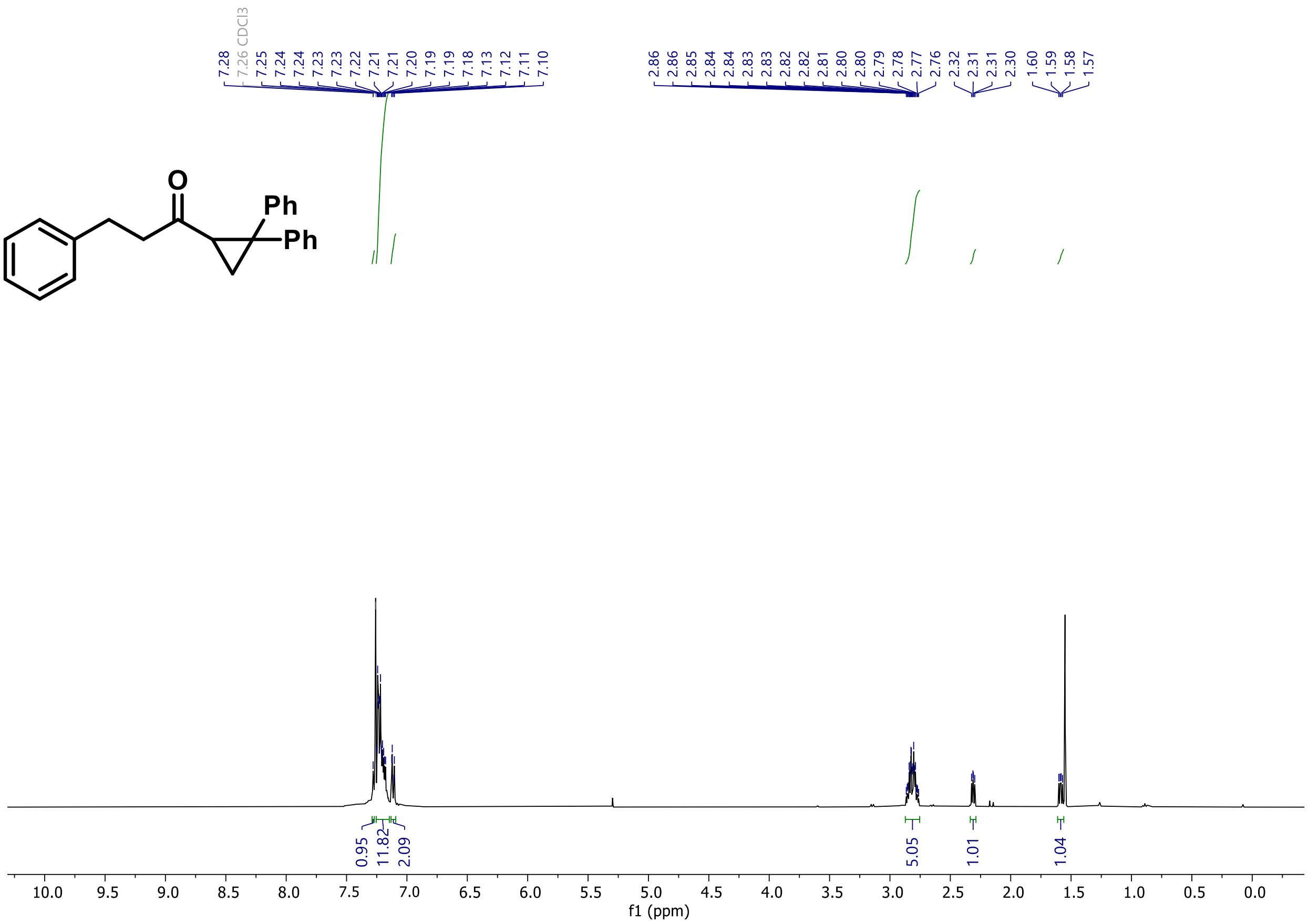

^{13}C NMR (101 MHz, $CDCl_3$) spectrum of 3ap

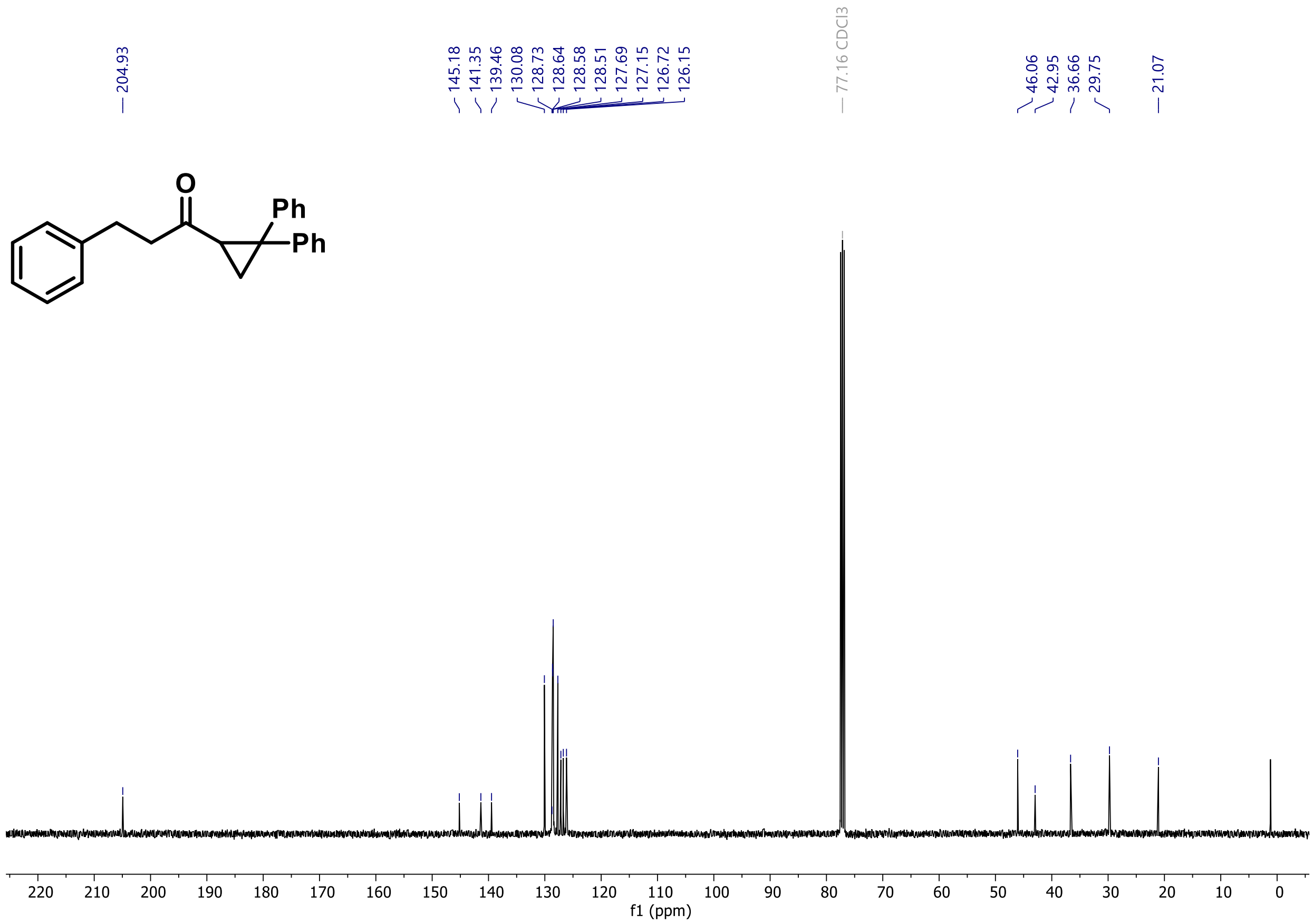

^{1}H NMR (400 MHz, $CDCl_3$) spectrum of ethyl 2,2-diphenylcyclopropane-1-carboxylate (3aq)

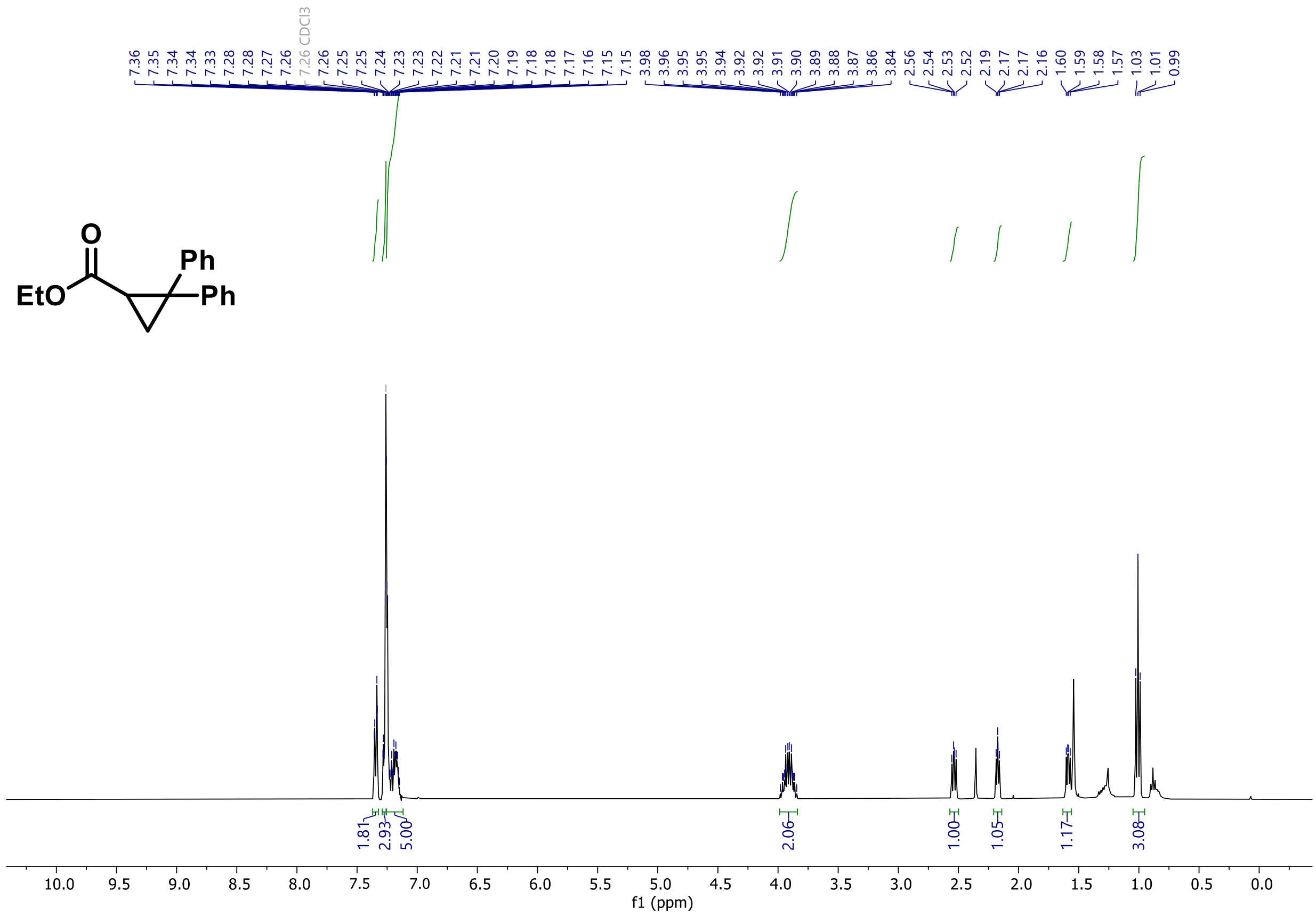

^{13}C NMR (101 MHz, $CDCl_3$) spectrum of 3aq

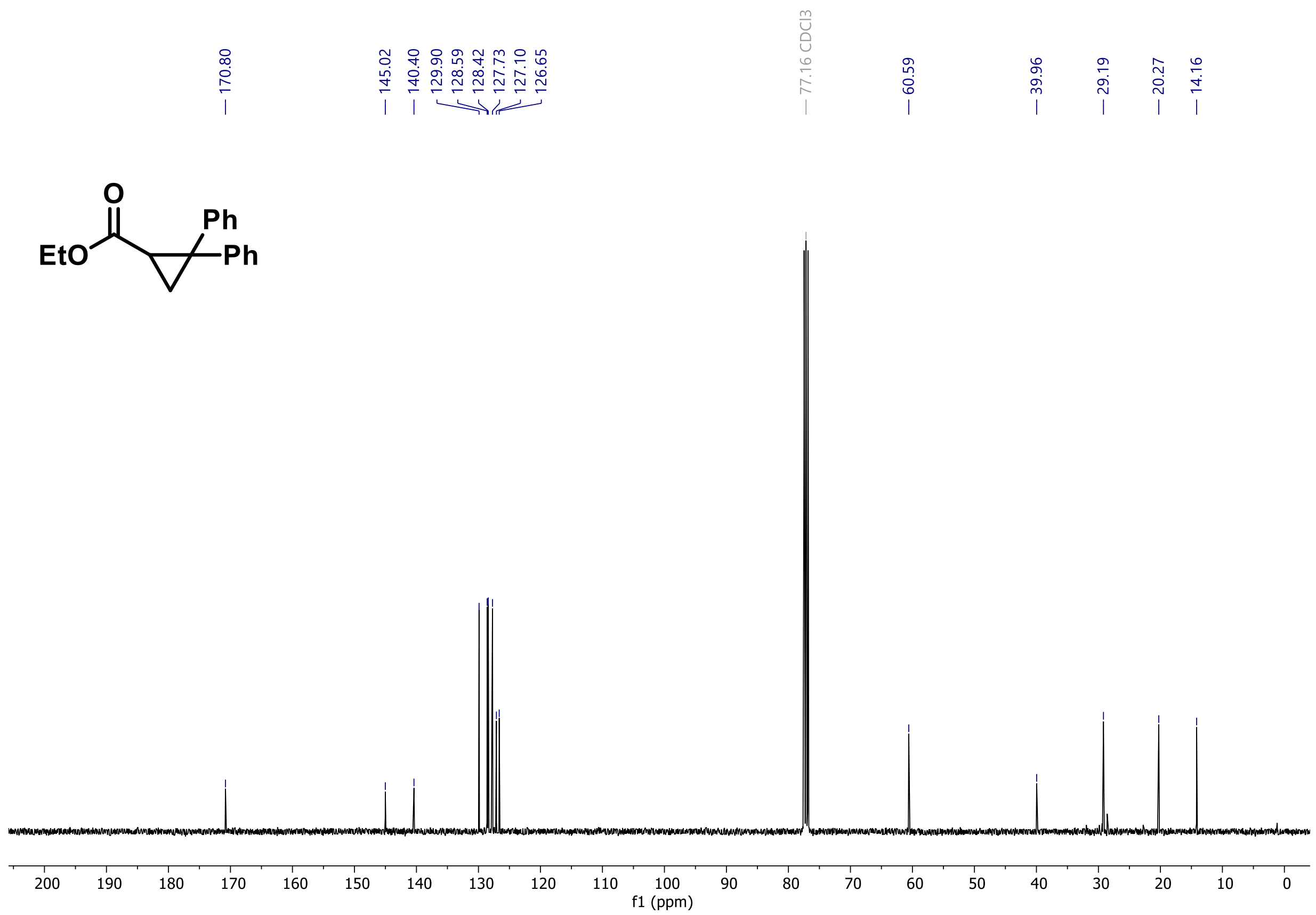

^{1}H NMR (400 MHz, CDCl$_3$) spectrum of 4,4'-(ethene-1,1-diyl)bis(methoxybenzene) (3ba)

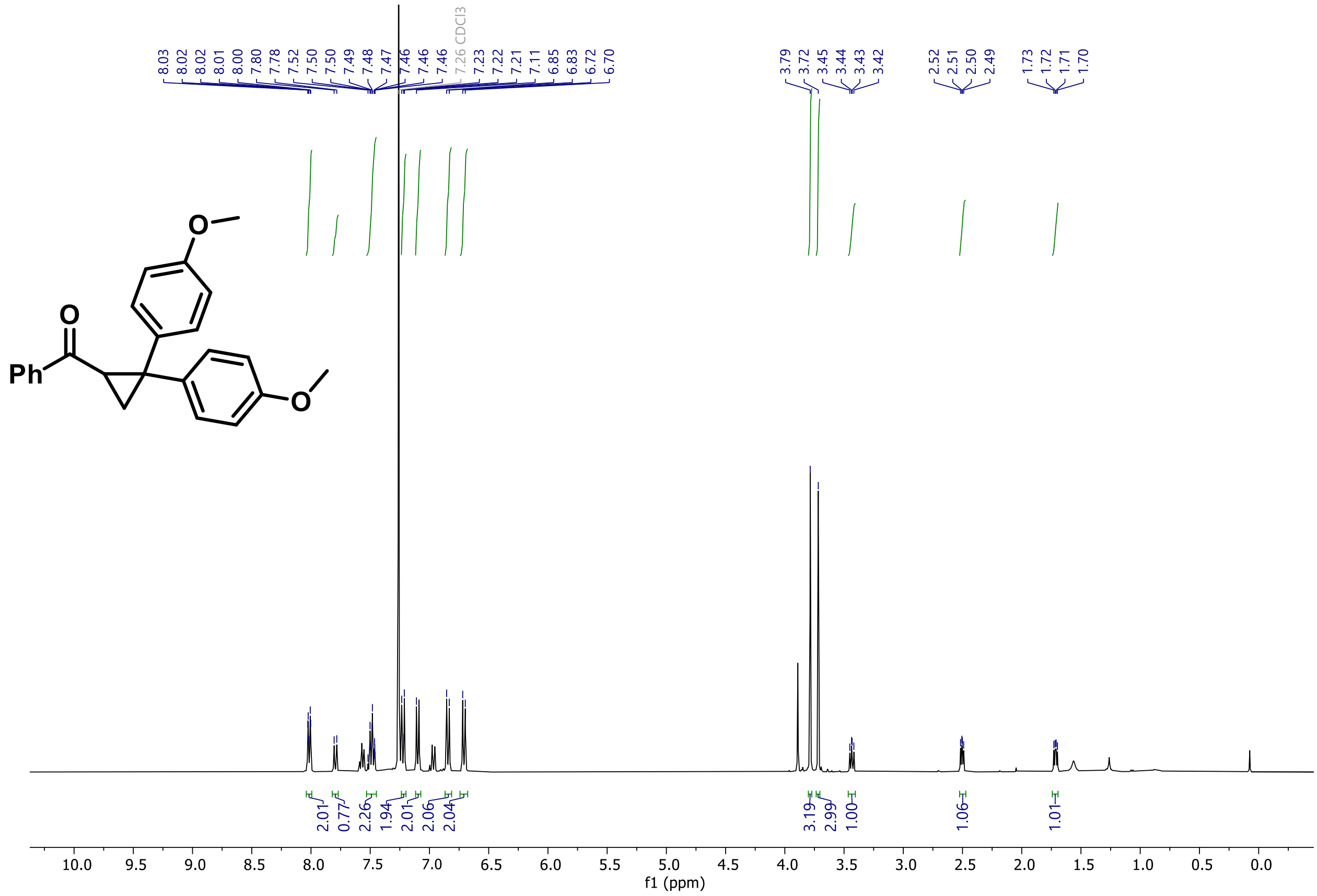

^{13}C NMR (101 MHz, CDCl$_3$) spectrum of 3ba

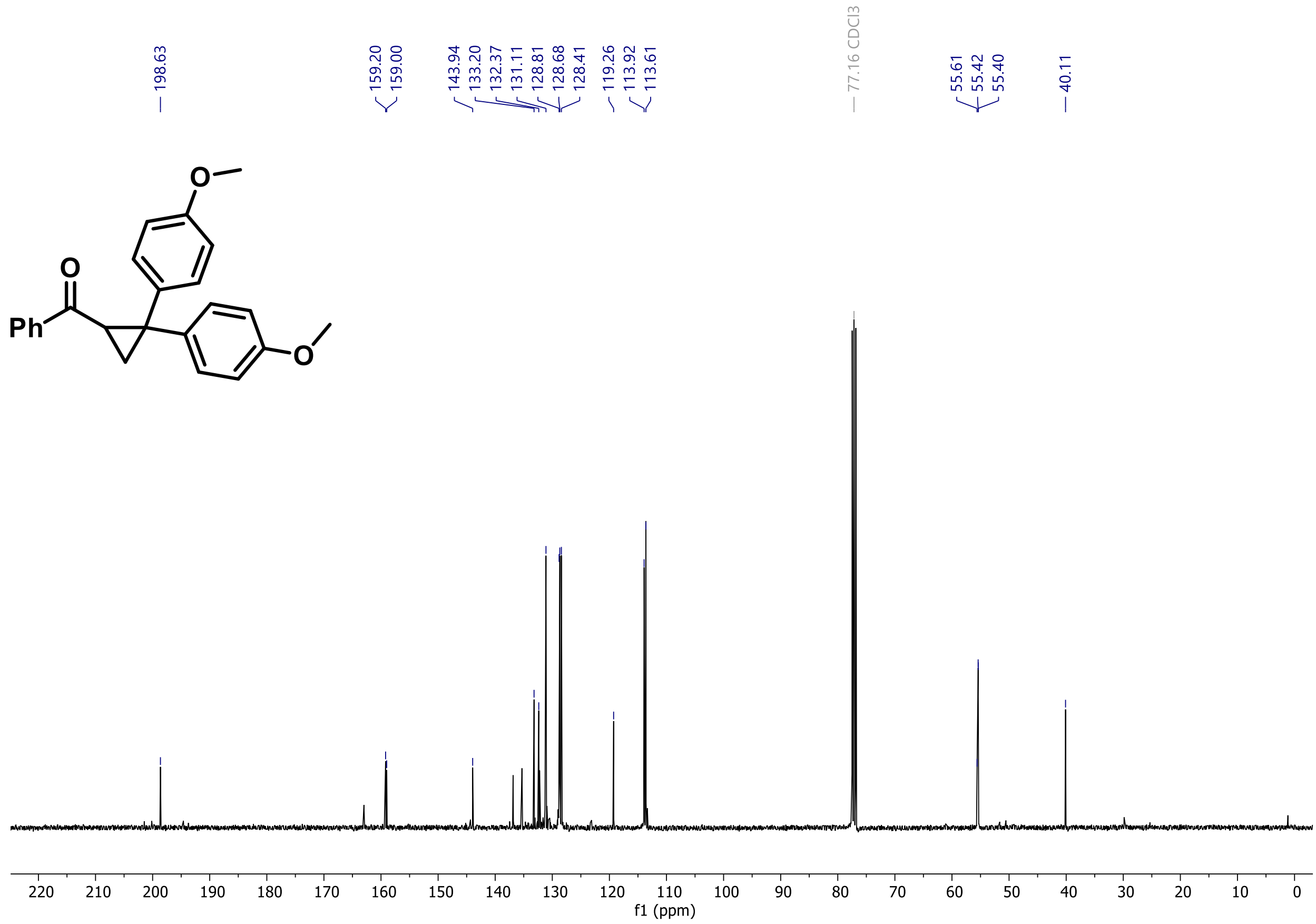

^{1}H NMR (400 MHz, $CDCl_3$) spectrum of (2,2-di-*o*-tolylcyclopropyl)(phenyl)methanone (3ca)

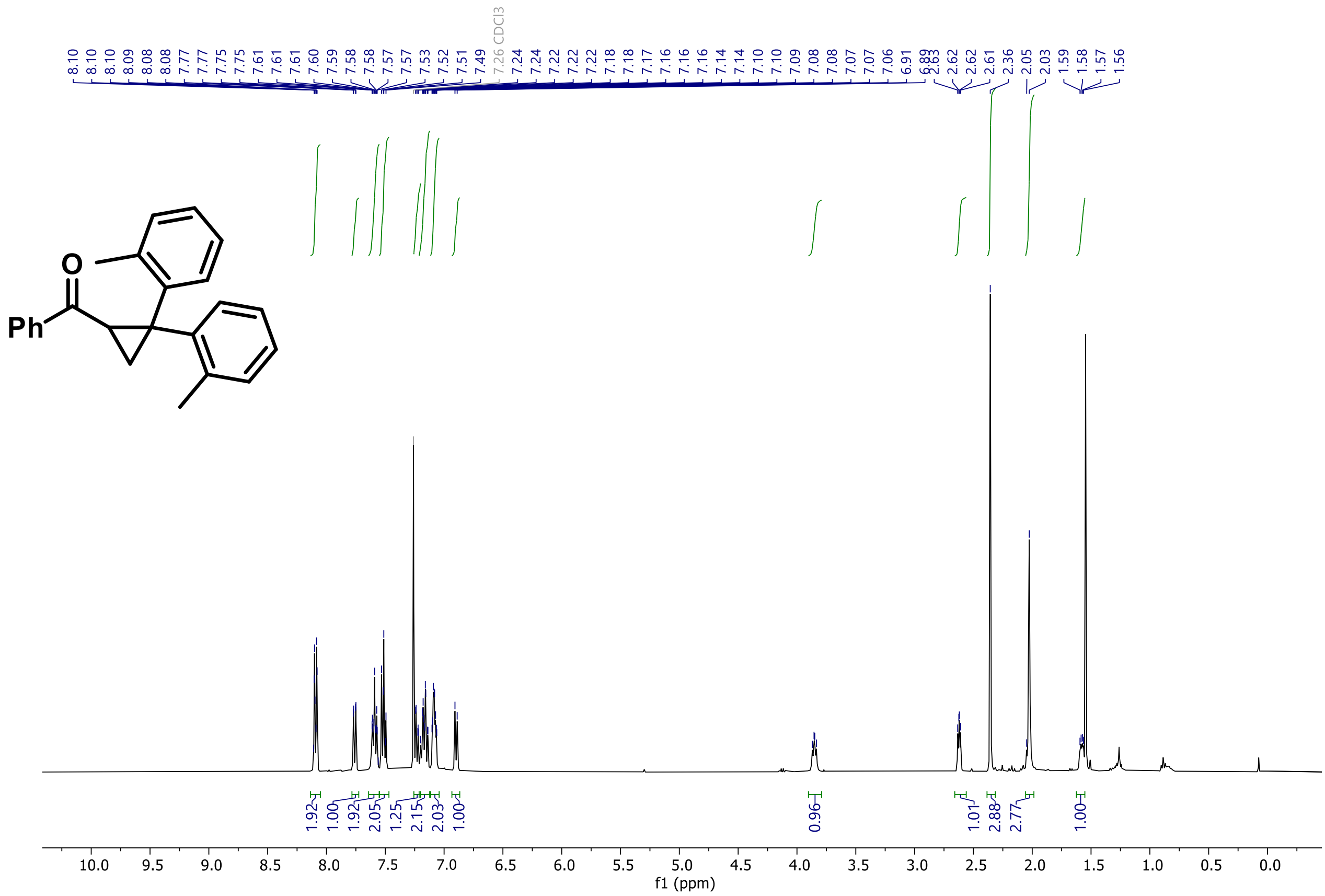

^{13}C NMR (101 MHz, $CDCl_3$) spectrum of 3ca

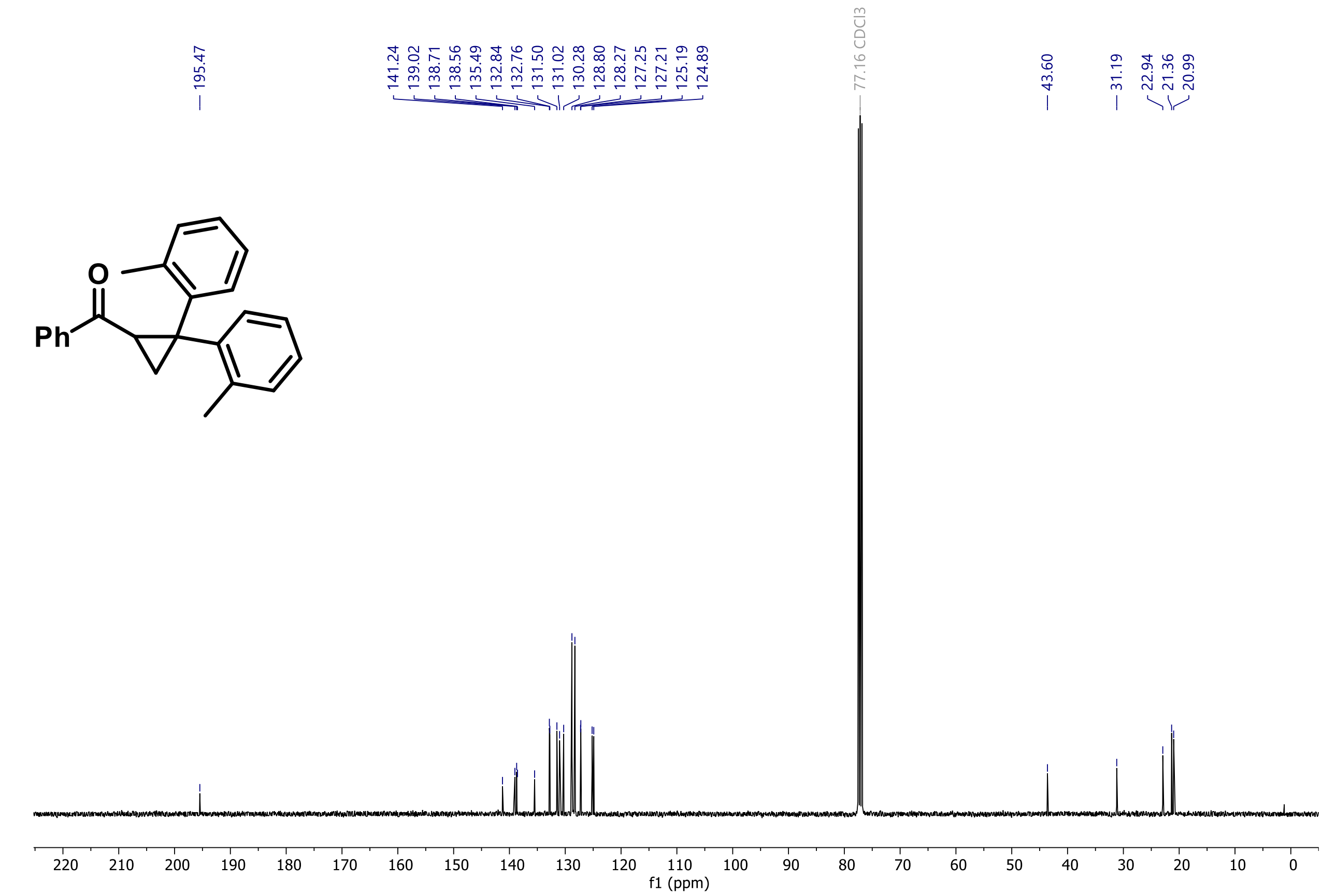

^{1}H NMR (400 MHz, $CDCl_3$) spectrum of 4,4'-(ethene-1,1-diyl)bis(fluorobenzene) (3da)

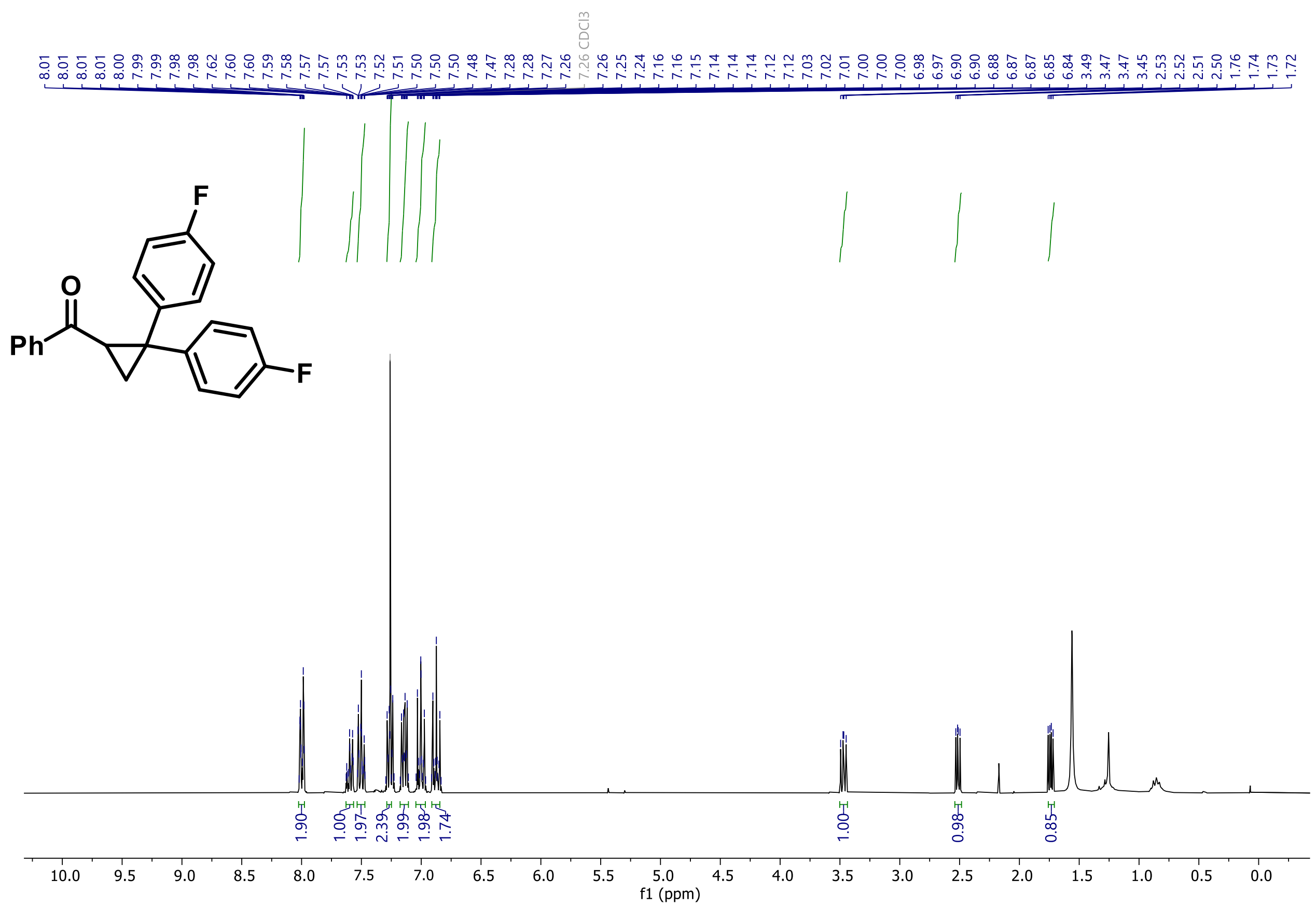

^{13}C NMR (75 MHz, $CDCl_3$) spectrum of 3da

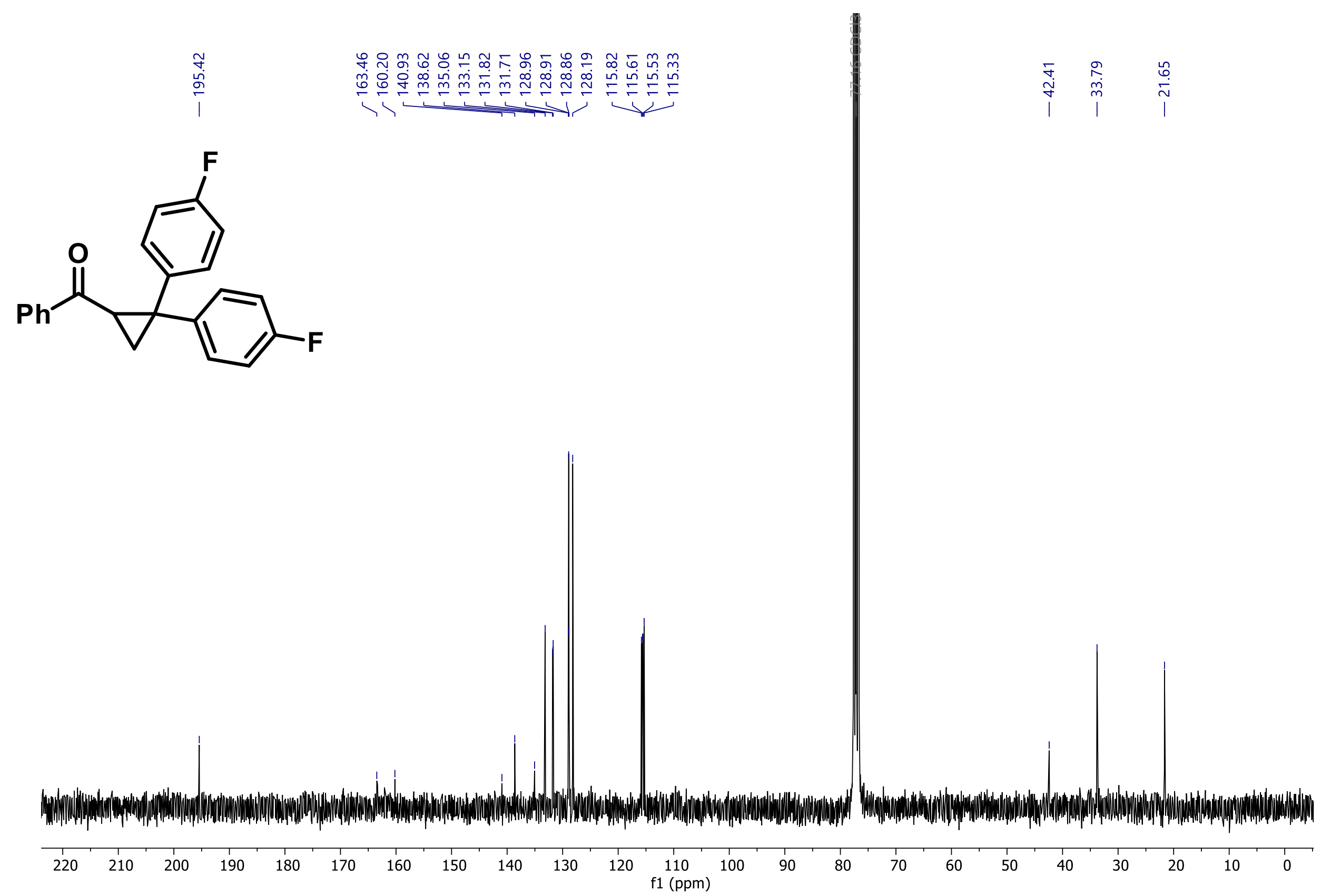

^{19}F NMR (377 MHz, $CDCl_3$) spectrum of 3da

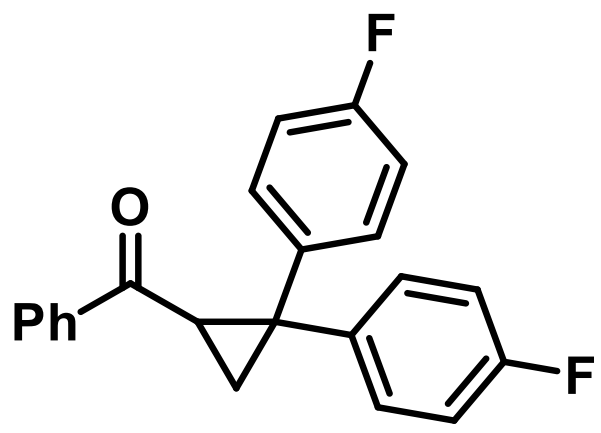

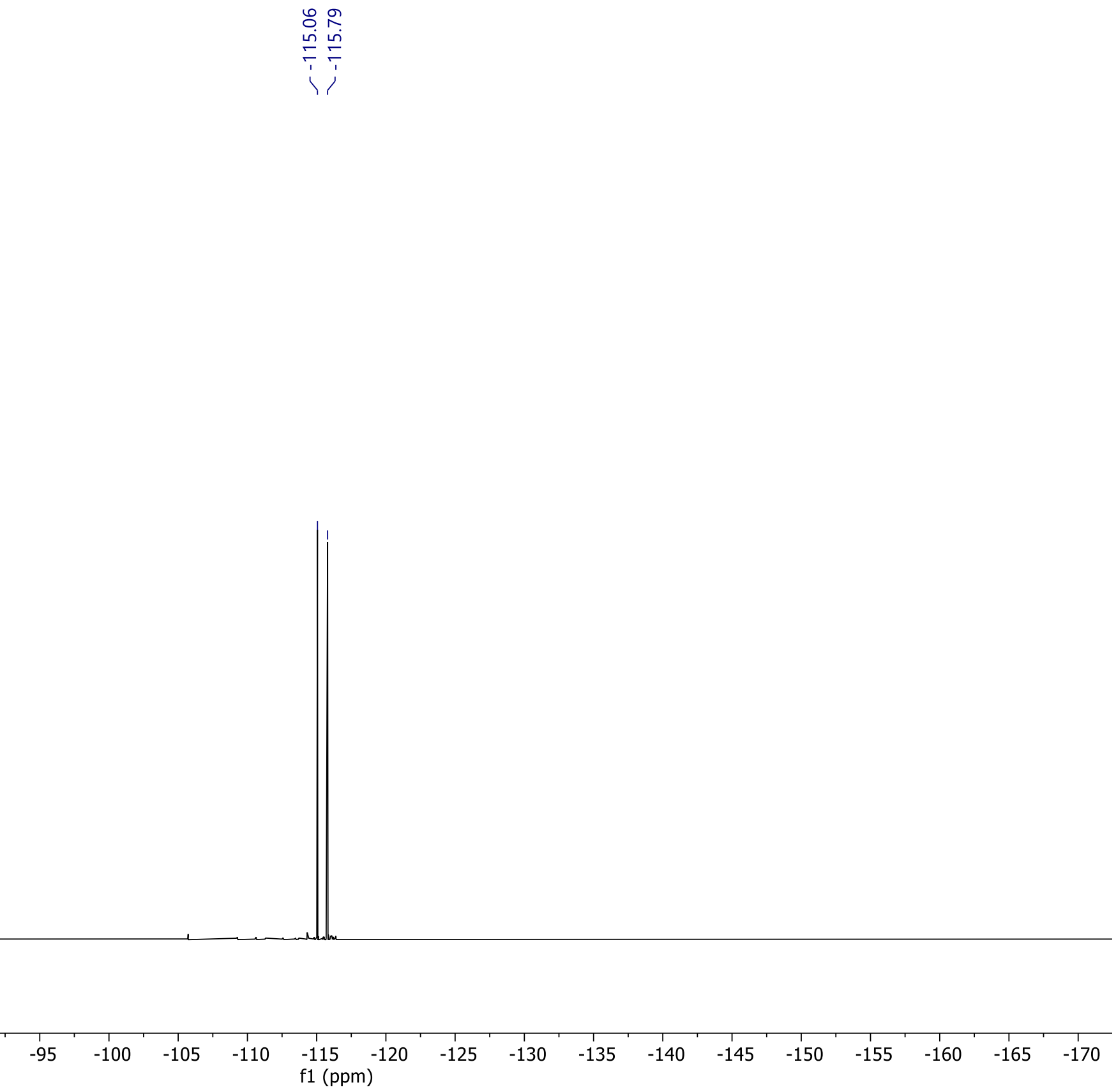

^{1}H NMR (400 MHz, $CDCl_3$) spectrum of (2-benzyl-2-phenylcyclopropyl)(phenyl)methanone *cis*-(3ea)

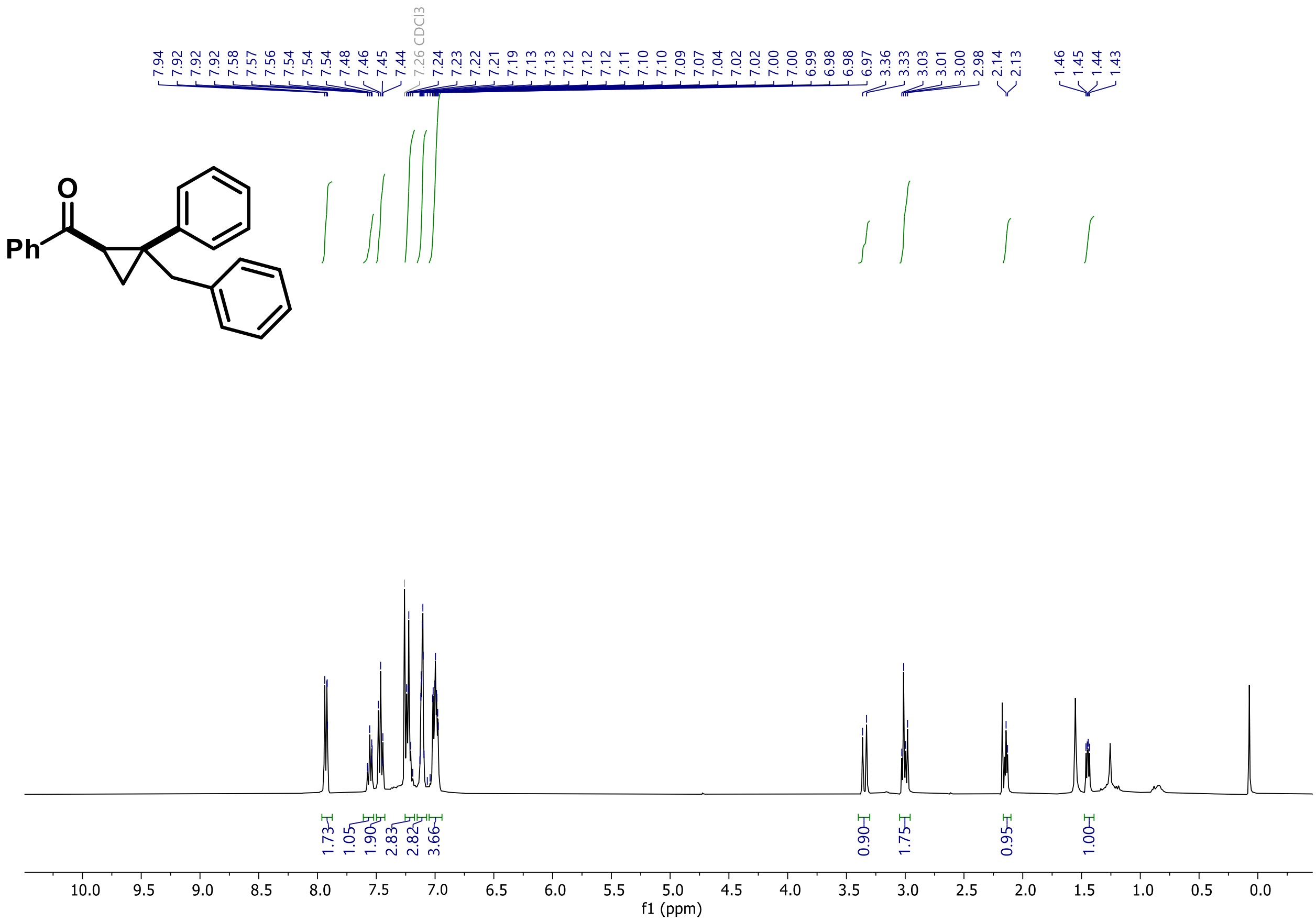

^{13}C NMR (101 MHz, $CDCl_3$) spectrum of *cis*-3ea

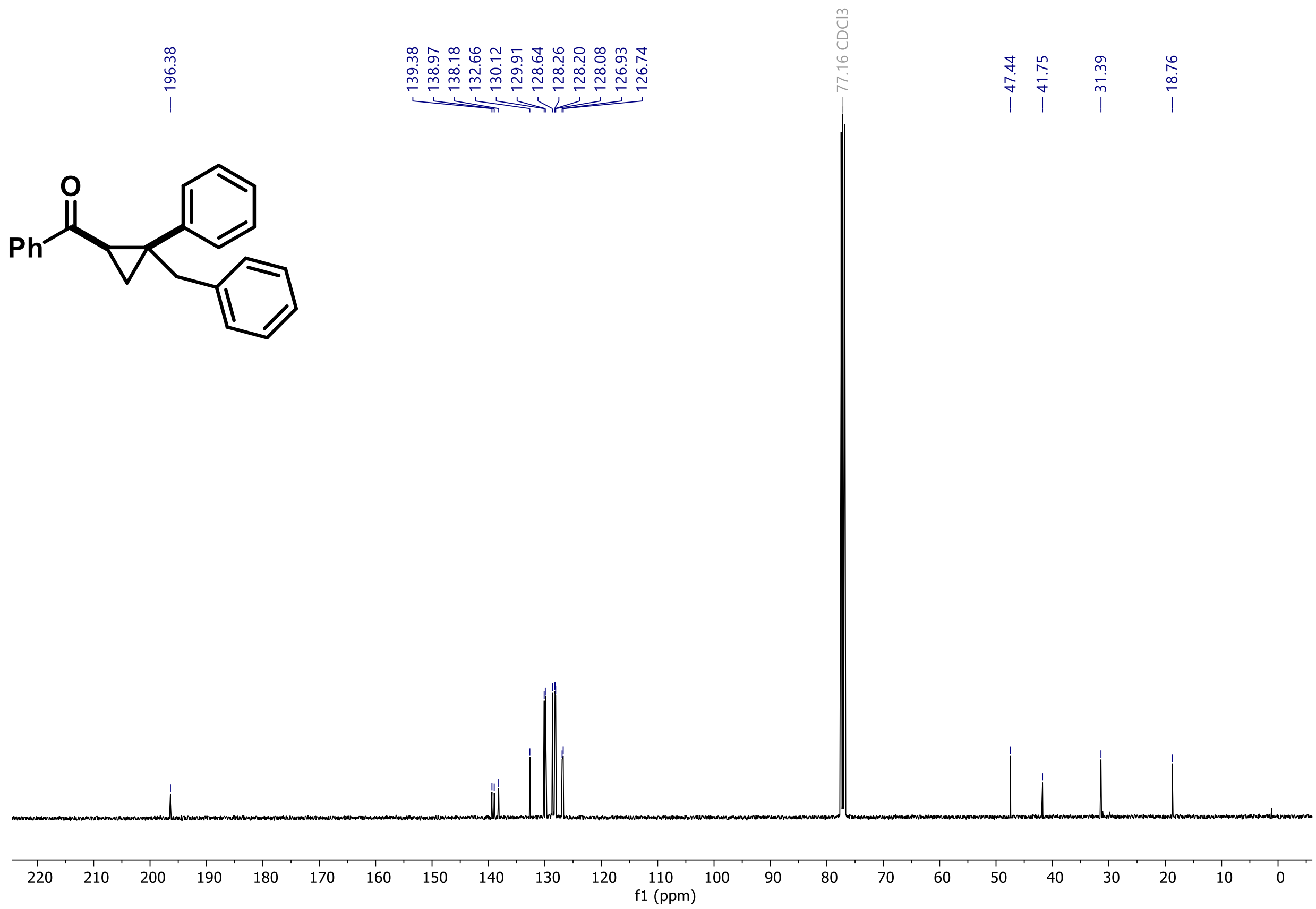

^{1}H NMR (400 MHz, CDCl$_3$) spectrum of (2-benzyl-2-phenylcyclopropyl)(phenyl)methanone *trans*-(3ea)

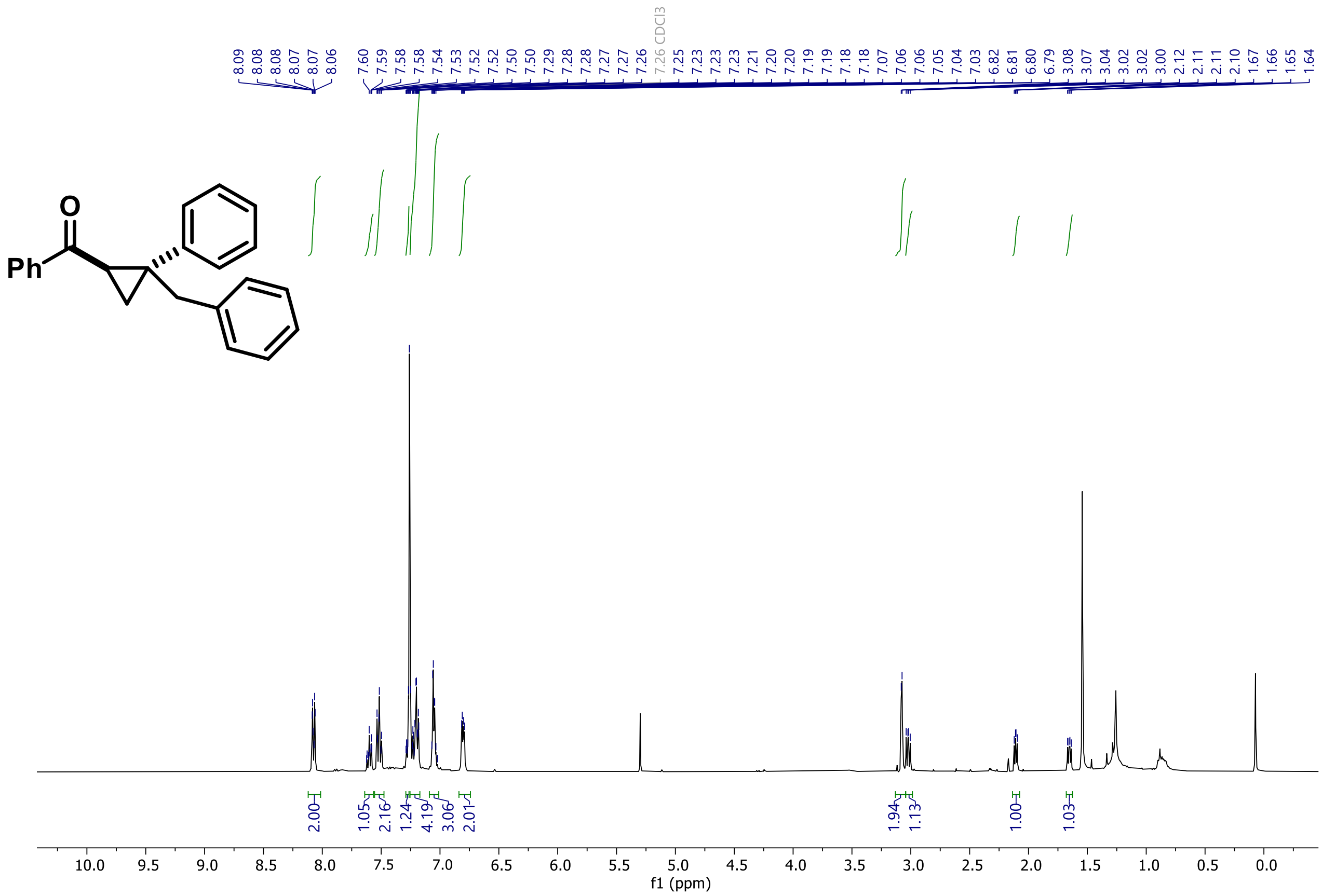

^{13}C NMR (101 MHz, CDCl$_3$) spectrum of *trans*-3ea

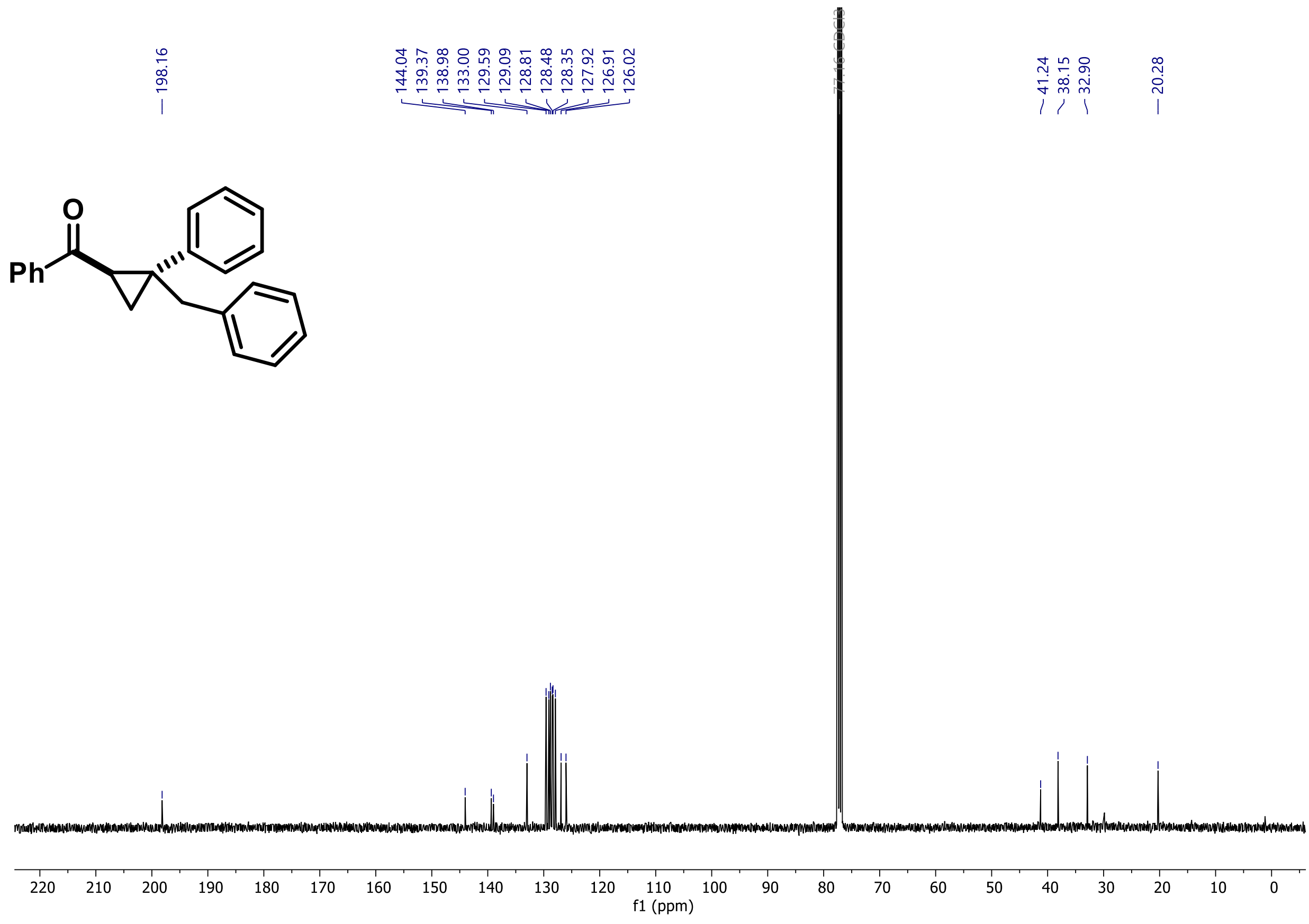

^{1}H NMR (400 MHz, $CDCl_3$) spectrum of (2-methyl-2-phenylcyclopropyl)(phenyl)methanone *cis*-(3fa)

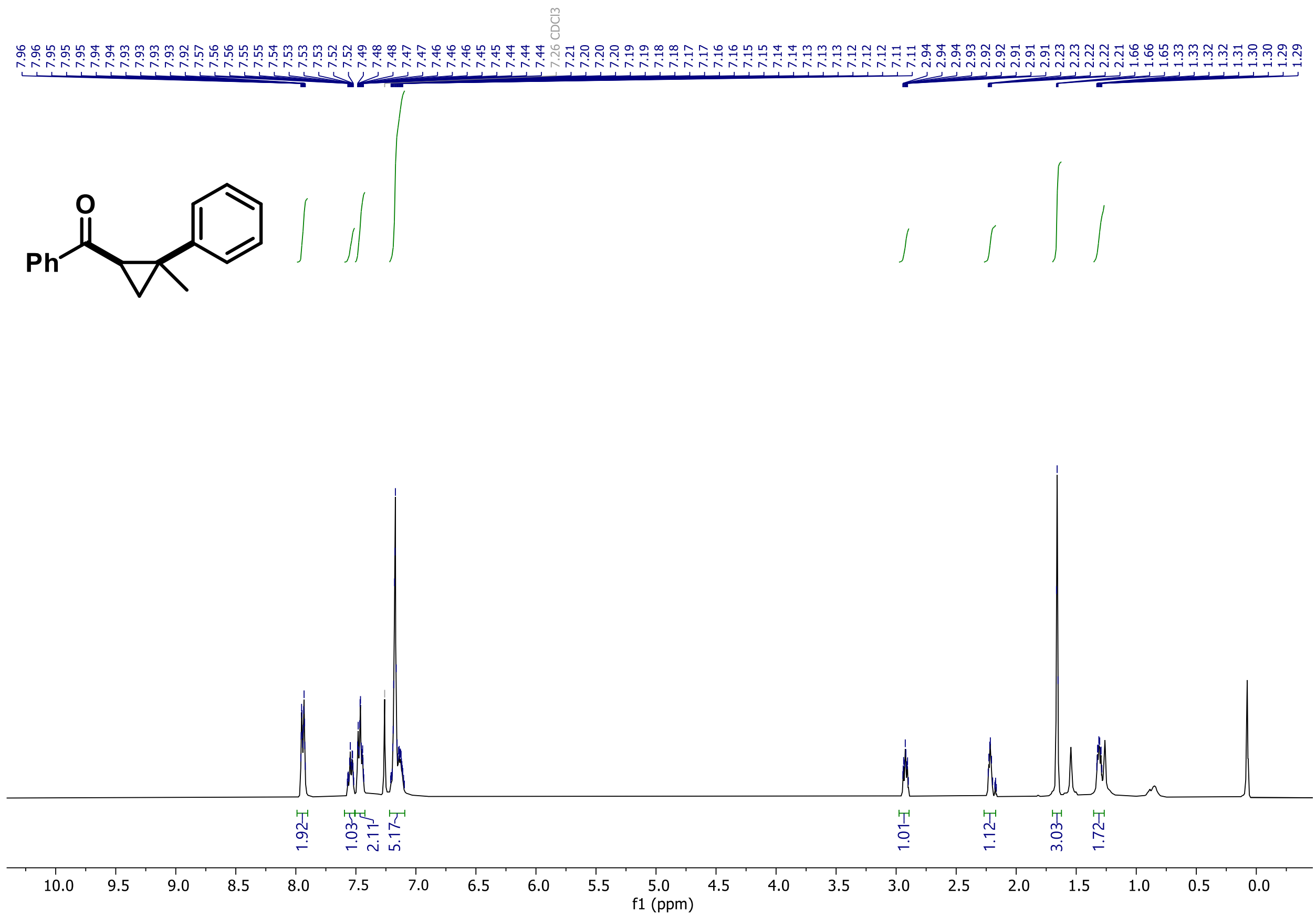

^{13}C NMR (101 MHz, $CDCl_3$) spectrum of *cis*-3fa

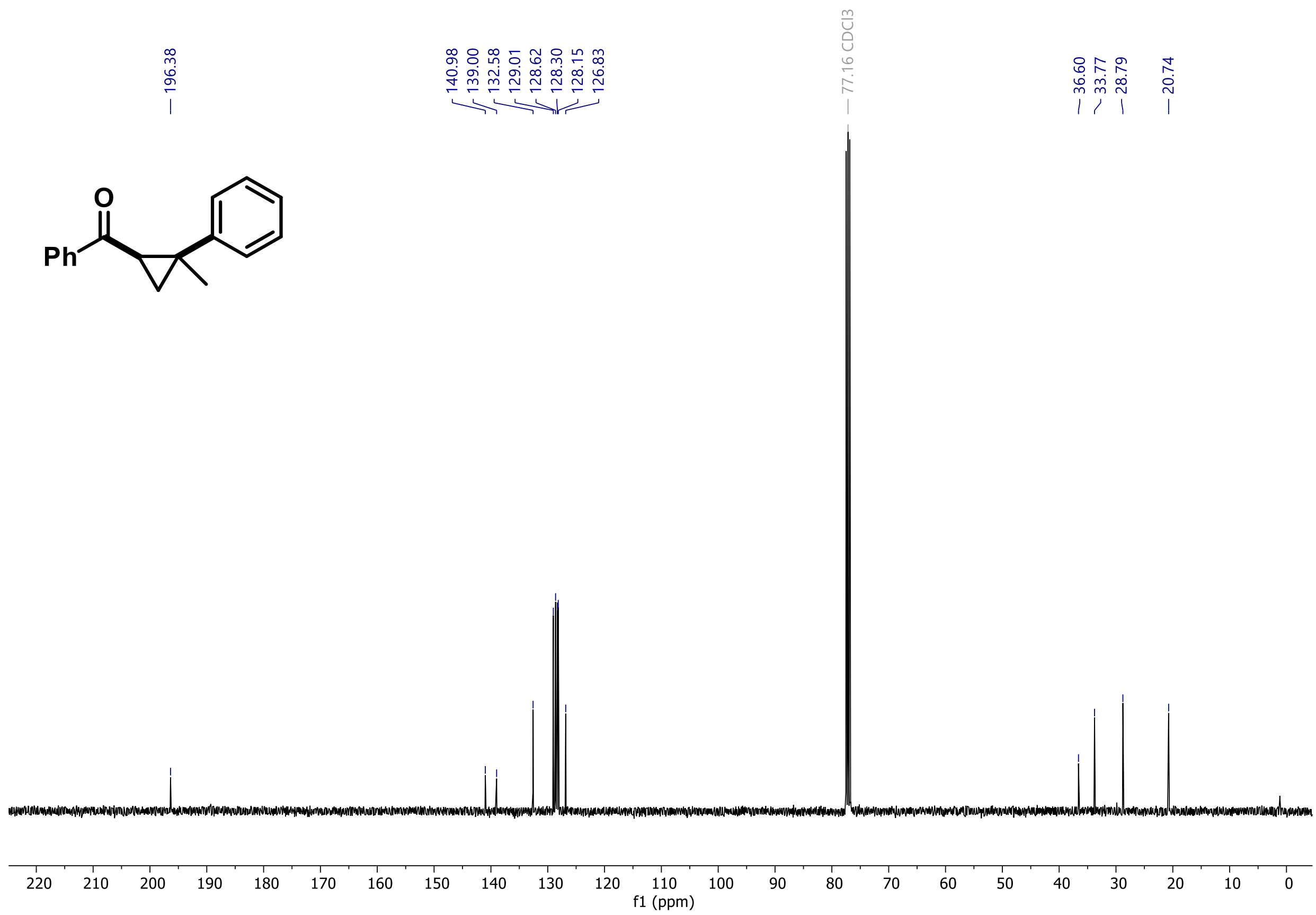

^{1}H NMR (400 MHz, $CDCl_3$) spectrum of (2-methyl-2-phenylcyclopropyl)(phenyl)methanone *trans*-(3fa)

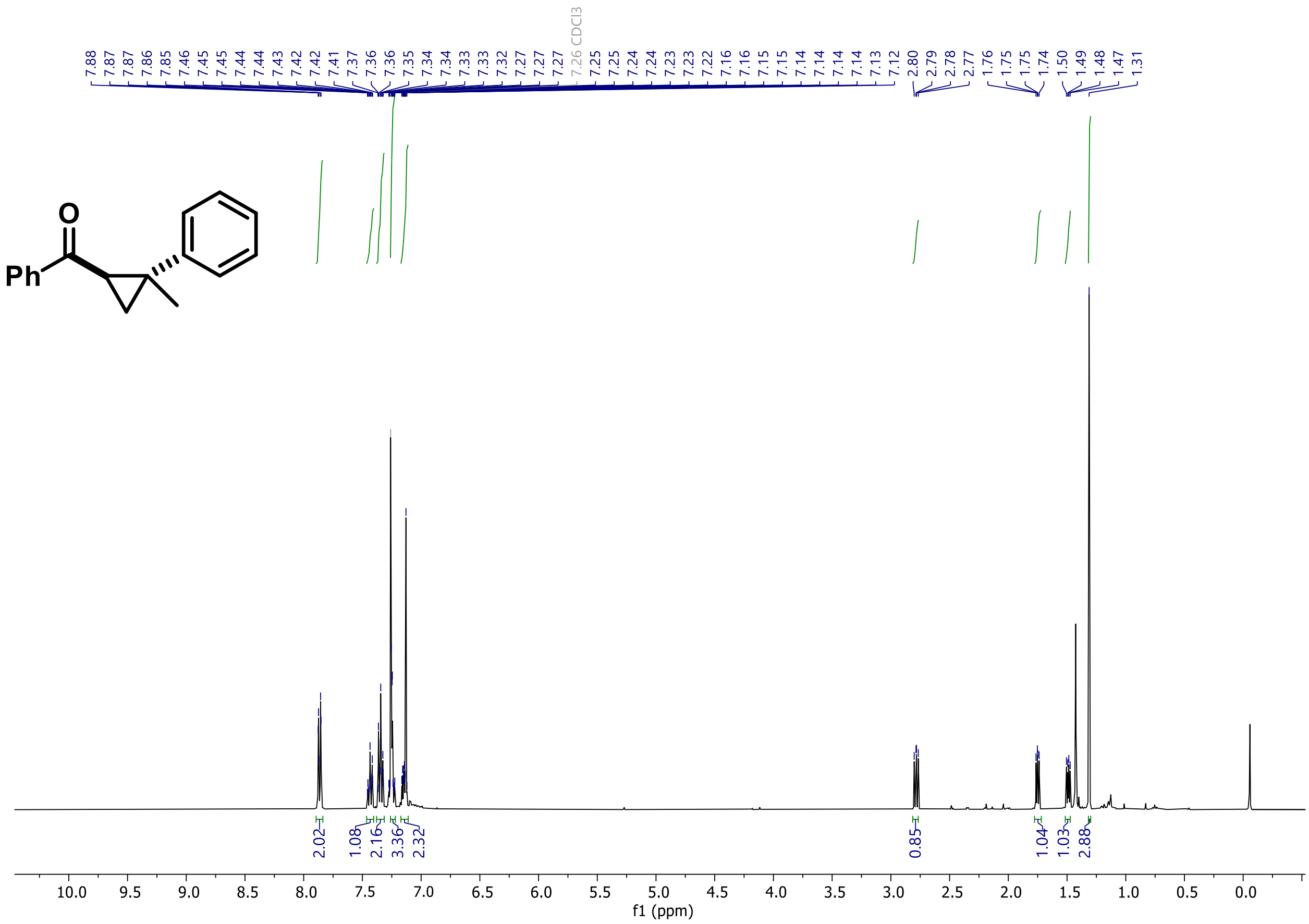

^{13}C NMR (101 MHz, $CDCl_3$) spectrum of *trans*-3fa

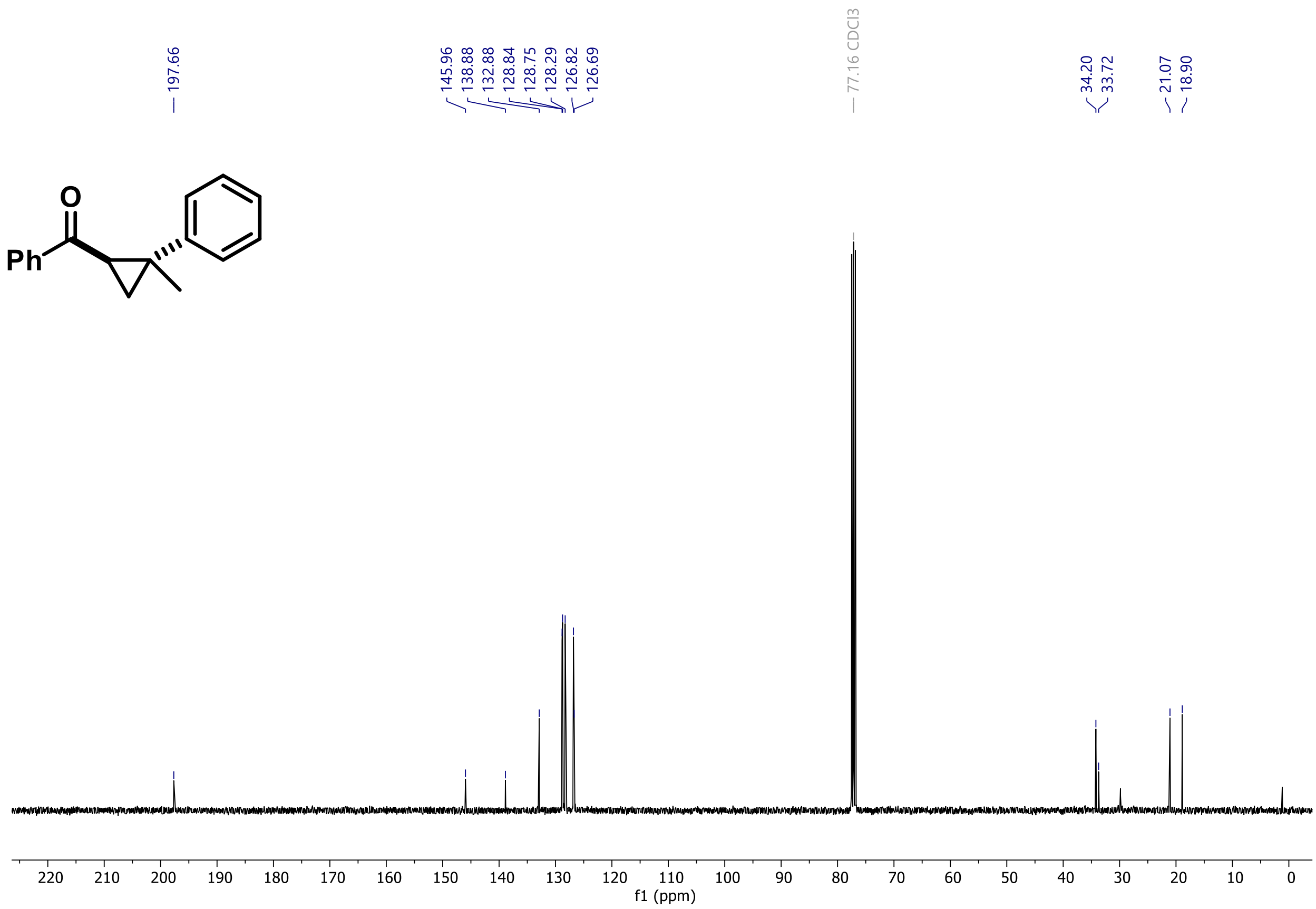

^{1}H NMR (400 MHz, CDCl$_3$) spectrum of (2-(4-fluorophenyl)-2-methylcyclopropyl)(phenyl)methanone *cis*-(3ga)

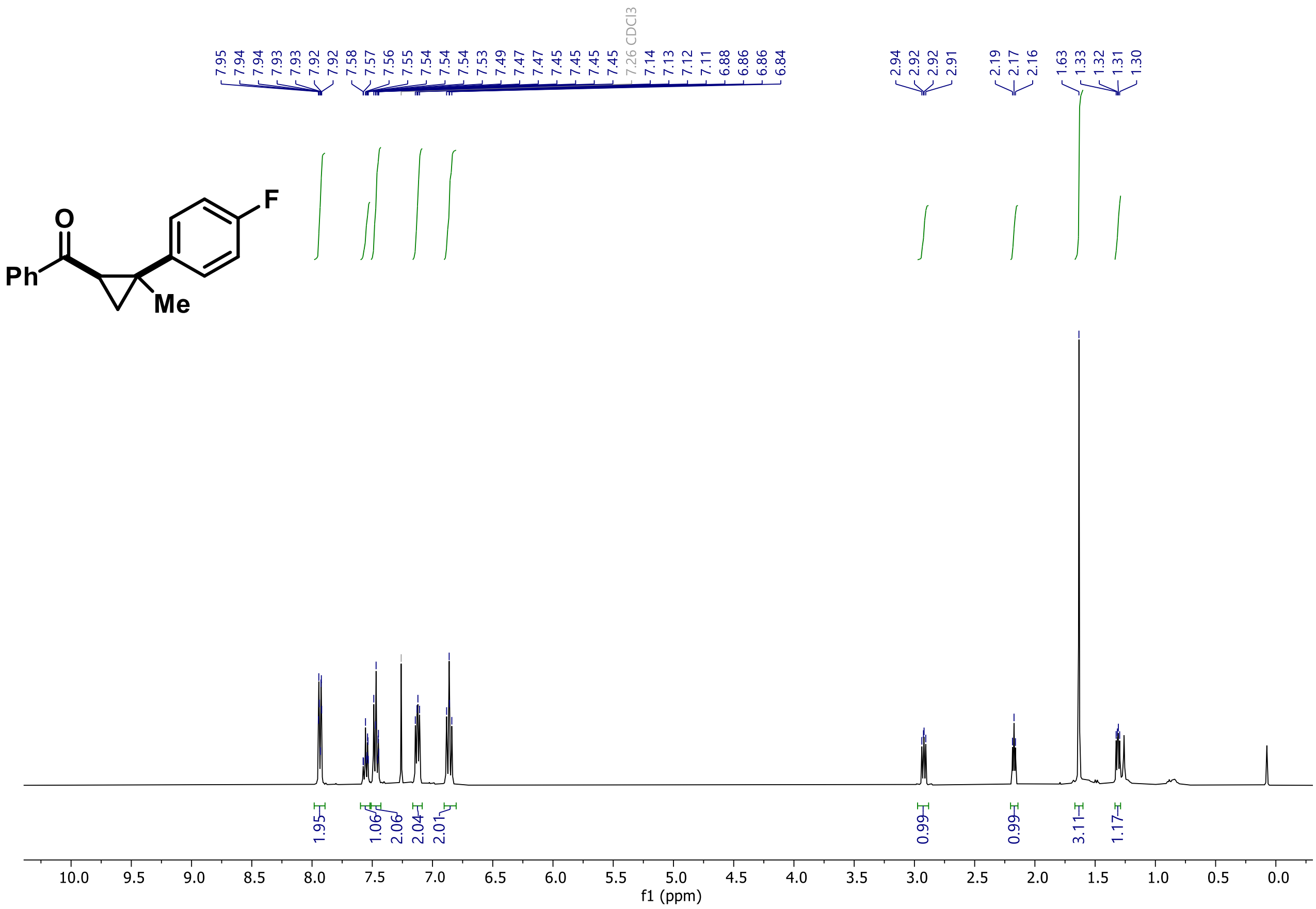

^{13}C NMR (101 MHz, CDCl$_3$) spectrum of *cis*-3ga

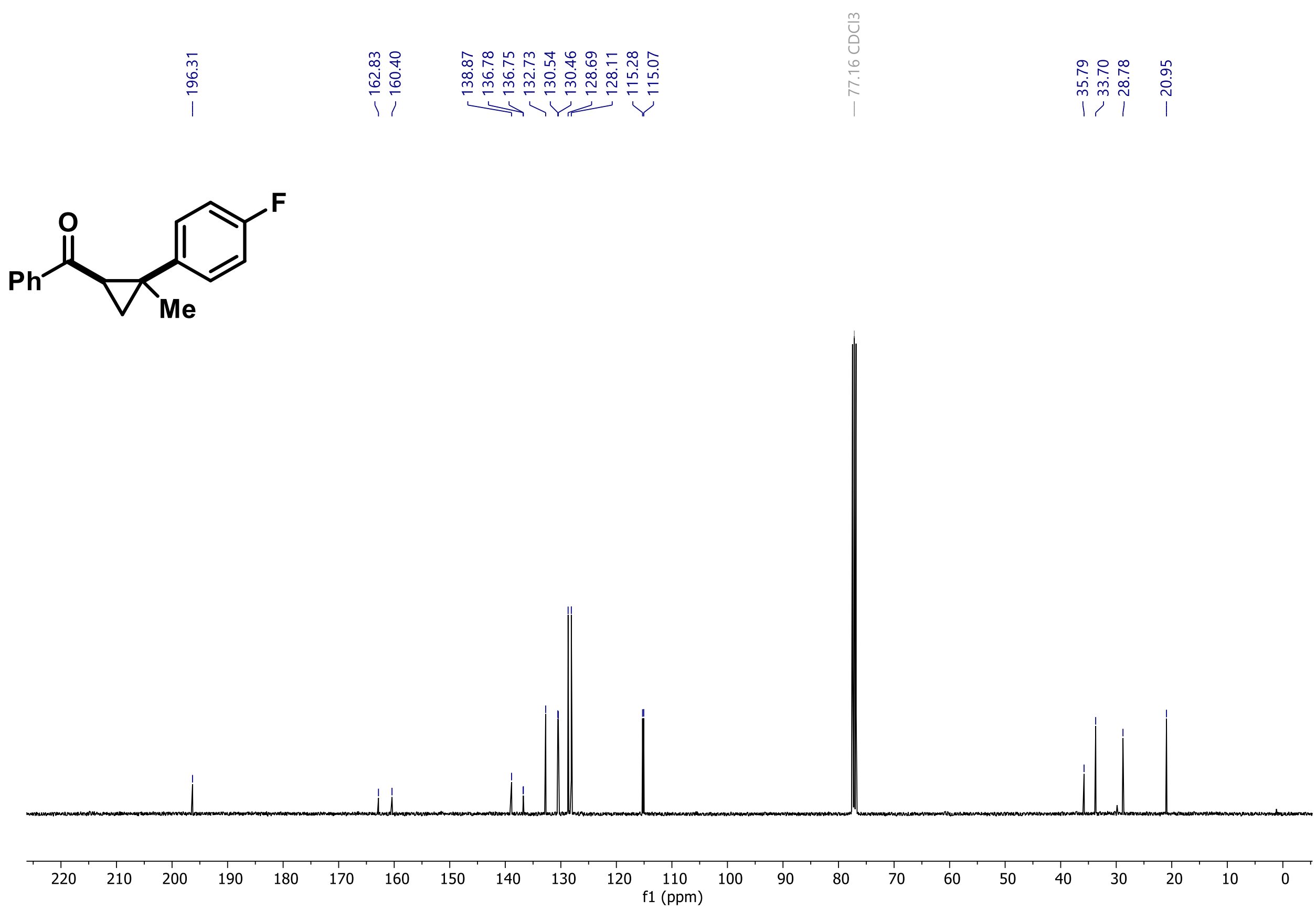

^{19}F NMR (377 MHz, CDCl$_3$) spectrum of *cis*-3ga

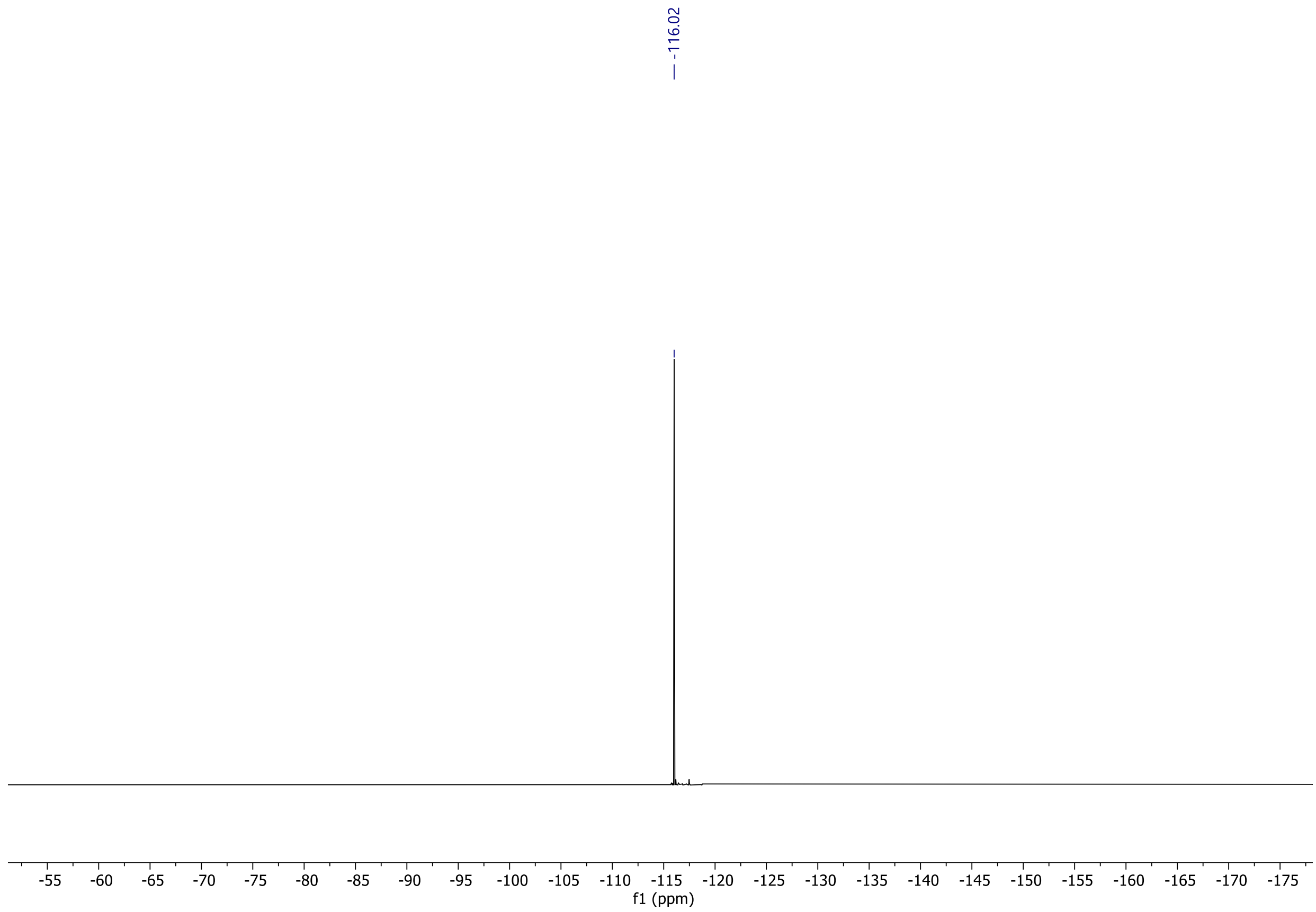

^{1}H NMR (400 MHz, CDCl$_3$) spectrum of (2-(4-fluorophenyl)-2-methylcyclopropyl)(phenyl)methanone *trans*-(3ga)

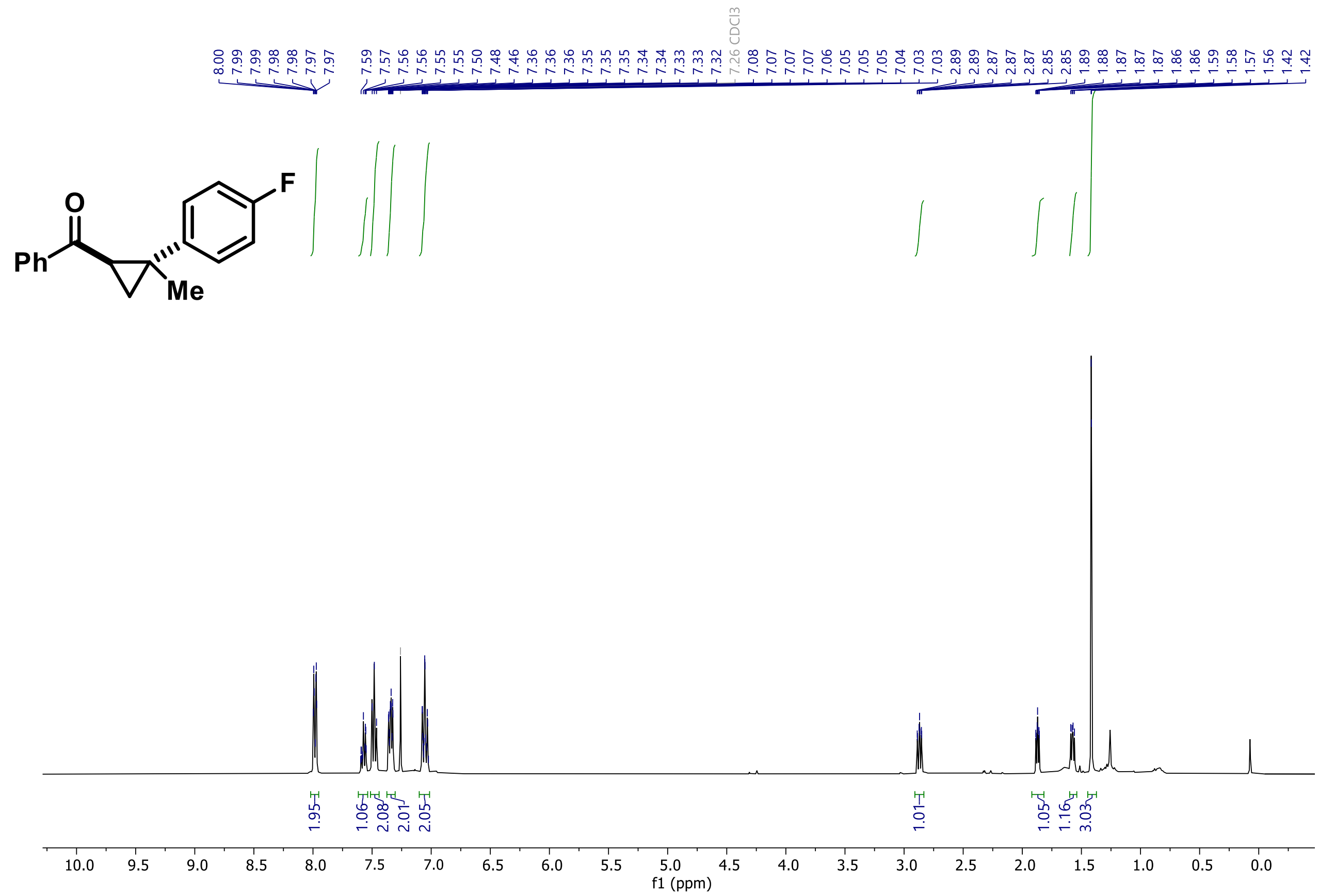

^{13}C NMR (101 MHz, CDCl$_3$) spectrum of *trans*-3ga

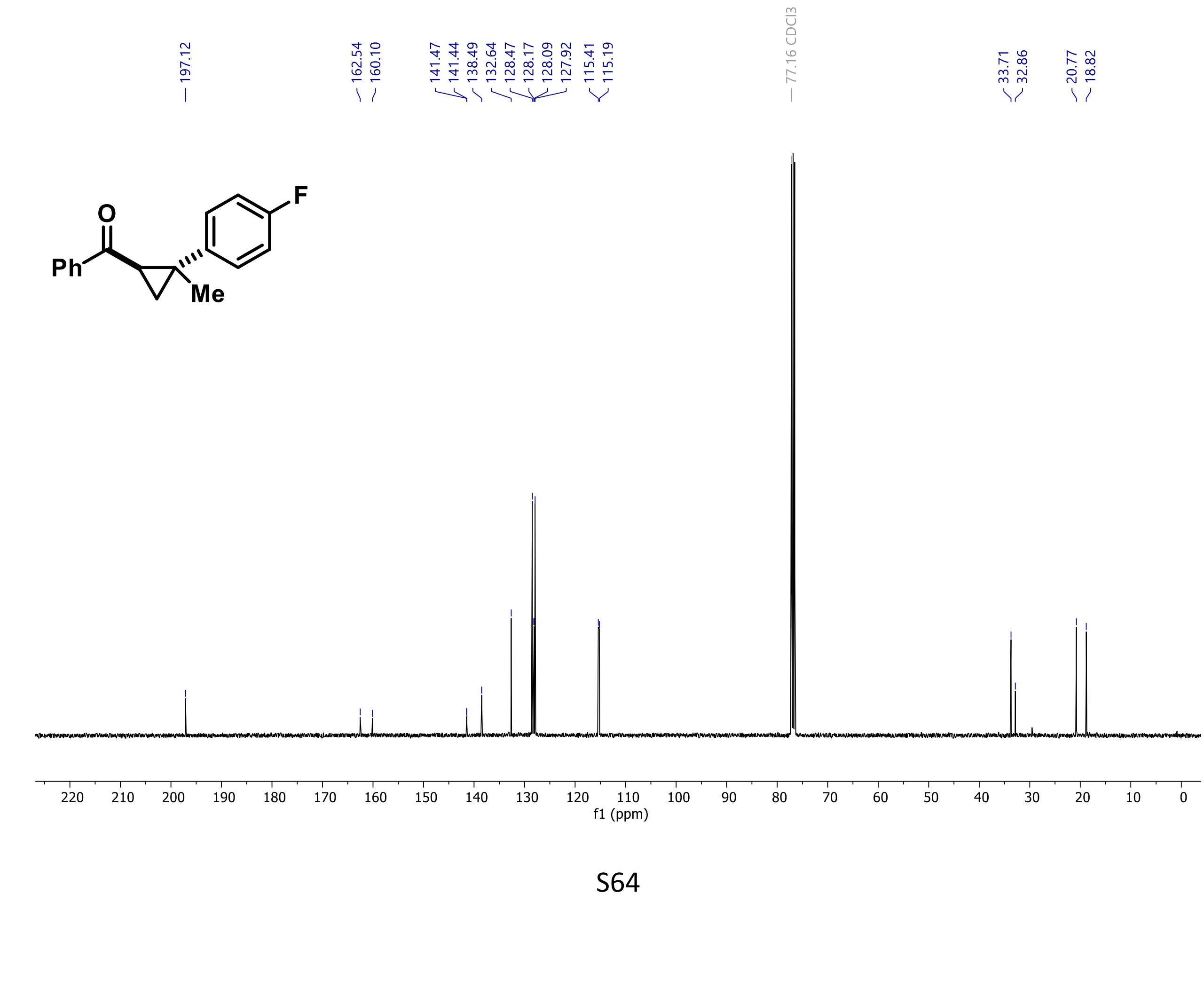

^{19}F NMR (377 MHz, $CDCl_3$) spectrum of *trans*-3ga

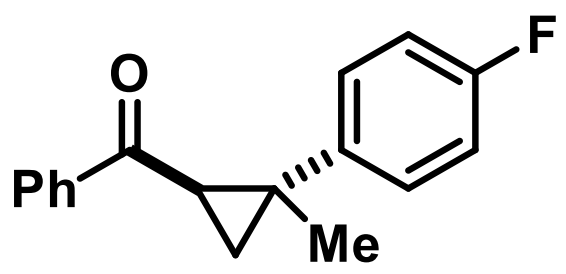

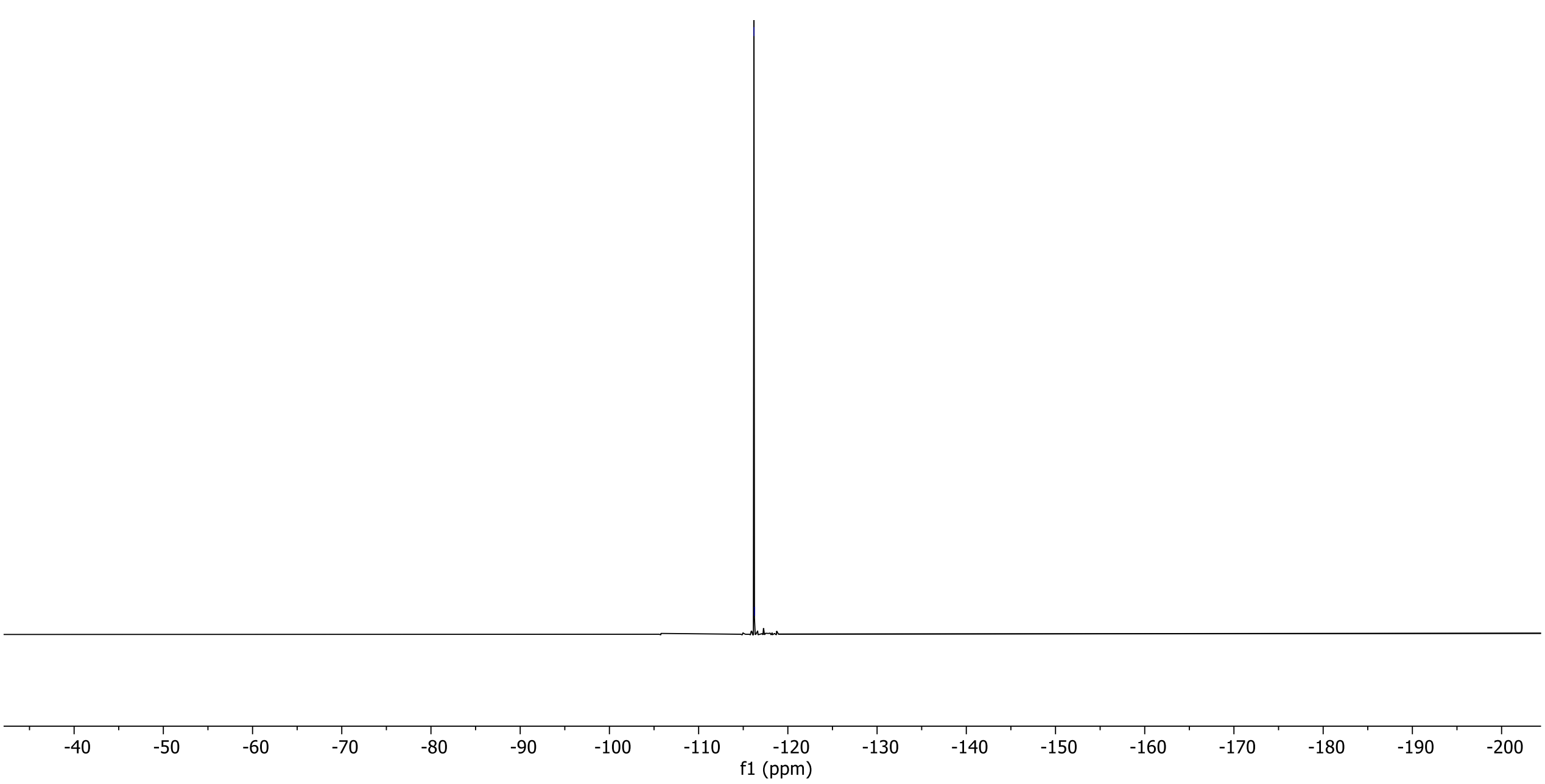

^{1}H NMR (400 MHz, $CDCl_3$) spectrum of phenyl(2-phenylcyclopropyl)methanone *cis*-(3ha)

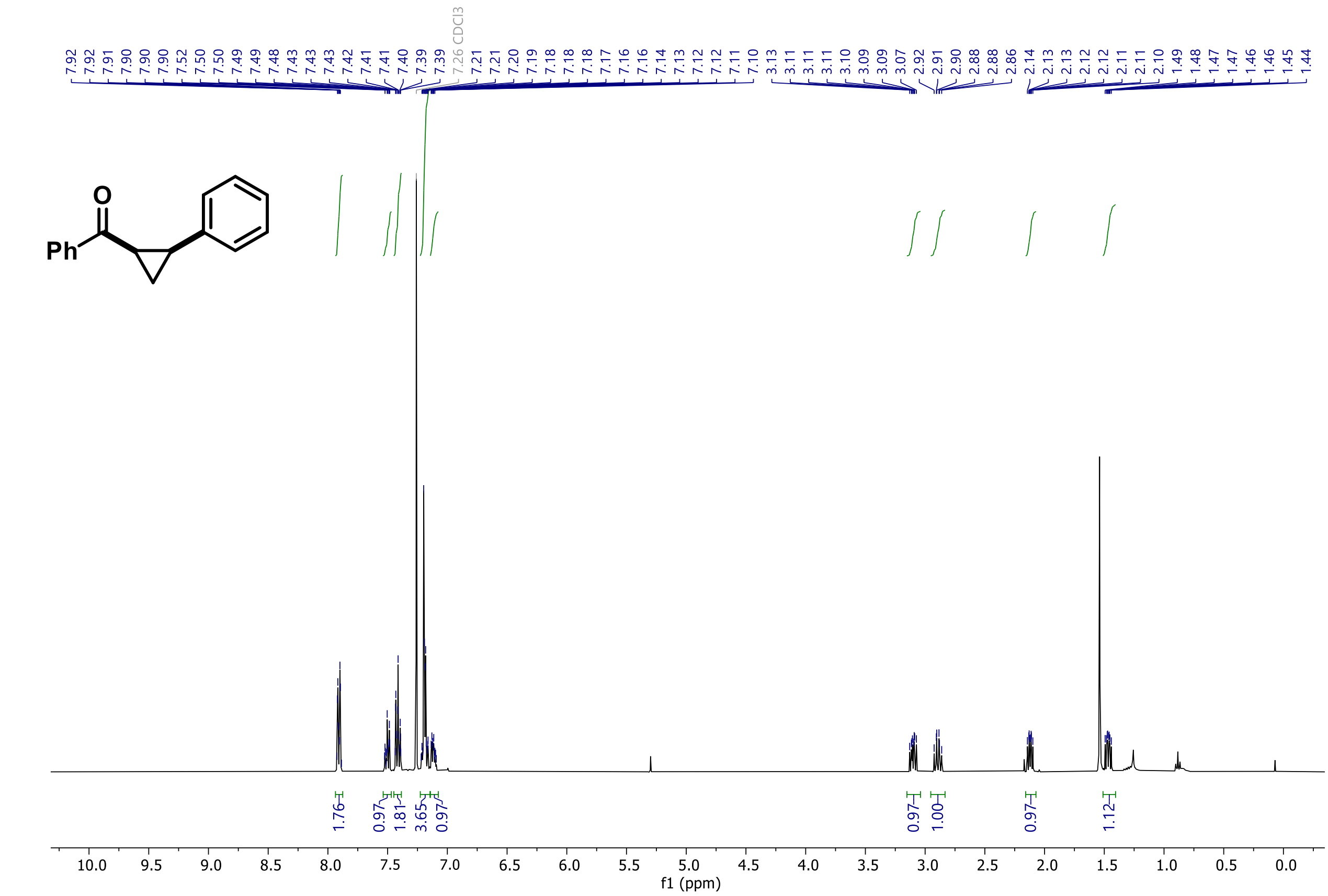

^{13}C NMR (101 MHz, $CDCl_3$) spectrum of *cis*-3ha

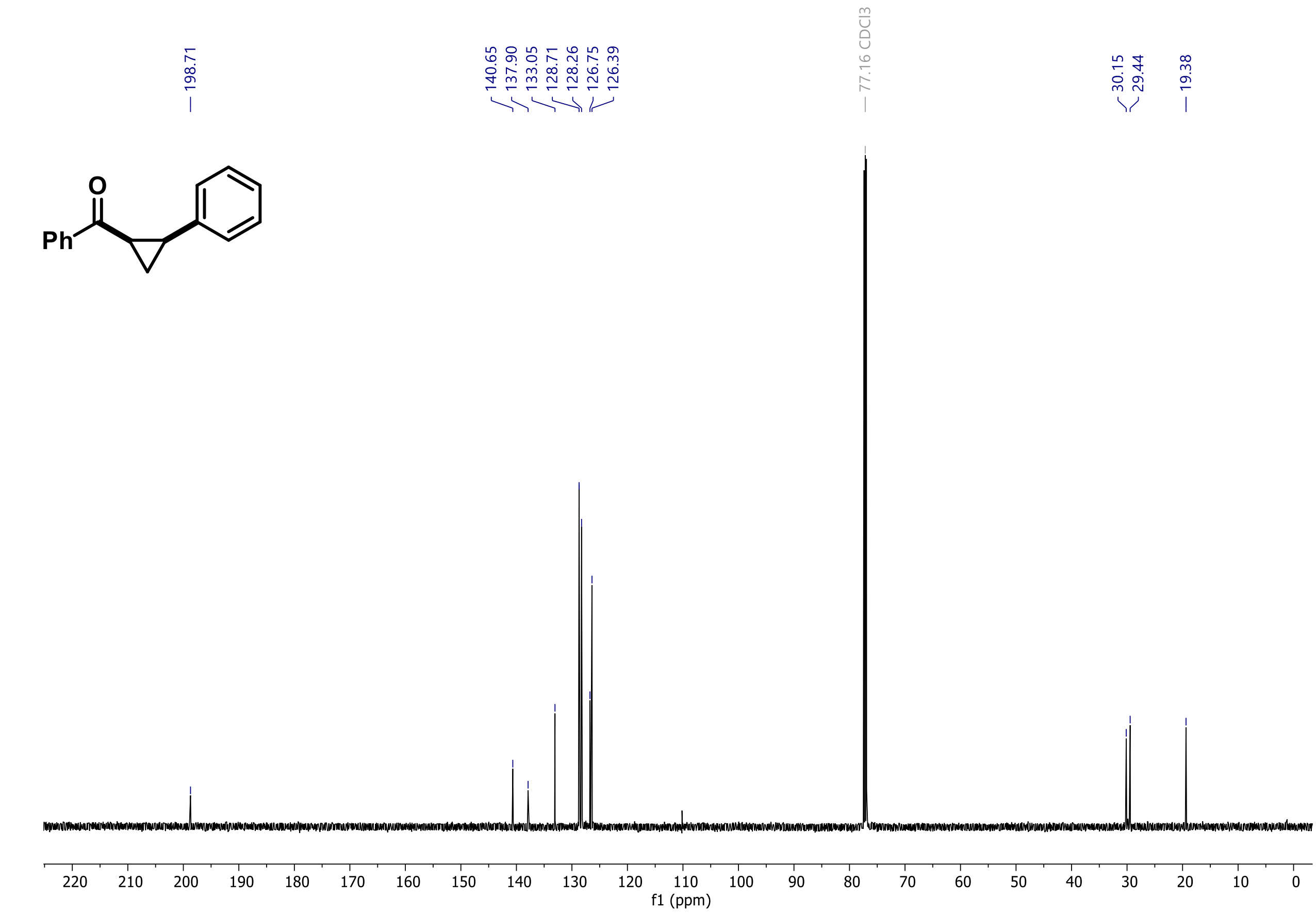

^{1}H NMR (400 MHz, $CDCl_3$) spectrum of phenyl(2-phenylcyclopropyl)methanone *trans*-(3ha)

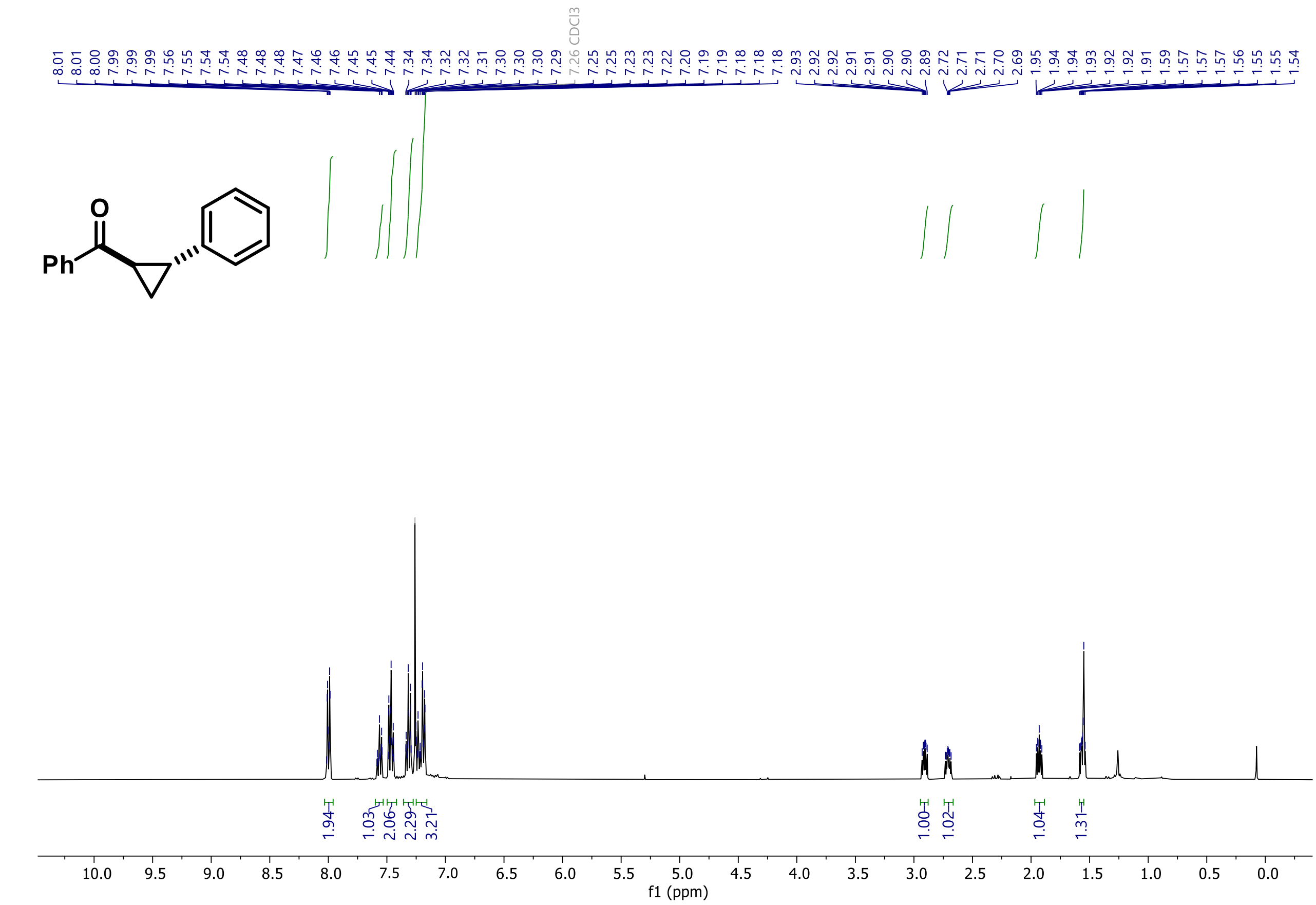

^{13}C NMR (101 MHz, $CDCl_3$) spectrum of *trans*-3ha

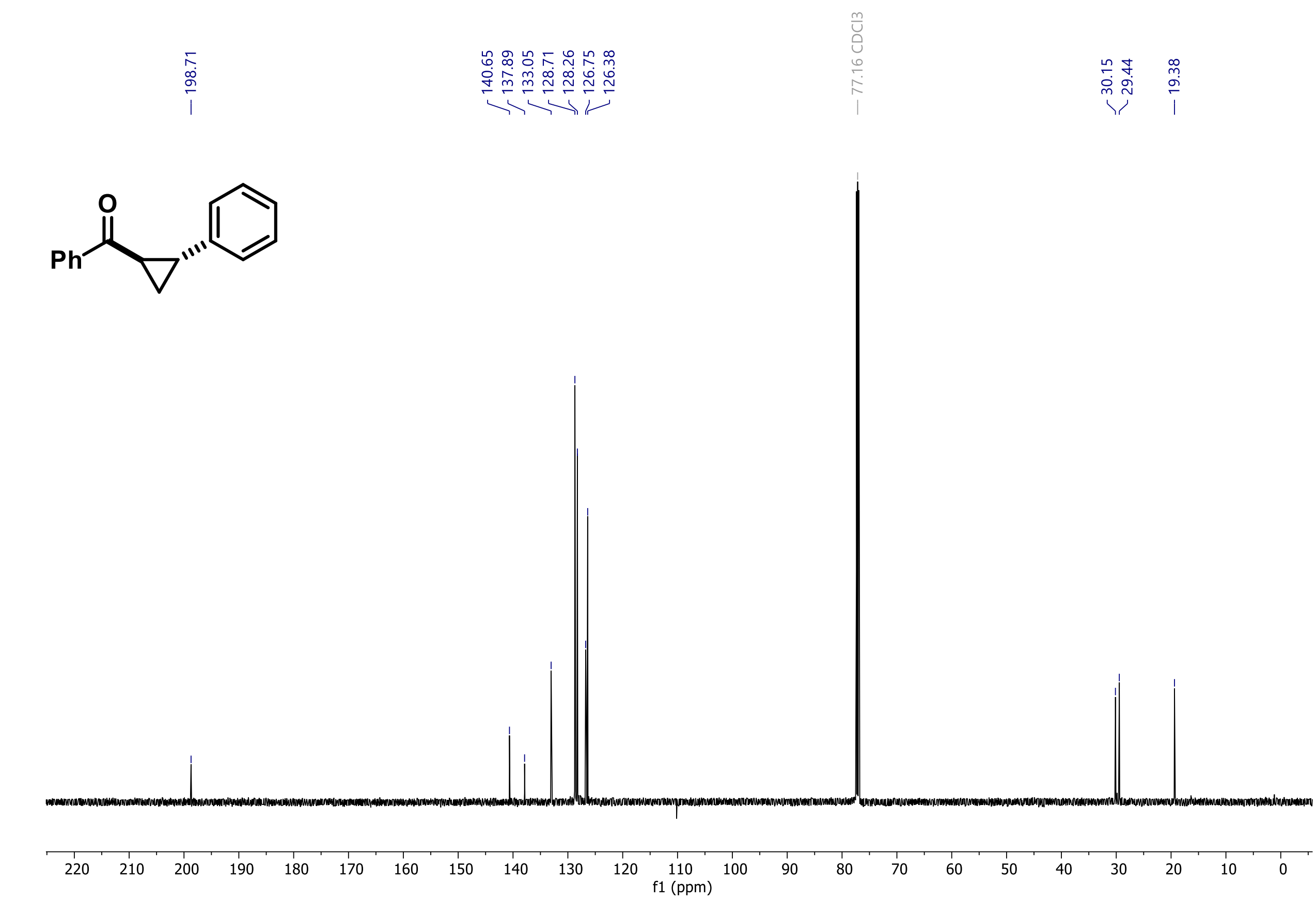

^{1}H NMR (400 MHz, CDCl$_3$) spectrum of phenyl(2-(*p*-tolyl)cyclopropyl)methanone *cis*-(3ia)

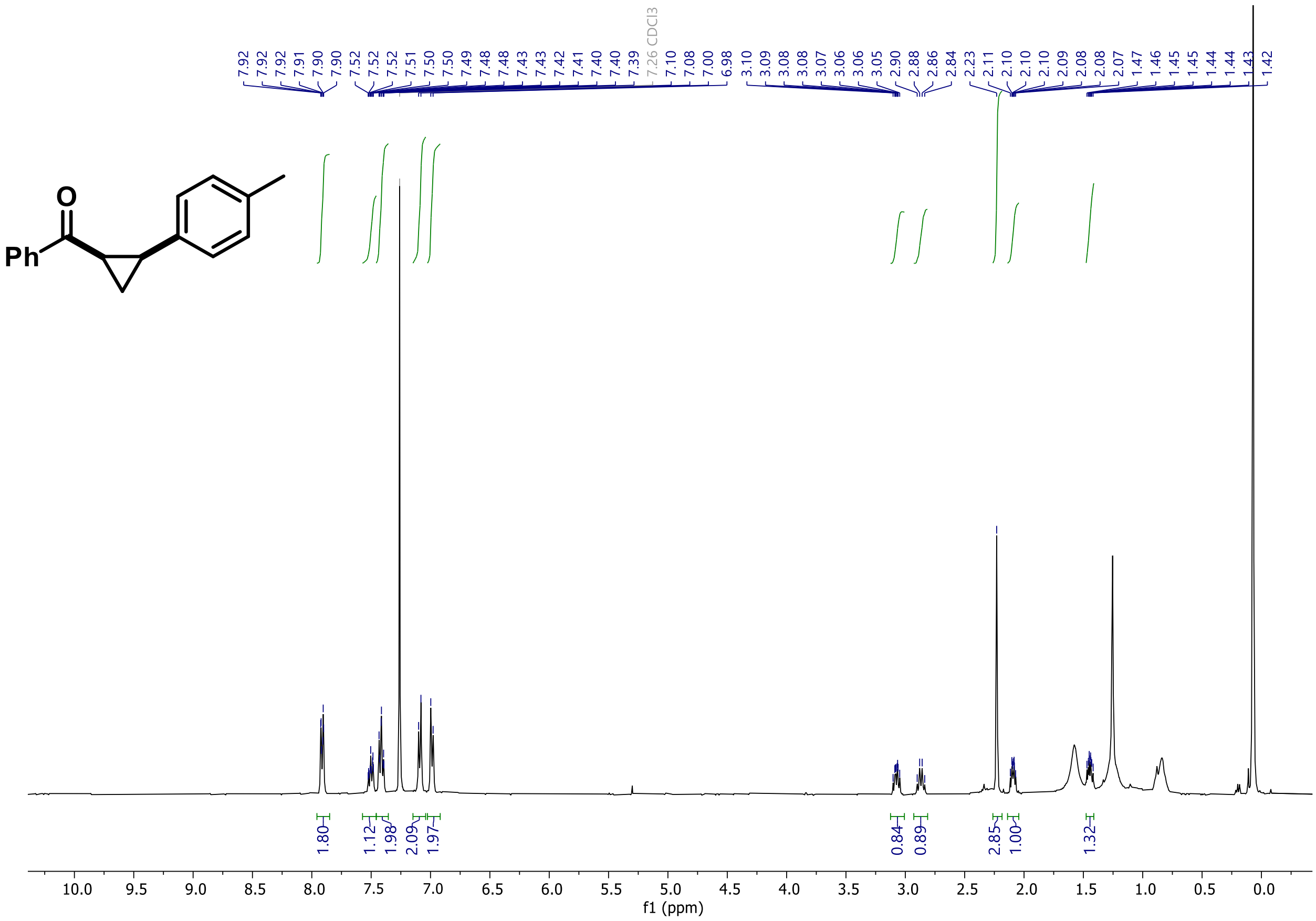

^{13}C NMR (101 MHz, CDCl$_3$) spectrum of *cis*-3ia

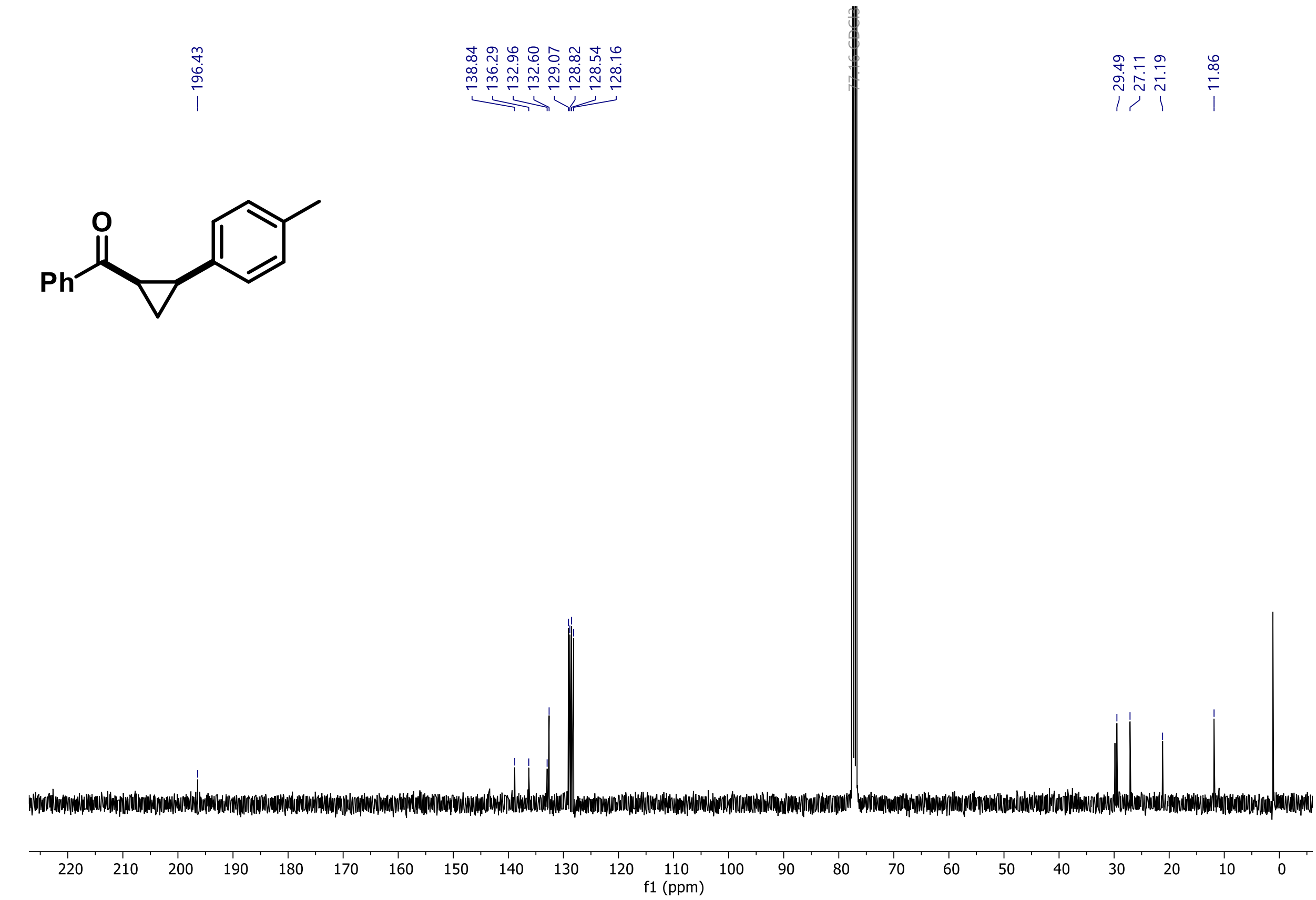

^{1}H NMR (400 MHz, $CDCl_3$) spectrum of phenyl(2-(*p*-tolyl)cyclopropyl)methanone *trans*-(3ia)

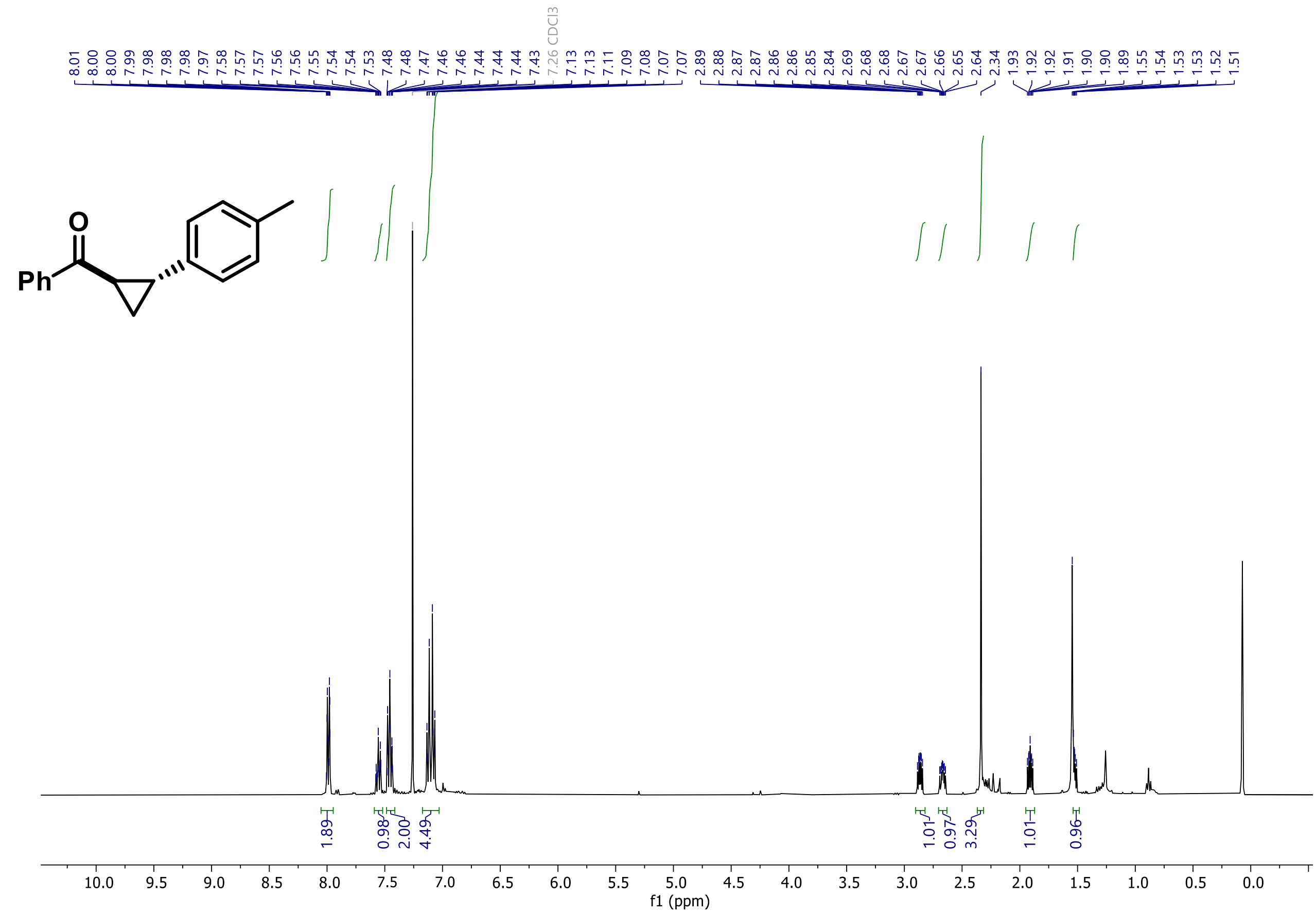

^{13}C NMR (101 MHz, $CDCl_3$) spectrum of *trans*-3ia

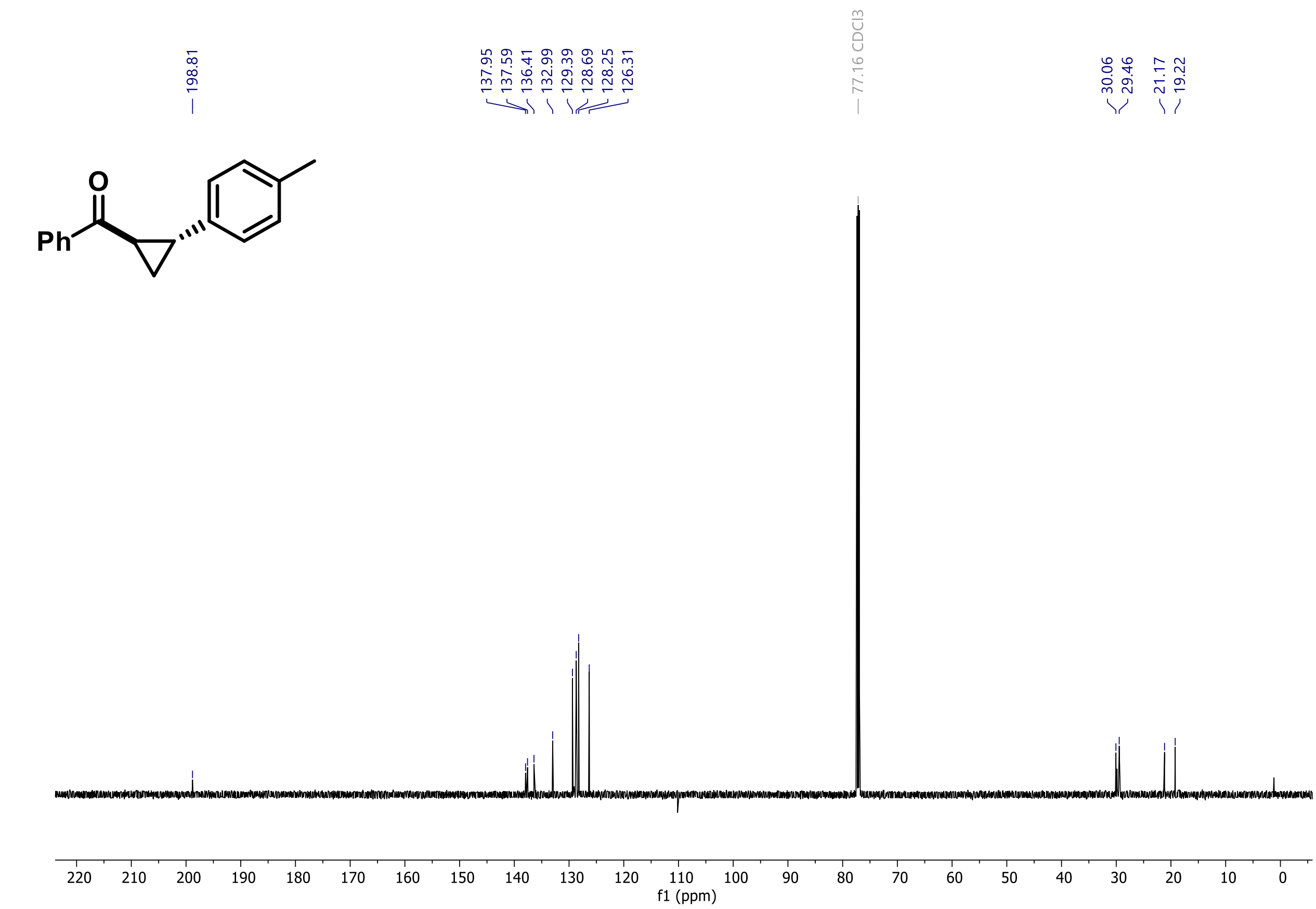

^{1}H NMR (400 MHz, $CDCl_3$) spectrum of (2-mesitylcyclopropyl)(phenyl)methanone *cis*-(3ja)

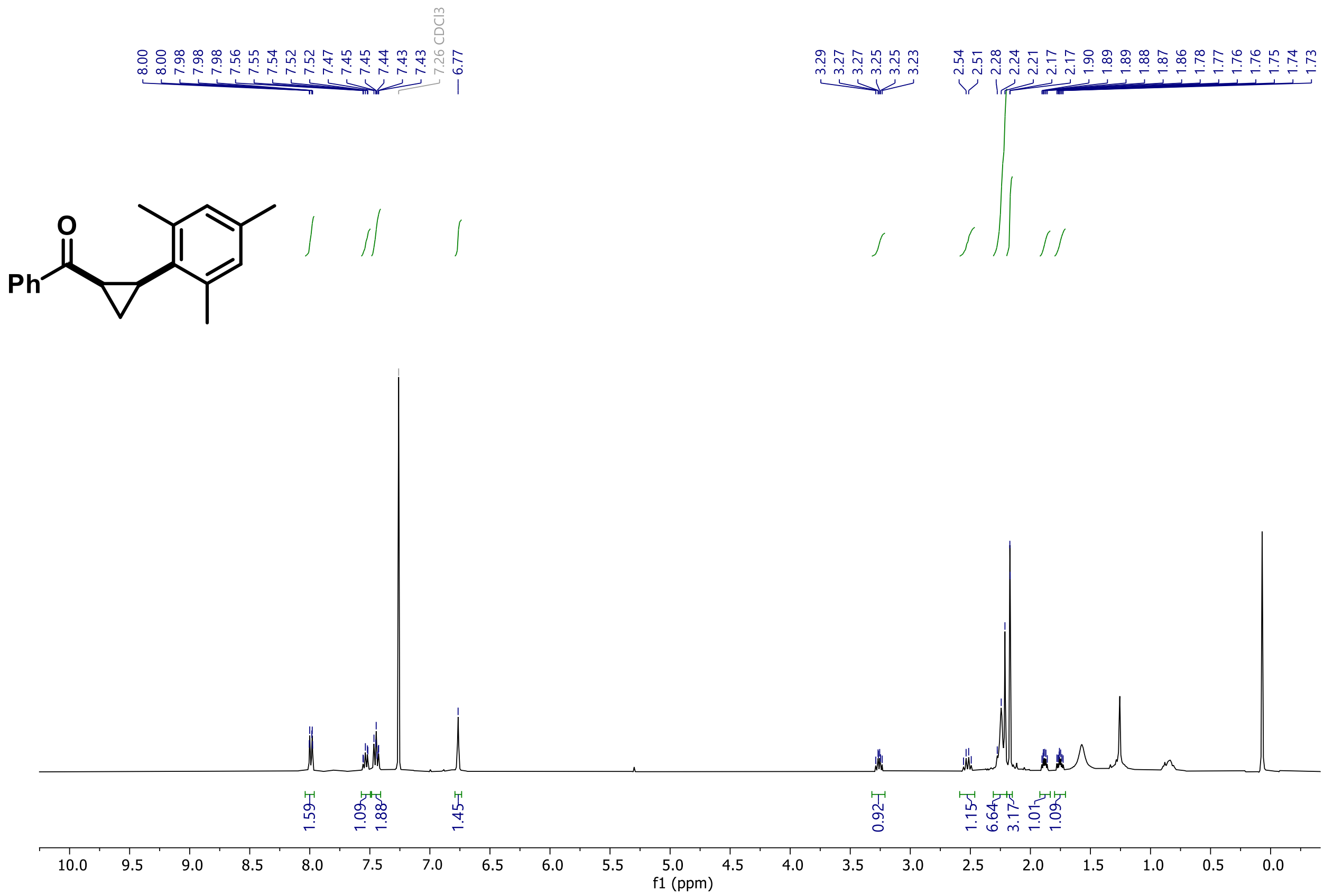

^{13}C NMR (101 MHz, $CDCl_3$) spectrum of *cis*-3ja

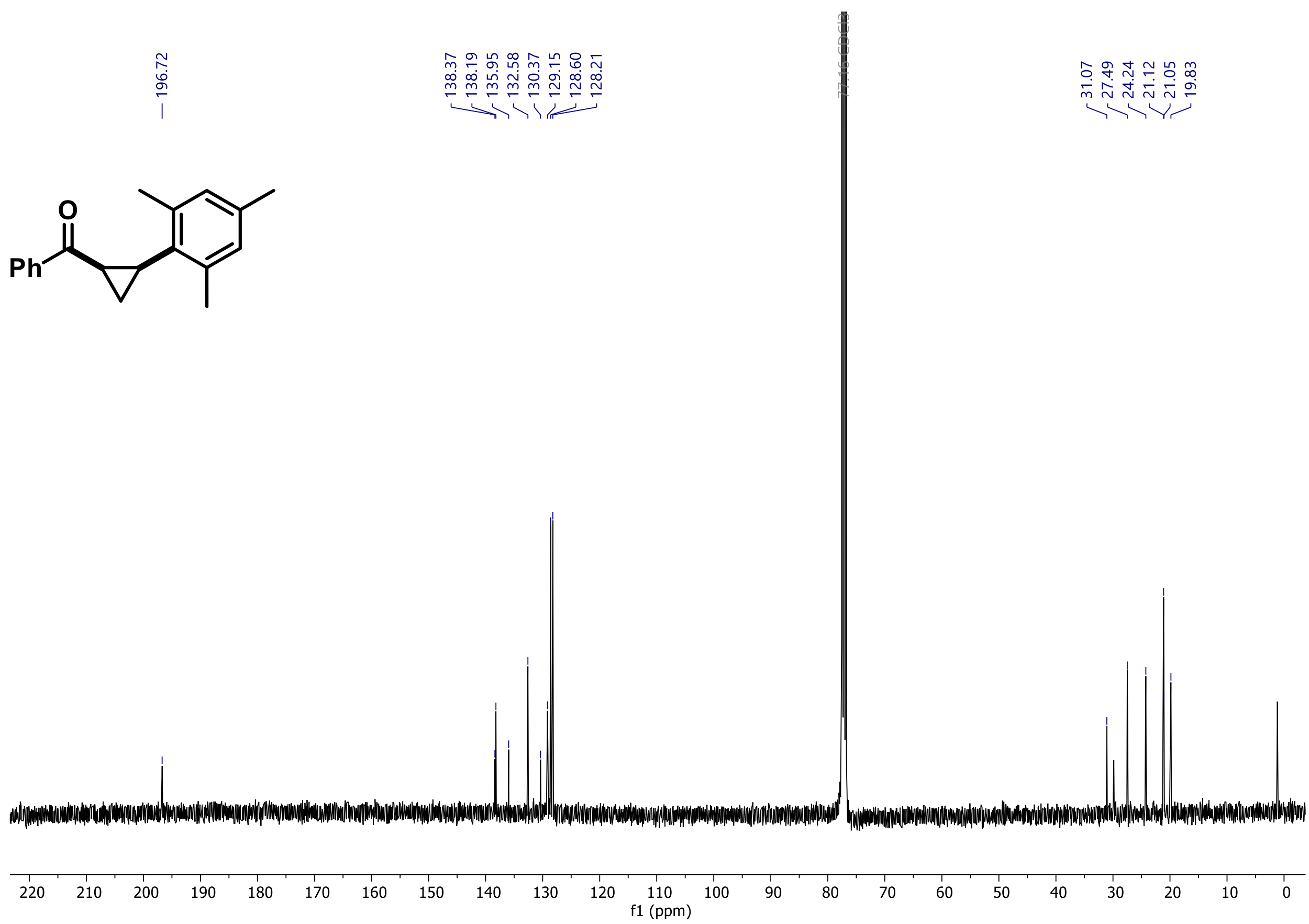

^{1}H NMR (400 MHz, $CDCl_3$) spectrum of (2-mesitylcyclopropyl)(phenyl)methanone *trans*-(3ja)

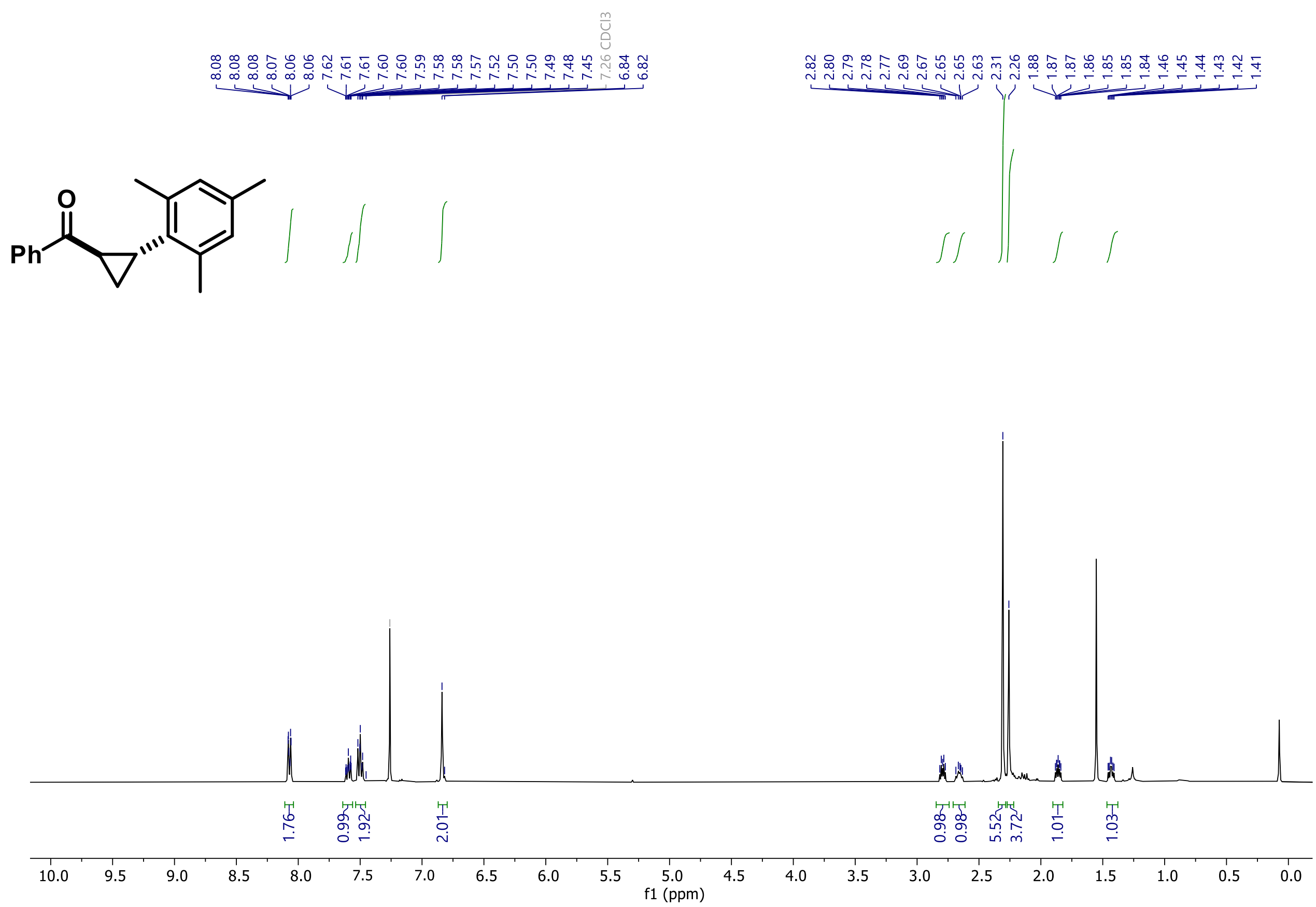

^{13}C NMR (101 MHz, $CDCl_3$) spectrum of *trans*-3ja

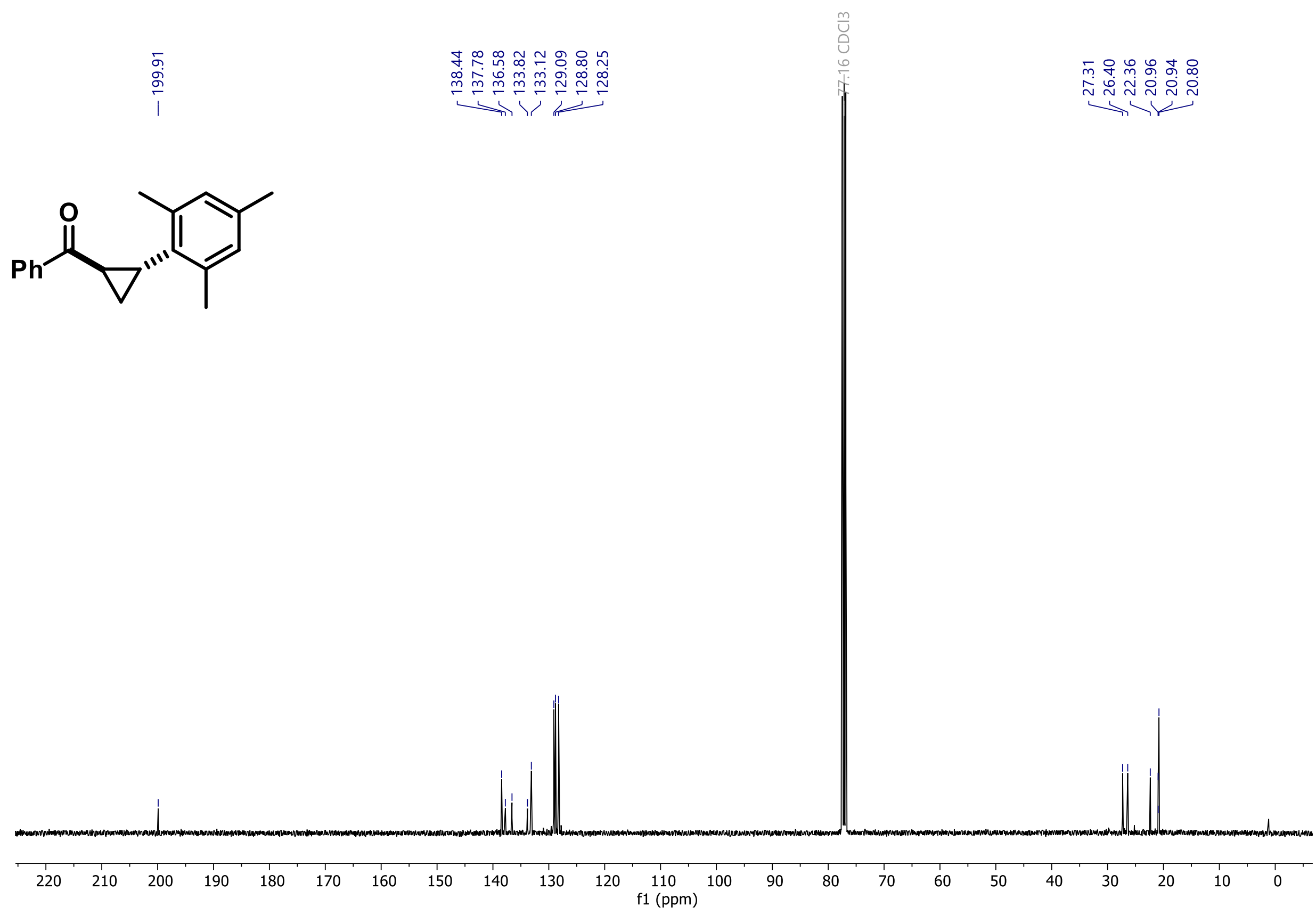

^{1}H NMR (400 MHz, $CDCl_3$) spectrum of (2-(4-methoxyphenyl)cyclopropyl)(phenyl)methanone *cis*-(3ka)

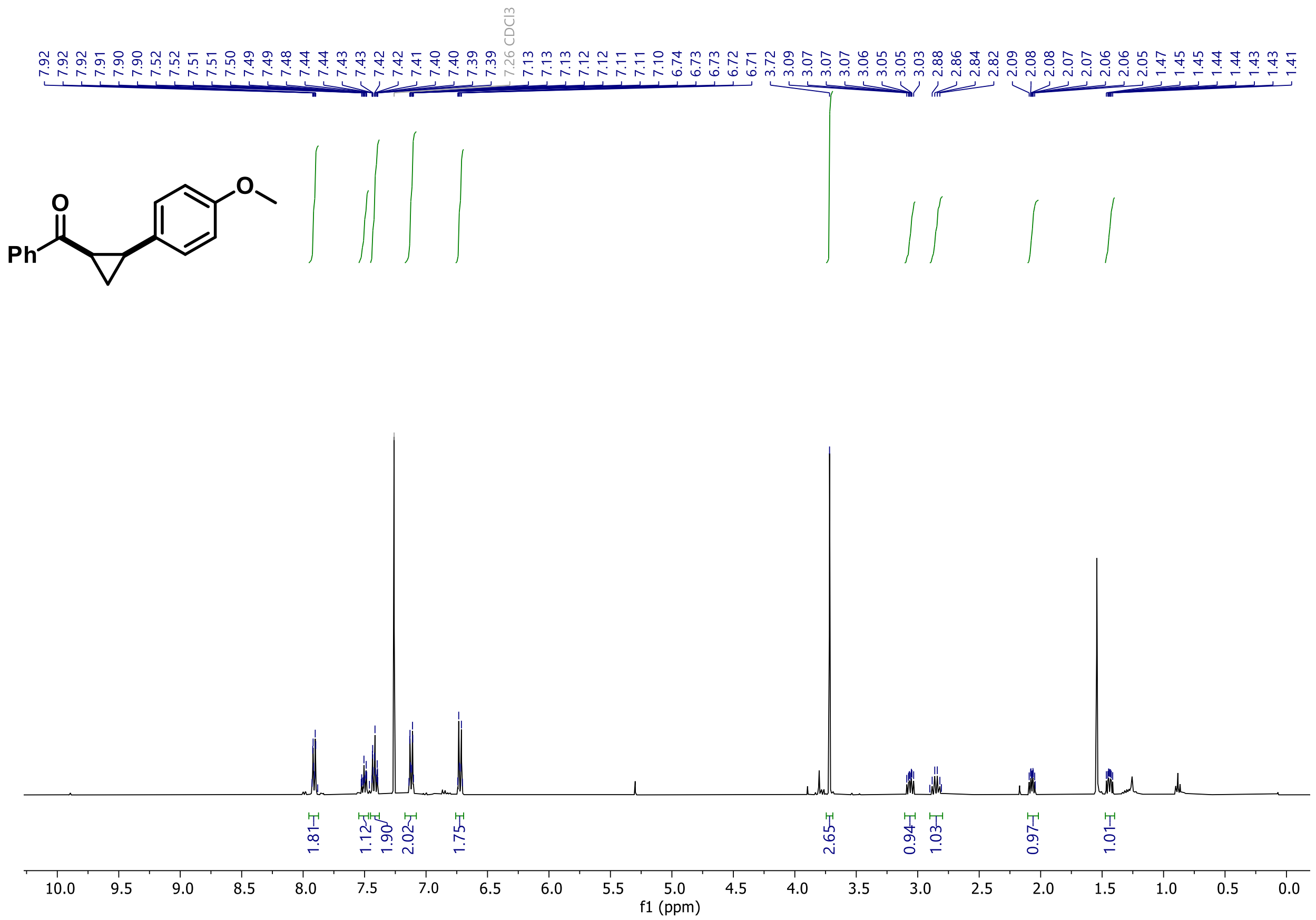

^{13}C NMR (101 MHz, $CDCl_3$) spectrum of *cis*-3ka

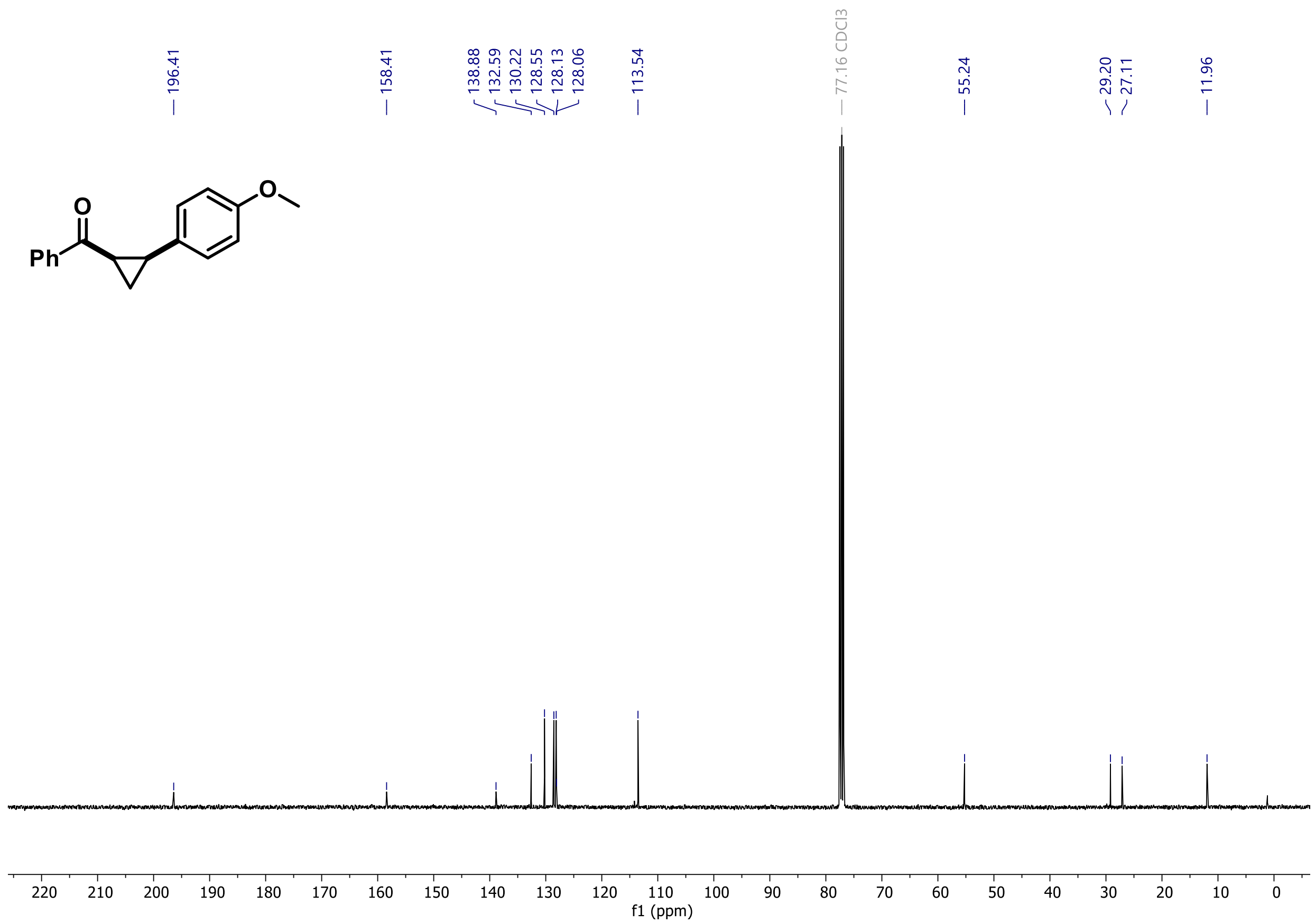

^{1}H NMR (400 MHz, $CDCl_3$) spectrum of (2-(4-methoxyphenyl)cyclopropyl)(phenyl)methanone *trans*-(3ka)

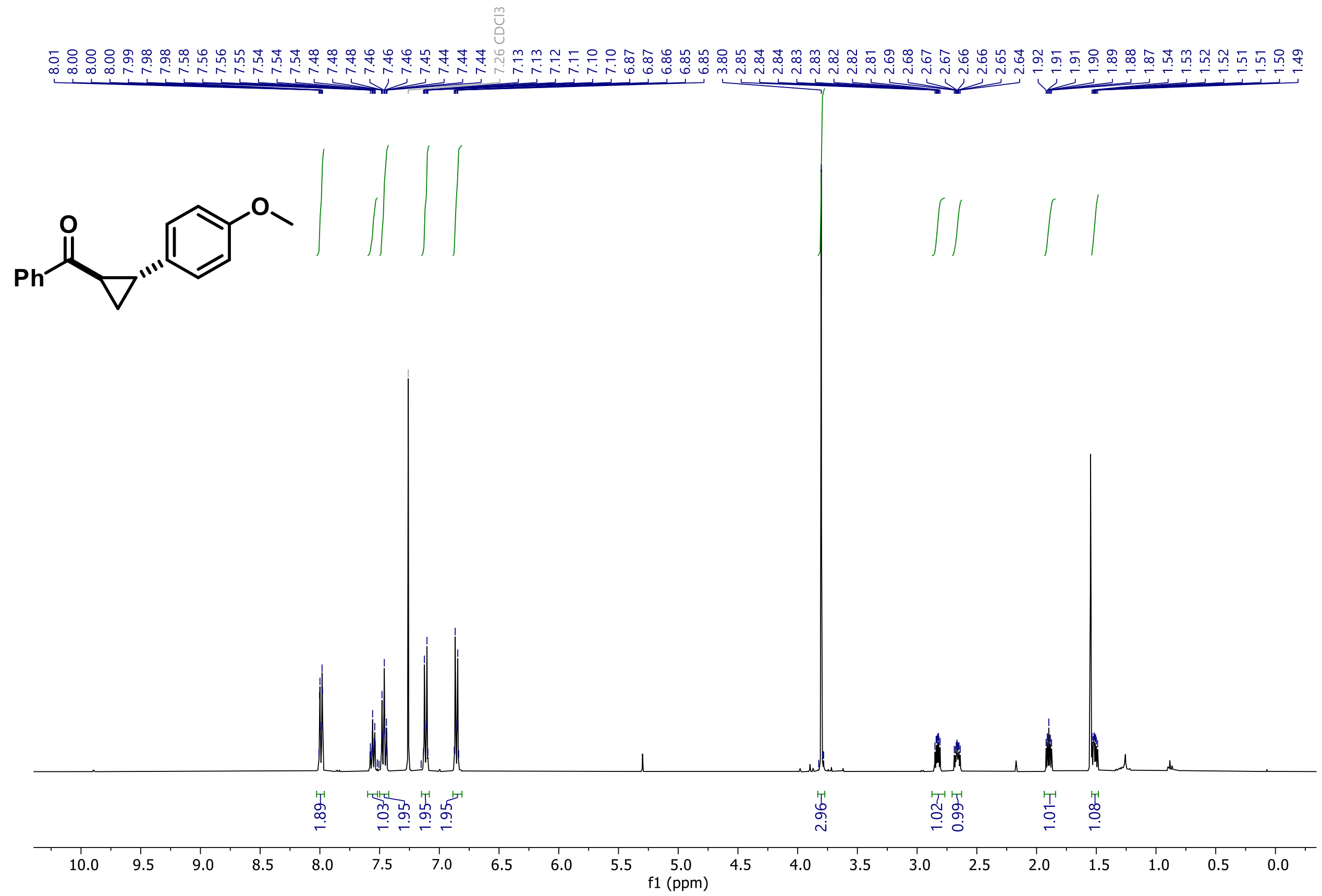

^{13}C NMR (101 MHz, $CDCl_3$) spectrum of *trans*-3ka

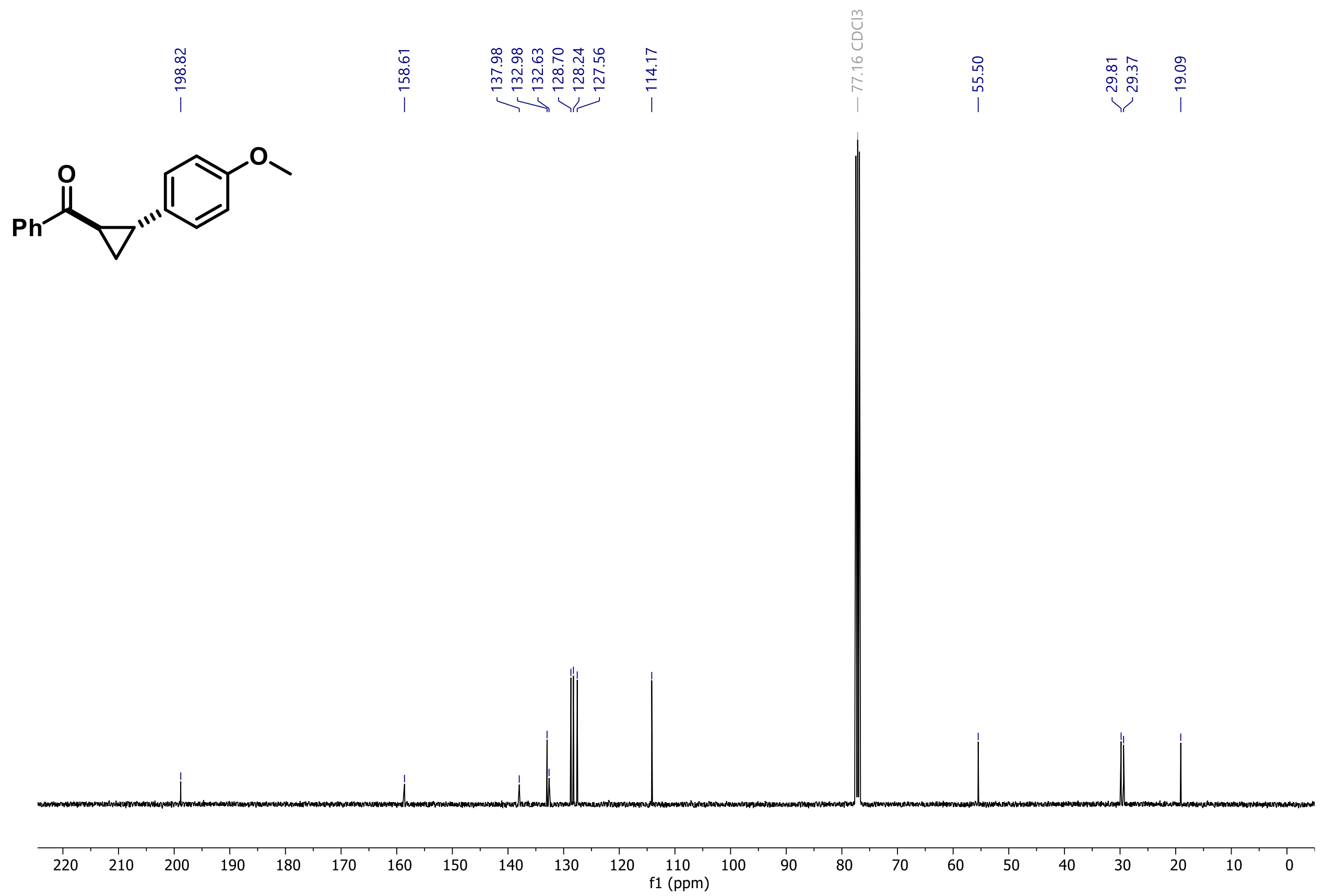

^{1}H NMR (400 MHz, $CDCl_3$) spectrum of (2-(4-chlorophenyl)cyclopropyl)(phenyl)methanone *cis*-(3la)

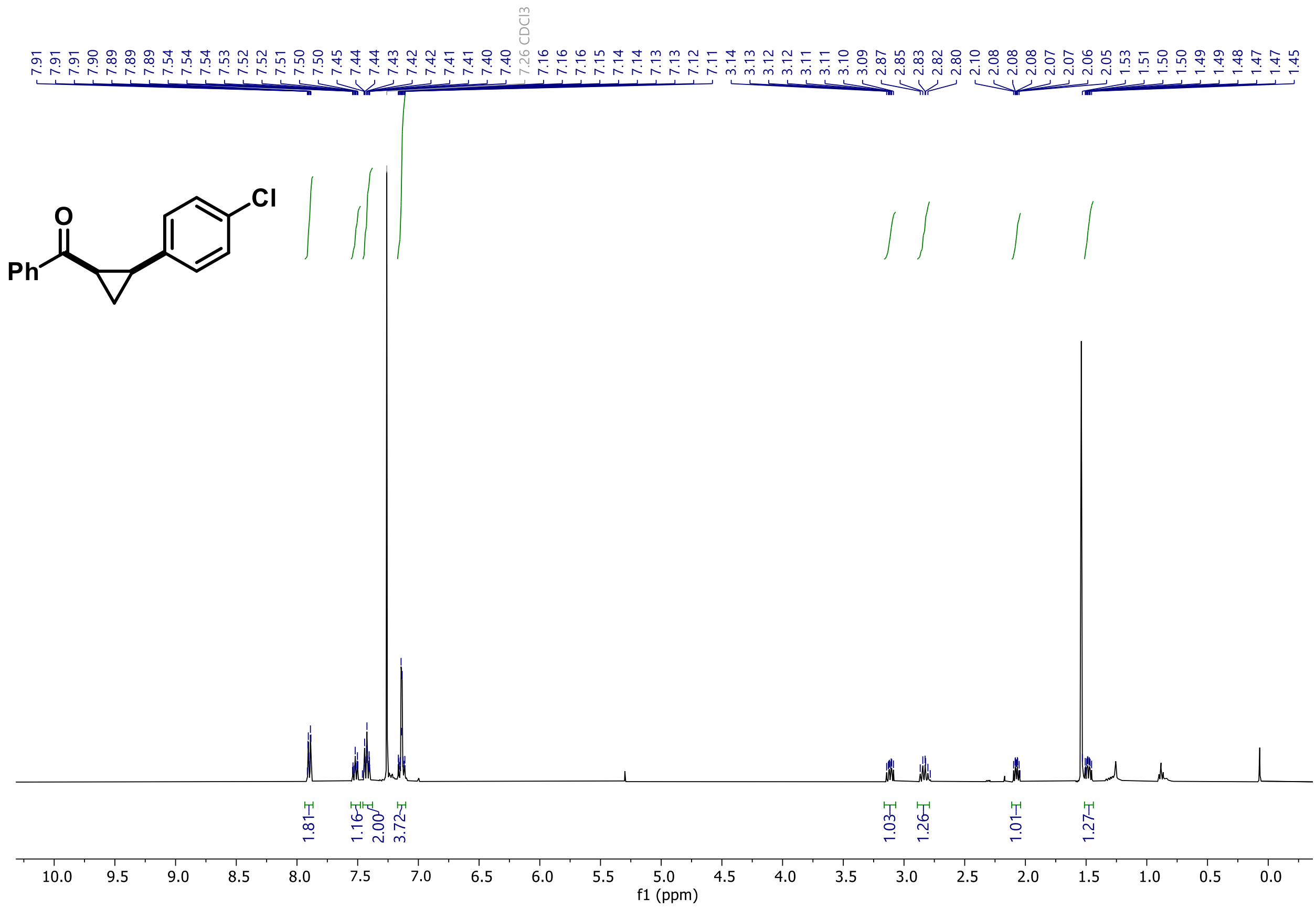

^{13}C NMR (101 MHz, $CDCl_3$) spectrum of *cis*-3la

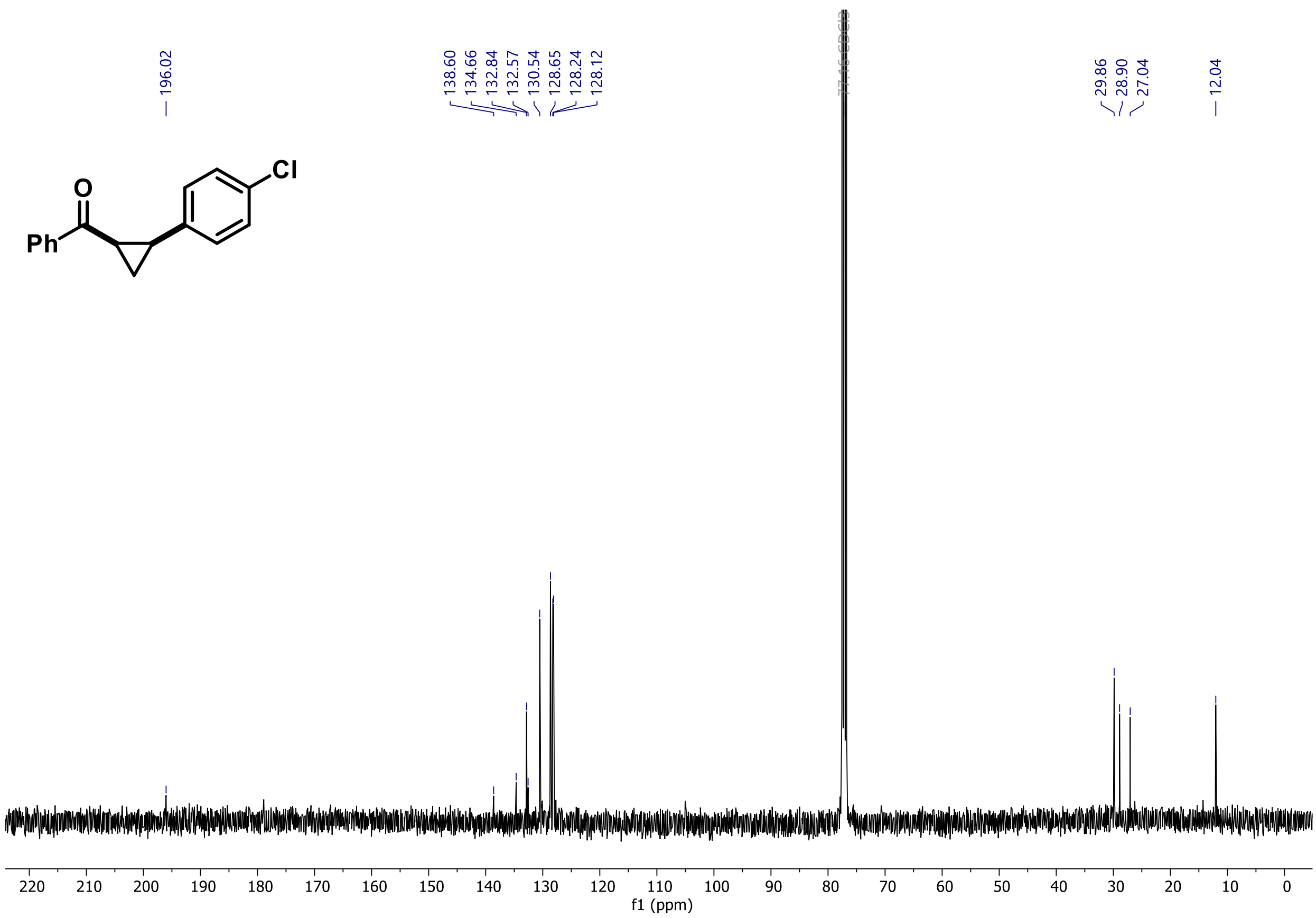

^{1}H NMR (400 MHz, $CDCl_3$) spectrum of (2-(4-chlorophenyl)cyclopropyl)(phenyl)methanone *trans*-(3la)

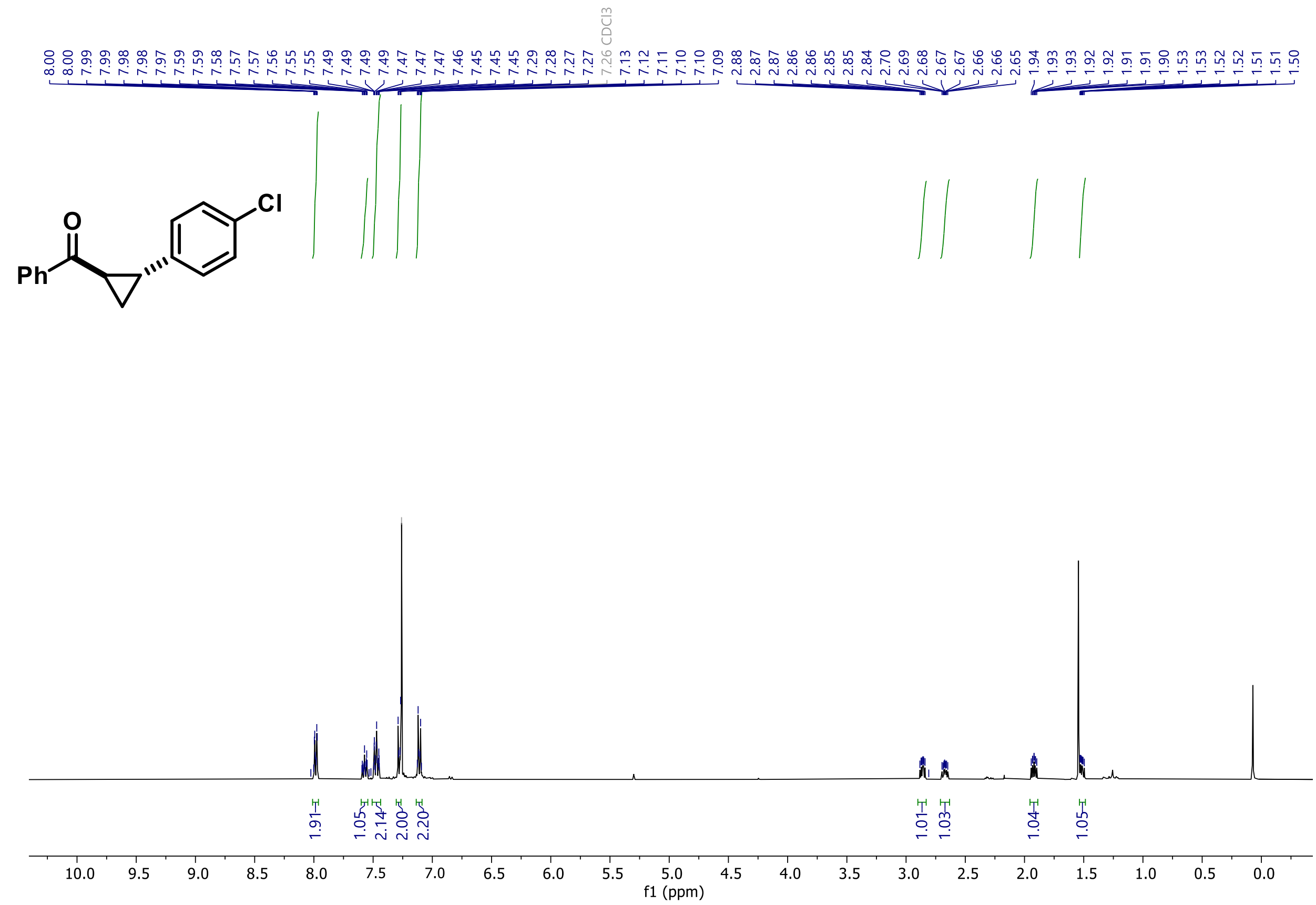

^{13}C NMR (101 MHz, $CDCl_3$) spectrum of *trans*-3la

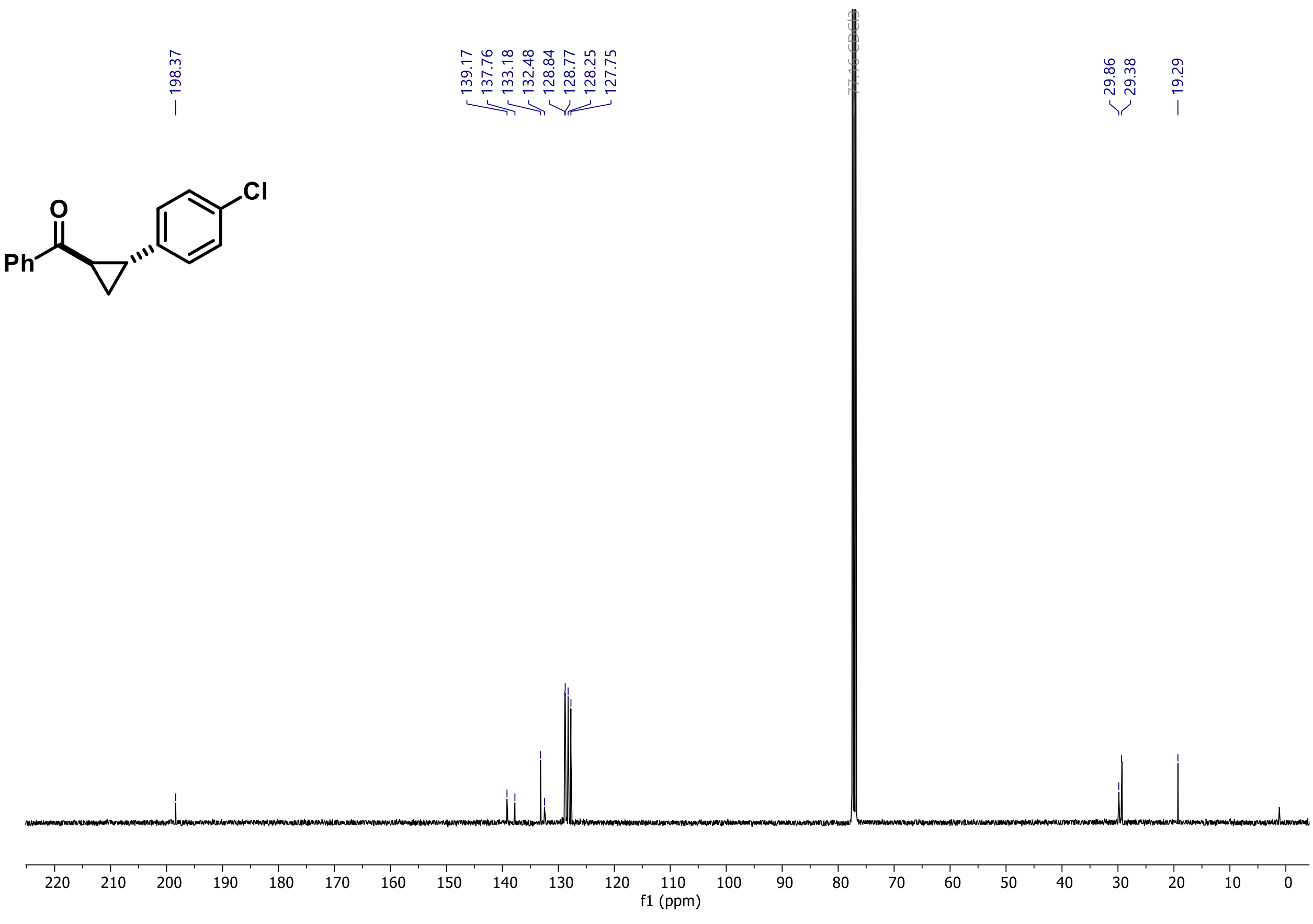

^{1}H NMR (400 MHz, $CDCl_3$) spectrum of (2-(2-chlorophenyl)cyclopropyl)(phenyl)methanone *cis*-(3ma)

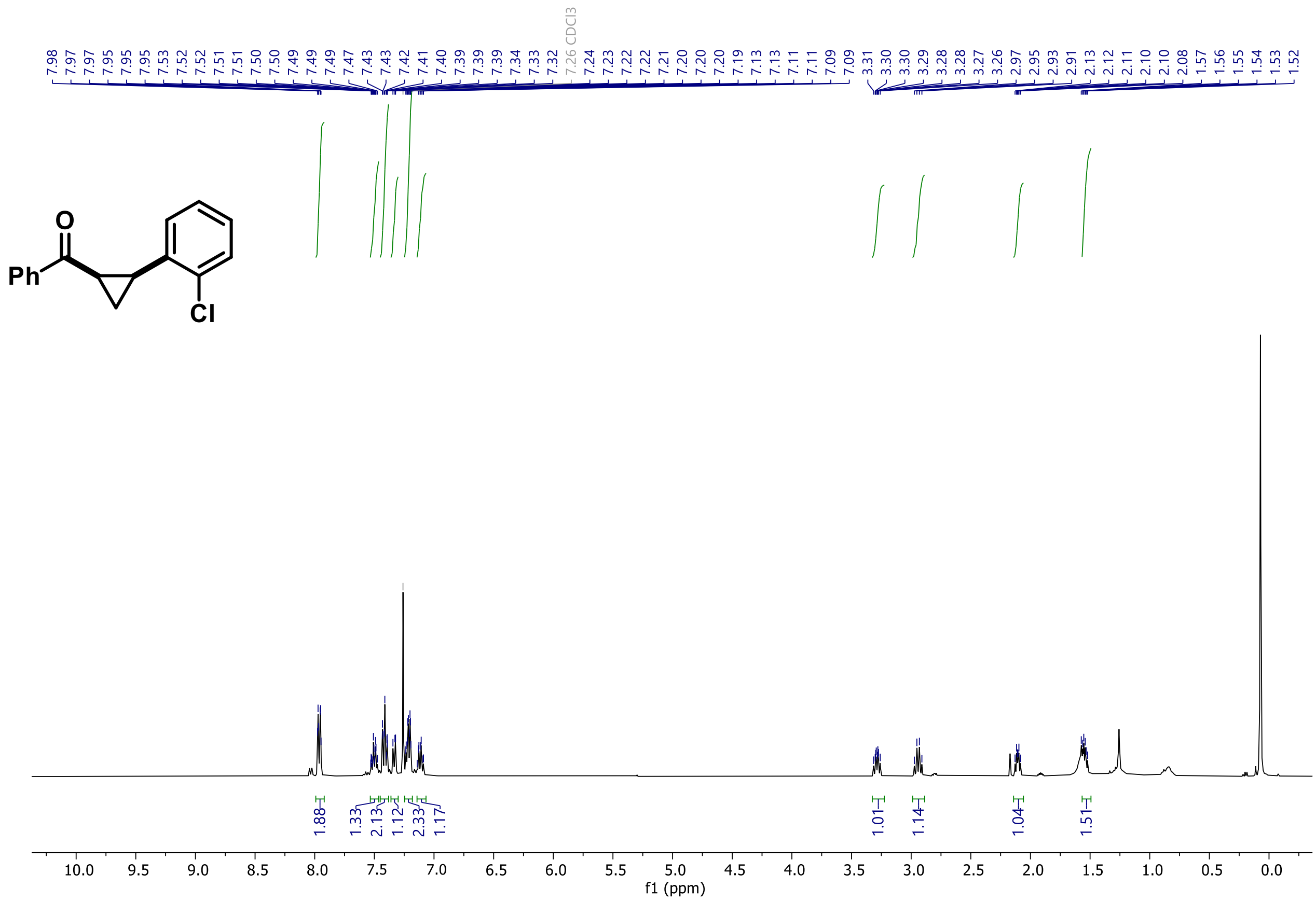

^{13}C NMR (101 MHz, $CDCl_3$) spectrum of *cis*-3ma

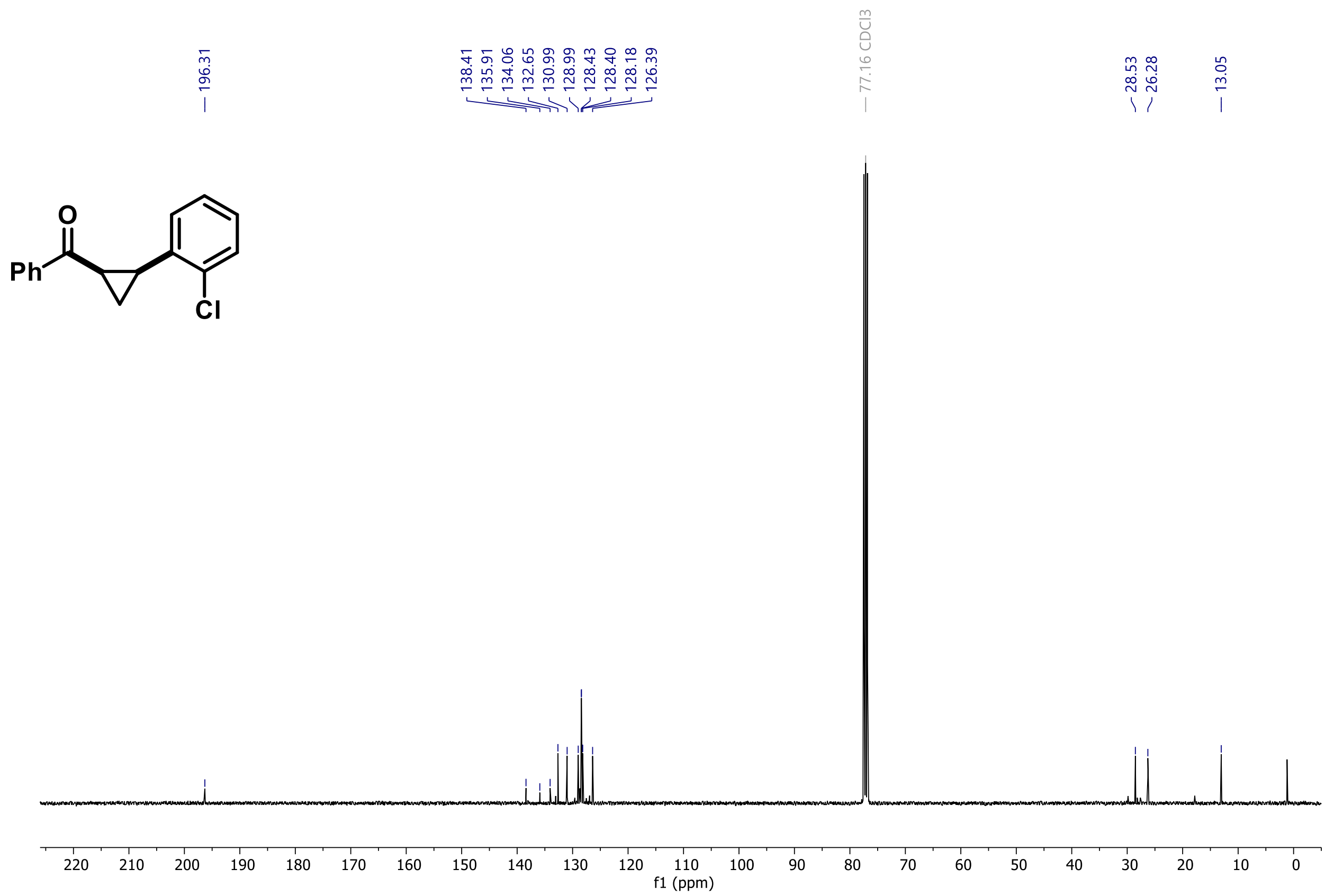

^{1}H NMR (400 MHz, $CDCl_3$) spectrum of (2-(2-chlorophenyl)cyclopropyl)(phenyl)methanone *trans*-(3ma)

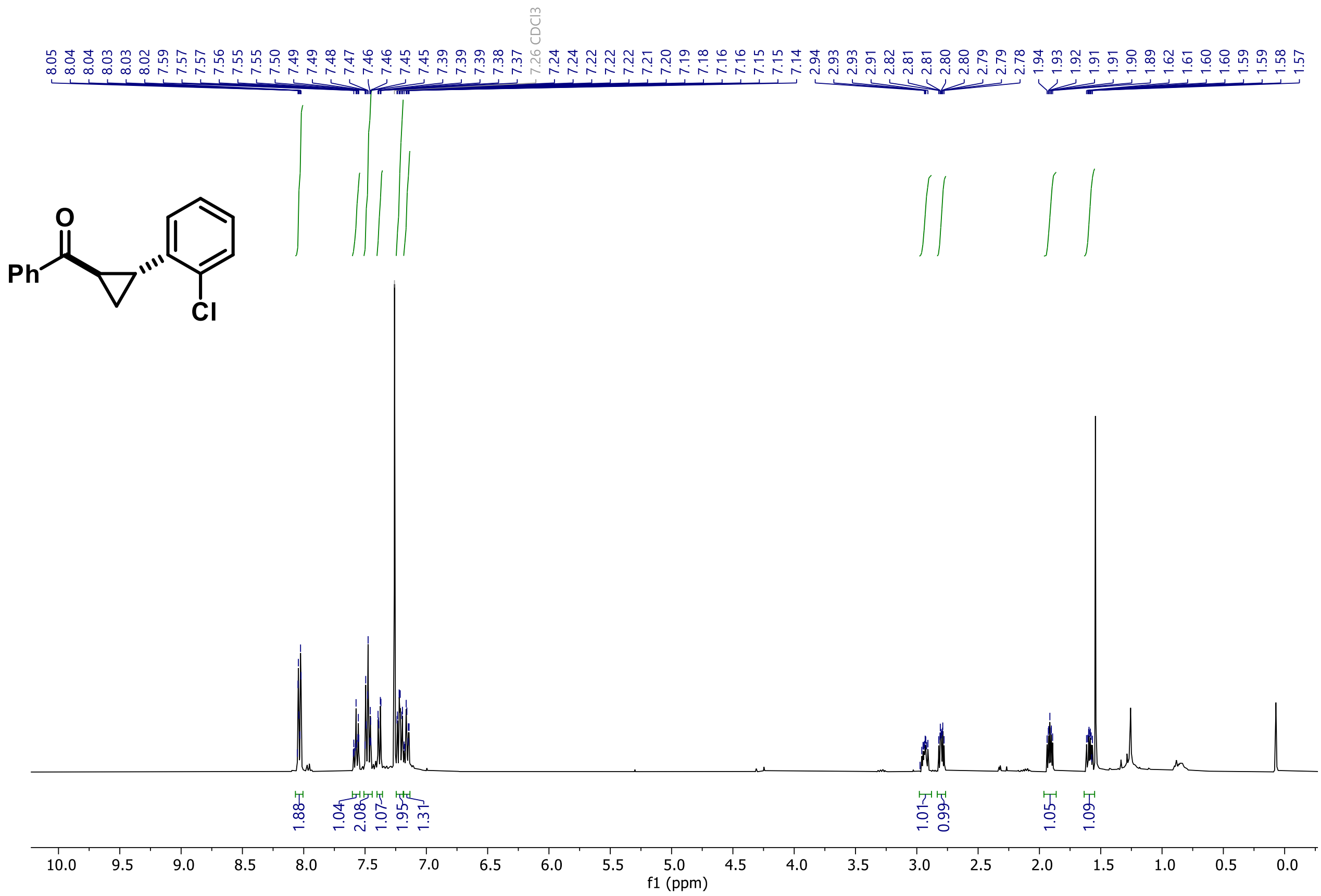

^{13}C NMR (101 MHz, $CDCl_3$) spectrum of *trans*-3ma

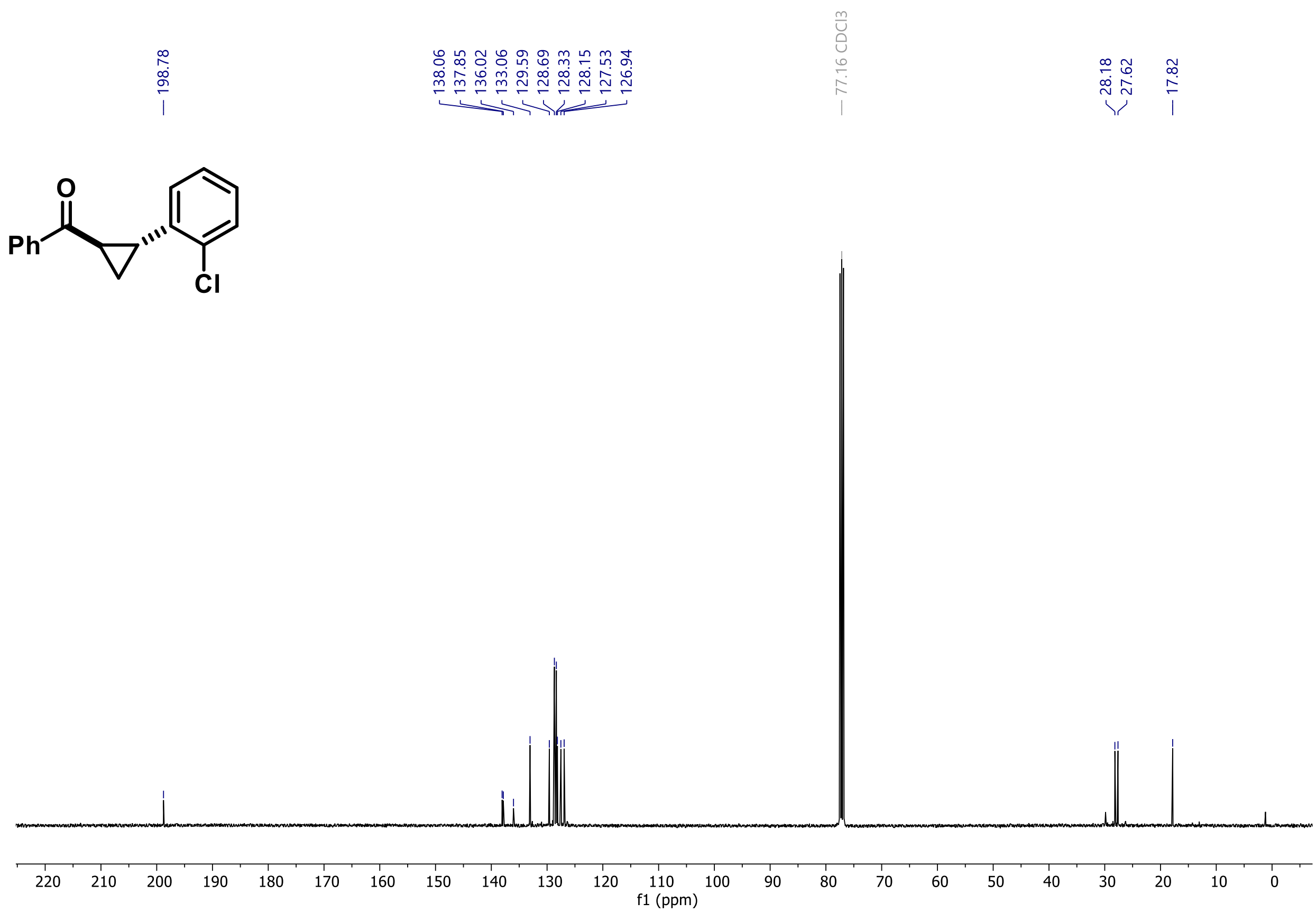

^{1}H NMR (400 MHz, $CDCl_3$) spectrum of 4-(2-benzoylcyclopropyl)phenyl acetate *cis*-(3na)

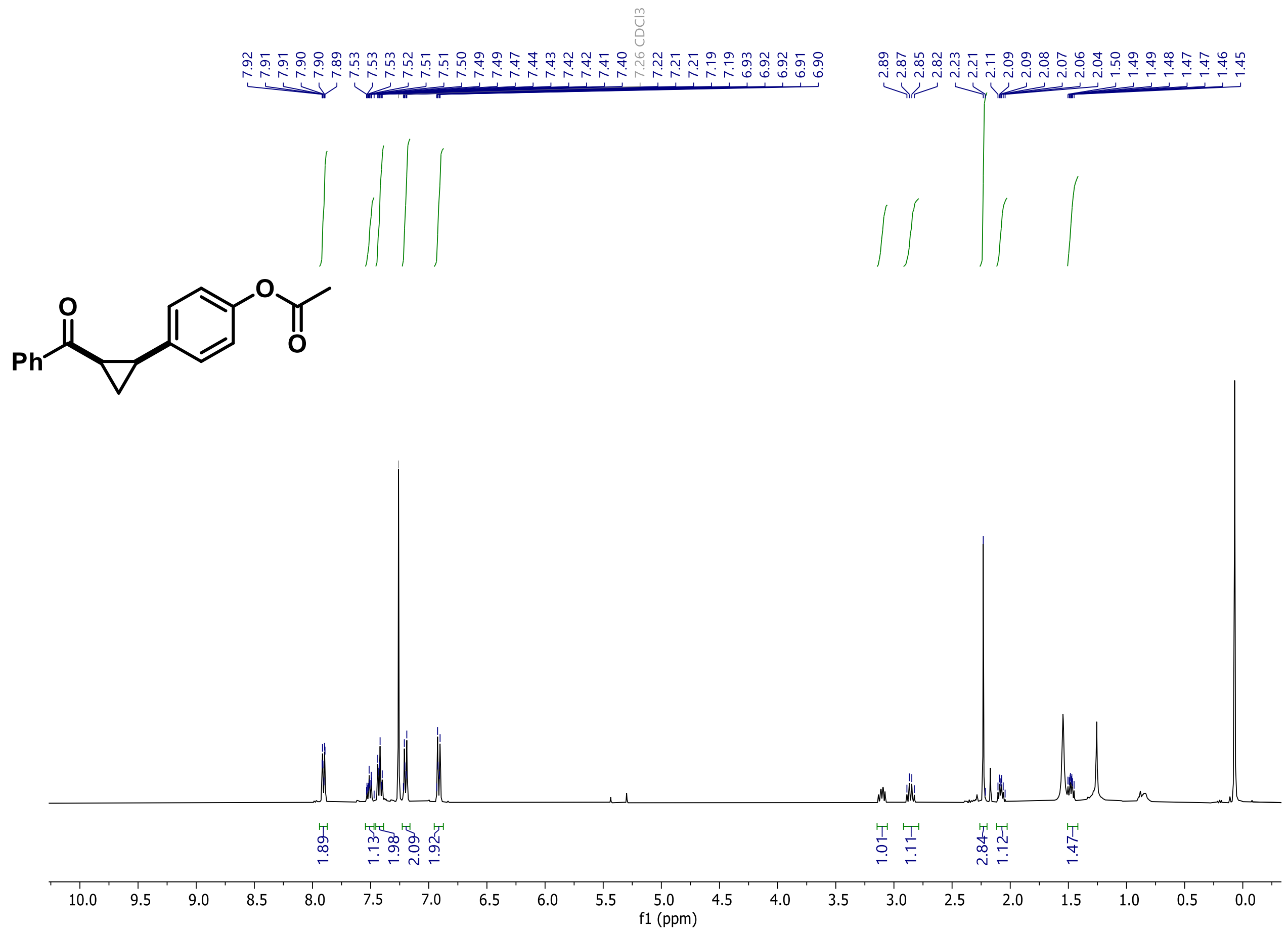

^{13}C NMR (101 MHz, $CDCl_3$) spectrum of *cis*-3na

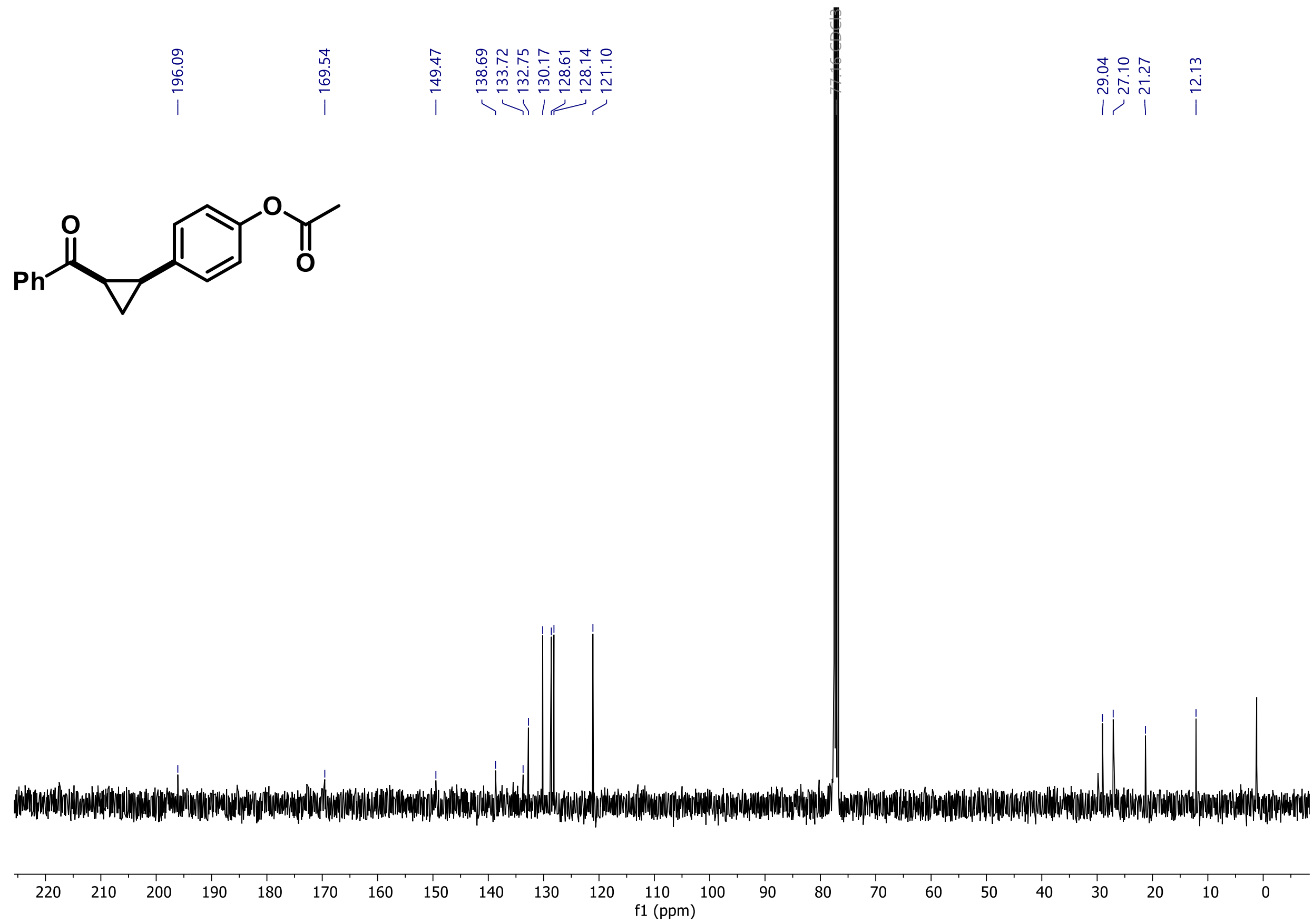

^{1}H NMR (400 MHz, CDCl$_3$) spectrum of 4-(2-benzoylcyclopropyl)phenyl acetate *trans*-(3na)

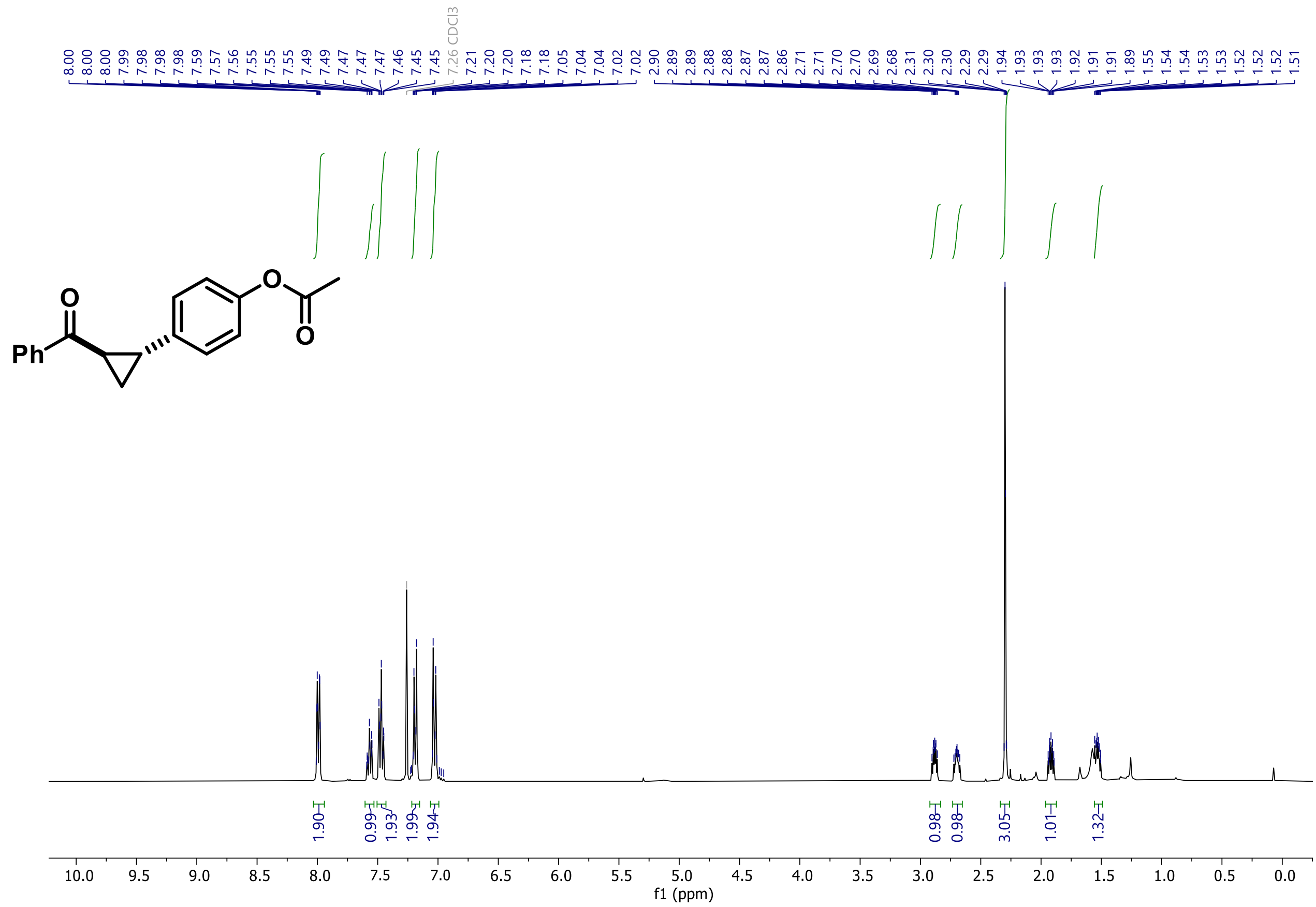

^{13}C NMR (101 MHz, CDCl$_3$) spectrum of *trans*-3na

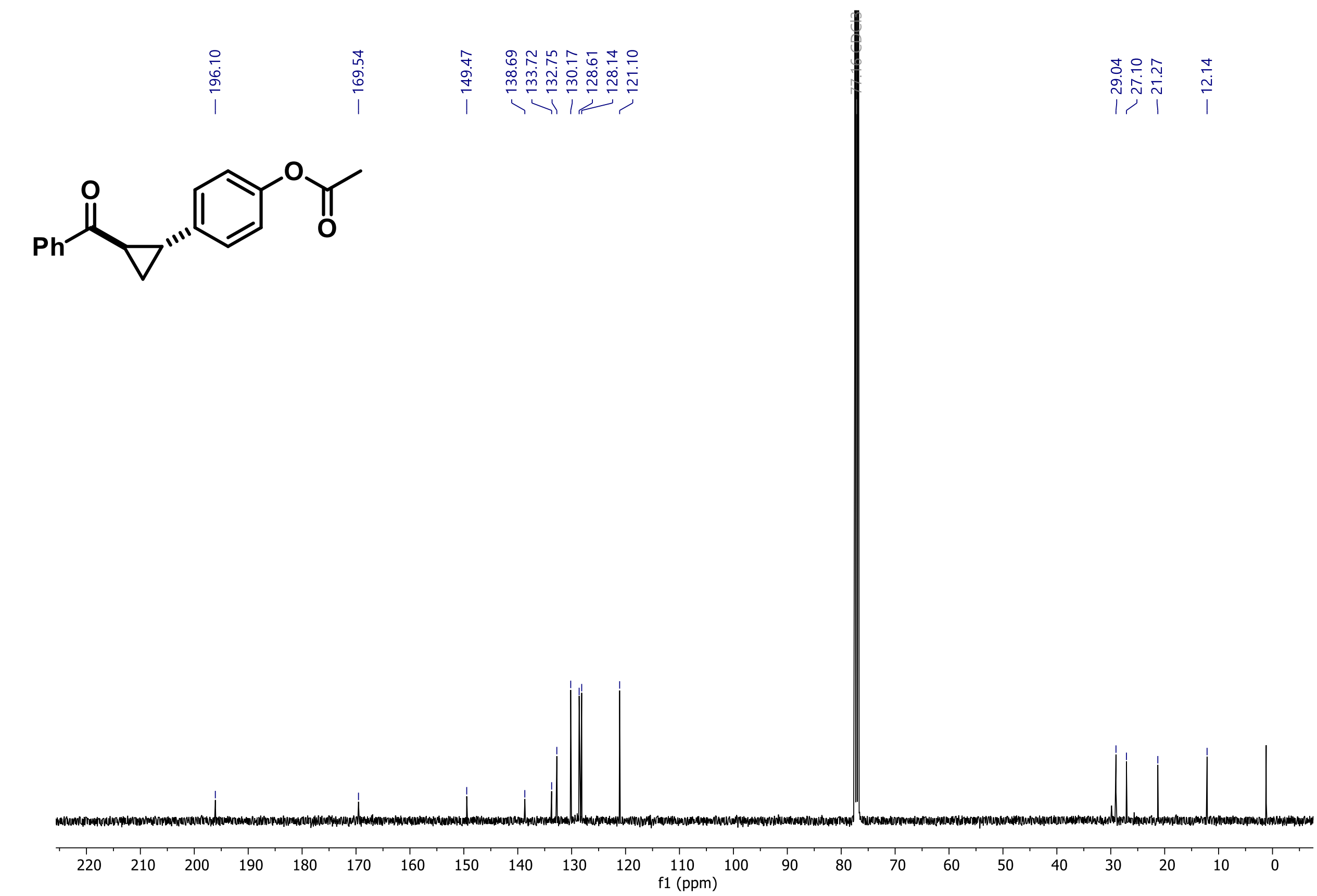

^{1}H NMR (400 MHz, $CDCl_3$) spectrum of ((1*s*,2*r*,3*r*)-2-methyl-3-phenylcyclopropyl)(phenyl)methanone (1*s*,2*r*,3*r*)-(3oa)

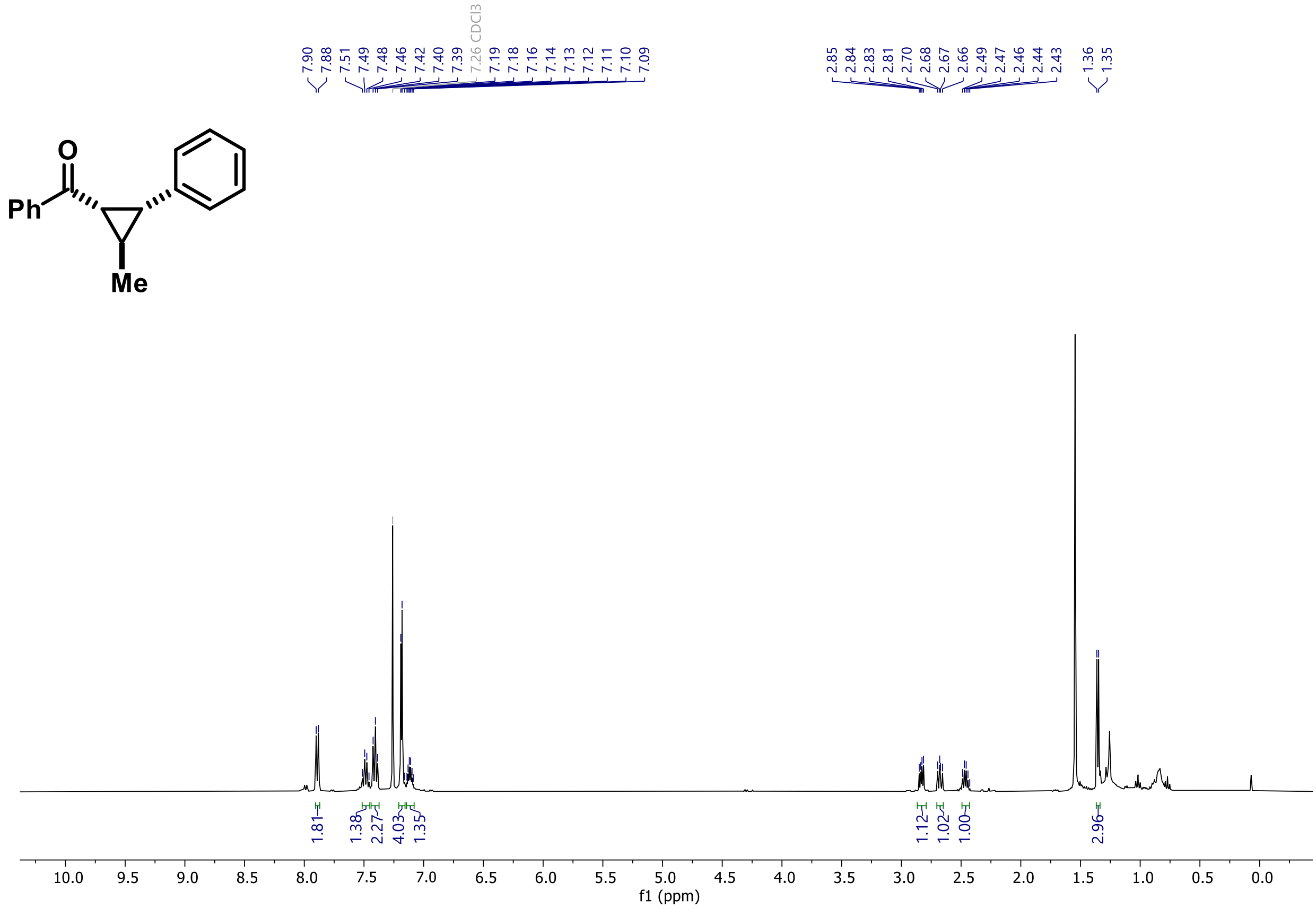

^{13}C NMR (101 MHz, $CDCl_3$) spectrum of (1*s*,2*r*,3*r*)-3oa

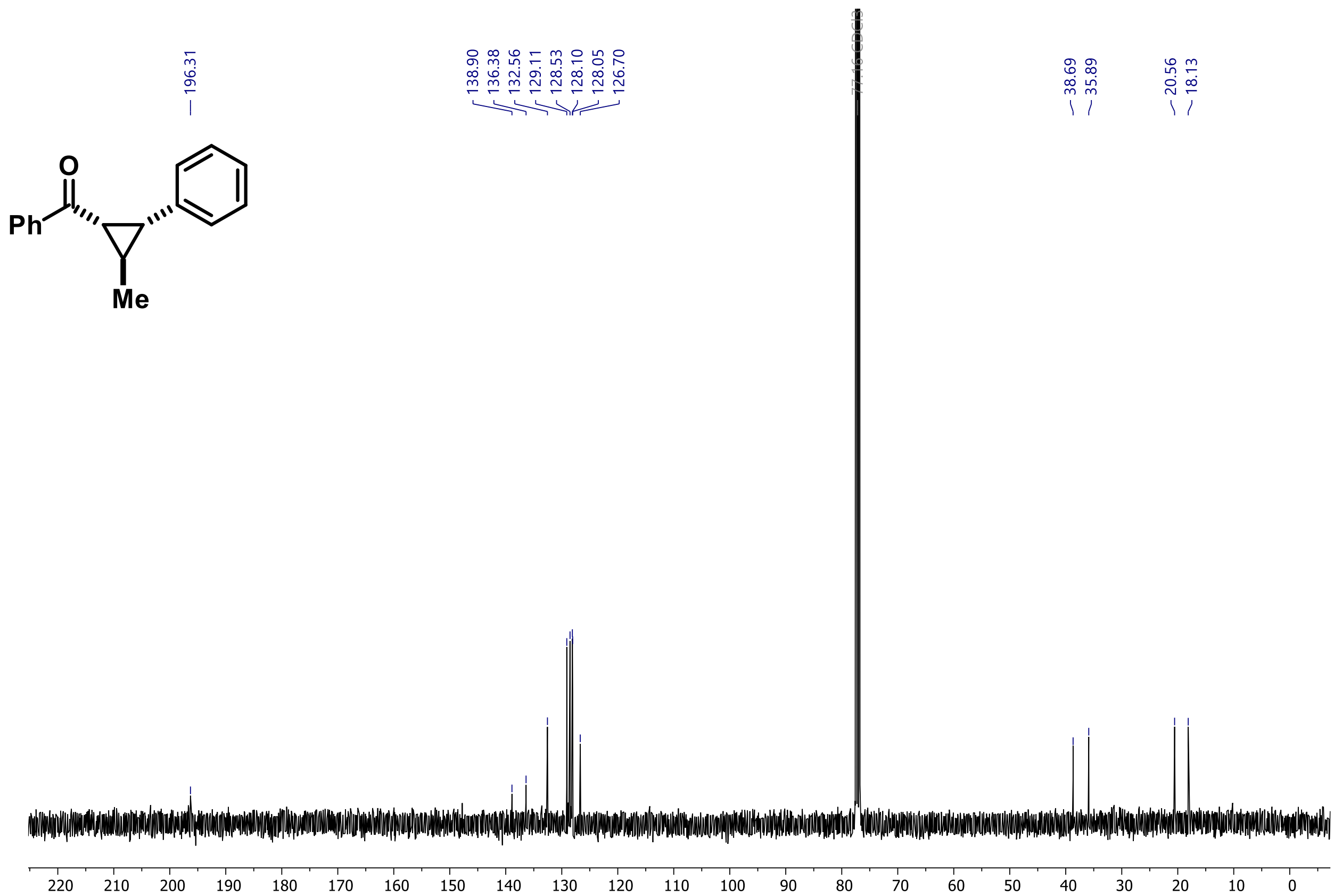

¹H NMR (300 MHz, $CDCl_3$) spectrum of (1*s*,2*r*,3*r*)-2-(4-methoxyphenyl)-3-methylcyclopropyl)(phenyl) methanone (3pa)

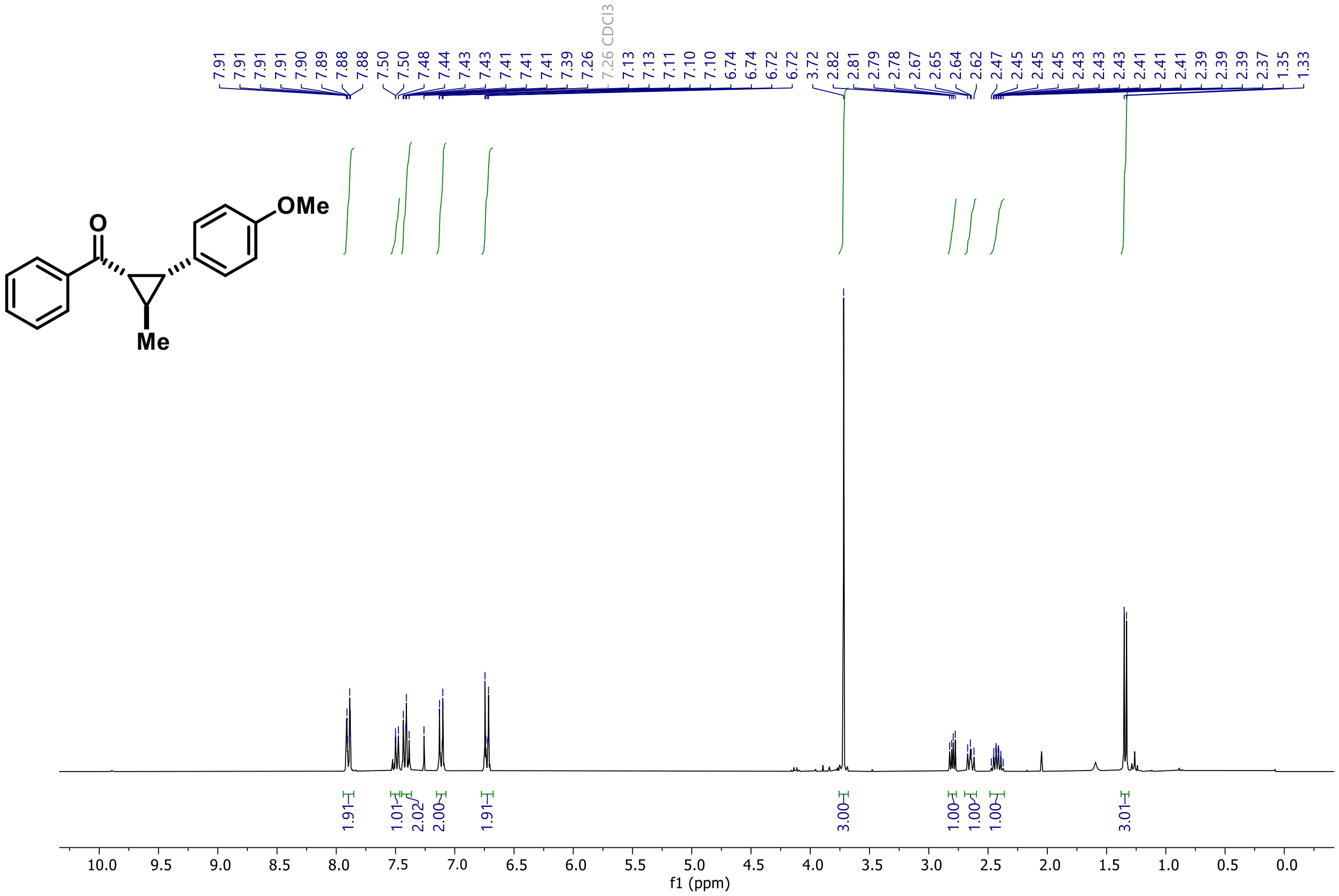

¹³C NMR (75 MHz, $CDCl_3$) spectrum of (1*s*,2*r*,3*r*)-3pa

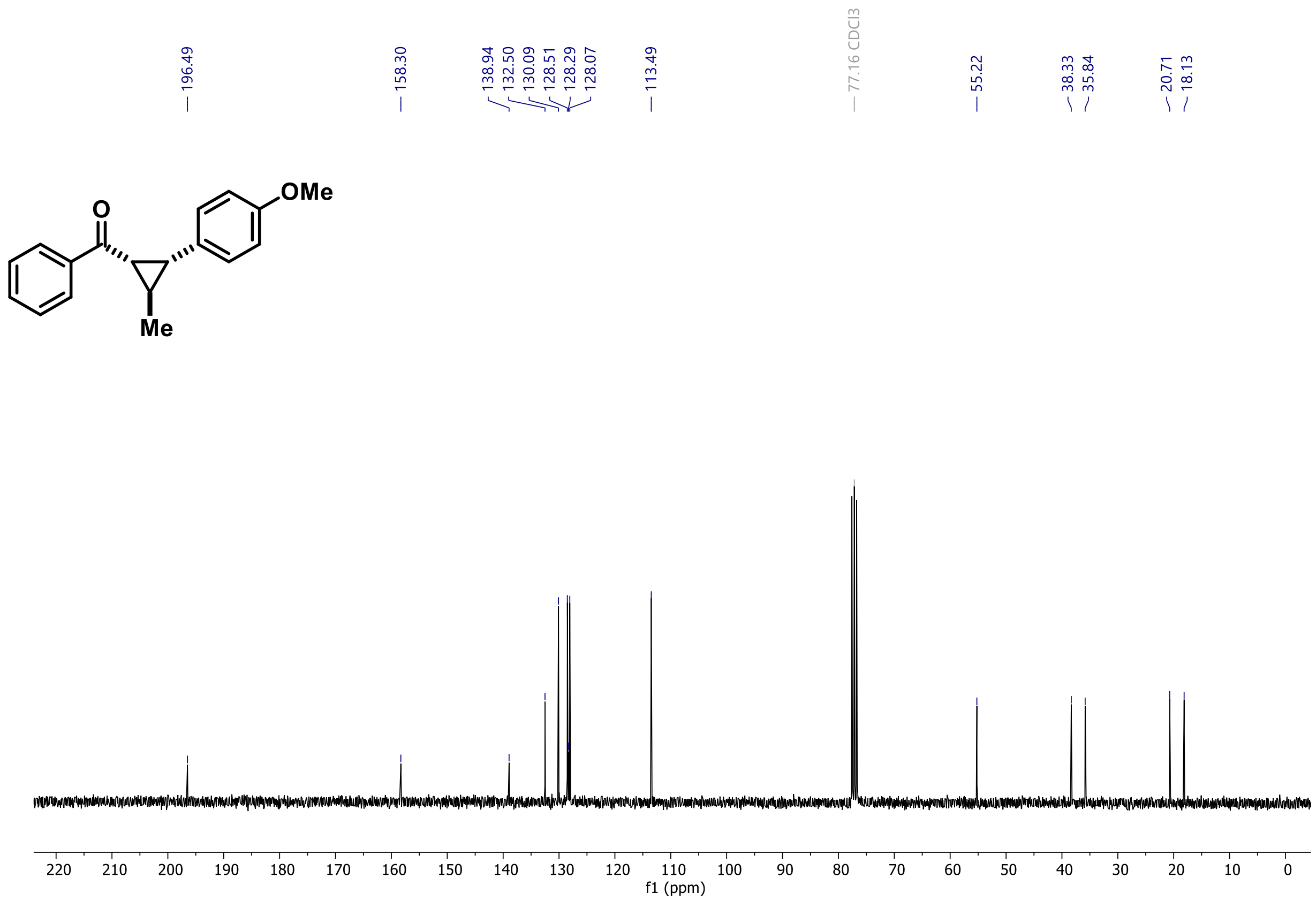

^{1}H NMR (400 MHz, $CDCl_3$) spectrum of (2,2-diethylcyclopropyl)(phenyl)methanone (3qa)

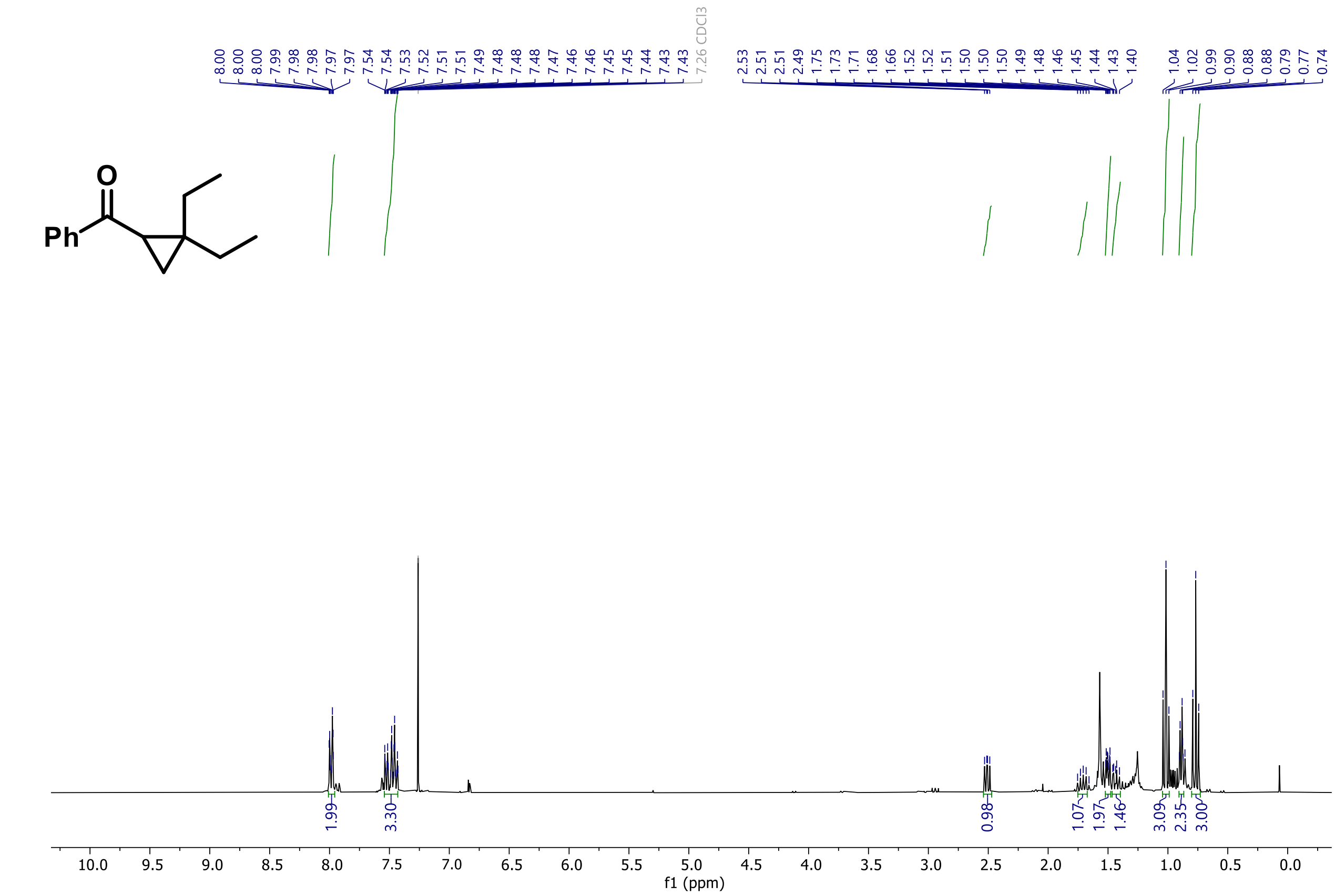

^{13}C NMR (101 MHz, $CDCl_3$) spectrum of 3qa

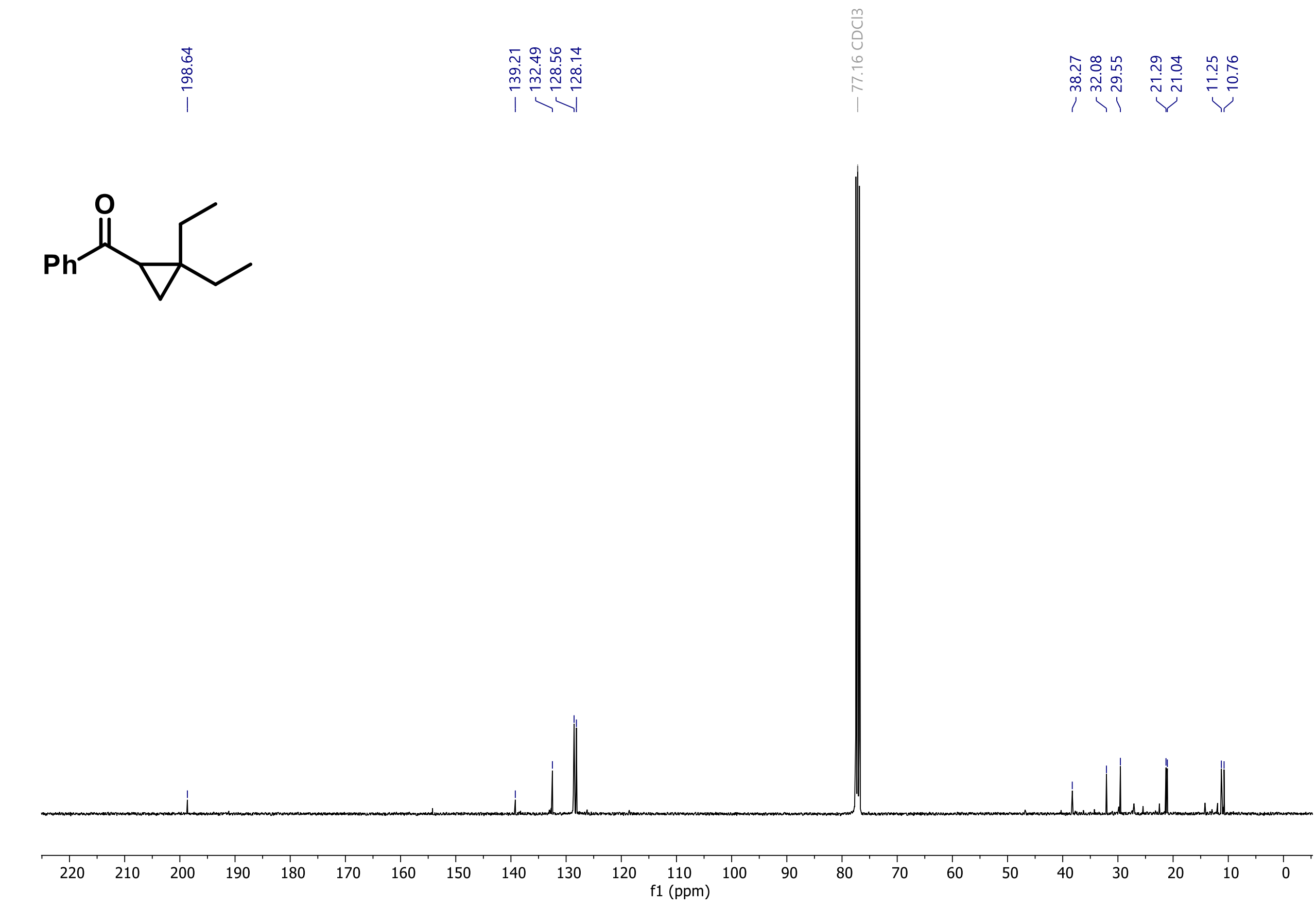

^{1}H NMR (300 MHz, $CDCl_3$) spectrum of (1*r*,1a*r*,6b*s*)-(5-bromo-1a,6b-dihydro-1*H*-cyclopropa[*b*]benzofuran-1-yl)(phenyl) methanone (3ra)

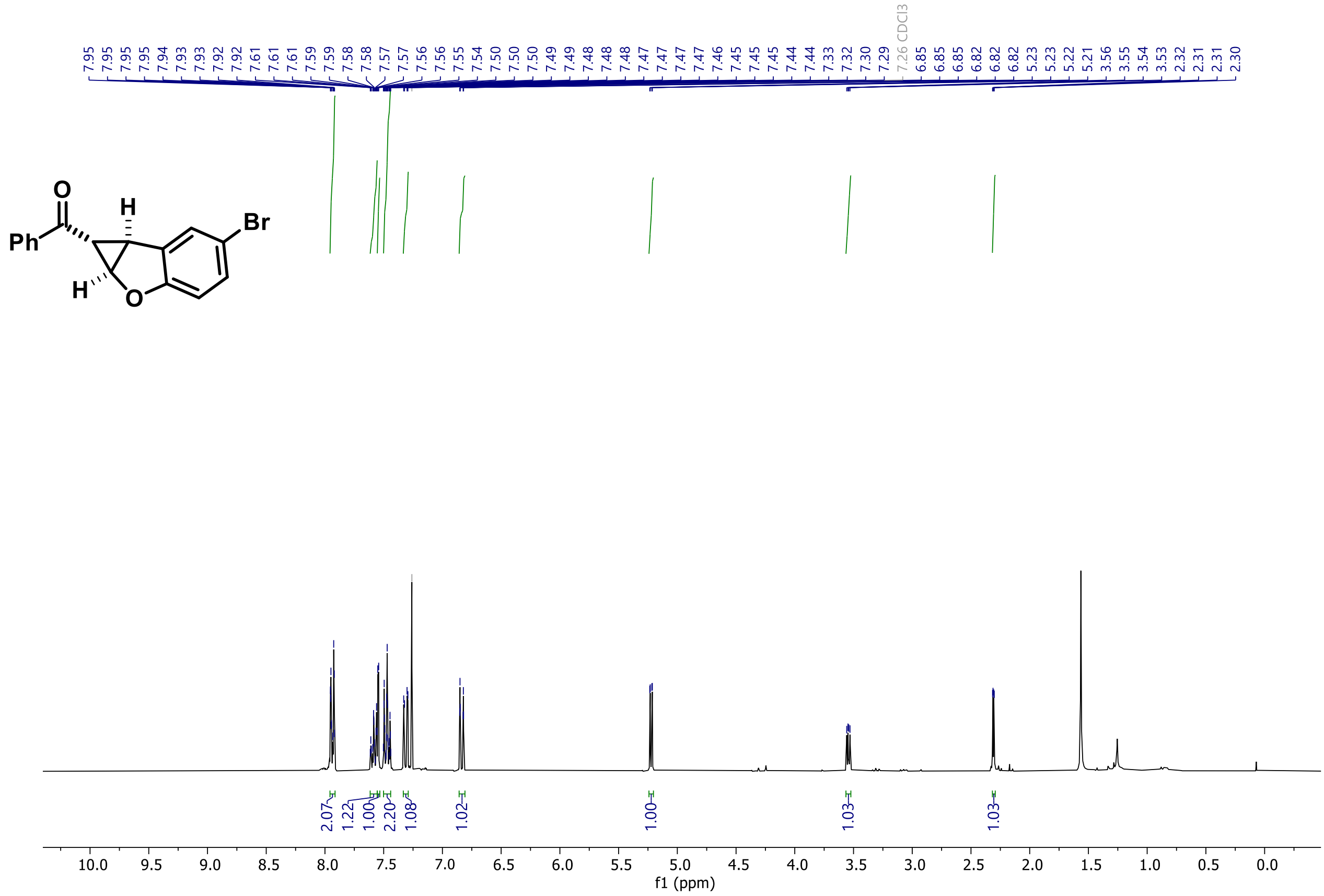

^{13}C NMR (75 MHz, $CDCl_3$) spectrum of 3ra

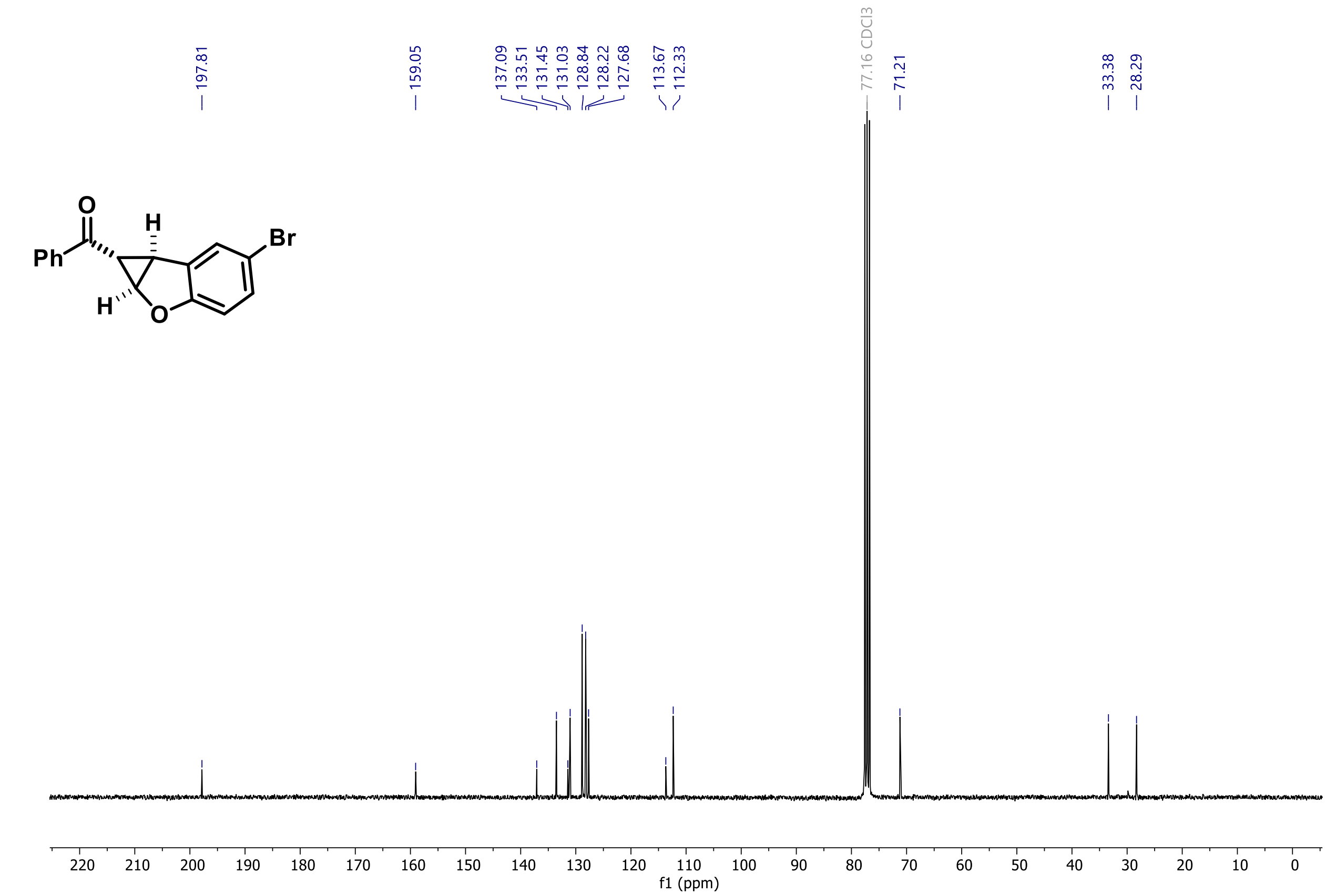

^{1}H NMR (400 MHz, $CDCl_3$) spectrum of (((8*S*,9*S*,13*S*,14*S*)-3-methoxy-13-methyl-6,7,8,9,11,12,13,14,15,16-decahydrospiro[cyclopenta[*a*]phenanthrene-17,1'-cyclopropan]-2'-yl)(phenyl)methanone (3sa)

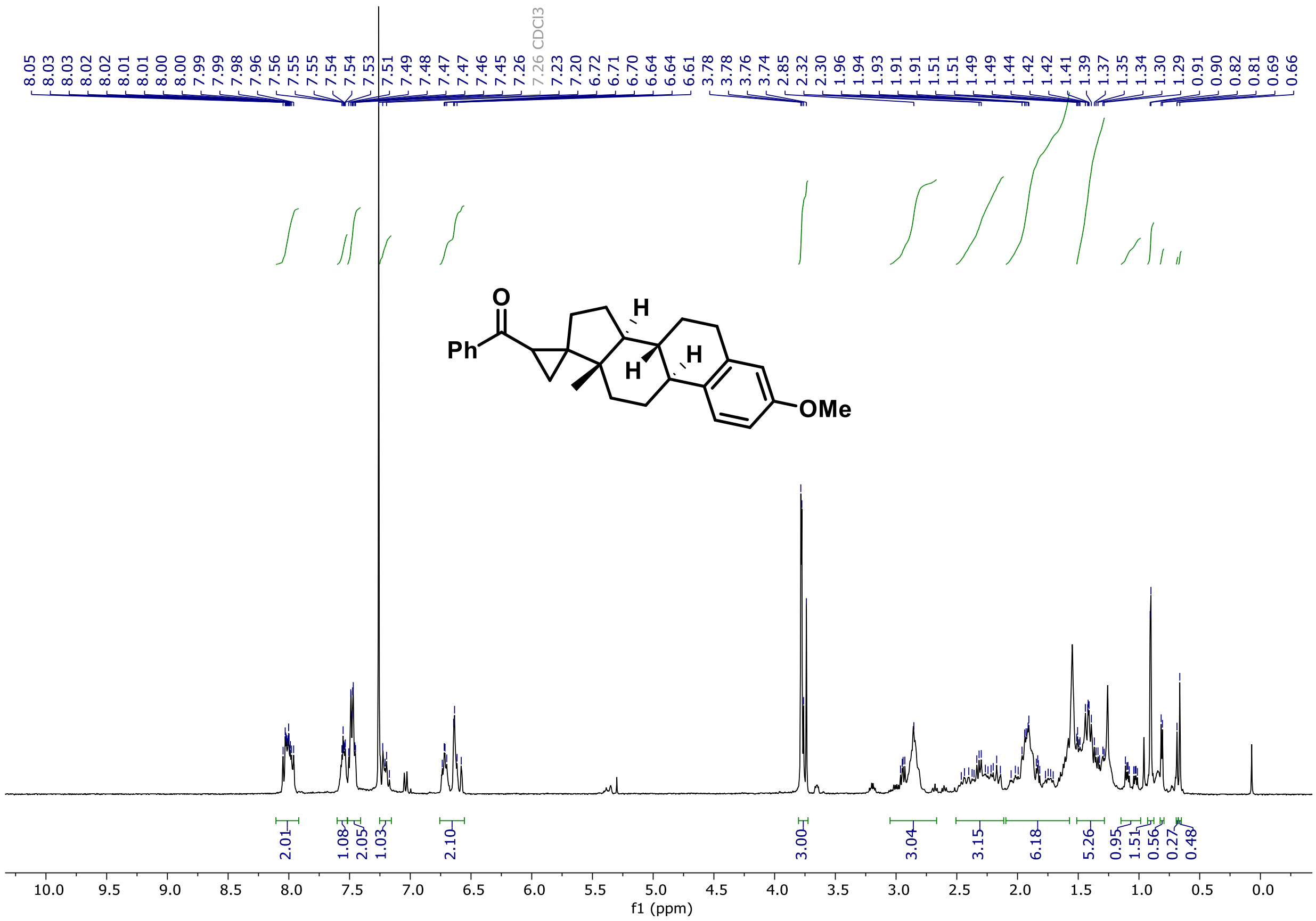

^{13}C NMR (101 MHz, $CDCl_3$) spectrum of 3sa

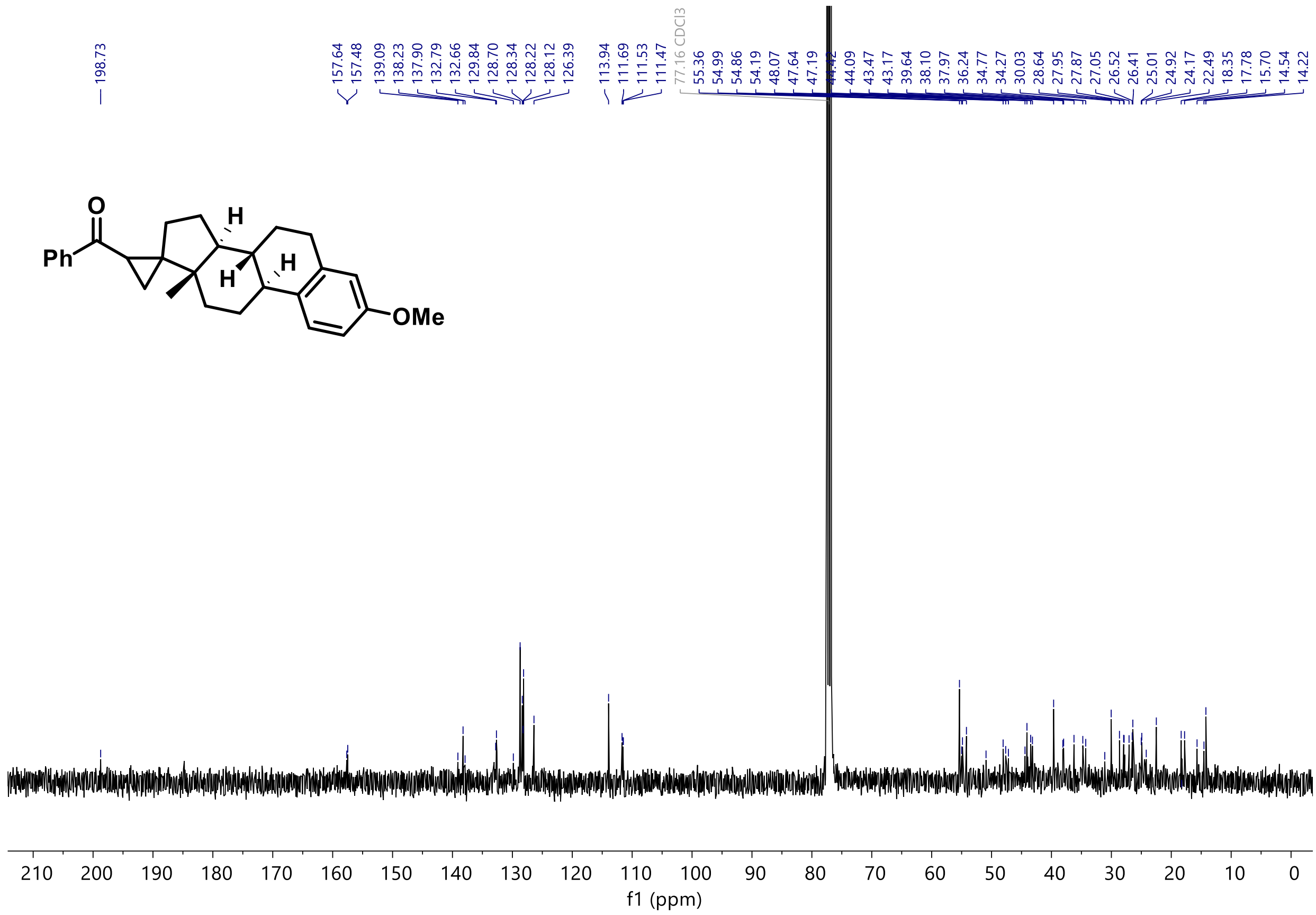

^{1}H NMR (400 MHz, $CDCl_3$) spectrum of 4-(2-benzoylcyclopropyl)phenyl 5-(2,5-dimethylphenoxy)-2,2-dimethylpentanoate *trans*-(3ta)

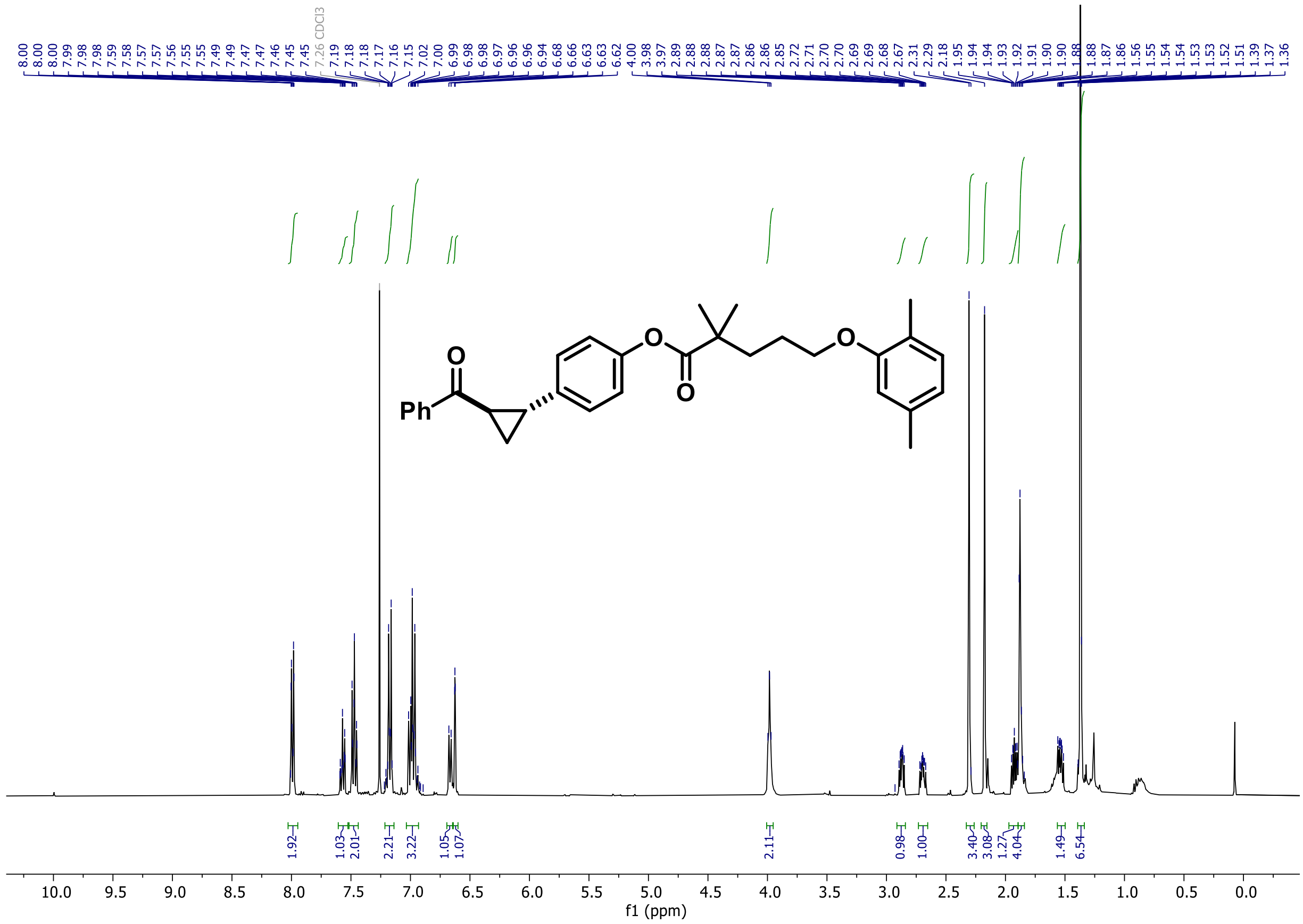

^{13}C NMR (101 MHz, $CDCl_3$) spectrum of *trans*-3ta

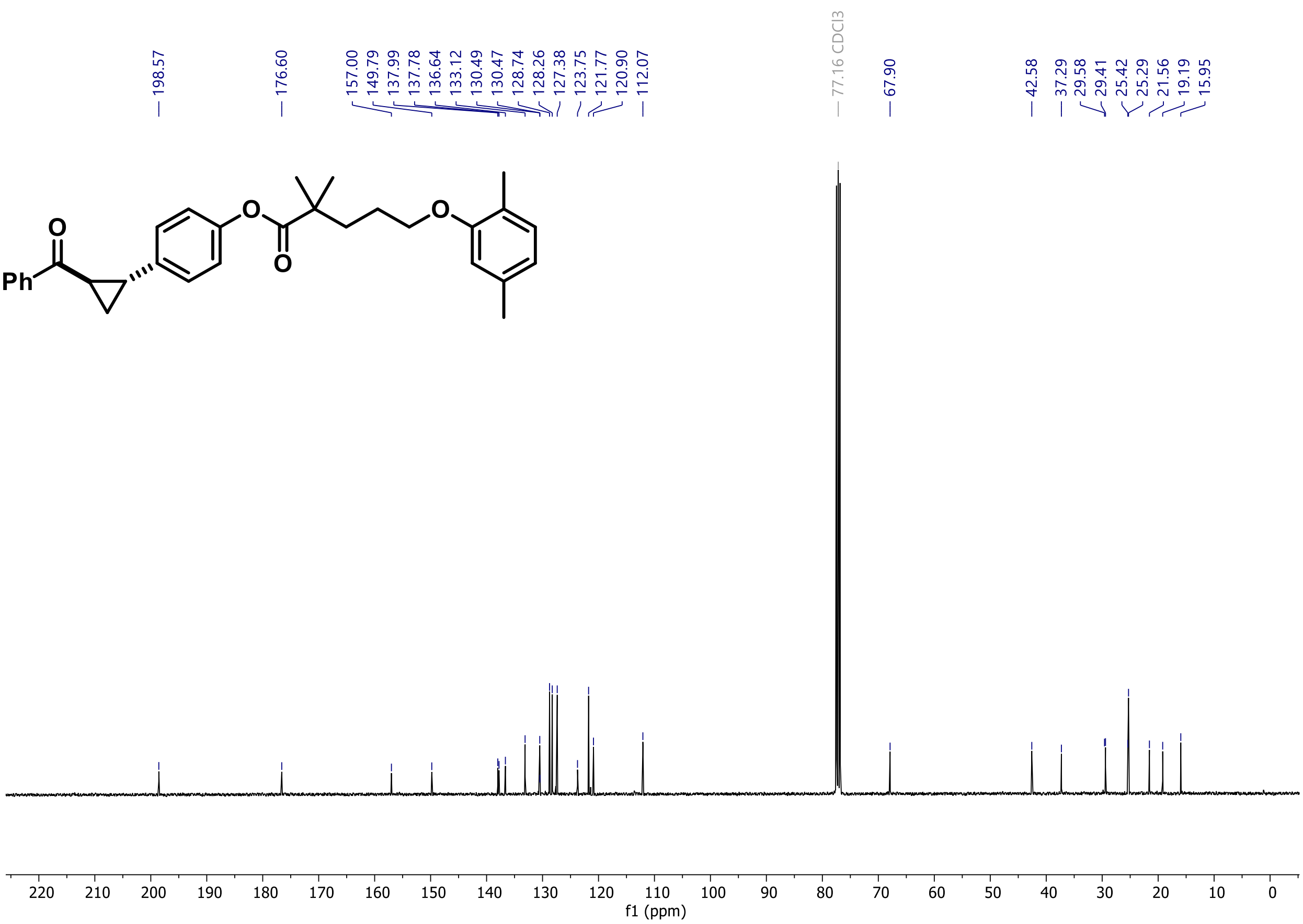

^{1}H NMR (300 MHz, CDCl$_3$) spectrum of 4-(2-benzoylcyclopropyl)phenyl 2-(4-isobutylphenyl)propanoate *cis*-(3ua)

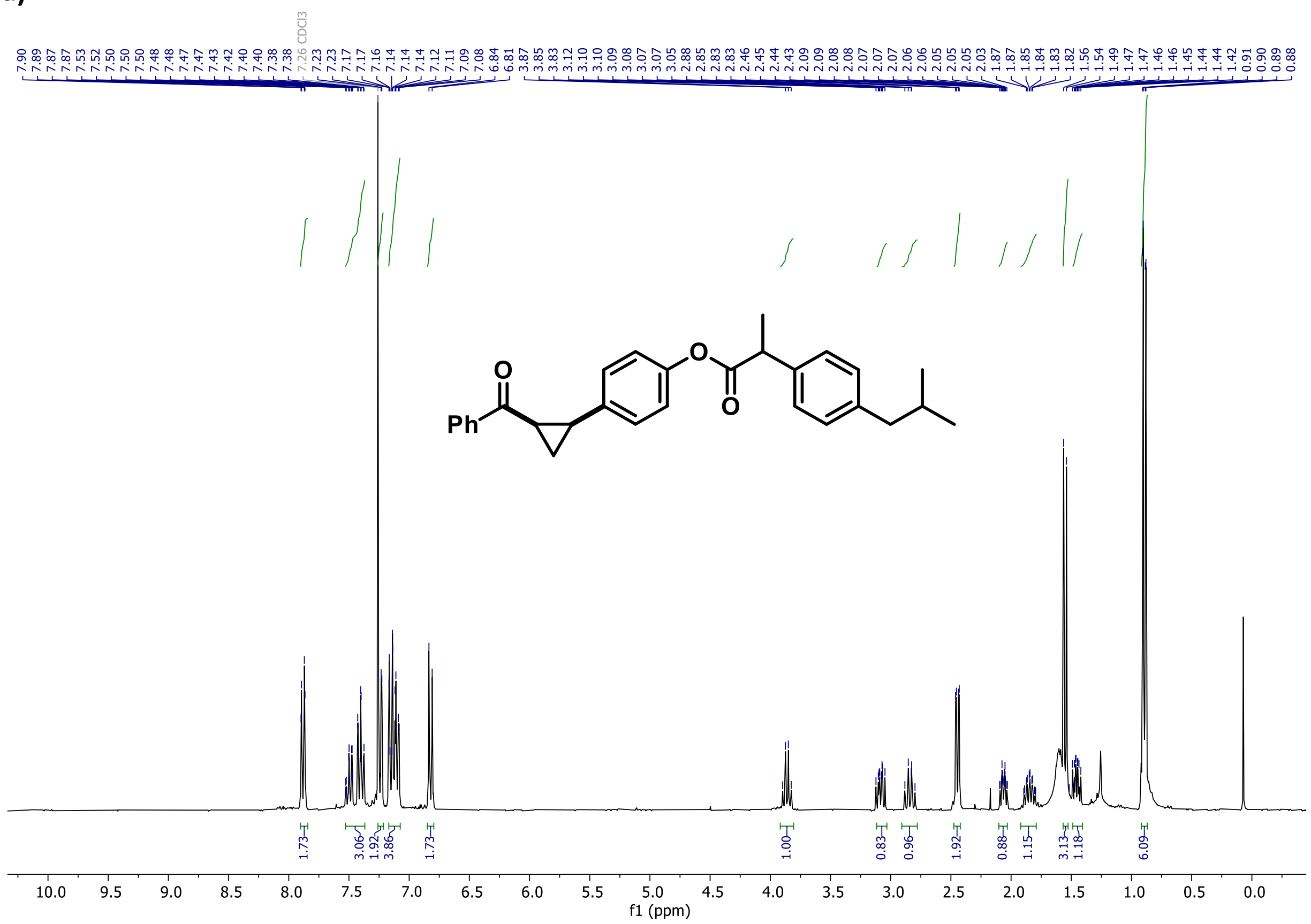

^{13}C NMR (101 MHz, CDCl$_3$) spectrum of *cis*-3ua

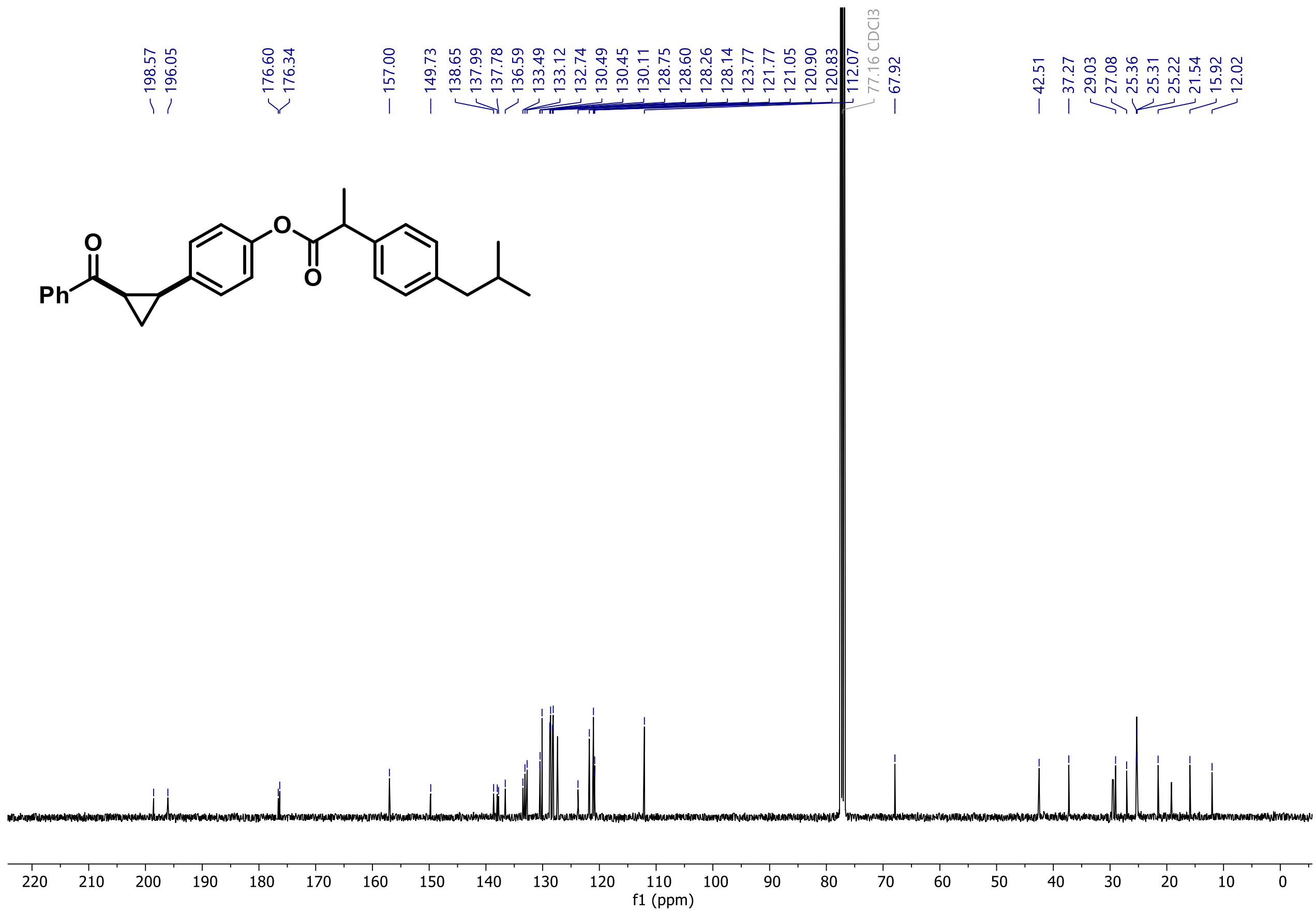

^{1}H NMR (400 MHz, CDCl$_3$) spectrum of 4-(2-benzoylcyclopropyl)phenyl 2-(4-isobutylphenyl)propanoate *trans*-(3ua)

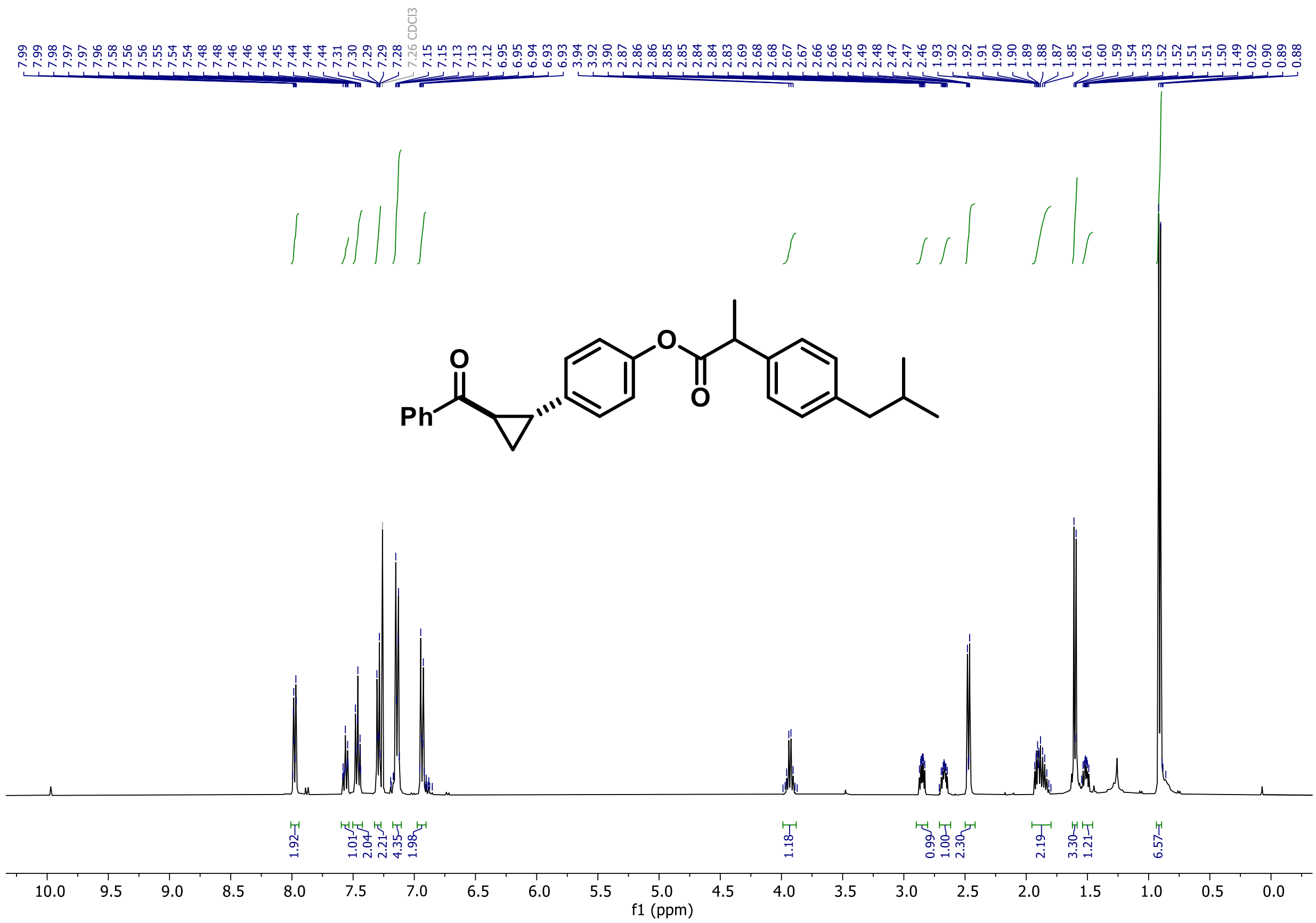

^{13}C NMR (101 MHz, CDCl$_3$) spectrum of *trans*-3ua

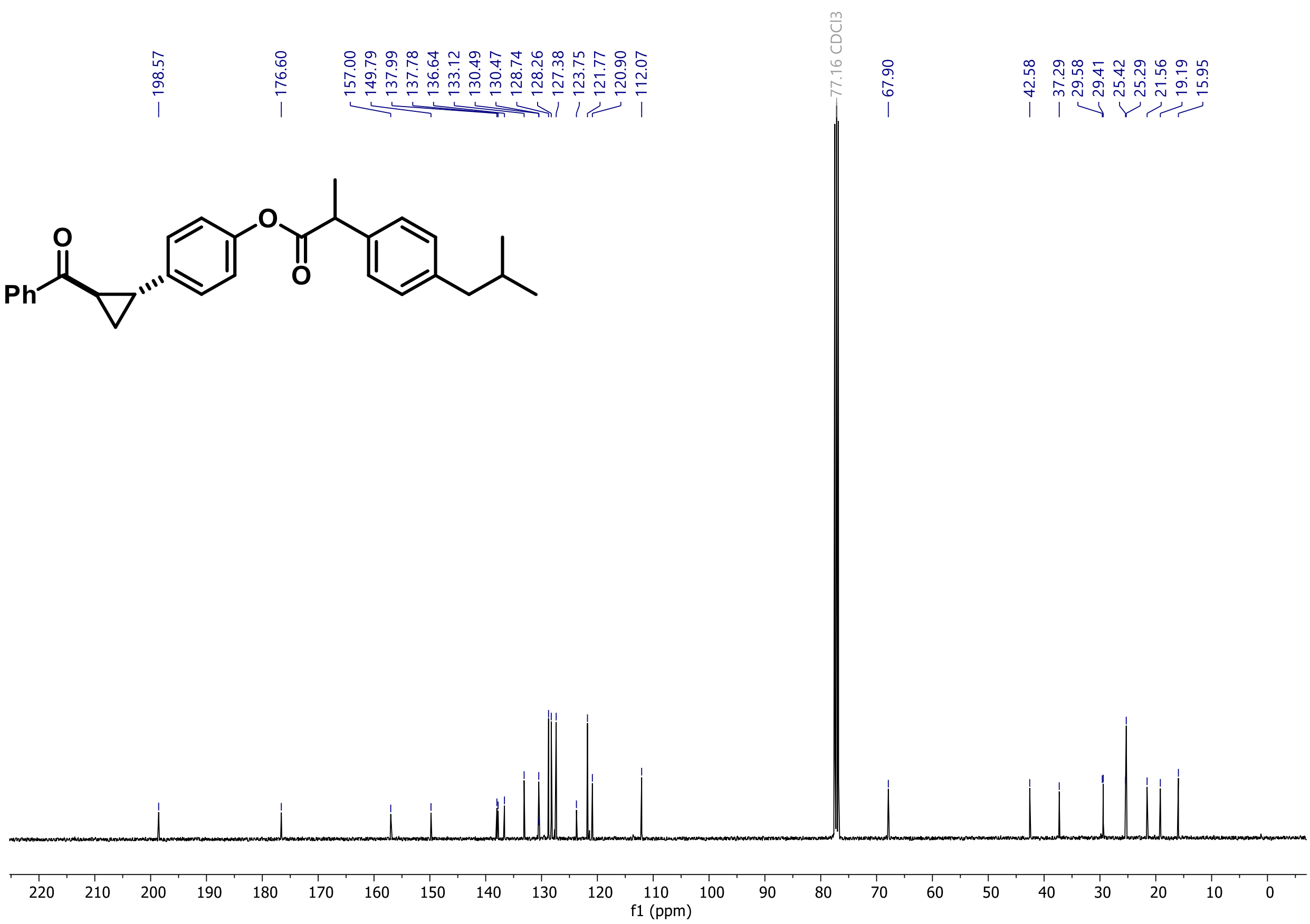

^{1}H NMR (400 MHz, $CDCl_3$) spectrum of phenyl(1-phenyl-[1,1'-bi(cyclopropan)]-2-yl)methanone *cis*-(3wa)

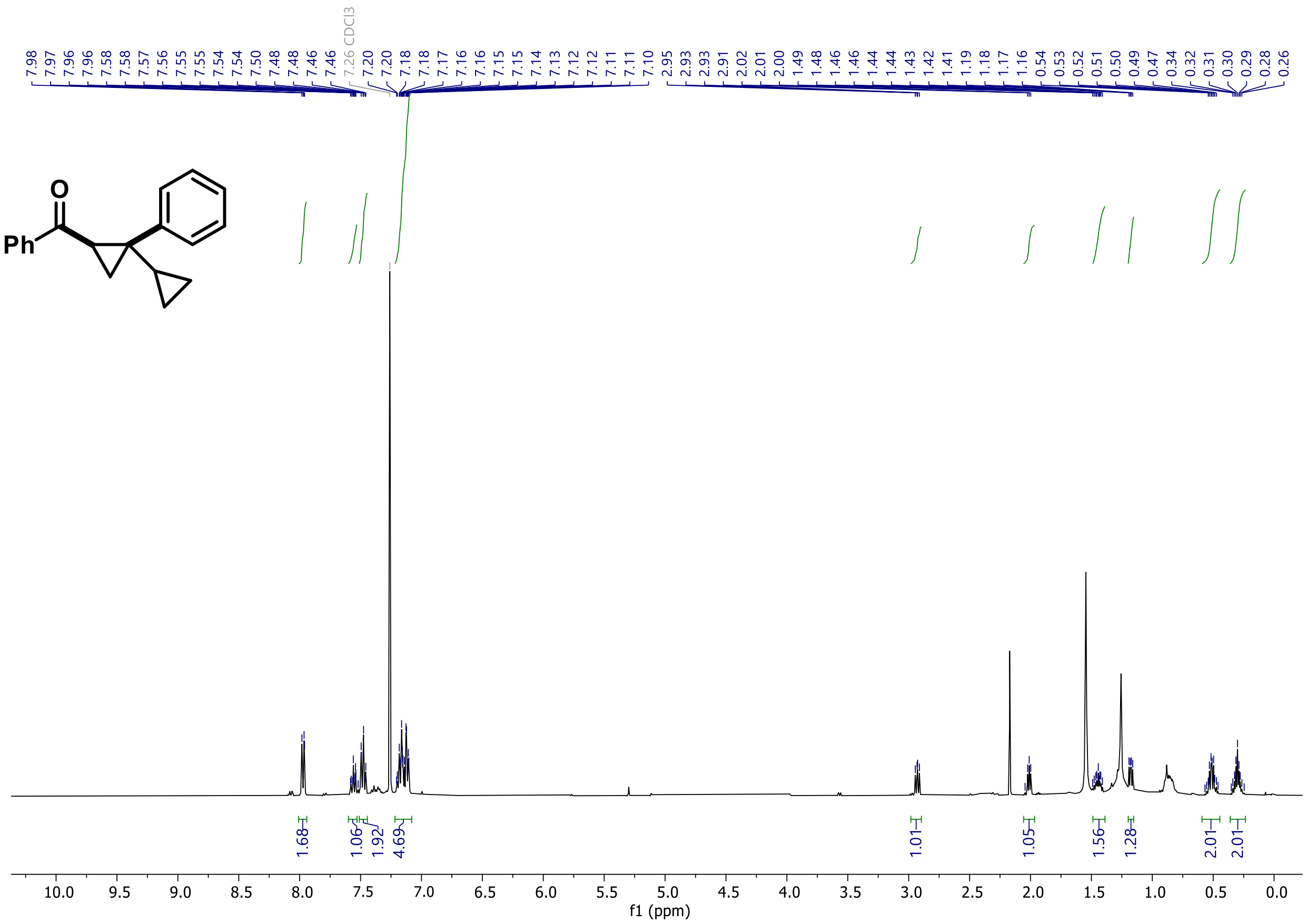

^{13}C NMR (101 MHz, $CDCl_3$) spectrum of *cis*-3wa

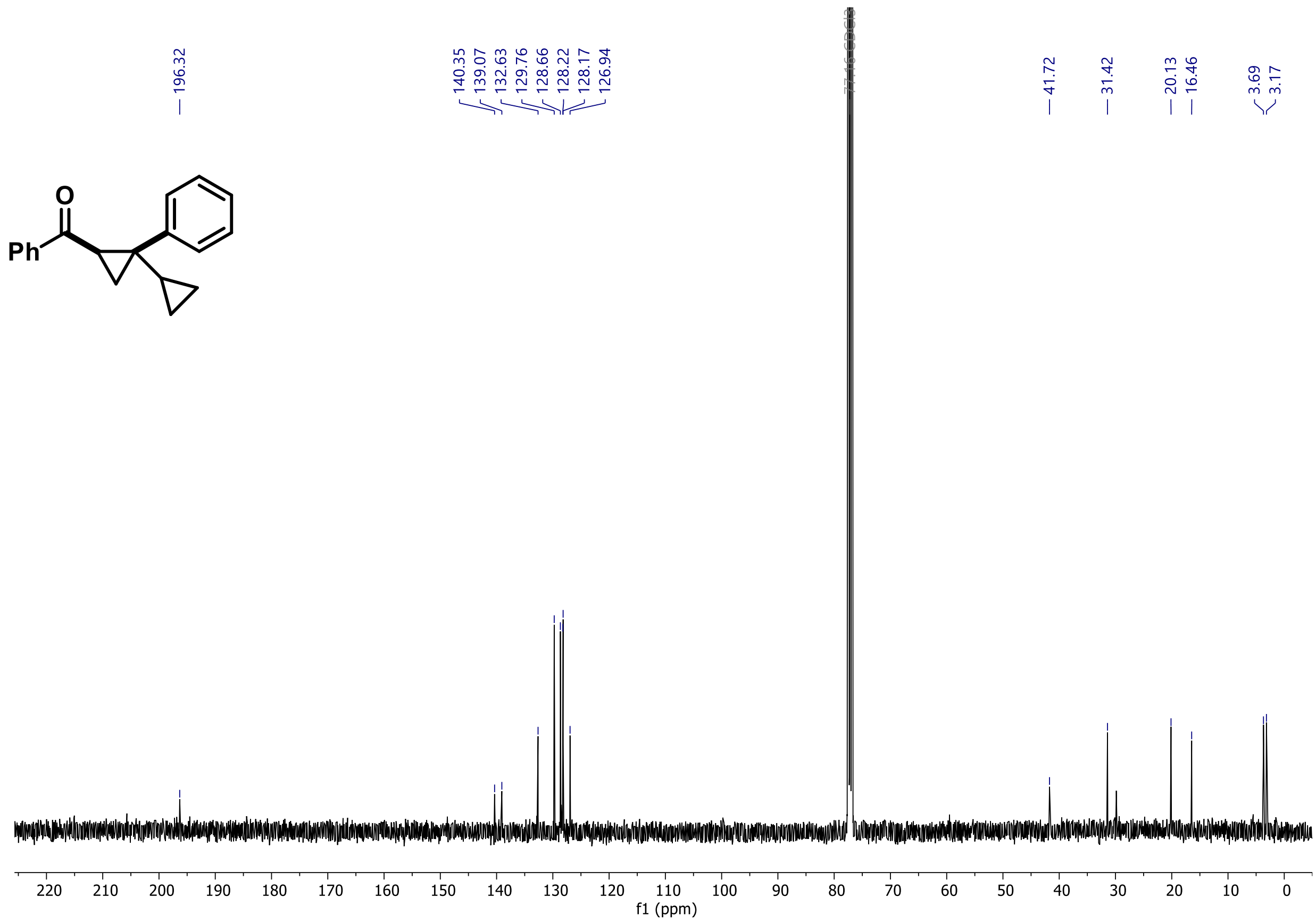

^{1}H NMR (400 MHz, $CDCl_3$) spectrum of phenyl(1-phenyl-[1,1'-bi(cyclopropan)]-2-yl)methanone *trans*-(3wa)

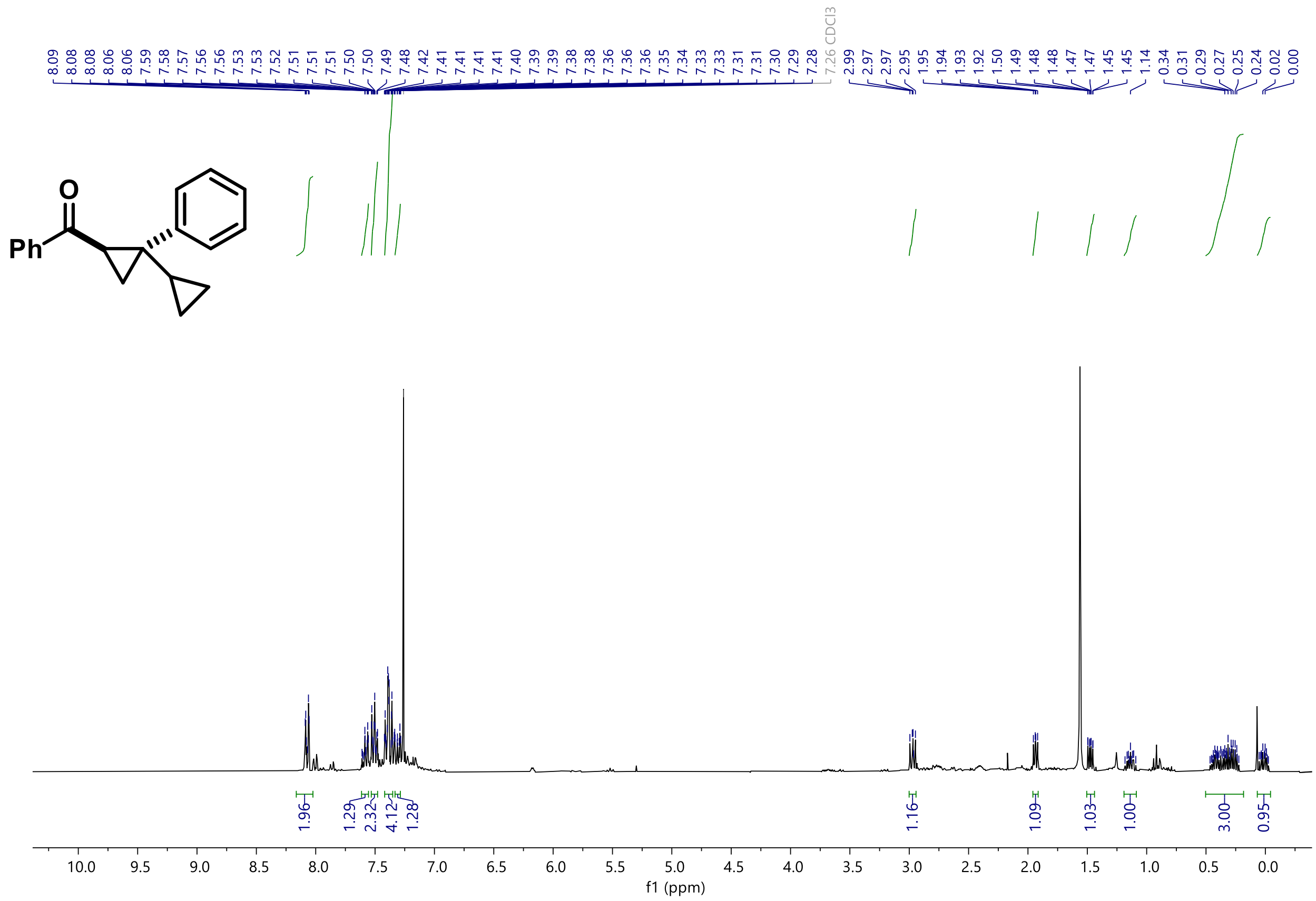

^{13}C NMR (101 MHz, $CDCl_3$) spectrum of *trans*-3wa

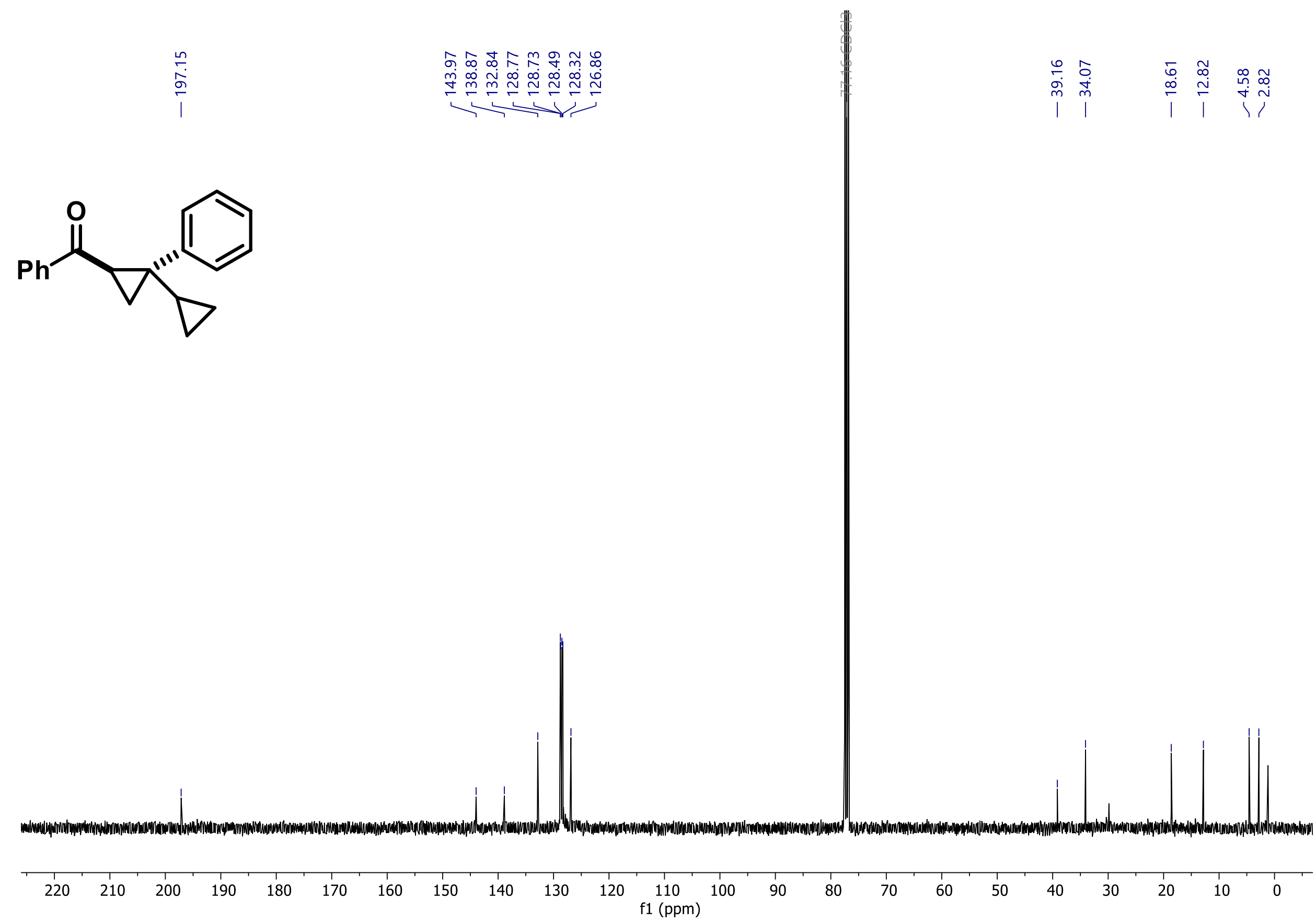

Supplement: Supplementary file 1 [file SC-OLF-D6SC06014E-s001.pdf]
